# 4,5-Dihydro-2*H*-pyridazin-3-ones as a Platform for the Construction of Chiral 4,4-Disubstituted-dihydropyridazin-3-ones †

**DOI:** 10.3390/molecules31010083

**Published:** 2025-12-24

**Authors:** Paul Joël Henry, Gabriel Burel, William Nzegge, Mario Waser, Jean-François Brière

**Affiliations:** 1 Institute of Organic Chemistry, Johannes Kepler University Linz, Altenbergerstrasse 69, 4040 Linz, Austria; paul.henry@insa-rouen.fr (P.J.H.);; 2 CNRS, INSA Rouen Normandie, Univ Rouen Normandie, Univ Caen Normandie, ENSICAEN, Institut CARMeN UMR 6064, F-76000 Rouen, France

**Keywords:** organic chemistry, Meldrum’s acid, asymmetric synthesis, phase-transfer catalysis, pyridazinones

## Abstract

4,5-Dihydro-2*H*-pyridazin-3-ones (DHPDOs) are important synthetic as well as naturally occurring heterocycles. We herein report the synthesis of various 4-monofunctionalized 4,5-dihydro-2*H*-pyridazin-3-ones and their use as starting materials to access 4,4-disubstituted dihydropyridazin-3-ones in an asymmetric fashion. By using chiral ammonium salt phase-transfer catalysts, conjugate additions of these scaffolds to classical acrylate-based Michael acceptors, as well as quinone methides, can be carried out with moderate to good enantioselectivities and in reasonable yields, affording a new pathway to dihydropyridazin-3-one derivatives with an all-carbon stereocenter.

## 1. Introduction

**Scheme 1:**
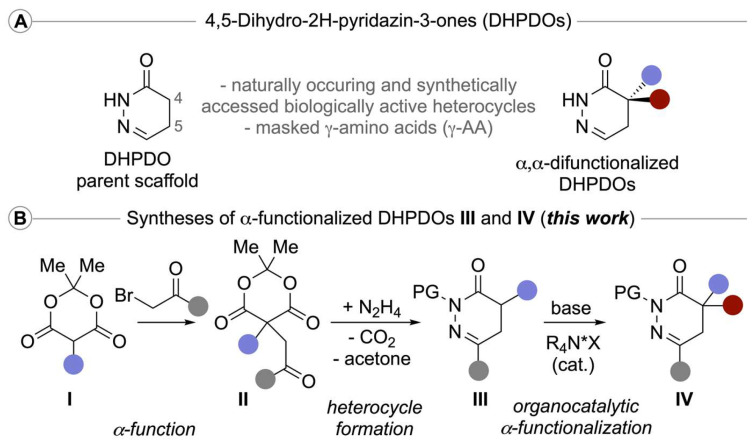
(**A**) General structures of DHPDOs and (**B**) the herein investigated strategy to access α-functionalized DHPDOs **III** and **IV** starting from Meldrum’s acid derivatives **I**.

The 4,5-dihydro-2*H*-pyridazin-3-one scaffold (DHPDO; Scheme 1A) represents an important structural motif that can be frequently found in a variety of synthetic and naturally occurring biologically active 6-membered *N-*containing heterocycles [1,2,3,4,5,6,7,8,9,10,11,12]. Not surprisingly, several research groups have developed catalytic asymmetric syntheses of DHPDO derivatives which are either mono-substituted at the C5- [13,14], or the C4-position (α-position of the carbonyl functionality) [15,16,17,18,19,20,21,22]. Furthermore, (chiral) substituted 4,5-dihydropyridazinone derivatives are useful building blocks and may be considered as masked γ-amino acid (γ-AA) derivatives [23,24], after reduction of the hydrazone moiety or cleavage of the N-N bond [16,25,26,27,28,29]. In that domain, several of these potentially medicinally interesting heterocycles contain one or two substituents at the 4-position [5,11]. However, to the best of our knowledge, the asymmetric catalytic elaboration of α,α-disubstituted DHPDOs, containing a quaternary stereocenter has not been covered yet. Thus, the development of reliable strategies to access different α-functionalized DHPDOs straightforwardly, even in an asymmetric manner, represents a worthwhile goal. Our groups have a longstanding interest in asymmetric synthesis and functionalization of chiral heterocycles, i.e., those that can be considered as masked α- and β-amino acids [30,31,32,33].

In line with our general research interests and the aforementioned value of chiral 4,5-dihydro-2*H*-pyridazin-3-ones, we now set out to establish versatile synthesis routes to access various α-functionalized DHPDOs **III** based on hydrazine and Meldrum’s acid chemistry, i.e., derivatives **I** [34,35,36,37] (Scheme 1). The easily accessible compounds **I** were believed to allow the elaboration of appropriately functionalized precursors **II**, which can be seen as 1,4-ketoester equivalents enabling the synthesis of DHPDOs **III** upon treatment with hydrazine (Scheme 1B). We herein furthermore report a strategy which demonstrates the organocatalytic asymmetric α-functionalization of DHPDO compounds **III** with different Michael-type acceptors under chiral ammonium salt phase-transfer catalytic conditions en route to the construction of new heterocyclic derivatives **IV** with an all-carbon stereocenter [38,39,40,41,42].

## 2. Results and Discussion

### 2.1. Syntheses of Pyridazinones

**Scheme 2:**
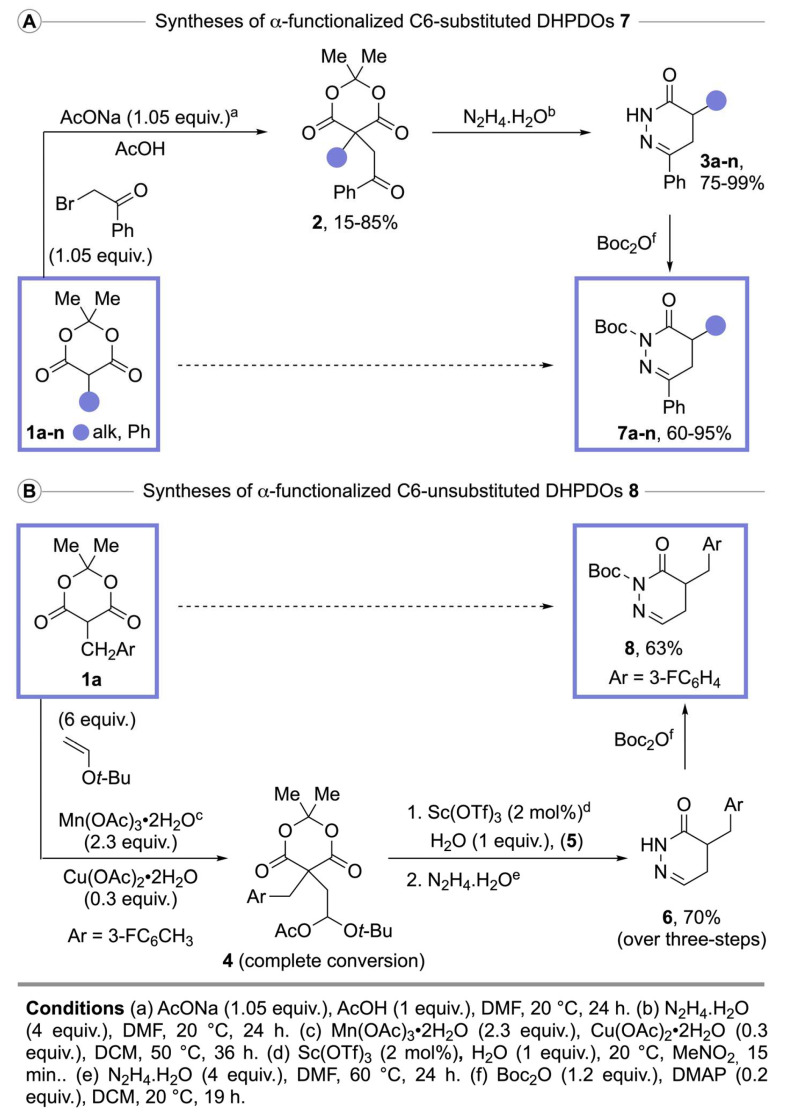
Elaboration of α-functionalized DHPDOs **7–8** starting from Meldrum’s acid derivatives, either (**A**) C6-substituted **7** or (**B**) C6-unsubstituted derivatives **8**.

At the onset, we sought a straightforward and versatile route to α-functionalized DHPDOs in order to allow the investigation of the catalytic asymmetric α-alkylation reactions (**III** to **IV**, Scheme 1). Inspired by the pioneering contribution of Tóth and collaborators [43], we exploited the readily available substituted Meldrum’s acid derivatives **1** as convenient precursors of 1,4-ketoester equivalents (Scheme 2) [11,44,45]. After an alkylation step of **1** with α-bromoacetophenone under basic conditions, producing access to products **2**, the facile condensation of hydrazine led to a series of free NH-containing DHPDOs **3** in good yields. On the other hand, in the purpose of expanding the scope of DHPDO substrates, we tackled the introduction of the CH_2_CH_2_CHO motif. However, this turned out to be not a trivial task on Meldrum’s acid derivatives **1**, according to the weak nucleophilic properties of its corresponding anion [34,35,36,37]. By revisiting Parsons’ conditions, in the presence of manganese(III) acetate as a single-electron oxidant, we found that tert-butyl vinyl ether smoothly underwent a radical addition of the model benzylated Meldrum’s acid derivative **1a,** yielding product **4** [46,47]. This *tert*-butyl derived acetal **4** was easily deprotected into the corresponding aldehyde **5** in 15 min using Sc(OTf)_3_ as a Lewis acid [48]. (We initially tried the commonly employed *n*-butyl vinyl ether as radical reaction partner [46], but the transformation proved to be less efficient than with *tert*-butyl vinyl ether, and the corresponding addition product was reluctant to be deprotected into aldehyde without leading to decomposition events under forced conditions). This rather unstable aldehyde has to be rapidly engaged in the subsequent condensation with hydrazine at 60 °C, affording the DHPDO **6** in excellent 70% yield over three steps. Eventually, in order to explore the reactivity of various molecular platforms, the *N*-Boc-protected DHPDOs **7** and **8** were also synthesized.

### 2.2. α-Functionalizations of Pyridazinones

**Figure 1:**
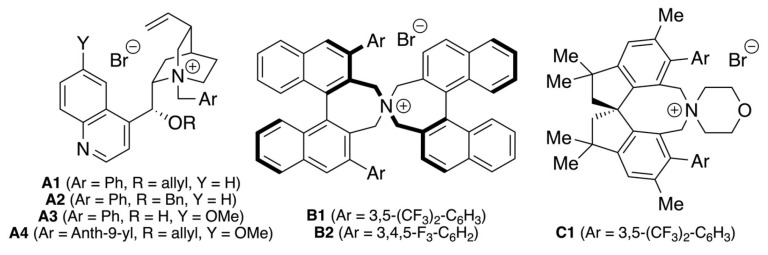
Chiral ammonium salt catalysts R_4_NX used herein.

With a robust route for the syntheses of differently functionalized pyridazinones **3** and **6**, as well as the *N*-protected derivatives **7** and **8** at hand, we next focused on the asymmetric α-functionalization of these heterocycles. We started our investigations by testing the quaternary ammonium salt-catalysed Michael addition of the parent compound **7a** to acrylate **9a** (Table 1 gives a condensed overview of the most significant results obtained by using the catalysts depicted in Figure 1).

First experiments using the well-established Cinchona alkaloid-based Quatsalts **A** showed that the 1,4-addition of **7a** to **9a** is possible in principle, but the enantioselectivities obtained for product **10a** were disappointing (entries 1–4) [38,39,40,41,42]. Also, varying the base and solvent did not allow us to achieve any notable levels of enantiodifferentiation (entries 5–7 give illustrative examples of a broader screening). Thus, we focused on alternative catalyst scaffolds. Unfortunately, the commercially available Maruoka catalysts **B** allowed for low conversions and low enantioselectivities only (entries 8 and 9; other conditions were tested as well, but did not lead to any improvement) [49].

Interestingly, the recently introduced spirobiindane-based salt **C1** was the only catalyst scaffold that gave reasonable conversion combined with somewhat more promising levels of enantioselectivities under the initially chosen conditions (entry 10) [50,51,52]. Variation of base and solvent (entries 10–13) revealed that Cs_2_CO_3_ in toluene allows for notable enantioselectivities (up to 90:10 e.r.), but conversion was slow (entry 13). While increased reaction times resulted in more practical rates of conversion, this was accompanied by a slight decrease in enantioselectivity (entries 13–15). Such a decrease in enantioselectivity with increased reaction progress can most likely be rationalized by a change of the catalyst’s integrity, i.e., its counter anion or also some catalyst degradation over the course of several catalyst turnovers. Using a slightly larger excess of base (3 eq.) at room temperature finally gave product **10a** with a high NMR yield of around 90% and a moderate enantioselectivity of 81:19 e.r. (entry 16). Unfortunately, the reaction became again much slower when carried out at 0 °C (entry 17). Noteworthy, the isolated yield of **10a** was lower than expected from the NMR yield (54% vs 90%; entry 16), which can be explained by a partial retro-Michael reaction of **10a** under the applied “classical” normal-phase silica gel column chromatography conditions (as observed by the formation of notable amounts of starting material **7a** again; this obstacle can to some extent be reduced, but not totally suppressed by using basified high-quality silica gel).

**Scheme 3:**
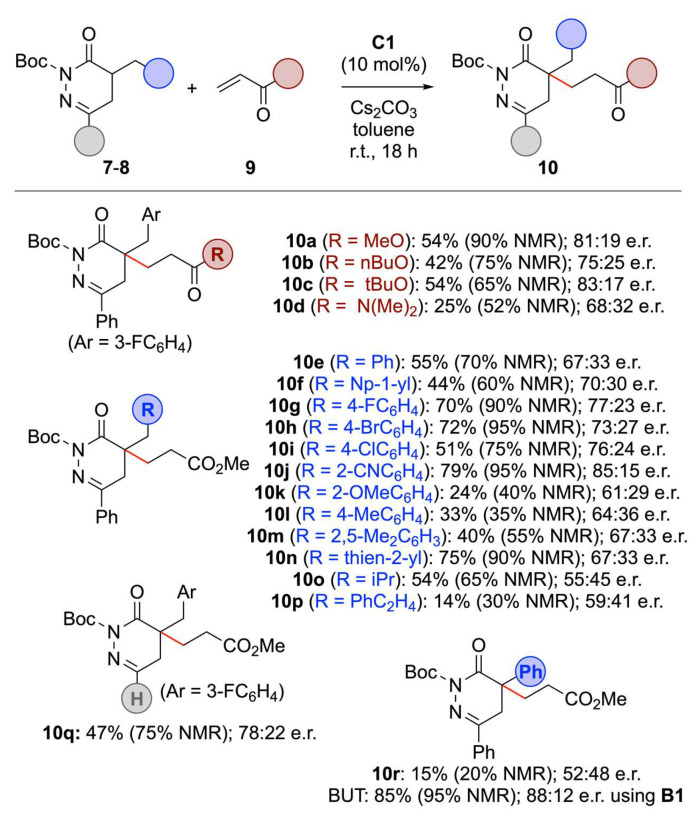
Application scope for the asymmetric 1,4-addition of compounds **7** and **8** to various acceptors **9**.

Having identified operationally reliable conditions that represent a reasonable compromise between conversion (yield) and enantioselectivity, we next investigated the generality of this protocol (Scheme 3). Testing different acrylates **9** first revealed that the ester functionality does not massively influence the outcome of this transformation (products **10a**–**c**). Interestingly, even the poorly electrophilic dimethylacrylamide was transformed into the corresponding Michael adduct **10d** in 32:68 er while in moderate NMR yield of 52%. Variations of the (hetero)benzylic α-substituents of starting materials **7** were investigated next (products **10e**–**k** and **10n**). Unfortunately, enantioselectivities were only modest as well, while conversion rates and yields somewhat depended on the nature of the benzylic groups (i.e., Me-containing electron-rich aryl groups led to lower yields as exemplified for products **10m** and **10n**). Other alkyl substituents were less well tolerated in terms of yield and e.r. (**10l**, **10p**).

The use of the 6-H containing DHPDO **8** was also reasonably well tolerated, producing product **10q** in comparable yield and enantiopurity as for the 4-Ph-substituted analogue **10a**. Furthermore, we also tested an α-Ph-containing DPHDO and hereby made an interesting observation (product **10r**). While the standard conditions using Quatsalt **C1** resulted in low yield and racemic product formation only, the use of Maruoka’s catalyst **B1** resulted in high yield and good e.r. of 88:12. This result, which is in clear contrast to the observations made when optimizing the parent reaction using α-alkylated pyridazinone **7a** (Table 1), clearly underscore the difficulties in predicting a suited catalyst for a given transformation and the importance of proper catalyst screening for differently substituted starting materials!

**Scheme 4:**
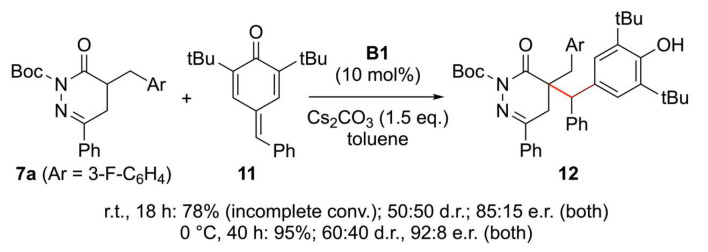
1,6-addition of compound **7a** to *p*-QM **11**.

In addition to using classical 1,4-acceptors **9**, we also tested if compounds **7** may undergo 1,6-additions to *para*-quinone methides (*p*-QMs) such as compound **11** (Scheme 4) [53,54,55,56]. We quickly realized that the addition of **7a** to **11** is a rather rapid and high-yielding process under quaternary ammonium salt catalysis conditions, affording the corresponding adduct **12** in up to 95% yield. When using chiral Quatsalts, the diastereoselectivity was always low (<60:40 d.r.)—this limitation is not rare. Reactions of enolate species to *p*-QMs could also not be overcome by any alteration of the reaction conditions. In contrast, enantioselectivities for both diastereomers were up to 92:8 e.r. when using Maruoka’s catalyst **B1** at 0 °C.

## 3. Experimental Details

Detailed experimental procedures and analytical details can be found in the online supporting information.

**General procedure for the synthesis of N-Boc 4,5-dihydropyridazinone derivatives 7.**
Compounds **2**: C5-monosubstituted Meldrum’s acid derivatives **1** (4.9 mmol), 2-bromo acetophenone (1.05 g, 5.3 mmol) and sodium acetate (435 mg, 5.3 mmol) were introduced into a round-bottom flask (N_2_ atmosphere). The mixture was dissolved in DMF (5 mL, 0.98 M), and acetic acid (0.29 mL, 4.9 mmol) was added dropwise at room temperature (vigorous stirring). After a night, the solvent was evaporated under reduced pressure. The crude product was dissolved in DCM and washed with a mixture of H_2_O/Na_2_CO_3_ (sat.) (9:1), and then with brine. The resulting organic phase was dried over anhydrous Na_2_SO_4_, filtrated, and finally the solvent was evaporated under reduced pressure. The crude reaction mixture was triturated with Et_2_O to give products **2a**–**o** (exact experimental and analytical details can be found in the online supporting information).
Compounds **3**: C5-disubstituted Meldrum’s acid derivative **2** (3.3 mmol) was solubilized in DMF (11 mL, 0.3 M) first (N_2_ atmosphere). At 0 °C, under vigorous stirring, hydrazine monohydrate (0.65 mL, 13.3 mmol) was added dropwise. The reaction was stirred for 24 h at room temperature, then the solvent was evaporated under reduced pressure. The crude was dissolved in DCM and washed with an aqueous acidic solution (pH 3–4) and then with brine. The combined organic phases were dried over anhydrous Na_2_SO_4_ and finally, the solvent was evaporated under reduced pressure, producing products **3** (exact experimental and analytical details can be found in the online supporting information).
Compounds **7**: Boc_2_O (938.5 mg, 4.3 mmol) and DMAP (87.5 mg, 716 µmol) were introduced in a round-bottom flask (N_2_ atmosphere). The N-unsubstituted 4,5-dihydropyridazinone **3** (3.58 mmol) was dissolved in anhydrous DCM (24 mL, 0.15 M) and introduced slowly into the round-bottom flask at room temperature. The solution was vigorously stirred overnight. Then, the solvent was evaporated under reduced pressure and the crude mixture was purified by silica gel column chromatography producing **7** (exact experimental and analytical details can be found in the online supporting information).
**General procedure for the quaternary ammonium salt-catalyzed Michael addition of pyridazinones to Michael acceptors.**
Spirobiindane-based ammonium salt **C1** (9.2 mg, 0.01 mmol) and Cs_2_CO_3_ (98 mg, 0.3 mmol) were introduced into a 2 mL vial at room temperature. The N-Boc dihydropyridazinone derivative **7** or **8** (0.1 mmol) was dissolved in toluene (1 mL, 0.1 M) and added to the vial followed by the Michael acceptor **9** (0.3 mmol). The mixture was stirred at room temperature for 18 h (1100 rpm). The crude mixture was filtered through a pad of silica gel using EtOAc as an eluent. Then, the crude mixture was purified by silica gel column chromatography producing the corresponding α,α-difunctionalized pyridazinone derivatives **10** (exact experimental and analytical details can be found in the online supporting information).

## 4. Conclusions

We herein introduced a straightforward route for the synthesis of various 4-monofunctionalized 4,5-dihydro-2*H*-pyridazin-3-ones. These heterocycles can then be employed as starting materials to access various 4,4-disubstituted dihydropyridazin-3-ones in an asymmetric fashion upon using chiral ammonium salt phase-transfer catalysts. More specifically, several conjugate addition reactions of these scaffolds to acrylate-based Michael acceptors (1,4-additions) as well as quinone methides (1,6-additions) were carried out with moderate to good enantioselectivities and in reasonable yields, allowing the construction and control of pyridazinone derivatives with an all-carbon stereocenter. Parts of this research have been presented at the 29th International Electronic Conference on Synthetic Organic Chemistry [57].

## Figures and Tables

**Table 1:** Optimization of the Quatsalt-catalysed Michael addition of pyridazinone **7a** to acrylate **9a**. ^1^

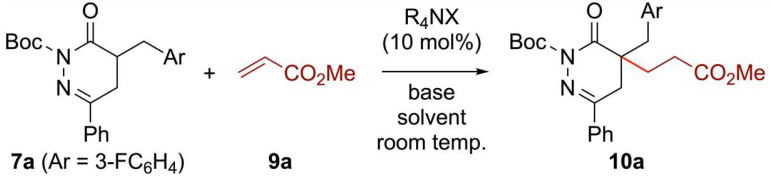							
**Entry**	**R_4_NX**	**Solvent**	**Base (Eq.)**	**t (h)**	**Conv. (%) ^2^**	**10a (%) ^3^**	**e.r. ^4^**
1	**A1**	CH_2_Cl_2_	Cs_2_CO_3_ (1.5)	3	70	70	55:45
2	**A2**	CH_2_Cl_2_	Cs_2_CO_3_ (1.5)	3	55	55	54:46
3	**A3**	CH_2_Cl_2_	Cs_2_CO_3_ (1.5)	3	10	10	n.d.
4	**A4**	CH_2_Cl_2_	Cs_2_CO_3_ (1.5)	3	90	85	54:46
5	**A4**	THF	Cs_2_CO_3_ (1.5)	3	100	95	52:48
6	**A4**	toluene	Cs_2_CO_3_ (1.5)	3	80	80	52:48
7	**A4**	CH_2_Cl_2_	K_3_PO_4_ (1.5)	3	40	35	50:50
8	**B1**	CH_2_Cl_2_	Cs_2_CO_3_ (1.5)	3	40	30	40:60
9	**B2**	CH_2_Cl_2_	Cs_2_CO_3_ (1.5)	3	40	35	43:57
10	**C1**	CH_2_Cl_2_	Cs_2_CO_3_ (1.5)	3	90	85	36:64
11	**C1**	CH_2_Cl_2_	K_3_PO_4_ (1.5)	3	25	25	36:64
12	**C1**	THF	Cs_2_CO_3_ (1.5)	3	95	50	42:58
13	**C1**	toluene	Cs_2_CO_3_ (1.5)	3	30	25	10:90
14	**C1**	toluene	Cs_2_CO_3_ (1.5)	18	80	75	15:85
15	**C1**	toluene	Cs_2_CO_3_ (1.5)	40	100	90	19:81
16	**C1**	toluene	Cs_2_CO_3_ (3)	18	100	90 (54)	19:81
17 ^5^	**C1**	toluene	Cs_2_CO_3_ (3)	18	40	40	10:90

^1^ All reactions were run using 0.1 mmol **7a**, 0.3 mmol **9a**, the indicated base and 10 mol% of the indicated catalyst in the given solvent (0.1 M) at room temperature unless otherwise stated. ^2^ Based on remaining **7a** calculated from the ^1^H NMR spectrum of the crude product using nitromethane as an internal standard (rounded in increments of 5%). ^3^ NMR yields using nitromethane as an internal standard (rounded in increments of 5%)—isolated yields given in brackets. ^4^ Determined by HPLC using a chiral stationary phase (given as ratio of (−):(+)-enantiomer ^5^ at 0 °C.

## Data Availability

The data underlying this study are available in the published article and its Supplementary Materials.

## Supplementary Materials

The following supporting information can be downloaded at: https://www.mdpi.com/article/10.3390/molecules31010083/s1, Full experimental details and characterization of intermediates and products. NMR spectra and HPLC traces. Reference [58] is cited in the supplementary materials.
