# Supplementary material for: 4,5-Dihydro-2H-pyridazin-3-ones as a Platform for the Construction of Chiral 4,4-Disubstituted-dihydropyridazin-3-ones
Source: Molecules. 2025 Dec 24;31(1):83. doi: 10.3390/molecules31010083 (PMC12786495; doi:10.3390/molecules31010083)
Supplement: Supplementary file 1 [file molecules-31-00083-s001.zip › molecules-4041431-supplementary.pdf]

Supporting Information

4,5-Dihydro-2H-pyridazin-3-ones as a Platform for the Construction of Chiral 4,4-Disubstituted-Dihydropyridazin-3-ones

Paul Joël Henry [1,2], Gabriel Burel [2], William Nzegge [1] , Mario Waser [1,*] and Jean-François Brière [2,*]

[1] Institute of Organic Chemistry, Johannes Kepler University Linz, Altenbergerstrasse 69, 4040 Linz, Austria; paul.henry@insa-rouen.fr (P.J.H.)

[2] CNRS, INSA Rouen Normandie, Univ Rouen Normandie, Univ Caen Normandie, ENSICAEN, Institut CARMeN UMR 6064, F-76000 Rouen, France; paul.henry@insa-rouen.fr (P.J.H.);

* Correspondence: mario.waser@jku.at (M.W.); jean-francois.briere@insa-rouen.fr (J.F.B.)

Contents

I. General information....2

II. Elaboration of pyridazinones....2

III. Quaternary ammonium salt-catalyzed a-functionalization of pyridazinones 21

IV. NMR spectra 31

IV. 1. 1. NMR spectra of C5-monosubstituted Meldrum's acid derivatives 331

IV. 1. 2. NMR spectra of C5-disubstituted Meldrum's acid derivatives....47

IV. 1. 3. NMR spectra of NH-pyridazinones....63

IV. 1. 4. NMR spectra of *N*-Boc pyridazinones....81

IV. 1. 5. NMR spectra of α-disubstituted pyridazinones 99

V. HPLC analyses 125

I. General information

Reactions were performed using oven dried glassware under inert atmosphere of nitrogen. Unless otherwise noted, all reagent-grade chemicals and solvents were obtained from commercial suppliers and were used as received. THF, toluene, MeCN and DCM were dried over MBRAUN MB SPS-800 Apparatus. Reactions were monitored by thin-layer chromatography with silica gel 60 F254 pre-coated aluminium plates (0.25 mm). Visualization was performed under UV light, phosphomolybdic acid or $KMnO_4$ oxidation. Chromatographic purification of compounds was achieved with 60 silica gel (40-63 µm). ^{1}H spectra (400 or 300 MHz) and ^{13}C NMR spectra (100 or 75 MHz) were recorded on a Bruker Avance 300 or NEO400As. Data appear in the following order: chemical shifts in ppm which were referenced to the internal solvent signal, integration value, multiplicity (*s*, singlet; *d*, doublet; *t*, triplet; *q*, quadruplet and combinations thereof; *m*, multiplet) and coupling constant *J* in Hertz. Semipreparative HPLC was carried out using a Dionex Ultimate 3000 system with variable wavelength detection and a Grace Alltima Silica 10 µm 250x10 mm column. Accurate Mass measurements (HRMS) were recorded with a Waters LCT 1er XR spectrometer or with an Agilent QTOF 6520 with ESI source. Chiral HPLC analyses were carried out on a Shimadzu Prominence HPLC system. HPLC analyses were performed with a CHIRALPAK® AD-H column (4.6 mm × 250 mm, 5 µm).

II. Elaboration of pyridazinones

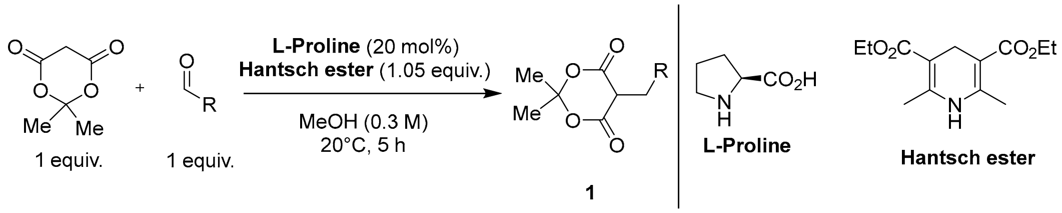

Typical procedure for C5-monosubstituted Meldrum's acid derivatives 1 preparation.[1] Meldrum's acid (2 g, 13.9 mmol), Hantzsch ester (3.6 g, 14.2 mmol) and L-Proline (320 mg, 2.78 mmol) were introduced in a round-bottom flask Under N_2 and dissolved in MeOH (0.3 M, 47 mL) at room temperature. Under stirring, the corresponding aldehyde (13.9 mmol) was added dropwise. After 5h of reaction at room temperature, the solvent was evaporated under reduce pressure. Excepted for products **1a** and **1b**, the crude mixture was purified by silica gel column chromatography to give the desired C5-monosubstituted Meldrum's acid derivatives **1c**-**n**.

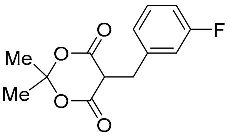

5-(3-fluorobenzyl)-2,2-dimethyl-1,3-dioxane-4,6-dione (1a) was synthesized following the general procedure using 3-fluorobenzaldehyde (0.98 mL, 13.9 mmol). After a recrystallization in hexane/acetone (9:1) followed by a wash with cold hexane, the compound **1a** (2.63 g, 75% yield) was isolated as white needles. mp = 95-97°C. ^{1}H NMR ($CDCl_3$, 300 MHz) δ_H 7.29-7.22 (1H, m), 7.12-7.03 (2H, m), 6.97-6.90 (1H, m), 3.75 ppm (1H, t, *J* = 5.0 Hz), 3.48 (2H, d, *J* = 5.0 Hz), 1.76 (3H, s), 1.58 (3H, s). ^{13}C{^{1}H} NMR ($CDCl_3$, 75 MHz) δ_C 165.1 (C), 162.8 (C, d, *J* = 244.5 Hz), 139.8 (C, d, *J* = 7.5 Hz), 130.2 (CH, d, *J* = 8.2 Hz), 125.6 (CH, d, *J* = 3 Hz), 116.9 (CH, d, *J* = 21.0 Hz), 114.3 (CH, d, *J* = 21.0 Hz), 105.4 (C), 48.1 (CH), 31.7 (CH_2), 28.6 (CH_3), 27.2 (CH_3). ^{19}F{^{1}H} NMR ($CDCl_3$, 282 MHz) δ_F -112.8. HRMS (ESI-): calcd for $C_{13}H_{12}FO_4$ [(M-H]$^-$): 251.0725; Found: 251.0728.

[1] Kishor, M.; Ramakumar, K.; Ramachary, D. B. *Tetrahedron Lett.* **2006**, *47*, 651.

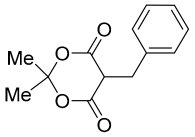

5-(3-fluorobenzyl)-2,2-dimethyl-1,3-dioxane-4,6-dione (**1b**) was synthesized following the general procedure benzaldehyde (1.42 mL, 13.9 mmol). The crude mixture was purified by silica gel column chromatography using DCM/heptane (7:3) as eluent giving the compound **1b** (1.2 g, 37% yield) as a white solid. ^{1}H NMR (CDCl$_3$, 300 MHz) δ_H 7.34-7.20 (5H, m), 3.76 (1H, t, J = 5.0 Hz), 3.49 (2H, d, J = 5.0 Hz), 1.73 (3H, s), 1.49 (3H, s). ^{13}C{^{1}H} NMR (CDCl$_3$, 75 MHz) δ_C 165.1 (C), 137.2 (C), 129.7 (CH), 128.6 (CH), 127.1 (CH), 105.3 (C), 48.4 (CH), 32.4 (CH$_2$), 28.8 (CH$_3$), 27.5 (CH$_3$). The analytical data match the published ones.[1]

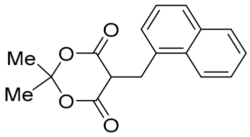

2,2-dimethyl-5-(naphthalen-1-ylmethyl)-1,3-dioxane-4,6-dione (**1c**) was synthesized following the general procedure using 1-naphtaldehyde (1.89 mL, 13.9 mmol). The crude mixture was purified by silica gel column chromatography using DCM/heptane (7:3) as eluent giving the compound **1c** (3.2 g, 81% yield) as a white solid. ^{1}H NMR (CDCl$_3$, 300 MHz) δ_H 8.08 (1H, d, J = 8.4 Hz), 7.91-7.88 (1H, m), 7.79 (1H, d, J = 8.3 Hz), 7.65 (1H, d, J = 6.5 Hz), 7.59-7.41 (3H, m), 3.93 (2H, d, J = 5.0 Hz), 3.80 (1H, t, J = 5.0 Hz), 1.70 (3H, s), 1.69 (3H, s). ^{13}C{^{1}H} NMR (CDCl$_3$, 75 MHz) δ_C 165.1 (C), 134.0 (C), 133.9 (C), 131.2 (C), 129.1 (CH), 128.3 (CH), 127.8 (CH), 126.7 (CH), 125.7 (CH), 125.5 (CH), 122.8 (CH), 105.2 (C), 48.0 (CH), 29.1 (CH$_2$), 28.9 (CH$_3$), 26.8 (CH$_3$). The analytical data match the published ones.[1]

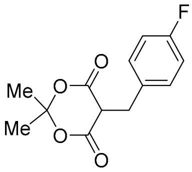

5-(4-fluorobenzyl)-2,2-dimethyl-1,3-dioxane-4,6-dione (**1d**) was synthesized following the general procedure using 4-fluorobenzaldehyde (1.5 mL, 13.9 mmol). After a recrystallization in hexane/acetone (9:1) followed by a wash with cold hexane, the compound **1d** was isolated (2.38 g, 68% yield) as white needles. ^{1}H NMR (CDCl$_3$, 300 MHz) δ_H 7.33-7.28 (2H, m), 7.01-6.93 (2H, m), 3.73 (1H, t, J = 4.9 Hz), 3.46 (2H, d, J = 4.7 Hz), 1.74 (3H, s), 1.54 (3H, s). ^{13}C{^{1}H} NMR (CDCl$_3$, 75 MHz) δ_C 165.3 (C), 162.1 (C, d, J = 183.0 Hz), 132.9 (C, d, J = 2.2 Hz), 131.7 (CH), 131.6 (CH); 115.6 (CH), 115.4 (CH), 105.4 (C), 48.3 (CH), 31.4 (CH$_2$), 28.5 (CH$_3$), 27.4 (CH$_3$). ^{19}F{^{1}H} NMR (CDCl$_3$, 282 MHz) δ_F -115.4. The analytical data match the published ones.[1]

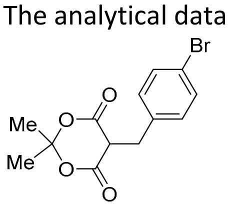

5-(4-bromobenzyl)-2,2-dimethyl-1,3-dioxane-4,6-dione (**1e**) was synthesized following the general procedure using 4-bromobenzaldehyde (2.57, 13.9 mmol). The crude mixture was purified by silica gel column chromatography using DCM/heptane (7:3) as eluent giving the compound **1e** (3.75 g, 86% yield) as a white solid. ^{1}H NMR (CDCl$_3$, 300 MHz) δ_H 7.43-7.38 (2H, m), 7.23- 7.19 (2H, m), 3.74 (1H, t, J = 5.0 Hz), 3.43 (2H, d, J = 5.0 Hz), 1.74 (3H, s), 1.59 (3H, s). ^{13}C{^{1}H} NMR (CDCl$_3$, 75 MHz) δ_C 165.1 (C), 136.2 (C), 131.8 (4 CH), 121.4 (C), 105.4 (C), 48.0 (CH), 31.4 (CH$_2$), 28.5 (CH$_3$), 27.2 (CH$_3$). The analytical data match the published ones.[1]

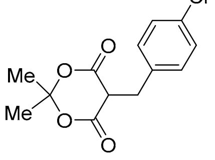

5-(4-chlorobenzyl)-2,2-dimethyl-1,3-dioxane-4,6-dione (**1f**) was synthesized following the general procedure using 4-chlorobenzaldehyde (1.96 g, 13.9 mmol). The crude mixture was purified by silica gel column chromatography using DCM/heptane (7:3) as eluent giving the compound **1f** (2.95 g, 79% yield) as a white solid. ^{1}H NMR ($CDCl_3$, 300 MHz) δ_H 7.29-7.23 (4H, m), 3.74 (1H, t, J = 4.9 Hz), 3.45 (2H, d, J = 4.9 Hz), 1.75 (3H, s), 1.58 (3H, s). ^{13}C{^{1}H} NMR ($CDCl_3$, 75 MHz) δ_C 165.2 (C), 135.7 (C), 133.2 (C), 131.4 (CH), 128.8 (CH), 105.4 (C), 48.1 (CH), 31.4 (CH_2), 28.5 (CH_3), 27.3 (CH_3). The analytical data match the published ones.[1]

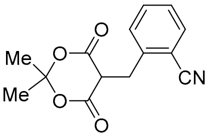

2-((2,2-dimethyl-4,6-dioxo-1,3-dioxan-5-yl)methyl)benzonitrile (**1g**) was synthesized following the general procedure using 2-cyanobenzaldehyde (1.82 g, 13.9 mmol). The crude mixture was purified by silica gel column chromatography using DCM/heptane (7:3) as eluent giving the compound **1g** (2.63 g, 73% yield) as a white solid. mp = 139-141°C. ^{1}H NMR ($CDCl_3$, 300 MHz) δ_H 7.65-7.63 (1H, m), 7.58-7.50 (2H, m), 7.38-7.32 (1H, m), 4.03 (1H, t, J = 6.0 Hz), 3.63 (2H, d, J = 6.0 Hz), 1.83 (3H, s), 1.77 (3H, s). ^{13}C{^{1}H} NMR ($CDCl_3$, 75 MHz) δ_C 164.1 (C), 141.3 (C), 132.9 (CH), 132.8 (CH), 130.6 (CH), 127.5 (CH), 117.8 (C), 112.9 (C), 105.4 (C), 47.2 (CH), 30.5 (CH_2), 28.9 (CH_3), 26.7 (CH_3). HRMS (ESI-): calcd for $C_{14}H_{12}NO_4$ [(M-H)$^-$]: 258.0772; Found: 258.0775.

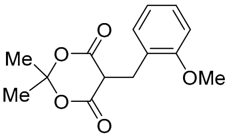

5-(2-methoxybenzyl)-2,2-dimethyl-1,3-dioxane-4,6-dione (**1h**) was synthesized following the general procedure using 2-methoxybenzaldehyde (1.89 g, 13.9 mmol). The crude mixture was purified by silica gel column chromatography using DCM/heptane (7:3) as eluent giving the compound **1h** (1.84 g, 50% yield) as a white solid. mp = 96-98°C. ^{1}H NMR ($CDCl_3$, 300 MHz) δ_H 7.37-7.34 (1H, m), 7.27-7.22 (1H, m), 6.96-6.84 (2H, m), 4.02 (1H, t, J = 5.9 Hz), 3.83 (3H, s), 3.40 (2H, d, J = 5.8 Hz), 1.83 (3H, s), 1.77 (3H, s), 1.72 (3H, s). ^{13}C{^{1}H} NMR ($CDCl_3$, 75 MHz) δ_C 165.1 (C), 157.0 (C), 131.8 (CH), 128.2 (CH), 125.7 (C), 120.6 (CH), 110.2 (CH), 104.9 (C), 55.4 (CH_3), 46.4 (CH), 29.0 (CH_3), 28.3 (CH_2), 26.9 (CH_3). HRMS (ESI-): calcd for $C_{14}H_{15}O_5$ [(M-H)$^-$]: 263.0925; Found: 263.0932.

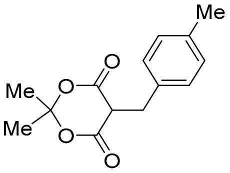

2,2-dimethyl-5-(4-methylbenzyl)-1,3-dioxane-4,6-dione (**1i**) was synthesized following the general procedure using 4-methylbenzaldehyde (1.64 mL, 13.9 mmol). The crude mixture was purified by silica gel column chromatography using DCM/heptane (7:3) as eluent giving the compound **1i** (1.79 g, 52% yield) as a white solid. ^{1}H NMR ($CDCl_3$, 300 MHz) δ_H 7.22-7.19 (2H, m), 7.11-7.08 (2H, m), 3.74 (1H, t, J = 4.9 Hz), 3.45 (2H, d, J = 4.9 Hz), 2.30 (3H, s), 1.72 (3H, s), 1.50 (3H, s). ^{13}C{^{1}H} NMR ($CDCl_3$, 75 MHz) δ_C 165.2 (C), 136.7 (C), 134.0 (C), 129.6 (CH), 129.2 (CH), 105.2 (C), 48.5 (CH), 32.1 (CH_2), 28.7 (CH_3), 27.5 (CH_3), 21.4 (CH_3). The analytical data match the published ones.[1]

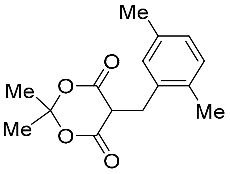

5-(2,5-dimethylbenzyl)-2,2-dimethyl-1,3-dioxane-4,6-dione (**1j**) was synthesized following the general procedure using 2,5-dimethylbenzaldehyde (1.97 mL, 13.9 mmol). The crude mixture was purified by silica gel column chromatography using DCM/heptane (7:3) as eluent giving the compound **1j** (2.15 g, 59% yield) as a white solid. mp = 110-115°C. ^{1}H NMR ($CDCl_3$, 300 MHz) δ_H 7.12-6.96 (3H, m), 3.69 (1H, t, *J* = 5.2 Hz), 3.43 (2H, d, *J* = 5.2 Hz), 2.34 (3H, s), 2.30 (3H, s); 1.78 (3H, s), 1.69 (3H, s). ^{13}C{^{1}H} NMR ($CDCl_3$, 75 MHz) δ_C 165.1 (C), 136.1 (C), 135.5 (C), 133.1 (C), 130.4 (CH), 130.1 (CH), 127.7 (CH), 105.1 (C), 47.9 (CH), 29.0 (CH_2), 28.9 (CH_3), 27.0 (CH_3), 21.3 (CH_3), 19;4 (CH_3). HRMS (ESI-): calcd for $C_{15}H_{17}O_4$ [(M-H]$^-$): 261.1132; Found: 261.1138.

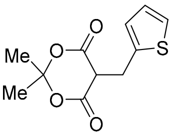

2,2-dimethyl-5-(thiophen-2-ylmethyl)-1,3-dioxane-4,6-dione (**1k**) was synthesized following the general procedure using 2-thiophenecarboxaldehyde (1.3 mL, 13.9 mmol). The crude mixture was purified by silica gel column chromatography using DCM/heptane (7:3) as eluent giving the compound **1k** (1.9 g, 57% yield) as a white solid. ^{1}H NMR ($CDCl_3$, 300 MHz) δ_H 7.14 (1H, dd, *J* = 5.1, 1.2 Hz), 6.97-6.96 (1H, m), 6.90 (1H, dd, *J* = 5.1, 3.5 Hz), 3.79 (1H, t, *J* = 4.6 Hz), 3.67 (2H, d, *J* = 4.7 Hz), 1.75 (3H, s), 1.58 (3H, s). ^{13}C{^{1}H} NMR ($CDCl_3$, 75 MHz) δ_C 165.0 (C), 138.3 (C), 128.0 (CH_3), 127.0 (CH), 125.1 (CH), 105.4 (C), 48.3 (CH), 28.5 (CH_3), 27.3 (CH_3), 26.5 (CH_2). The analytical data match the published ones.[1]

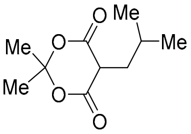

5-isobutyl-2,2-dimethyl-1,3-dioxane-4,6-dione (**1l**) was synthesized following the general procedure using isobutyraldehyde (1.27 mL, 13.9 mmol). The crude mixture was purified by silica gel column chromatography using DCM/heptane (7:3) as eluent giving the compound **1m** (2.11 g, 76% yield) as a white solid. ^{1}H NMR ($CDCl_3$, 300 MHz) δ_H 3.43 (1H, t, *J* = 5.5 Hz), 2.09-1.95 (3H, m), 1.79 (3H, s), 1.75 (3H, s); 0.95 (6H, d, *J* = 6.3 Hz). ^{13}C{^{1}H} NMR ($CDCl_3$, 75 MHz) δ_C 166.1 (C), 105.0 (C), 44.3 (CH), 35.5 (CH_2), 28.7 (CH_3), 27.0 (CH_3), 26.1 (CH), 22.2 (CH_3). The analytical data match the published ones.[1]

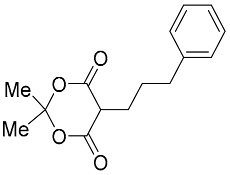

2,2-dimethyl-5-(3-phenylpropyl)-1,3-dioxane-4,6-dione (**1m**) was synthesized following the general procedure using 3-phenypropionaldehyde (1.85 mL, 13.9 mmol). The crude mixture was purified by silica gel column chromatography using DCM/heptane (7:3) as eluent giving the compound **1l** (2.77 g, 76% yield) as a white solid. ^{1}H NMR ($CDCl_3$, 300 MHz) δ_H 7.31-7.15 (5H, m), 3.48 (1H, t, *J* = 5.1 Hz), 2.69 (2H, t, *J* = 7.7 Hz), 2.20-2.13 (2H, m), 1.87-1.79 (2H, m), 1.74 (6H, s). ^{13}C{^{1}H} NMR ($CDCl_3$, 75 MHz) δ_C 165.2 (C), 141.4 (C), 128.3 (CH), 125.9 (CH), 104.8 (C), 46.3 (CH), 36.0 (CH_2), 28.7 (CH_3), 28.4 (CH_2), 27.1 (CH_3), 26.6 (CH_2). The analytical data match the published ones.[1]

2,2-dimethyl-5-phenyl-1,3-dioxane-4,6-dione (**1n**)[2]

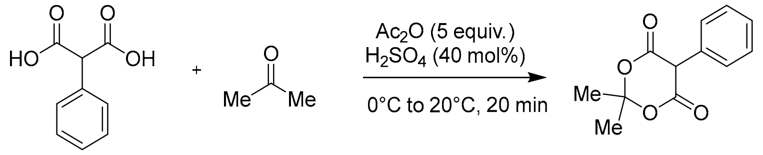

phenylmalonic acid (1 equiv.) **acetone** (1.6 equiv.) **1n**, 57%

Under N_2, in a round-bottom flask, phenylmalonic acid (10 g, 55.5 mmol) and acetic anhydride (34.7 mL, 278 mmol) were introduced. The mixture was cooled at 0°C using an ice bath and under stirring, concentrated sulfuric acid (1.2 mL, 22.5 mmol) and acetone (6.5 mL, 88.5 mmol) were added dropwise. The solution was warmed to room temperature and after 20 minutes, the mixture was filtered. The solid was solubilized in DCM, washed with a brine solution and dried over anhydrous Na_2SO_4. The solution was filtered, and the solvent was evaporated under reduce pressure. The resulting solid was triturated in a mixture *n*-hexane/AcOEt (7:3) to recover C5-phenyl Meldrum's acid **1n** (6.97 g, 57% yield) as a white powder. ^{1}H NMR ($CDCl_3$, 300 MHz) δ_H 7.47-7.26 (5H, m), 4.77 (1H, s), 1.88 (3H, s), 1.76 (3H, s). $^{13}C\{^1H\}$ NMR ($CDCl_3$, 75 MHz) δ_C 164.9 (C), 130.7 (C), 129.3 (2 CH), 129.3 (2 CH), 128.9 (CH), 105.9 (C), 52.9 (CH_3), 28.7 (CH_3), 27.6 (CH_3). The analytical data match the published ones.[2]

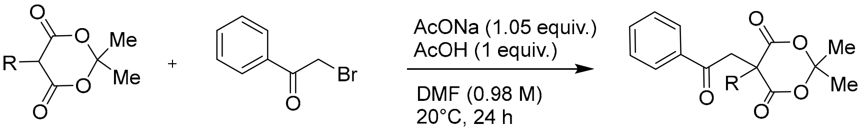

1 (1 equiv.) **2-bromoacetophenone** (1.05 equiv.) **2**

Typical procedure for the synthesis of C5-disubstituted Meldrum's acid derivatives 2.[3] Under N_2, C5-monosubstituted Meldrum's acid **1** (4.9 mmol), 2-bromo acetophenone (1.05 g, 5.3 mmol) and sodium acetate (435 mg, 5.3 mmol) were introduced in a round-bottom flask. The mixture was dissolved in DMF (5 mL, 0.98 M) and under vigorous stirring, acetic acid (0.29 mL, 4.9 mmol) was added dropwise at room temperature. After a night, the solvent was evaporated under reduce pressure. The crude was dissolved in DCM and washed with a mixture $H_2O/Na_2CO_{3\,sat.}$ (9:1) and then with a brine solution. The resulting organic phase was dried over anhydrous Na_2SO_4, filtrated, and finally, the solvent was evaporated under reduce pressure. The crude reaction mixture was triturated in Et_2O to give products **2a**-**o**.

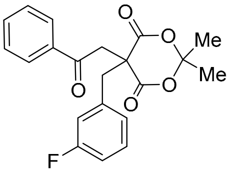

5-(3-fluorobenzyl)-2,2-dimethyl-5-(2-oxo-2-phenylethyl)-1,3-dioxane-4,6-dione (**2a**) was synthesized following the general procedure using C5-monosubstituted Meldrum's acid **1a** (1.24 g, 4.9 mmol). The trituration process gave the compound **2a** (1.32 g, 73% yield) as a white powder. ^{1}H NMR ($CDCl_3$, 300 MHz) δ_H 7.97-7.93 (2H, m), 7.64-7.58 (1H, m), 7.50-7.45 (2H, m), 7.34-7.26 (1H, m), 7.05-6.91 (3H, m), 4.05 (2H, s), 3.30 (2H, s), 2.00 (3H, s), 0.82 (3H, s). $^{13}C\{^1H\}$ NMR ($CDCl_3$, 75 MHz) δ_C 196.0 (C), 167.7 (C), 162.7 (C, d, J = 245.2 Hz), 135.9 (C, d, *J* = 7.5 Hz), 134.8 (C), 134.1 (CH), 130.4 (CH, d, *J* = 7.5 Hz), 128.7 (CH), 128.3 (CH), 125.9 (CH, d, *J* = 3.0 Hz), 117.3 (CH, d, *J* = 21.0 Hz), 115.4 (CH, d, *J* = 21.0 Hz), 107.7 (C), 52.0 (C), 48.2 (CH_2), 45.0 (CH_2, d, *J* = 1.5 Hz), 28.7 (CH_3), 28.6 (CH_3). $^{19}F\{^1H\}$ NMR ($CDCl_3$, 282 MHz) δ_F -112.0. HRMS (ESI-): calcd m/z for $C_{17}H_{12}FO_2$ [$(M-H-CO_2-C_3H_6O)^-$]: 267,0827; Found: 267.0826.

[2] Chidipudi, S. R.; Khan, I.; Lam, H. W. *Angew. Chem. Int. Ed.* **2012**, *51*, 12115.

[3] Tóth, G.; Molnár, S.; Tivadar Tamás, T.; Borbély, I. *Synth. Commun.* **2006**, *27*, 20, 3513.

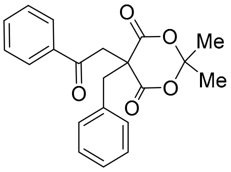

5-benzyl-2,2-dimethyl-5-(2-oxo-2-phenylethyl)-1,3-dioxane-4,6-dione (**2b**) was synthesized following the general procedure using C5-monosubstituted Meldrum's acid **1b** (1.15 g, 4.9 mmol). The trituration process gave the compound **2b** (1.22 g, 71% yield) as a white powder. ^{1}H NMR ($CDCl_3$, 300 MHz) δ_H 7.97-7.94 (2H, m); 7.63-7.58 (1H, m), 7.50-7.45 (2H, m), 7.37-7.30 (3H, m), 7.22-7.19 (2H, m), 4.06 (2H, s), 3.32 (2H, s), 1.97 (3H, s), 0.71 (3H, s). ^{13}C{^{1}H} NMR ($CDCl_3$, 75 MHz) δ_C 196.2 (C), 168.0 (C), 134.9 (C), 134.0 (CH), 133.6 (C), 130.1 (CH), 128.9 (CH), 128.7 (CH), 128.3 (CH), 128.2 (CH), 107.6 (C), 52.2 (C), 48.2 (CH_2), 45.5 (CH_2), 28.8 (CH_3), 28.4 (CH_3). HRMS (ESI-): calcd m/z for $C_{17}H_{13}O_2$ [(M-H-CO_2-C_3H_6O)$^-$]: 249.0921; Found: 249.0922.

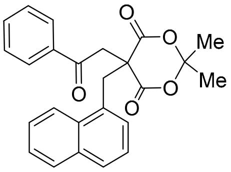

2,2-dimethyl-5-(naphthalen-1-ylmethyl)-5-(2-oxo-2-phenylethyl)-1,3-dioxane-4,6-dione (**2c**) was synthesized following the general procedure using C5-monosubstituted Meldrum's acid **1c** (1.4 g, 4.9 mmol). The trituration process gave the compound **2c** (926.8 mg, 47% yield) as a yellowish powder. ^{1}H NMR ($CDCl_3$, 300 MHz) δ_H 8.06-7.96 (3H, m), 7.88-7.78 (2H, m), 7.62-7.41 (7H, m), 4.19 (2H, s), 3.85 (2H, s), 1.88 (3H, s), 0.42 (3H, s). ^{13}C{^{1}H} NMR ($CDCl_3$, 75 MHz) δ_C 196.3 (CH_3), 168.3 (C), 135.0 (C), 134.0 (CH), 133.7 (C), 132.2 (C), 130.1 (C), 129.3 (CH), 128.9 (CH), 128.7 (CH), 128.6 (CH), 128.3 (CH), 126.8 (CH), 126.1 (CH), 125.4 (CH), 123.4 (CH), 107.4 (C), 52.0 (C), 48.2 (CH_2), 40.9 (CH_2), 28.8 (CH_3), 28.0 (CH_3). HRMS (ESI-): calcd m/z for $C_{21}H_{15}O_2$ [(M-H-CO_2-C_3H_6O)$^-$]: 299,1078; Found: 299,1080.

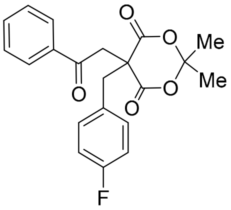

5-(4-fluorobenzyl)-2,2-dimethyl-5-(2-oxo-2-phenylethyl)-1,3-dioxane-4,6-dione (**2d**) was synthesized following the general procedure using C5-monosubstituted Meldrum's acid **1d** (1.24 g, 4.9 mmol). The trituration process gave the compound **2d** (1.14 g, 63% yield) as a white powder. ^{1}H NMR ($CDCl_3$, 300 MHz) δ_H 7.96-7.93 (2H, m), 7.63-7.58 (1H, m), 7.50-7.45 (2H, m), 7.20-7.15 (2H, m), 7.06-7.00 (2H, m), 4.04 (2H, s), 3.29 (2H, s), 1.99 (3H, s), 0.82 (3H, s). ^{13}C{^{1}H} NMR ($CDCl_3$, 75 MHz) δ_C 196.5 (C), 168.2 (C), 162.9 (C, d, J = 246.0 Hz), 135.0 (C), 134.3 (CH), 132.1 (CH), 132.0 (CH), 129.62 (C, d, J = 3.0 Hz), 128.9 (CH), 128.5 (CH), 116.2 (CH), 115.9 (CH), 107.8 (C), 52.0 (C, d, J = 1.5 Hz), 48.0 (CH_2), 44.5 (CH_2), 28.5 (CH_3), 28.5 (CH_3). ^{19}F{^{1}H} NMR ($CDCl_3$, 282 MHz) δ_F -113.5. HRMS (ESI-): calcd m/z for $C_{17}H_{12}FO_2$ [(M-H-CO_2-C_3H_6O)$^-$]: 267.0827; Found: 267.0828.

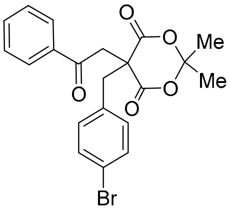

5-(4-bromobenzyl)-2,2-dimethyl-5-(2-oxo-2-phenylethyl)-1,3-dioxane-4,6-dione (**2e**) was synthesized following the general procedure using C5-monosubstituted Meldrum's acid **1e** (1.54 g, 4.9 mmol). The trituration process gave the compound **2e** (1.61 g, 76% yield) as a white powder. ^{1}H NMR ($CDCl_3$, 300 MHz) δ_H 7.96- 7.93 (2H, m), 7.63-7.58 (1H, m), 7.50-7.45 (4H, m), 7.09-7.06 (2H, m), 4.03 (2H, s), 3.26 (2H, s), 2.00 (3H, s), 0.85 (3H, s). ^{13}C{^{1}H} NMR ($CDCl_3$, 75 MHz) δ_C 196.5 (C), 168.1 (C), 135.0

(C), 134.3 (CH), 132.7 (C), 132.2 (CH), 132.0 (CH), 128.9 (CH), 128.5 (CH), 122.6 (C), 107.8 (C), 51.9 (C), 47.9 (CH_2), 44.7 (CH_2), 28.6 (CH_3), 28.5 (CH_3). HRMS (ESI-): calcd m/z for $C_{17}H_{12}BrO_2$ [$(M-H-CO_2-C_3H_6O)^-$]: 327.0026; Found: 327.0031.

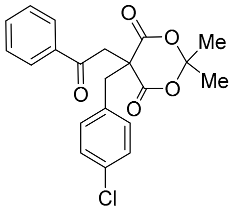

5-(4-chlorobenzyl)-2,2-dimethyl-5-(2-oxo-2-phenylethyl)-1,3-dioxane-4,6-dione (**2f**) was synthesized following the general procedure using C5-monosubstituted Meldrum's acid **1f** (1.32 g, 4.9 mmol). The trituration process gave the compound **2f** (1.31 g, 69% yield) as a white powder. ^{1}H NMR ($CDCl_3$, 300 MHz) δ_H 7.96-7.93 (2H, m), 7.63-7.58 (1H, m), 7.50-7.45 (2H, m), 7.33- 7.29 (2H, m), 7.15- 7.12 (2H, m), 4.04 (2H, s), 3.27 (2H, s), 2.00 (3H, s), 0.84 (3H, s). ^{13}C{^{1}H} NMR ($CDCl_3$, 75 MHz) δ_C 196.5 (C), 168.1 (C), 135.0 (C), 134.6 (C), 134.3 (CH), 132.2 (C), 131.7 (CH), 129.2 (CH), 128.9 (CH), 128.5 (CH), 107.8 (C), 51.9 (C), 47.9 (CH_2), 44.6 (CH_2), 28.6 (CH_3), 28.5 (CH_3). The analytical data match the published ones.[3]

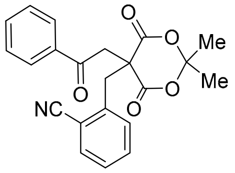

2-((2,2-dimethyl-4,6-dioxo-5-(2-oxo-2-phenylethyl)-1,3-dioxan-5-yl)methyl)benzonitrile (**2g**) was synthesized following the general procedure using C5-monosubstituted Meldrum's acid **1g** (1.27 g, 4.9 mmol). The trituration process gave the compound **2g** (536.3 mg, 29% yield) as a white powder. ^{1}H NMR ($CDCl_3$, 300 MHz) δ_H 7.95-7.92 (2H, m), 7.72-7.69 (1H, m), 7.62-7.57 (2H, m), 7.49-7.42 (3H, m), 7.38-7.36 (1H, m), 4.05 (2H, s), 3.55 (2H, s), 2.06 (3H, s), 1.31 (3H, s). ^{13}C{^{1}H} NMR ($CDCl_3$, 75 MHz) δ_C 196.0 (C), 167.0 (C), 137.1 (C), 134.8 (C), 134.1 (CH), 133.4 (CH), 132.8 (CH), 131.7 (CH), 128.8 (CH), 128.7 (CH), 128.3 (CH), 117.3 (C), 114.7 (C), 107.6 (C), 51.6 (C), 46.2 (CH_2), 42.1 (CH_2), 29.0 (2 CH_3). HRMS (ESI-): calcd m/z for $C_{22}H_{22}N_2O_5$ [$(M-H+NH_4)^-$]: 394.1528; Found: 394.1594.

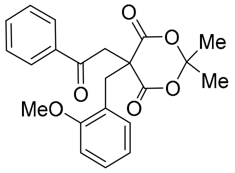

5-(2-methoxybenzyl)-2,2-dimethyl-5-(2-oxo-2-phenylethyl)-1,3-dioxane-4,6-dione (**2h**) was synthesized following the general procedure using C5-monosubstituted Meldrum's acid **1h** (1.3 g, 4.9 mmol). The trituration process gave the compound **2h** (506 mg, 27% yield) as a white powder. ^{1}H NMR ($CDCl_3$, 300 MHz) δ_H 7.90-7.88 (2H, m), 7.56-7.51 (1H, m), 7.43-7.38 (2H, m), 7.24-7.21 (1H, m), 7.13-7.11 (1H, m), 6.91-6.81 (2H, m), 3.95 (2H, s), 3.74 (3H, s), 3.31 (2H, s), 1.94 (3H, s), 1.05 (3H, s). ^{13}C{^{1}H} NMR ($CDCl_3$, 75 MHz) δ_C 196.9 (C), 168.2 (C), 158.4 (C), 135.4 (C), 134.0 (CH), 132.1 (CH), 129.7 (CH), 128.8 (CH), 128.4 (CH), 122.4 (C), 120.8 (CH), 110.8 (CH), 107.1 (C), 55.3 (CH_3), 51.2 (C), 46.9 (CH_2), 38.8 (CH_2), 29.0 (CH_3), 28.2 (CH_3). HRMS (ESI-): calcd m/z for $C_{18}H_{17}O_4$ [$(M-H-CO_2-C_3H_6O+H_2O)^-$]: 297.1132; Found: 297.1136.

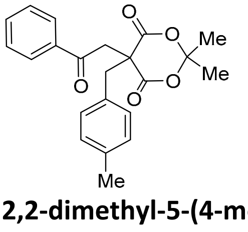

2,2-dimethyl-5-(4-methylbenzyl)-5-(2-oxo-2-phenylethyl)-1,3-dioxane-4,6-dione (**2i**) was synthesized following the general procedure using C5-monosubstituted Meldrum's acid **1i** (1.22 g, 4.9 mmol). The trituration process gave the compound **2i** (1.26 g, 70% yield) as a white powder. ^{1}H NMR ($CDCl_3$, 300 MHz) δ_H) 7.96-7.93 (2H, m), 7.63-7.57 (1H, m), 7.50-7.45 (2H, m), 7.15-7.07 (4H, m), 4.04

(2H, s), 3.28 (2H, s), 2.32 (3H, m), 1.98 (3H, s), 0.76 (3H, s). $^{13}C\{^1H\}$ NMR ($CDCl_3$, 75 MHz) δ_C 196.3 (C), 168.0 (C), 138.0 (C), 134.9 (C), 134.0 (CH), 130.4 (C), 130.0 (CH), 129.5 (CH), 128.7 (CH), 128.3 (CH), 107.6 (C), 52.2 (C), 48.1 (CH_2), 46.1 (CH_2), 28.8 (CH_3), 28.3 (CH_3), 21.4 (CH_3). HRMS (ESI-): calcd m/z for $C_{18}H_{15}O_2$ [$(M-H-CO_2-C_3H_6O)^-$]: 263.1078; Found: 263.1077.

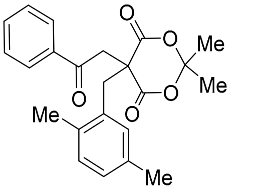

5-(2,5-dimethylbenzyl)-2,2-dimethyl-5-(2-oxo-2-phenylethyl)-1,3-dioxane-4,6-dione (**2j**) was synthesized following the general procedure using C5-monosubstituted Meldrum's acid **1j** (1.29 g, 4.9 mmol). The trituration process gave the compound **2j** (801.6 mg, 43% yield) as a white powder. ^1H NMR ($CDCl_3$, 300 MHz) δ_H 7.98-7.95 (2H, m), 7.63-7.58 (1H, m), 7.50-7.45 (2H, m), 7.08-6.97 (3H, m), 4.07 (2H, s), 3.36 (2H, s), 2.27 (3H, s), 2.26 (3H, s), 1.98 (3H, s), 0.81 (3H, s). $^{13}C\{^1H\}$ NMR ($CDCl_3$, 75 MHz) δ_C 196.8 (C), 168.6 (C), 135.9 (C), 135.2 (C), 134.7 (C), 134.2 (CH), 132.1 (CH), 132.0 (C), 131.1 (CH), 129.0 (C), 128.8 (CH), 128.5 (CH), 107.6 (C), 51.3 (C), 48.0 (CH_2), 41.5 (CH_2), 28.9 (CH_3), 27.8 (CH_3), 20.9 (CH_3), 18.9 (CH_3). HRMS (ESI-): calcd m/z for $C_{19}H_{17}O_2$ [$(M-H-CO_2-C_3H_6O)^-$]: 277.1234; Found: 277.1239.

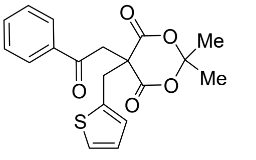

2,2-dimethyl-5-(2-oxo-2-phenylethyl)-5-(thiophen-2-ylmethyl)-1,3-dioxane-4,6-dione (**2k**) was synthesized following the general procedure using C5-monosubstituted Meldrum's acid **1k** (1.18 g, 4.9 mmol). The trituration process gave the compound **2k** (860.5 mg, 49% yield) as a white powder. ^1H NMR ($CDCl_3$, 300 MHz) δ_H 7.95-7.92 (2H, m), 7.63-7.57 (1H, m), 7.49-7.44 (2H, m), 7.23 (1H, dd, J = 5.0, 1.2 Hz), 7.00-6.95 (2H, m), 4.03 (2H, s), 3.55 (2H, s), 2.02 (3H, s), 0.98 (3H, s). $^{13}C\{^1H\}$ NMR ($CDCl_3$, 75 MHz) δ_C 196.4 (C), 168.2 (C), 135.0 (C), 134.6 (C), 134.3 (CH), 128.9 (CH), 128.8 (CH), 128.5 (CH), 127.7 (CH), 126.1 (CH), 107.9 (C), 52.2 (C), 47.9 (CH_2), 39.1 (CH_2), 28.7 (CH_3), 28.5 (CH_3). HRMS (ESI-): calcd m/z for $C_{15}H_{11}O_2S$ [$(M-H-CO_2-C_3H_6O)^-$]: 255.0485; Found: 255.0487.

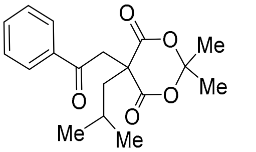

5-isobutyl-2,2-dimethyl-5-(2-oxo-2-phenylethyl)-1,3-dioxane-4,6-dione (**2l**) was synthesized following the general procedure using C5-monosubstituted Meldrum's acid **1l** (981.2 mg, 4.9 mmol). The trituration process gave the compound **2l** (780 mg, 50% yield) as a white powder. ^1H NMR ($CDCl_3$, 300 MHz) δ_H 7.94-7.91 (2H, m), 7.61-7.56 (1H, m), 7.48-7.43 (2H, m), 3.90 (2H, s), 2.12 (3H, s), 1.95 (2H, d, 5.2 Hz), 1.92-1.87 (1H, m), 1.85 (3H, s), 0.99 (6H, d, J = 6.4 Hz). $^{13}C\{^1H\}$ NMR ($CDCl_3$, 75 MHz) δ_C 196.9 (C), 168.8 (C), 135.3 (C), 134.1 (CH), 128.82 (CH), 128.4 (CH), 107.3 (C), 49.5 (C), 48.1 (CH_2), 47.0 (CH_2), 29.5 (CH_3), 29.0 (CH_3), 24.9 (CH), 24.2 (CH_3). HRMS (ESI-): calcd m/z for $C_{14}H_{15}O_2$ [$(M-H-CO_2-C_3H_6O)^-$]: 215.1078; Found: 215.1079.

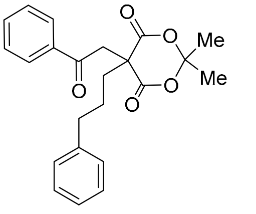

2,2-dimethyl-5-(2-oxo-2-phenylethyl)-5-(3-phenylpropyl)-1,3-dioxane-4,6-dione (**2m**) was synthesized following the general procedure using C5-monosubstituted Meldrum's acid **1m** (1.29 g, 4.9 mmol). The trituration process gave the compound **2m** (727 mg, 39% yield) as a white powder. ^1H

NMR ($CDCl_3$, 300 MHz) δ_H 7.88-7.85 (2H, m), 7.55-7.50 (1H, m), 7.42-7.37 (2H, m), 7.24-7.08 (5H, m), 3.81 (2H, s), 2.59 (2H, t, J = 7.3 Hz), 2.04 (3H, s), 1.99-1.93 (2H, m), 1.79-1.71 (2H, m), 1.69 (3H, s). $^{13}C\{^1H\}$ NMR ($CDCl_3$, 75 MHz) δ_C 196.9 (C), 168.6 (C), 140.6 (C), 135.2 (C), 134.1 (CH), 128.8 (CH), 128.7 (CH), 128.4 (CH), 126.4 (CH), 107.3 (C), 49.9 (C), 45.8 (CH_2), 38.4 (CH_2), 35.4 (CH_2), 29.5 (CH_3), 28.9 (CH_3), 26.53 (CH_2). HRMS (ESI-): calcd m/z for $C_{19}H_{17}O_2$ [$(M-H-CO_2-C_3H_6O)^-$]: 277.1234; Found: 277.1238.

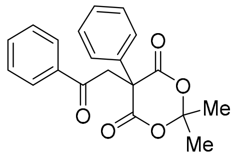

2,2-dimethyl-5-(2-oxo-2-phenylethyl)-5-phenyl-1,3-dioxane-4,6-dione (**2n**) was synthesized following the general procedure using C5-phenyl Meldrum's acid **1n** (1.08 g, 4.9 mmol). The trituration process gave the compound **2n** (281.8 mg, 17% yield) as a white powder. ^1H NMR ($CDCl_3$, 300 MHz) δ_H 7.95-7.91 (2H, m), 7.58-7.41 (8H, m), 4.13 (2H, s), 1.92 (3H, s), 1.34 (3H, s). $^{13}C\{^1H\}$ NMR ($CDCl_3$, 75 MHz) δ_C 196.2 (C), 166.5 (C), 135.0 (C), 133.9 (CH), 133.4 (C), 129.8 (CH), 129.1 (CH), 128.6 (CH), 128.3 (CH), 126.6 (CH), 106.1 (C), 56.1 (C), 51.3 (CH_2), 29.5 (CH_3), 28.3 (CH_3). The analytical data match the published ones.[3]

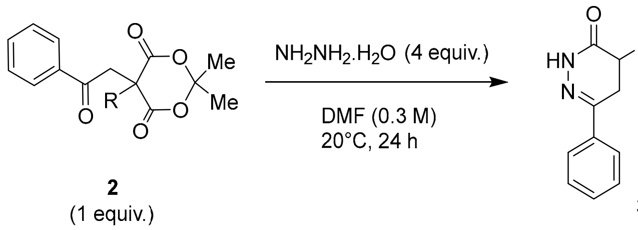

Typical procedure for *N*-unsubstituted C6-Ph 4,5-dihydropyridazinone derivatives 3 preparation. Under N_2, the C5-disubstituted Meldrum's acid derivative **2** (3.3 mmol) was solubilized in DMF (11 mL, 0.3 M) in a round-bottom flask. At 0°C, under vigorous stirring, hydrazine mono-hydrated (0.65 mL, 13.3 mmol) was added dropwise. The reaction was stirred for 24 hours at room temperature and then, the solvent was evaporated under reduce pressure. The crude was dissolved in DCM and washed with an aqueous acidic solution (pH 3-4) and then with a brine solution. The combined organic phases were dried over anhydrous Na_2SO_4 and finally, the solvent was evaporated under reduce pressure. Excepted the compounds **3c** and **3l**, all the products **3a**-**o** were obtained without further purification.

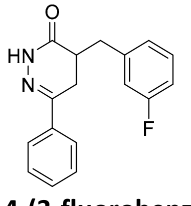

4-(3-fluorobenzyl)-6-phenyl-4,5-dihydropyridazin-3(*2H*)-one (**3a**) was synthesized following the general procedure using C5-disubstituted Meldrum's acid **2a** (1.23 g, 3.3 mmol). Compound **3a** (922.3 mg, 99% yield) was obtained as a white powder. ^1H NMR ($CDCl_3$, 300 MHz) δ_H 8.59 (1H, s), 7.64-7.61 (2H, m), 7.40-7.38 (3H, m), 7.29-7.23 (1H, m), 6.98-6.89 (3H, m), 3.35 (1H, dd, J = 13.1, 3.1 Hz), 2.93-2.59 (4H, m). $^{13}C\{^1H\}$ NMR ($CDCl_3$, 75 MHz) δ_C 169.1 (C), 163.1 (C, d, J = 183.7 Hz), 150.8 (C), 140.6 (C, d, J = 6.0 Hz), 135.6 (C), 130.3 (CH, d, J = 6.0 Hz), 130.1 (CH), 128.8 (CH), 125.9 (CH), 125.0 (CH), 116.3 (CH, d, J = 15.7 Hz), 113.9 (CH, d, J = 15.7 Hz), 37.7 (CH), 35.2 (CH_2), 26.8 (CH_2). $^{19}F\{^1H\}$ NMR ($CDCl_3$, 282 MHz) δ_F -112.8. HRMS (ESI+): calcd m/z for $C_{17}H_{16}FN_2O$ [$(M+H)^+$]: 283.1241; Found: 283.1235.

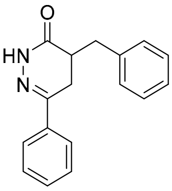

4-benzyl-6-phenyl-4,5-dihydropyridazin-3(*2H*)-one (**3b**) was synthesized following the general procedure using C5-disubstituted Meldrum's acid **2b** (1.17 g, 3.3 mmol). Compound **3b** (863.5 mg, 99% yield) was obtained as a white powder. ^{1}H NMR ($CDCl_3$, 300 MHz) δ_H 8.96 (1H, s), 7.67-7.60 (2H, m), 7.39-7.17 (8H, m), 3.36 (1H, dd, *J* = 13.4, 3.3 Hz), 2.90-2.59 (4H, m). $^{13}C\{^1H\}$ NMR ($CDCl_3$, 75 MHz) δ_C 169.7 (C), 150.8 (C), 138.0 (C), 135.7 (C), 130.0 (CH), 129.3 (CH), 128.8 (CH), 128.7 (CH), 126.9 (CH), 126.0 (CH), 37.9 (CH), 35.4 (CH_2), 26.6 (CH_2). HRMS (ESI+): calcd m/z for $C_{17}H_{17}N_2O$ [$(M+H)^+$]: 265.1335; Found: 265.1334.

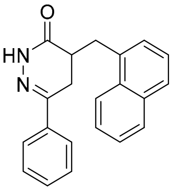

4-(naphthalen-1-ylmethyl)-6-phenyl-4,5-dihydropyridazin-3(*2H*)-one (**3c**) was synthesized following the general procedure using C5-disubstituted Meldrum's acid **2c** (1.33 g, 3.3 mmol). The crude mixture was purified by silica gel column chromatography using AcOEt/heptane (1:1) as eluent giving the compound **2c** (778.1 mg, 75% yield) as a white powder. ^{1}H NMR ($CDCl_3$, 300 MHz) δ_H 9.20 (1H, s), 8.15-8.10 (1H, m), 7.91-7.86 (1H, m), 7.81-7.78 (1H, m), 7.63-7.60 (2H, m), 7.53-7.50 (2H, m), 7.40-7.36 (4H, m), 7.26-7.24 (1H, m), 4.05 (1H, dd, *J* = 13.2, 2.8 Hz), 3.08-2.89 (2H, m), 2.83- 2.66 (2H, m). $^{13}C\{^1H\}$ NMR ($CDCl_3$, 75 MHz) δ_C 169.9 (C), 150.6 (C), 135.6 (C), 134.2 (C), 134.0 (C), 131.7 (C), 129.9 (CH), 129.1 (CH), 128.7 (CH), 127.9 (CH), 127.8 (CH), 126.5 (CH), 126.0 (CH), 125.9 (CH), 125.4 (CH), 123.7 (CH), 37.0 (CH), 33.0 (CH_2), 27.0 (CH_2). HRMS (ESI+): calcd m/z for $C_{21}H_{19}N_2O$ [$(M+H)^+$]: 315.1492; Found: 315.1487.

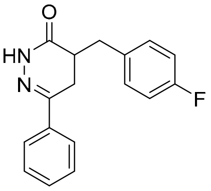

4-(4-fluorobenzyl)-6-phenyl-4,5-dihydropyridazin-3(*2H*)-one (**3d**) was synthesized following the general procedure using C5-disubstituted Meldrum's acid **2d** (1.23 g, 3.3 mmol). Compound **3d** (913 mg, 98% yield) was obtained as a white powder. ^{1}H NMR ($CDCl_3$, 300 MHz) δ_H 8.93 (1H, s), 7.64-7.62 (2H, m), 7.40-7.38 (3H, m), 7.16-7.12 (2H, m), 7.01-6.96 (2H, m), 3.28 (1H, dd, *J* = 12.6, 2.5 Hz), 2.89-2.61 (m, 4H, m). $^{13}C\{^1H\}$ NMR ($CDCl_3$, 75 MHz) δ_C 169.5 (C), 161.9 (C, d, *J* = 182.2 Hz), 150.7 (C), 135.6 (C), 133.6 (C), 130.8 (CH), 130.7 (CH), 130.1 (CH), 128.8 (CH), 125.9 (CH), 115.8 (CH), 115.6 (CH), 37.9 (CH), 34.6 (CH_2), 26.7 (CH_2). $^{19}F\{^1H\}$ NMR ($CDCl_3$, 282 MHz) δ_F -116.0. HRMS (ESI+): calcd m/z for $C_{17}H_{16}FN_2O$ [$(M+H)^+$]: 283.1241; Found: 283.1235.

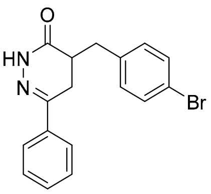

4-(4-bromobenzyl)-6-phenyl-4,5-dihydropyridazin-3(2H)-one (**3e**) was synthesized following the general procedure using C5-disubstituted Meldrum's acid **2e** (1.43 g, 3.3 mmol). Compound **3e** (1.06 mg, 94% yield) was obtained as a white powder. ^{1}H NMR ($CDCl_3$, 300 MHz) δ_H 9.02 (1H, s), 7.66-7.60 (2H, m), 7.43-7.38 (5H, m), 7.08-7.04 (2H, m), 3.26 (1H, dd, *J* = 12.8, 3.0 Hz), 2.91-2.58 (4H, m). $^{13}C\{^1H\}$ NMR ($CDCl_3$, 75 MHz) δ_C 169.3 (C), 150.7 (C), 137.0 (C), 135.5 (C), 131.9 (CH), 131.0 (CH), 130.1 (CH),

128.8 (CH), 125.9 (CH), 120.9 (C), 37.6 (CH), 34.8 (CH_2), 26.7 (CH_2). HRMS (ESI+): calcd m/z for $C_{17}H_{16}BrN_2O$ [$(M+H)^+$]: 343.0441; Found: 343.0439.

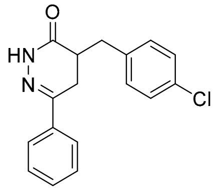

4-(4-chlorobenzyl)-6-phenyl-4,5-dihydropyridazin-3(2H)-one (**3f**) was synthesized following the general procedure using C5-disubstituted Meldrum's acid **2f** (1.28 g, 3.3 mmol). Compound **3f** (976.1 mg, 99% yield) was obtained as a white powder. ^{1}H NMR ($CDCl_3$, 300 MHz) δ_H 9.11 (1H, s), 7.65-7.59 (2H, m), 7.40-7.36 (3H, m), 7.27-7.24 (2H, m), 7.11-7.08 (2H, m), 3.27 (1H, dd, J = 12.5, 2.6 Hz), 2.89-2.56 (4H, m). ^{13}C{^{1}H} NMR ($CDCl_3$, 75 MHz) δ_C 169.4 (C), 150.7 (C), 136.5 (C), 135.6 (C), 132.8 (C), 130.6 (CH), 130.0 (CH), 128.9 (CH), 128.7 (CH), 125.9 (CH), 37.7 (CH), 34.8 (CH_2), 26.7 (CH_2). The analytical data match the published ones.[3]

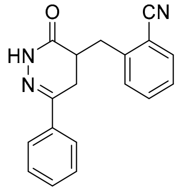

2-((3-oxo-6-phenyl-2,3,4,5-tetrahydropyridazin-4-yl)methyl)benzonitrile (**3g**) was synthesized following the general procedure using C5-disubstituted Meldrum's acid **2g** (1.25 g, 3.3 mmol). Compound **3g** (878.4 mg, 92% yield) was obtained as a white powder. ^{1}H NMR ($CDCl_3$, 300 MHz) δ_H 8.99 (1H, s), 7.67-7.63 (3H, m), 7.55-7.52 (1H, m), 7.47-7.44 (1H, m), 7.39-7.35 (4H, m), 3.54 (1H, dd, J = 14.1, 5.6 Hz), 3.18-3.02 (2H, m), 2.98-2.87 (1H, m), 2.71 (1H, dd, J = 16.0, 11.9 Hz). ^{13}C{^{1}H} NMR ($CDCl_3$, 75 MHz) δ_C 168.6 (C), 150.9 (C), 142.5 (C), 135.4 (C), 133.2 (CH), 133.1 (CH), 130.7 (CH), 130.1 (CH), 128.7 (CH), 127.6 (CH), 125.9 (CH), 118.2 (C), 113.2 (C), 37.4 (CH), 33.8 (CH_2), 27.5 (CH_2). HRMS (ESI+): calcd m/z for $C_{18}H_{16}N_3O$ [$(M+H)^+$]: 290.1288; Found: 290.1284.

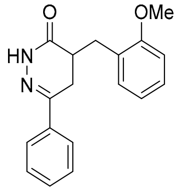

4-(2-methoxybenzyl)-6-phenyl-4,5-dihydropyridazin-3(*2H*)-one (**3h**) was synthesized following the general procedure using C5-disubstituted Meldrum's acid **2h** (1.26 g, 3.3 mmol). Compound **3h** (961.6 mg, 99% yield) was obtained as a white powder. ^{1}H NMR ($CDCl_3$, 300 MHz) δ_H 8.78 (1H, s), 7.65-7.60 (2H, m), 7.40-7.35 (3H, m), 7.25-7.20 (1H, m), 7.12-7.09 (1H, m), 6.91-6.80 (2H, m), 3.61 (3H, s), 3.33 (1H, dd, J = 13.3, 4.3 Hz), 2.98-2.88 (1H, m), 2.85-2.65 (3H, m). ^{13}C{^{1}H} NMR ($CDCl_3$, 75 MHz) δ_C 170.2 (C), 157.75 (C), 150.8 (C), 136.1 (C), 131.2 (CH), 129.7 (CH), 128.6 (CH), 128.3 (CH), 126.2 (C), 125.9 (CH), 120.6 (CH), 110.4 (CH), 55.0 (CH_3), 36.6 (CH), 30.3 (CH_2), 26.7 (CH_2). HRMS (ESI+): calcd m/z for $C_{18}H_{19}N_2O_2$ [$(M+H)^+$]: 295.1441; Found: 295.1444.

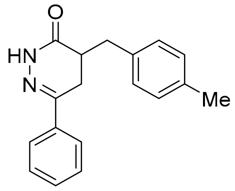

4-(4-methylbenzyl)-6-phenyl-4,5-dihydropyridazin-3(*2H*)-one (**3i**) was synthesized following the general procedure using C5-disubstituted Meldrum's acid **2i** (1.21 g, 3.3 mmol). Compound **3i** (780.8 mg, 85% yield) was obtained as a white powder. ^{1}H NMR ($CDCl_3$, 300 MHz) δ_H 9.12 (1H, s), 7.68-7.62 (2H, m), 7.41-7.37 (3H, m), 7.13-7.05 (4H, m), 3.31 (1H, dd, J = 12.8, 3.0 Hz), 2.89-2.61 (4H, m), 2.34 (3H, s). ^{13}C{^{1}H} NMR ($CDCl_3$, 75 MHz) δ_C 169.9 (C), 150.7 (C), 136.5 (C), 135.7 (C), 134.8 (C), 129.9 (CH),

129.5 (CH), 129.2 (CH), 128.7 (CH), 126.0 (CH), 38.0 (CH), 35.0 (CH_2), 26.6 (CH_2), 21.1 (CH_3). HRMS (ESI+): calcd m/z for $C_{18}H_{19}N_2O$ [$(M+H)^+$]: 279.1492; Found: 279.1498.

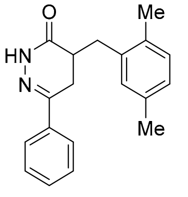

4-(2,5-dimethylbenzyl)-6-phenyl-4,5-dihydropyridazin-3(2H)-one (**3j**) was synthesized following the general procedure using C5-disubstituted Meldrum's acid **2j** (1.26 g, 3.3 mmol). Compound **3j** (935.9 mg, 97% yield) was obtained as a white powder. ^1H NMR ($CDCl_3$, 300 MHz) δ_H 9.02 (1H, s), 7.68-7.64 (2H, m), 7.41-7.39 (3H, m), 7.07 (1H, d, *J* = 7.7 Hz), 6.99-6.96 (1H, m), 6.81 (1H, s), 3.38 (1H, dd, *J* = 13.8, 3.4 Hz), 2.86-2.52 (4H, m), 2.28 (3H, s), 2.22 (3H, s). $^{13}C\{^1H\}$ NMR ($CDCl_3$, 75 MHz) δ_C 170.0 (C), 150.6 (C), 135.9 (C), 135.7 (C), 135.6 (C), 133.3 (C), 131.1 (CH), 130.8 (CH), 130.0 (CH), 128.7 (CH), 127.8 (CH), 126.0 (CH), 36.7 (CH), 32.9 (CH_2), 26.5 (CH_2), 20.9 (CH_3), 19.0 (CH_3). HRMS (ESI+): calcd m/z for $C_{19}H_{21}N_2O$ [$(M+H)^+$]: 293.1648; Found: 293.1656.

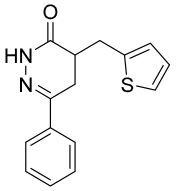

6-phenyl-4-(thiophen-2-ylmethyl)-4,5-dihydropyridazin-3(*2H*)-one (**3k**) was synthesized following the general procedure using C5-disubstituted Meldrum's acid **2k** (1.19 g, 3.3 mmol). Compound **3k** (865.4 mg, 97% yield) was obtained as a white powder. ^1H NMR ($CDCl_3$, 300 MHz) δ_H 9.10 (1H, s), 7.70-7.65 (2H, m), 7.42-7.38 (H, m), 7.19 (1H, dd, *J* = 5.1, 1.1 Hz), 6.95 (1H, dd, *J* = 5.1, 3.4 Hz); 6.85 (1H, d, *J* = 3.3 Hz); 3.49 (1H, dd, *J* = 14.9, 3.9 Hz), 3.10 (1H, dd, *J* = 14.9, 8.9 Hz), 3.00 (1H, dd, *J* = 15.8, 5.9 Hz); 2.87-2.77 (1H, m), 2.70 (1H, dd, *J* = 15.8, 10.8 Hz). $^{13}C\{^1H\}$ NMR ($CDCl_3$, 75 MHz) δ_C 169.1 (C), 150.9 (C), 140.0 (C), 135.7 (C), 130.0 (CH), 128.7 (CH), 127.1 (CH), 126.7 (CH), 126.0 (CH), 124.6 (CH), 38.0 (CH), 29.5 (CH_2), 26.8 (CH_2). HRMS (ESI+): calcd m/z for $C_{15}H_{15}N_2OS$ [$(M+H)^+$]: 271.0900; Found: 271.0902.

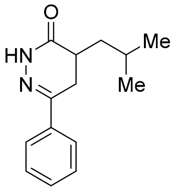

4-isobutyl-6-phenyl-4,5-dihydropyridazin-3(2H)-one (**3l**) was synthesized following the general procedure using C5-disubstituted Meldrum's acid **2l** (1.05 g, 3.3 mmol). The crude mixture was purified by silica gel column chromatography using AcOEt/heptane (1:1) as eluent giving the compound **3l** (752.4 mg, 99% yield) as a white powder. ^1H NMR ($CDCl_3$, 300 MHz) δ_H 8.91 (1H, s), 7.76-7.70 (2H, m), 7.44-7.40 (3H, m), 3.04 (1H, dd, *J* = 16.5, 6.5 Hz), 2.72 (1H, dd, *J* = 16.6, 9.2 Hz), 2.63-2.55 (1H, m), 1.85-1.72 (2H, m), 1.36-1.28 (1H, m), 1.01-0.88 (6H, m). $^{13}C\{^1H\}$ NMR ($CDCl_3$, 75 MHz) δ_C 170.7 (C), 150.7 (C), 136.0 (C), 129.9 (CH), 128.8 (CH), 125.9 (CH), 38.6 (CH_2), 34.0 (CH), 28.0 (CH_2), 25.3 (CH), 23.2 (CH_3), 21.8 (CH_3). HRMS (ESI+): calcd m/z for $C_{14}H_{19}N_2O$ [$(M+H)^+$]: 231.1492; Found: 231.1498.

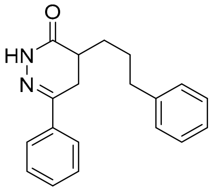

6-phenyl-4-(3-phenylpropyl)-4,5-dihydropyridazin-3(*2H*)-one (**3m**) was synthesized following the general procedure using C5-disubstituted Meldrum's acid **2m** (1.26 g, 3.3 mmol). Compound **3m** (945.5 mg, 98% yield) was obtained as a white powder. ^1H NMR ($CDCl_3$, 300 MHz) δ_H 8.91 (1H, s), 7.75-7.69

(2H, m), 7.47-7.40 (3H, m), 7.31-7.26 (2H, m), 7.21-7.17 (3H, m), 3.05 (1H, dd, *J* = 16.7, 6.7 Hz), 2.78-2.49 (4H, m), 2.02-1.68 (3H, m), 1.62-1.50 (1H, m). $^{13}C\{^1H\}$ NMR ($CDCl_3$, 75 MHz) δ_C 170.2 (C), 150.8 (C), 141.9 (C), 135.9 (C), 129.9 (CH), 128.8 (CH), 128.5 (CH), 126.0 (CH), 125.9 (CH), 35.9 (CH), 35.8 (CH_2), 29.3 (CH_2), 28.6 (CH_2), 27.9 (CH_2). HRMS (ESI+): calcd m/z for $C_{19}H_{21}N_2O$ [$(M+H)^+$]: 293.1648; Found: 293.1646.

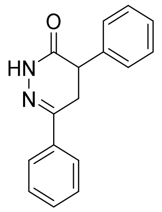

4,6-diphenyl-4,5-dihydropyridazin-3(*2H*)-one (**3n**) was synthesized following the general procedure using C5-disubstituted Meldrum's acid **2n** (1.12 g, 3.3 mmol). Compound **3n** (793 mg, 96% yield) as a white powder. ^1H NMR ($CDCl_3$, 300 MHz) δ_H 8.78 (1H, s), 7.75-7.69 (2H, m), 7.44-7.26 (8H, m), 3.85 (1H, dd, *J* = 9.1, 7.2 Hz), 3.33 (1H, dd, *J* = 16.9, 7.2 Hz), 3.22 (1H, dd, *J* = 16.9, 9.3 Hz). $^{13}C\{^1H\}$ NMR ($CDCl_3$, 75 MHz) δ_C 167.9 (C), 150.8 (C), 137.0 (C), 135.4 (C), 129.9 (CH), 129.0 (CH), 128.7 (CH), 127.7 (3 CH), 125.8 (2 CH), 42.8 (CH), 30.9 (CH_2). The analytical data match the published ones.[3]

4-(3-fluorobenzyl)-4,5-dihydropyridazin-3(*2H*)-one (**6**)

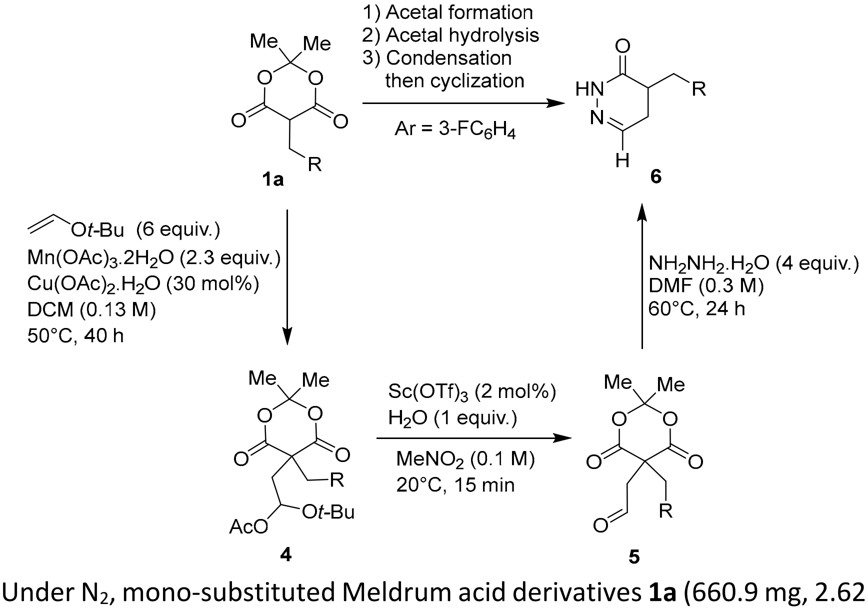

Under N_2, mono-substituted Meldrum acid derivatives **1a** (660.9 mg, 2.62 mmol) was dissolved in DCM (21 mL, 0.13 M) in a light shielded round-bottom flask and *tert*-butyl vinyl ether (2.1 mL, 15.72 mmol) was added at room temperature. Under stirring, manganese acetate dihydrate (1.62 g, 6.03 mmol) was added followed with copper acetate monohydrate (158 mg, 0.79 mmol). The mixture was heated at reflux (50°C, oil bath temperature) for 40 hours. Then, the crude was filtered on celite using DCM as eluent. The filtrate was washed with water 2 times then with a brine solution. The combined organic phases were dried over anhydrous Na_2SO_4 and finally, the solvent was evaporated under reduce pressure. The crude acetal **4** was dried under vacuum.

Under N_2, scandium triflate (20 mol%) and the crude acetal **4** were dissolved in nitromethane (2.6 mL, 1 M), in a round-bottom flask then under stirring water (0.05 mL, 2.62 mmol) was added. The mixture was stirred at room temperature for 15 minutes. Then, the solvent was evaporated under reduce pressure and the crude was dried under vacuum. The crude was dissolved in DCM and filtered on silica gel using DCM/AcOEt (9:1) as eluent. Then, the solvent was evaporated under reduce pressure and the crude aldehyde **5** was dried under vacuum.

Under N_2, the crude aldehyde **5** was dissolved in DMF (8.7 mL, 0.3 M) and hydrazine monohydrate **2c** (0.51 mL, 10.5 mmol) was added under stirring at 0°C. The solution was heated at 60°C (oil bath

temperature) for 24 hours. Then, the solvent was evaporated under reduce pressure. The crude was dissolved in DCM and washed with an aqueous acidic solution (pH 3-4) followed with a brine solution. The combined organic phases were dried over anhydrous Na_2SO_4 and finally, the solvent was evaporated under reduce pressure. The crude mixture was purified by silica gel column chromatography using AcOEt/heptane (1:1) as eluent giving the compound **6** (378.2 mg, 70% yield over three steps) as a white powder. ^{1}H NMR ($CDCl_3$, 300 MHz) δ_H 8.84 (1H, s), 7.30-7.22 (1H, m), 7.11 (1H, t, *J* = 3.1 Hz), 6.96-6.87 (3H, m), 3.34-3.25 (1H, m), 2.72-2.61 (2H, m), 2.46- 2.37 (1H, m), 2.27-2.11 (1H, m). ^{13}C{^{1}H} NMR ($CDCl_3$, 75 MHz) δ_C 169.4 (C), 163.0 (C, d, *J* = 244.5 Hz), 144.5 (CH), 140.6 (C, d, *J* = 7.5 Hz), 130.3 (CH, d, *J* = 8.2 Hz), 124.9 (CH, d, *J* = 3.0 Hz), 116.1 (CH, d, *J* = 21.0 Hz), 113.9 (CH, d, *J* = 20.2 Hz), 37.2 (CH, d, *J* = 1.5 Hz), 35.1 (CH_2), 26.7 (CH_2). ^{19}F{^{1}H} NMR ($CDCl_3$, 282 MHz) δ_F -112.9. HRMS (ESI+): calcd m/z for $C_{11}H_{12}FN_2O$ [$(M+H)^+$]: 207.0928; Found: 207.0931.

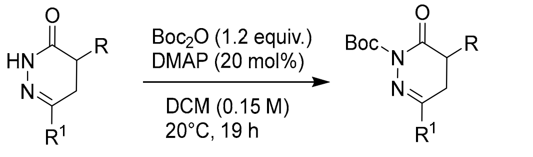

3 (R^1 = Ph)
6 (R = 3-FC_6H_4 R^1 = H)
(1 equiv.)

7 (R^1 = Ph)
8 (R = 3-FC_6H_4 R^1 = H)

Typical procedure for *N*-Boc 4,5-dihydropyridazinone derivatives 7 and 8 preparation. Under N_2, Boc_2O (938.5 mg, 4.3 mmol) and DMAP (87.5 mg, 716 µmol) were introduced in a round-bottom flask. The *N*-unsubstituted 4,5-dihydropyridazinone **3** or **6** (3.58 mmol) was dissolved in anhydrous DCM (24 mL, 0.15 M) and introduced slowly in the round-bottom flask at room temperature. The solution was kept under vigorous stirring overnight. Then, the solvent was evaporated under reduce pressure and the crude mixture was purified by silica gel column chromatography to give products **7** or **8**.

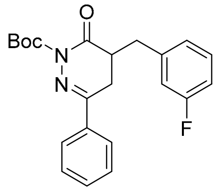

***tert*-butyl 5-(3-fluorobenzyl)-6-oxo-3-phenyl-5,6-dihydropyridazine-1(*4H*)-carboxylate** (**7a**) was synthesized following the general procedure using *N*-unsubstituted 4,5-dihydropyridazinone **3b** (1.01 g, 3.58 mmol). The crude mixture was purified by silica gel column chromatography using DCM/heptane (8:2) as eluent giving the compound **7a** (1.2 g, 88% yield) as a yellowish powder. mp = 139-141°C. ^{1}H NMR ($CDCl_3$, 300 MHz) δ_H 7.75-7.67 (2H, m), 7.42-7.37 (3H, m), 7.28-7.23 (1H, m), 6.98-6.86 (3H, m), 3.35 ppm (dd, 1H, dd, *J* = 13.7, 4.0 Hz), 2.95-2.59 (4H, m), 1.62 (9H, s). ^{13}C{^{1}H} NMR ($CDCl_3$, 75 MHz) δ_C 168.0 (C), 163.0 (C, d, *J* = 245.2 Hz), 152.3 (C), 150.4 (C), 140.3 (C, d, *J* = 7.5 Hz), 135.2 (C), 130.6 (CH), 130.4 (CH, d, *J* = 8.2 Hz), 128.8 (CH), 126.5 (CH), 125.0 (CH, d, *J* = 3.0 Hz), 116.2 (CH, d, *J* = 21.0 Hz), 114.0 (CH, d, *J* = 21.0 Hz), 84.5 (C), 40.1 (CH), 35.2 (CH_2), 28.1 (CH_3), 27.1 (CH_2). ^{19}F{^{1}H} NMR ($CDCl_3$, 282 MHz) δ_F -112.7. HRMS (ESI+): calcd m/z for $C_{17}H_{16}FN_2O$ [$(M+H-C_5H_9O_2)^+$]: 283.1241; Found: 283.1240.

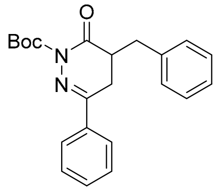

***tert*-butyl 5-benzyl-6-oxo-3-phenyl-5,6-dihydropyridazine-1(*4H*)-carboxylate** (**7b**) was synthesized following the general procedure using *N*-unsubstituted 4,5-dihydropyridazinone **3b** (946.3 mg, 3.58 mmol). The crude mixture was purified by silica gel column chromatography using DCM/heptane (8:2) as eluent giving the compound **7b** (861 mg, 66% yield) as a white powder. ^{1}H NMR ($CDCl_3$, 300 MHz) δ_H 7.75-7.67 (2H, m), 7.44-7.23 (6H, m), 7.17-7.15 (2H, m), 3.37 (1H, dd, J = 13.8, 4.0 Hz), 2.93-2.83 (2H,

m), 2.76-2.62 (2H, m), 1.64 (9H, s). $^{13}C\{^1H\}$ NMR ($CDCl_3$, 75 MHz) δ_C 168.4 (C), 152.4 (C), 150.5 (C), 137.8 (C), 135.3 (C), 130.5 (CH), 129.3 (CH), 128.9 (CH), 128.7 (CH), 127.0 (CH), 126.5 (CH), 84.4 (C), 40.4 (CH), 35.4 (CH_2), 28.1 (CH_3), 26.9 (CH_2). The analytical data match the published ones.[4]

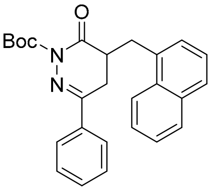

***tert*-butyl 5-(naphthalen-1-ylmethyl)-6-oxo-3-phenyl-5,6-dihydropyridazine-1(*4H*)-carboxylate** (**7c**) was synthesized following the general procedure using *N*-unsubstituted 4,5-dihydropyridazinone **3c** (1.13 g, 3.58 mmol). The crude mixture was purified by silica gel column chromatography using DCM/heptane (8:2) as eluent giving the compound **7c** (1.1 mg, 88% yield) as a yellowish foam. ^1H NMR ($CDCl_3$, 300 MHz) δ_H 8.07-8.04 (1H, m), 7.89-7.84 (1H, m), 7.78 (1H, d, *J* = 8.2 Hz), 7.70-7.66 (2H, m), 7.54-7.46 (2H, m), 7.43-7.33 (4H, m), 7.21 (1H, d, *J* = 6.4 Hz), 4.03 (1H, dd, J = 13.8, 3.4 Hz), 3.14-3.04 (1H, m), 2.94 (1H, dd, *J* = 13.8, 11.0 Hz), 2.81 (1H, dd, *J* = 16.9, 6.3 Hz), 2.72 (1H, dd, *J* = 16.9, 8.8 Hz), 1.66 (9H, s). $^{13}C\{^1H\}$ NMR ($CDCl_3$, 75 MHz) δ_C 168.5 (C), 152.4 (C), 150.5 (C), 135.3 (C), 134.2 (C), 133.7 (C), 131.7 (C), 130.5 (CH), 129.1 (CH), 128.7 (CH), 128.0 (CH), 127.9 (CH), 126.6 (CH), 126.5 (CH), 126.0 (CH), 125.4 (CH), 123.6 (CH), 84.5 (C), 39.4 (CH), 33.0 (CH_2), 28.1 (CH_3), 27.2 (CH_2). HRMS (ESI+): calcd m/z for $C_{21}H_{19}N_2O$ [$(M+H-C_5H_9O_2)^+$]: 315.1492; Found: 315.1500.

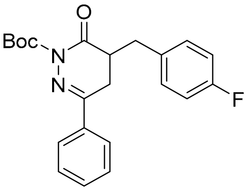

***tert*-butyl 5-(4-fluorobenzyl)-6-oxo-3-phenyl-5,6-dihydropyridazine-1(*4H*)-carboxylate** (**7d**) was synthesized following the general procedure using *N*-unsubstituted 4,5-dihydropyridazinone **3d** (1.01 g, 3.58 mmol). The crude mixture was purified by silica gel column chromatography using DCM/heptane (8:2) as eluent giving the compound **7d** (1.22 g, 89% yield) as a yellowish powder. mp = 111-113°C. ^1H NMR ($CDCl_3$, 300 MHz) δ_H 7.75-7.66 (2H, m), 7.43-7.36 (3H, m), 7.14-7.10 (2H, m), 7.02-6.94 (2H, m), 3.30 (1H, dd, *J* = 13.5, 3.9 Hz), 2.91-2.64 (4H, m), 1.62 (9H, s). $^{13}C\{^1H\}$ NMR ($CDCl_3$, 75 MHz) δ_C 168.1 (C), 161.9 (C, d, *J* = 243.7 Hz), 152.2 (C), 150.4 (C), 135.2 (C), 133.41 (C, d, *J* = 3.0 Hz), 130.8 (CH), 130.7 (CH), 130.5 (CH), 128.8 (CH), 126.4 (CH), 115.8 (CH), 115.6 (CH), 84.4 (C), 40.34 (CH, d, *J* = 1.5 Hz), 34.6 (CH_2), 28.1 (CH_3), 27.0 (CH_2). $^{19}F\{^1H\}$ NMR ($CDCl_3$, 282 MHz) δ_F -115.8. HRMS (ESI+): calcd m/z for $C_{17}H_{16}FN_2O$ [$(M+H-C_5H_9O_2)^+$]: 283.1241; Found: 283.1240.

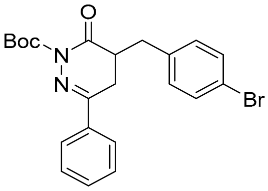

***tert*-butyl 5-(4-bromobenzyl)-6-oxo-3-phenyl-5,6-dihydropyridazine-1(*4H*)-carboxylate** (**7e**) was synthesized following the general procedure using *N*-unsubstituted 4,5-dihydropyridazinone **3e** (1.3 g, 3.58 mmol). The crude mixture was purified by silica gel column chromatography using DCM/heptane (8:2) as eluent giving the compound **7e** (952.3 mg, 60% yield) as a yellowish foam. ^1H NMR ($CDCl_3$, 300 MHz) δ_H 7.75-7.66 (2H, m), 7.44-7.35 (5H, m), 7.05-7.03 (2H, m), 3.28 (1H, dd, *J* = 13.6, 4.0 Hz), 2.93-2.80 (2H, m), 2.75-2.58 (2H, m), 1.62 (9H, s). $^{13}C\{^1H\}$ NMR ($CDCl_3$, 75 MHz) δ_C 168.0 (C), 152.2 (C), 150.4 (C), 136.8 (C), 135.2 (C), 132.0 (CH), 131.0 (CH), 130.6 (CH), 128.8 (CH), 126.4 (CH), 121.0 (C), 84.5 (C), 40.1 (CH), 34.9 (CH_2), 28.1 (CH_3), 27.0 (CH_2). HRMS (ESI+): calcd m/z for $C_{17}H_{16}BrN_2O$ [$(M+H-C_5H_9O_2)^+$]: 343.0441; Found: 343.0448.

[4] Zhou, Y.; Zhou, H.; Xu, J. *J. Org. Chem.* **2022**, *87*, 3677.

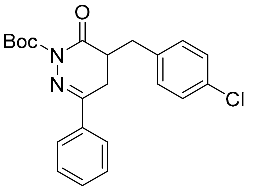

***tert*-butyl 5-(4-chlorobenzyl)-6-oxo-3-phenyl-5,6-dihydropyridazine-1(*4H*)-carboxylate** (**7f**) was synthesized following the general procedure using *N*-unsubstituted 4,5-dihydropyridazinone **3f** (1.07 g, 3.58 mmol). The crude mixture was purified by silica gel column chromatography using DCM/heptane (8:2) as eluent giving the compound **7f** (956.8 mg, 67% yield) as a yellowish foam. ^{1}H NMR ($CDCl_3$, 300 MHz) δ_H 7.75-7.66 (2H, m), 7.45-7.35 (3H, m), 7.29-7.25 (2H, m), 7.11-7.08 (2H, m), 3.30 (1H, dd, *J* = 13.6, 4.0 Hz), 2.93-2.80 (2H, m), 2.76-2.58 (2H, m), 1.62 (9H, s). ^{13}C{^{1}H} NMR ($CDCl_3$, 75 MHz) δ_C 168.0 (C), 152.2 (C), 150.4 (C), 136.2 (C), 135.2 (C), 132.9 (C), 130.65 (CH), 130.57 (CH), 129.0 (CH), 128.8 (CH), 126.4 (CH), 84.4 (C), 40.2 (CH), 34.8 (CH_2), 28.1 (CH_3), 27.0 (CH_2). HRMS (ESI+): calcd m/z for $C_{17}H_{16}ClN_2O$ [$(M+H-C_5H_9O_2)^+$]: 299.0946; Found: 299.0945.

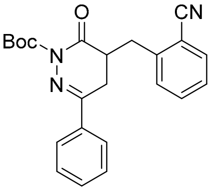

***tert*-butyl 5-(2-cyanobenzyl)-6-oxo-3-phenyl-5,6-dihydropyridazine-1(*4H*)-carboxylate** (**7g**) was synthesized following the general procedure using N-unsubstituted 4,5-dihydropyridazinone **3g** (1.04 g, 3.58 mmol). The crude mixture was purified by silica gel column chromatography using DCM/heptane (8:2) as eluent giving the compound **7g** (850.5 mg, 61% yield) as a yellowish foam. ^{1}H NMR (CDCl3, 300 MHz) δ_H 7.76-7.69 (2H, m), 7.64 (1H, dd, J = 7.7, 1.1 Hz), 7.54 (1H, td, J = 7.6, 1.4 Hz), 7.46-7.33 (5H, m), 3.54 (1H, dd, J = 14.0, 5.8 Hz), 3.15-3.06 (2H, m), 3.03-2.93 (1H, m), 2.75 (1H, dd, J = 15.9, 11.8 Hz), 1.61 (9H, s). ^{13}C{^{1}H} NMR (CDCl3, 75 MHz) δ_C 167.4 (C), 152.2 (C), 150.3 (C), 142.3 (C), 135.1 (C), 133.3 (CH), 133.1 (CH), 130.9 (CH), 130.6 (CH), 128.7 (CH), 127.7 (CH), 126.4 (CH), 118.1 (C), 113.1 (C), 84.5 (C), 39.7 (CH), 34.0 (CH2), 28.0 (CH3, CH2). HRMS (ESI+): calcd m/z for $C_{18}H_{16}N_3O$ [$(M+H-C_5H_9O_2)^+$]: 290.1288; Found: 290.1294.

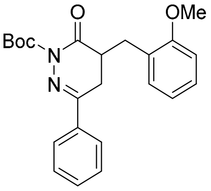

***tert*-butyl 5-(2-methoxybenzyl)-6-oxo-3-phenyl-5,6-dihydropyridazine-1(*4H*)-carboxylate** (**7h**) was synthesized following the general procedure using *N*-unsubstituted 4,5-dihydropyridazinone **3h** (1.05 g, 3.58 mmol). The crude mixture was purified by silica gel column chromatography using DCM/heptane (8:2) as eluent giving the compound **7h** (988.5 mg, 70% yield) as a yellowish foam. ^{1}H NMR ($CDCl_3$, 300 MHz) δ_H 7.74-7.67 (2H, m), 7.40-7.37 (3H, m), 7.26-7.22 (1H, m), 7.08 (1H, dd, *J* = 7.4, 1.7 Hz), 6.87 (1H, td, *J* = 7.4, 1.0 Hz), 6.82 (1H, d, *J* = 8.2 Hz), 3.63 (3H, s), 3.33 (1H, dd, *J* = 13.4, 4.6 Hz), 3.01 (1H, dddd, *J* = 10.7, 8.8, 6.2, 4.6 Hz), 2.87-2.66 (3H, m), 1.63 (9H, s). ^{13}C{^{1}H} NMR ($CDCl_3$, 75 MHz) δ_C 168.8 (C), 157.7 (C), 152.4 (C), 150.7 (C), 135.7 (C), 131.3 (CH), 130.2 (CH), 128.6 (CH), 128.4 (CH), 126.4 (CH), 126.0 (C), 120.6 (CH), 110.4 (CH), 84.2 (C), 55.0 (CH_3), 38.9 (CH), 30.4 (CH_2), 28.1 (CH_3), 27.2 (CH_2). HRMS (ESI+): calcd m/z for $C_{18}H_{19}N_2O_2$ [$(M+H-C_5H_9O_2)^+$]: 295.1441; Found: 295.1455.

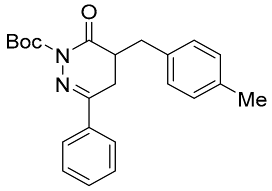

***tert*-butyl 5-(4-methylbenzyl)-6-oxo-3-phenyl-5,6-dihydropyridazine-1(*4H*)-carboxylate** (**7i**) was synthesized following the general procedure using *N*-unsubstituted 4,5-dihydropyridazinone **3i** (996.5 mg, 3.58 mmol). The crude mixture was purified by silica gel column chromatography using DCM/heptane (8:2) as eluent giving the compound **7i** (1.6 g, 86% yield) as a white solid. mp = 72-75°C. ^{1}H NMR ($CDCl_3$, 300 MHz) δ_H 7.77-7.66 (2H, m), 7.44-7.34 (3H, m), 7.12-7.02 (4H, m), 3.31 (1H, dd, J = 13.8, 3.9 Hz), 2.91-2.80 (2H, m), 2.73-2.62 (2H, m), 2.33 (3H, s), 1.64 (9H, s). $^{13}C\{^{1}H\}$ NMR ($CDCl_3$, 75 MHz) δ_C 168.5 (C), 152.4 (C), 150.5 (C), 136.6 (C), 135.3 (C), 134.6 (C), 130.4 (CH), 129.5 (CH), 129.2 (CH), 128.7 (CH), 126.5 (CH), 84.3 (C), 40.5 (CH), 35.0 (CH_2), 28.1 (CH_3), 26.9 (CH_2), 21.2 (CH_3). HRMS (ESI+): calcd m/z for $C_{18}H_{19}N_2O$ [$(M+H-C_5H_9O_2)^+$]: 279.1492; Found: 279.1487.

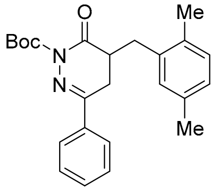

***tert*-butyl 5-(2,5-dimethylbenzyl)-6-oxo-3-phenyl-5,6-dihydropyridazine-1(*4H*)-carboxylate** (**7j**) was synthesized following the general procedure using *N*-unsubstituted 4,5-dihydropyridazinone **3j** (1.05 g, 3.58 mmol). The crude mixture was purified by silica gel column chromatography using DCM/heptane (8:2) as eluent giving the compound **7j** (1.17 g, 83% yield) as a white solid. mp = 112-116°C. ^{1}H NMR ($CDCl_3$, 300 MHz) δ_H 7.79-7.69 (2H, m), 7.46-7.35 (3H, m), 7.06 (1H, d, *J* = 7.7 Hz); 6.97 (1H, dd, *J* = 7.7, 1.2 Hz), 6.78 (1H, s), 3.37 (1H, dd, *J* = 13.9, 4.0 Hz), 2.92-2.68 (3H, m), 2.63-2.55 (1H, m), 2.26 (3H, s), 2.22 (3H, s), 1.65 (9H, s). $^{13}C\{^{1}H\}$ NMR ($CDCl_3$, 75 MHz) δ_C 168.5 (C), 152.3 (C), 150.5 (C), 135.60 (C), 135.58 (C), 135.3 (C), 133.3 (C), 131.1 (CH), 130.8 (CH), 130.5 (CH), 128.7 (CH), 127.8 (CH), 126.5 (CH), 84.4 (C), 39.1 (CH), 32.9 (CH_2), 28.1 (CH_3), 26.8 (CH_2), 20.9 (CH_3), 19.0 (CH_3). HRMS (ESI+): calcd m/z for $C_{19}H_{21}N_2O$ [$(M+H-C_5H_9O_2)^+$]: 293.1648; Found: 293.1650.

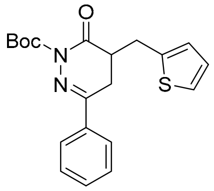

***tert*-butyl 6-oxo-3-phenyl-5-(thiophen-2-ylmethyl)-5,6-dihydropyridazine-1(*4H*)-carboxylate** (**7k**) was synthesized following the general procedure using *N*-unsubstituted 4,5-dihydropyridazinone **3k** (968 mg, 3.58 mmol). The crude mixture was purified by silica gel column chromatography using DCM/heptane (8:2) as eluent giving the compound **7k** (1.26 g, 95% yield) as a white foam. ^{1}H NMR ($CDCl_3$, 300 MHz) δ_H 7.77-7.71 (2H, m), 7.45-7.36 (3H, m), 7.19 (1H, dd, *J* = 5.1, 1.0 Hz), 6.94 (1H, dd, *J* = 5.1, 3.4 Hz), 6.83 (1H, d, *J* = 3.3 Hz), 3.50 (1H, dd, *J* = 14.9, 4.0 Hz), 3.12-3.00 (2H, m), 2.88 (1H, tdd, *J* = 10.0, 5.7, 4.2 Hz), 2.71 (1H, dd, *J* = 16.2, 10.6 Hz), 1.62 (9H, s). $^{13}C\{^{1}H\}$ NMR ($CDCl_3$, 75 MHz) δ_C 167.8 (C), 152.5 (C), 150.4 (C), 139.7 (C), 135.3 (C), 130.5 (CH), 128.7 (CH), 127.2 (CH), 126.8 (CH), 126.5 (CH), 124.7 (CH), 84.4 (C), 40.4 (CH), 29.5 (CH_2), 28.1 (CH_3), 27.1 (CH_2). HRMS (ESI+): calcd m/z for $C_{15}H_{15}N_2OS$ [$(M+H-C_5H_9O_2)^+$]: 271.0900; Found: 271.0907.

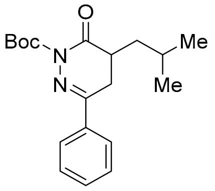

***tert*-butyl 5-isobutyl-6-oxo-3-phenyl-5,6-dihydropyridazine-1(*4H*)-carboxylate** (**7l**) was synthesized following the general procedure using *N*-unsubstituted 4,5-dihydropyridazinone **3l** (824.5 mg, 3.58 mmol). The crude mixture was purified by silica gel column chromatography using DCM/heptane (8:2) as eluent giving the compound **7l** (1.03 g, 87% yield) as a white powder. mp = 100-102°C. ^{1}H NMR ($CDCl_3$, 300 MHz) δ_H 7.82-7.77 (2H, m), 7.45-7.38 (3H, m), 3.05 (1H, dd, *J* = 16.2, 5.6 Hz), 2.80-2.62 (2H, m), 1.84-1.67 (2H, m), 1.60 (9H, s), 1.39-1.25 (2H, m), 0.93 (6H, dd, *J* = 17.3, 6.3 Hz). $^{13}C\{^{1}H\}$ NMR ($CDCl_3$, 75 MHz) δ_C 169.2 (C), 152.4 (C), 150.6 (C), 135.6 (C), 130.4 (CH), 128.8 (2 CH), 126.4 (2 CH), 84.2 (C), 38.6 (CH_2), 36.6 (CH), 28.5 (CH_2), 28.1 (3 CH_3), 25.4 (CH), 23.0 (CH_3), 22.0 (CH_3). HRMS (ESI+): calcd m/z for $C_{14}H_{19}N_2O$ [$(M+H-C_5H_9O_2)^+$]: 231.1492; Found: 231.1499.

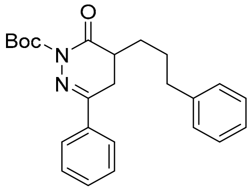

***tert*-butyl 6-oxo-3-phenyl-5-(3-phenylpropyl)-5,6-dihydropyridazine-1(*4H*)-carboxylate** (**7m**) was synthesized following the general procedure using *N*-unsubstituted 4,5-dihydropyridazinone **3m** (1.05 g, 3.58 mmol). The crude mixture was purified by silica gel column chromatography using DCM/heptane (8:2) as eluent giving the compound **7m** (1.23 g, 87% yield) as a white foam. ^{1}H NMR ($CDCl_3$, 300 MHz) δ_H 7.81-7.75 (2H, m), 7.46-7.38 (3H, m), 7.29-7.24 (2H, m), 7.19-7.15 (3H, m), 3.06 (1H, dd, *J* = 16.5, 6.0 Hz), 2.80-2.56 (4H, m), 2.01-1.88 (1H, m), 1.84-1.71 (2H, m), 1.61 (9H, s), 1.59-1.49 (1H, m). $^{13}C\{^{1}H\}$ NMR ($CDCl_3$, 75 MHz) δ_C 168.8 (C), 152.6 (C), 150.6 (C), 141.8 (C), 135.5 (C), 130.4 (CH), 128.8 (CH), 128.5 (CH), 126.5 (CH), 126.0 (CH), 84.2 (C), 38.5 (CH), 35.8 (CH_2), 29.2 (CH_2), 28.7 (CH_2), 28.3 (CH_2), 28.1 (CH_3). HRMS (ESI+): calcd m/z for $C_{19}H_{21}N_2O$ [$(M+H-C_5H_9O_2)^+$]: 293.1648; Found: 293.1643.

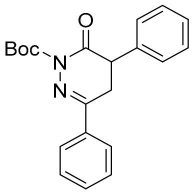

***tert*-butyl 6-oxo-3,5-diphenyl-5,6-dihydropyridazine-1(*4H*)-carboxylate** (**7n**) was synthesized following the general procedure using *N*-unsubstituted 4,5-dihydropyridazinone **3n** (896 mg, 3.58 mmol). The crude mixture was purified by silica gel column chromatography using DCM/heptane (8:2) as eluent giving the compound **7n** (1.08 g, 86% yield) as a white foam. ^{1}H NMR ($CDCl_3$, 300 MHz) δ_H 7.81-7.74 (2H, m), 7.42-7.21 (8H, m), 3.91-3.78 (1H, m), 3.39-3.20 (2H, m), 1.57 (9H, s). $^{13}C\{^{1}H\}$ NMR ($CDCl_3$, 75 MHz) δ_C 166.9 (C), 152.7 (C), 150.6 (C), 136.3 (C), 135.3 (C), 130.5 (CH), 129.1 (CH), 128.8 (CH), 128.04 (CH), 128.02 (CH), 126.5 (CH), 84.5 (C), 44.7 (CH), 30.5 (CH_2), 28.1 (CH_3). The analytical data match the published ones.[5]

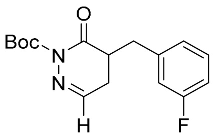

***tert*-butyl 5-(3-fluorobenzyl)-6-oxo-5,6-dihydropyridazine-1(*4H*)-carboxylate** (**8**) was synthesized following the general procedure using *N*-unsubstituted 4,5-dihydropyridazinone **6** (738.3 mg, 3.58 mmol). The crude mixture was purified by silica gel column chromatography using DCM/heptane (9:1)

[5] Li, X.; Gai, K.; Yuan, Z.; Wu, J.; Lin, A.; Yao, H. *Adv. Synth. Catal.* **2015**, *357*, 3479.

as eluent giving the compound **8** (691 mg, 63% yield) as a yellowish foam. ^{1}H NMR ($CDCl_3$, 300 MHz) δ_H 7.30-7.21 (2H, m), 6.96-6.86 (3H, m), 3.31 (1H, dd, *J* = 13.2, 4.1 Hz), 2.80-2.59 (2H, m), 2.47 (1H, ddd, *J* = 17.7, 6.4, 3.8 Hz), 2.24 (1H, ddd, *J* = 17.6, 10.8, 2.7 Hz), 1.58 (9H, s). ^{13}C{^{1}H} NMR ($CDCl_3$, 75 MHz) δ_C 167.8 (C), 163.0 (C, d, *J* = 244.5 Hz), 150.2 (C), 146.2 (CH), 140.3 (C, d, *J* = 7.5 Hz), 130.3 (CH, d, *J* = 8.2 Hz), 124.90 (CH, d, *J* = 3.0 Hz), 116.2 (CH, d, *J* = 21.0 Hz), 113.9 (CH, d, *J* = 21.0 Hz), 84.6 (C), 39.6 (CH), 35.1 (CH_2, d, *J* = 1.5 Hz), 28.0 (CH_3), 27.5 (CH_2). ^{19}F{^{1}H} NMR ($CDCl_3$, 282 MHz) δ_F -112.7. HRMS (ESI+): calcd m/z for $C_{11}H_{12}FN_2O$ [$(M+H-C_5H_9O_2)^+$]: 207.0928; Found: 207.0928.

III. Quaternary ammonium salt-catalyzed a-functionalization of pyridazinones

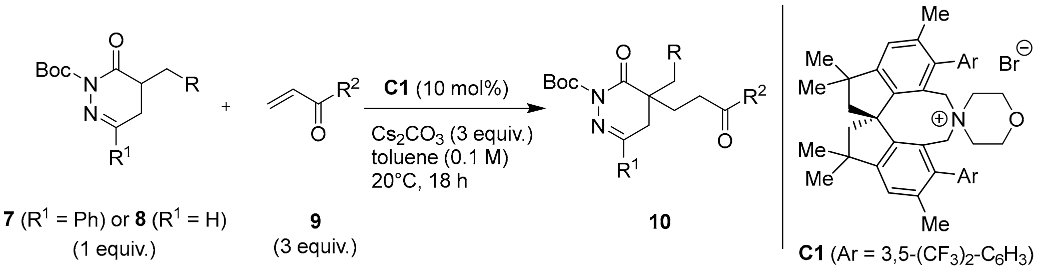

7 (R^1 = Ph) or 8 (R^1 = H) (1 equiv.) 9 (3 equiv.) 10

Typical procedure for α,α-functionalized pyridazinone derivatives preparation. In a 2 mL vial, spirobiindane-based salt **C1** (9.2 mg, 0.01 mmol) and Cs_2CO_3 (98 mg, 0.3 mmol) were introduced at room temperature. The *N*-Boc dihydropyridazinone derivative **7** or **8** (0.1 mmol) was dissolved in toluene (1 mL, 0.1 M) and added in the vial followed with the Michael acceptor **9** (0.3 mmol). The mixture was stirred at 1100 rpm at room temperature for 18 hours. The crude mixture was filtered through a pad of silica gel using AcOEt as eluent. Then, the crude mixture was purified by silica gel column chromatography giving the corresponding α,α-functionalized pyridazinone derivatives **10**.

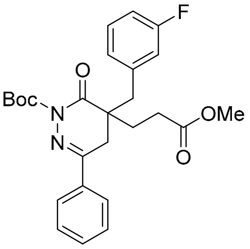

***tert*-butyl 5-(3-fluorobenzyl)-5-(3-methoxy-3-oxopropyl)-6-oxo-3-phenyl-5,6-dihydropyridazine-1(*4H*)-carboxylate** (**10a**) was synthesized following the general procedure using *N*-Boc dihydropyridazinone derivative **7a** (38.3 mg, 0.1 mmol) and methyl acrylate **9a** (27 µL, 0.3 mmol). The crude mixture was purified by silica gel column chromatography using DCM/AcOEt (98:2) as eluent giving the compound **10a** (25.3 mg, 54% yield) as a colorless oil. $[\alpha]_D^{20}$ +1.3 (*c* 0.1, $CHCl_3$). ^1H NMR ($CDCl_3$, 300 MHz) δ_H 7.74-7.72 (2H, m), 7.44-7.38 (3H, m), 7.21 (1H, dd, *J* = 14.1, 7.8 Hz); 6.96-6.85 (3H, m), 3.64 (3H, s), 3.19 (1H, d, *J* = 13.7 Hz); 2.83-2.74 (3H, m), 2.53 (1H, ddd, *J* = 16.2, 10.5, 5.6 Hz), 2.38 (1H, ddd, *J* = 16.2, 10.4, 5.7 Hz), 2.05 (1H, ddd, *J* = 15.9, 10.4, 5.6 Hz), 1.92 (1H, ddd, *J* = 14.4, 10.6, 5.8 Hz), 1.61 (9H, s). $^{13}C\{^1H\}$ NMR ($CDCl_3$, 75 MHz) δ_C 173.3 (C), 169.1 (C), 162.8 (C, d, *J* = 183.7 Hz), 151.4 (C), 150.7 (C), 138.1 (C, d, *J* = 5.3 Hz), 135.3 (C), 130.6 (CH), 130.0 (CH, d, *J* = 6 Hz), 128.8 (CH), 126.42 (CH), 126.39 (CH), 126.36 (CH), 117.5 (CH, d, *J* = 16.5 Hz), 114.4 (CH, d, *J* = 15.0 Hz) , 84.6 (C), 52.0 (CH), 44.0 (C), 41.0 (CH_2), 32.4 (CH_2), 30.4 (CH_2), 29.0 (CH_2), 28.0 (CH_3). $^{19}F\{^1H\}$ NMR ($CDCl_3$, 282 MHz) δ_F -112.9. HRMS (ESI+): calcd m/z for $C_{21}H_{22}FN_2O_3$ [$(M+H-C_5H_9O_2)^+$]: 369.1609; Found: 369.1614. HPLC analysis (19:81 er): CHIRALPAK AD-H, hexane/iPrOH = 9:1, flow rate 1.00 mL/min, 20°C, UV 254 nm, t_{min} = 13.4 min, t_{major} = 20.2 min.

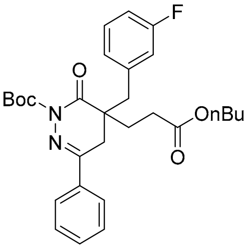

***tert*-butyl 5-(3-butoxy-3-oxopropyl)-5-(3-fluorobenzyl)-6-oxo-3-phenyl-5,6-dihydropyridazine-1(*4H*)-carboxylate** (**10b**) was synthesized following the general procedure using *N*-Boc dihydropyridazinone derivative **7a** (38.3 mg, 0.1 mmol) and butyl acrylate **9b** (43.0 µL, 0.3 mmol). The crude mixture was purified by silica gel column chromatography using DCM/hexane (95:5) as eluent

giving the compound **10a** (21.5 mg, 42% yield) as a colorless oil. ^{1}H NMR ($CDCl_3$, 300 MHz) δ_H 7.75-7.72 (2H, m), 7.46-7.37 (3H, m), 7.21 (1H, td, *J* = 7.9, 6.2 Hz), 6.97-6.85 (3H, m), 4.04 (2H, t, *J* = 6.7 Hz), 3.18 (1H, d, J = 13.6 Hz), 2.84-2.72 (3H, m), 2.53 (1H, ddd, *J* = 16.2, 10.6, 5.7 Hz), 2.36 (1H, ddd, *J* = 16.2, 10.3, 5.8 Hz); 2.05 (1H, ddd, *J* = 15.9, 10.3, 5.7 Hz), 1.91 (1H, ddd, *J* = 14.3, 10.6, 5.8 Hz), 1.62 (9H, s), 1.56 (2H, dd, *J* = 13.4, 5.3 Hz), 1.35 (2H, dq, *J* = 14.4, 7.3 Hz), 0.91 (3H, t, *J* = 7.3 Hz). ^{13}C{^{1}H} NMR ($CDCl_3$, 75 MHz) δ_C 173.0 (C), 169.2 (C), 162.8 (C, d, *J* = 244.5 Hz), 151.4 (C), 150.8 (C), 138.1 (C, d, *J* = 7.5 Hz), 135.3 (C), 130.6 (CH), 129.9 (CH, d, *J* = 8.2 Hz), 128.8 (CH), 126.43 (CH), 126.37 (CH), 117.5 (CH, d, *J* = 21.0 Hz), 114.3 (CH, d, *J* = 20.2 Hz), 84.6 (C), 64.8 (CH_2), 44.0 (C), 41.0 (CH_2), 32.5 (CH_2), 30.7 (CH_2), 30.4 (CH_2), 29.2 (CH_2), 28.0 (CH_3), 19.2 (CH_2), 13.8 (CH_3). ^{19}F{^{1}H} NMR ($CDCl_3$, 282 MHz) δ_F -112.9. HRMS (ESI+): calcd m/z for $C_{24}H_{28}FN_2O_3$ [$(M+H-C_5H_9O_2)^+$]: 411.2078; Found: 411.2089. HPLC analysis (25:75 er): CHIRALPAK AD-H, hexane/*i*PrOH = 9:1, flow rate 1.00 mL/min, 20°C, UV 254 nm, t_{min} = 9.5 min, t_{major} = 15.0 min.

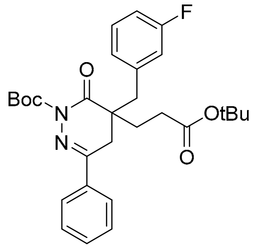

***tert*-butyl 5-(3-(tert-butoxy)-3-oxopropyl)-5-(3-fluorobenzyl)-6-oxo-3-phenyl-5,6-dihydropyridazine-1(*4H*)-carboxylate** (**10c**) was synthesized following the general procedure using *N*-Boc dihydropyridazinone derivative **7a** (38.3 mg, 0.1 mmol) and *tert*-butyl acrylate **9c** (44.0 µL, 0.3 mmol). The crude mixture was purified by silica gel column chromatography using DCM/hexane (95:5) as eluent giving the compound **10c** (27.6 mg, 54% yield) as a colorless oil. $[\alpha]_D^{20}$ +2.8 (*c* 0.6 , $CHCl_3$). ^{1}H NMR ($CDCl_3$, 300 MHz) δ_H 7.75-7.70 (2H, m), 7.45-7.36 (2H, m), 7.24-7.17 (1H, m), 6.96-6.84 (3H, m), 3.18 (1H, d, *J* = 13.6 Hz), 2.83-2.71 (m, 3H), 2.45 (1H, ddd, *J* = 16.2, 10.4, 5.7 Hz), 2.28 (1H, ddd, *J* = 16.2, 10.1, 6.0 Hz), 1.99 (1H, ddd, *J* = 15.8, 10.1, 5.7 Hz), 1.88 (1H, ddd, *J* = 14.5, 10.5, 6.1 Hz), 1.61 (9H, s), 1.42 (9H, s). ^{13}C{^{1}H} NMR ($CDCl_3$, 75 MHz) δ_C 172.2 (C), 169.2 (C), 162.8 (C, d, *J* = 244.5 Hz), 151.5 (C), 150.8 (C), 138.2 (C, d, *J* = 6.7 Hz), 135.4 (C), 130.5 (CH), 129.9 (CH, d, *J* = 8.2 Hz), 128.8 (CH), 126.42 (CH), 126.37 (CH), 117.5 (CH, d, *J* = 21.7 Hz), 114.3 (CH, d, *J* = 21.0 Hz), 84.5 (C), 80.9 (C), 44.0 (C), 41.0 (CH_2), 32.5 (CH_2), 30.31 (CH_2), 30.29 (CH_2), 28.2 (CH_3), 28.0 (CH_3). ^{19}F{^{1}H} NMR ($CDCl_3$, 282 MHz) δ_F -112.9. HRMS (ESI+): calcd m/z for $C_{24}H_{28}FN_2O_3$ [$(M+H-C_5H_9O_2)^+$]: 411.2078; Found: 411.2086. HPLC analysis (17:83 er): CHIRALPAK AD-H, hexane/*i*PrOH = 98:2, flow rate 1.00 mL/min, 20°C, UV 254 nm, t_{min} = 27.4 min, t_{major} = 30.8 min.

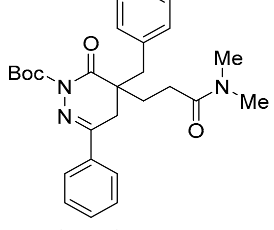

***tert*-butyl 5-(3-(dimethylamino)-3-oxopropyl)-5-(3-fluorobenzyl)-6-oxo-3-phenyl-5,6-dihydropyridazine-1(*4H*)-carboxylate** (**10d**) was synthesized following the general procedure using *N*-Boc dihydropyridazinone derivative **7a** (38.3 mg, 0.1 mmol) and *N,N*-dimethylacrylamide **9d** (31.0 µL, 0.3 mmol). The crude mixture was purified by silica gel column chromatography using AcOEt/hexane (1:9) as eluent giving the compound **10d** (12.1 mg, 25% yield) as a colorless oil. ^{1}H NMR ($CDCl_3$, 300 MHz) δ_H 7.75-7.71 (2H, m), 7.45-7.36 (3H, m), 7.23-7.16 (1H, m), 6.96-6.85 (3H, m), 3.22 (1H, d, *J* = 13.6 Hz), 2.99 (3H, s), 2.89 (3H, s), 2.82-2.73 (3H, m), 2.54 (1H, ddd, *J* = 15.7, 10.6, 5.4 Hz), 2.31 (1H, ddd, *J* = 15.4, 10.3, 5.5 Hz), 2.10 (1H, ddd, *J* = 15.6, 10.3, 5.4 Hz), 1.96 (1H, ddd, *J* = 14.3, 10.6, 5.5 Hz), 1.62 (9H, s). ^{13}C{^{1}H} NMR ($CDCl_3$, 75 MHz) δ_C 171.9 (C), 169.7 (C), 164.4 (C), 151.7 (C), 150.8 (C), 138.2 (C, d, *J* = 7.5 Hz), 135.3 (C), 130.6 (CH), 130.0 (CH), 129.9 (CH), 128.7 (CH), 126.4 (CH), 117.5 (CH, d, *J* = 21.0 Hz), 114.3 (CH, d, *J* = 21.0 Hz), 84.5 (C), 44.3 (C), 41.5 (CH_2), 37.4 (CH_3), 35.7 (CH_3), 32.4 (CH_2), 31.2 (CH_2),

28.2 (CH_2), 28.0 (CH_3). $^{19}F\{^1H\}$ NMR ($CDCl_3$, 282 MHz) δ_F -112.9. HRMS (ESI+): calcd m/z for $C_{22}H_{25}FN_3O_2$ [$(M+H-C_5H_9O_2)^+$]: 382.1925; Found: 382.1932. HPLC analysis (32:68 er): CHIRALPAK AD-H, hexane/*i*PrOH = 9:1, flow rate 1.00 mL/min, 20°C, UV 254 nm, t_{min} = 13.8 min, t_{major} = 15.2 min.

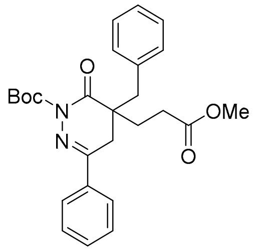

***tert*-butyl 5-benzyl-5-(3-methoxy-3-oxopropyl)-6-oxo-3-phenyl-5,6-dihydropyridazine-1(*4H*)-carboxylate** (**10e**) was synthesized following the general procedure using *N*-Boc dihydropyridazinone derivative **7b** (36.5 mg, 0.1 mmol) and methyl acrylate **9a** (27 µL, 0.3 mmol). The crude mixture was purified by silica gel column chromatography using DCM/AcOEt (98:2) as eluent giving the compound **10e** (24.4 mg, 54% yield) as a colorless oil. ^1H NMR ($CDCl_3$, 300 MHz) δ_H 7.75-7.72 (2H, m), 7.45-7.36 (3H, m), 7.26-7.22 (3H, m), 7.13-7.10 (2H, m), 3.64 (3H, s), 3.16 (1H, d, *J* = 13.6 Hz); 2.82-2.77 (3H, m), 2.56 (1H, ddd, *J* = 16.2, 10.7, 5.6 Hz), 2.38 (1H, ddd, *J* = 16.2, 10.5, 5.7 Hz), 2.07 (1H, ddd, *J* = 10.5, 9.1, 5.6 Hz), 1.90 (1H, ddd, *J* = 14.3, 10.7, 5.7 Hz), 1.62 (9H, s). $^{13}C\{^1H\}$ NMR ($CDCl_3$, 75 MHz) δ_C 173.4 (C), 169.4 (C), 151.4 (C), 150.8 (C), 135.4 (C), 135.4 (C, d, *J* = 2.2 Hz), 130.7 (2 CH), 130.5 (CH), 128.7 (CH), 128.5 (CH), 127.3 (CH), 126.4 (CH), 84.4 (C), 51.9 (CH_3), 43.9 (C), 41.1 (CH_2), 32.2 (CH_2), 30.1 (CH_2), 29.0 (CH_2), 28.0 (CH_3). HRMS (ESI+): calcd m/z for $C_{21}H_{23}N_2O_3$ [$(M+H-C_5H_9O_2)^+$]: 351.1703; Found: 351.1699. HPLC analysis (33:67 er): CHIRALPAK AD-H, hexane/*i*PrOH = 9:1, flow rate 1.00 mL/min, 20°C, UV 254 nm, t_{min} = 13.6 min, t_{major} = 17.5 min.

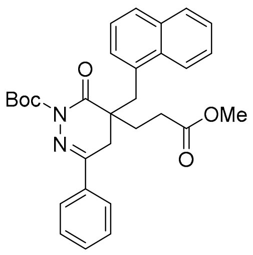

tert-butyl 5-(3-methoxy-3-oxopropyl)-5-(naphthalen-1-ylmethyl)-6-oxo-3-phenyl-5,6-dihydropyridazine-1(_4H_)-carboxylate (**10f**) was synthesized following the general procedure using _N_-Boc dihydropyridazinone derivative **7c** (41.5 mg, 0.1 mmol) and methyl acrylate **9a** (27 µL, 0.3 mmol). The crude mixture was purified by silica gel column chromatography using DCM/heptane (9:1) as eluent giving the compound **10f** (22.0 mg, 44% yield) as a yellowish oil. ^{1}H NMR ($CDCl_3$, 300 MHz) δ_H 8.00-7.97 (1H, m), 7.84-7.79 (1H, m), 7.75-7.72 (1H, m), 7.59-7.56 (2H, m), 7.45-7.29 (7H, m), 3.64 (3H, s), 3.37 (1H, d, _J_ = 14.2 Hz), 2.84-2.71 (2H, m), 2.59 (1H, ddd, _J_ = 15.9, 10.5, 5.5 Hz), 2.41 (1H, ddd, _J_ = 15.9, 10.3, 5.5 Hz), 2.31-2.21 (1H, m), 2.04-1.92 (1H, m), 1.60 (9H, s). ^{13}C{^{1}H} NMR ($CDCl_3$, 75 MHz) δ_C 173.4 (C), 169.5 (C), 151.6 (C), 150.7 (C), 135.4 (C), 134.0 (C), 133.0 (C), 132.1 (C), 130.3 (CH), 129.10 (CH), 129.08 (CH), 128.6 (CH), 128.2 (CH), 126.3 (CH), 125.7 (CH), 125.4 (CH), 123.8 (CH), 84.3 (C), 51.9 (CH_3), 45.0 (C), 36.7 (CH_2), 32.3 (CH_2), 31.4 (CH_2), 29.3 (CH_2), 28.0 (CH_3). HRMS (ESI+): calcd m/z for $C_{25}H_{25}N_2O_3$ [$(M+H-C_5H_9O_2)^+$]: 401.1860; Found: 401.1864. HPLC analysis (30:70 er): CHIRALPAK AD-H, hexane/_i_PrOH = 9:1, flow rate 1.00 mL/min, 20°C, UV 254 nm, t_{min} = 10.6 min, t_{major} = 14.5 min.

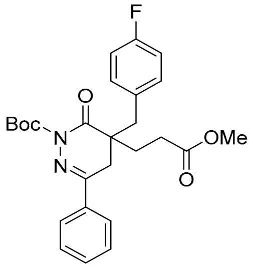

tert-butyl 5-(4-fluorobenzyl)-5-(3-methoxy-3-oxopropyl)-6-oxo-3-phenyl-5,6-dihydropyridazine-1(_4H_)-carboxylate (**10g**) was synthesized following the general procedure using _N_-Boc dihydropyridazinone derivative **7d** (38.3 mg, 0.1 mmol) and methyl acrylate **9a** (27 µL, 0.3 mmol). The crude mixture was purified by silica gel column chromatography using DCM/AcOEt (98:2) as eluent giving the compound **10g** (32.8 mg, 70% yield) as a colorless oil. ^{1}H NMR ($CDCl_3$, 300 MHz) δ_H 7.75-7.71 (2H, m), 7.44-7.40 (3H, m), 7.11-7.07 (2H, m), 6.96-6.88 (2H, m), 3.65 (3H, s), 3.14 (1H, d, _J_ = 13.8 Hz), 2.77-2.73 (3H, m); 2.54 (1H, ddd, _J_ = 16.2, 10.6, 5.6 Hz), 2.38 (1H, ddd, _J_ = 16.2, 10.4, 5.8 Hz), 2.06 (1H, ddd, _J_ = 16.0, 10.3, 5.6 Hz), 1.90 (1H, ddd, _J_ = 14.2, 10.6, 5.8 Hz), 1.61 (9H, s). ^{13}C{^{1}H} NMR ($CDCl_3$, 75 MHz) δ_C 173.4 (C), 169.2 (C), 162.2 (C, d, _J_ = 244.5 Hz), 151.3 (C), 150.7 (C), 135.3 (C), 132.2 (CH), 132.1 (CH), 131.2 (C, d, _J_ = 3 Hz), 130.6 (CH), 128.8 (CH), 126.4 (CH), 115.5 (CH), 115.3 (CH), 84.5 (C), 52.0 (CH_3), 43.9 (C), 40.5 (CH_2), 32.3 (CH_2), 30.2 (CH_2), 29.0 (CH_2), 28.0 (CH_3). ^{19}F{^{1}H} NMR ($CDCl_3$, 282 MHz) δ_F -115.4. HRMS (ESI+): calcd m/z for $C_{21}H_{22}FN_2O_3$ [$(M+H-C_5H_9O_2)^+$]: 369.1609; Found: 369.1616. HPLC analysis (23:77 er): CHIRALPAK AD-H, hexane/iPrOH = 9:1, flow rate 1.00 mL/min, 20°C, UV 254 nm, t_{min} = 14.3 min, t_{major} = 16.6 min.

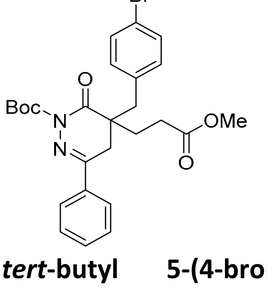

tert-butyl 5-(4-bromobenzyl)-5-(3-methoxy-3-oxopropyl)-6-oxo-3-phenyl-5,6-dihydropyridazine-1(_4H_)-carboxylate (**10h**) was synthesized following the general procedure using _N_-Boc dihydropyridazinone derivative **7e** (44.4 mg, 0.1 mmol) and methyl acrylate **9a** (27 µL, 0.3 mmol). The

crude mixture was purified by silica gel column chromatography using DCM/AcOEt (98:2) as eluent giving the compound **10h** (38.2 mg, 72% yield) as a colorless oil. ^{1}H NMR ($CDCl_3$, 300 MHz) δ_H 7.74-7.71 (2H, m), 7.46-7.35 (5H, m), 7.01-6.99 (2H, m), 3.64 (3H, s), 3.13 (1H, d, *J* = 13.7 Hz), 2.82-2.70 (3H, m), 2.53 (1H, ddd, *J* = 16.2, 10.5, 5.7 Hz), 2.37 (1H, ddd, *J* = 16.2, 10.3, 5.8 Hz), 2.04 (1H, ddd, *J* = 15.9, 10.3, 5.7 Hz), 1.94-1.82 (1H, m), 1.61 (9H, s). ^{13}C{^{1}H} NMR ($CDCl_3$, 75 MHz) δ_C 173.3 (C), 169.1 (C), 151.3 (C), 150.7 (C), 135.3 (C), 134.5 (C), 132.3 (CH), 131.7 (CH), 130.6 (CH), 128.8 (CH), 126.4 (CH), 121.5 (C), 84.5 (C), 52.0 (CH_3), 43.8 (C), 40.6 (CH_2), 32.3 (CH_2), 30.2 (CH_2), 29.0 (CH_2), 28.0 (CH_3). HRMS (ESI+): calcd m/z for $C_{21}H_{22}BrN_2O_3$ [(M+H-$C_5H_9O_2$)$^+$]: 429.0808; Found: 429.0814. HPLC analysis (27:73 er): CHIRALPAK AD-H, hexane/iPrOH = 9:1, flow rate 1.00 mL/min, 20°C, UV 254 nm, t_{min} = 14.3 min, t_{major} = 17.2 min.

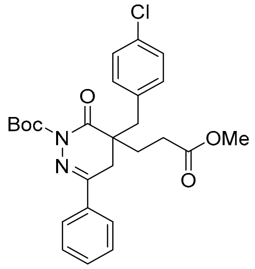

***tert*-butyl 5-(4-chlorobenzyl)-5-(3-methoxy-3-oxopropyl)-6-oxo-3-phenyl-5,6-dihydropyridazine-1(*4H*)-carboxylate** (**10i**) was synthesized following the general procedure using *N*-Boc dihydropyridazinone derivative **7f** (39.9 mg, 0.1 mmol) and methyl acrylate **9a** (27 µL, 0.3 mmol). The crude mixture was purified by silica gel column chromatography using DCM/AcOEt (98:2) as eluent giving the compound **10i** (24.8 mg, 51% yield) as a colorless oil. ^{1}H NMR ($CDCl_3$, 300 MHz) δ_H 7.74-7.69 (2H, m), 7.44-7.37 (3H, m), 7.23-7.20 (2H, m), 7.07-7.05 (2H, m), 3.64 (3H, s), 3.14 (1H, d, *J* = 13.7 Hz), 2.82-2.66 (3H, m), 2.55 (1H, ddd, *J* = 16.2, 11.1, 5.8 Hz), 2.38 (1H, ddd, *J* = 16.2, 10.3, 5.8 Hz), 2.04 (1H, ddd, *J* = 15.9, 10.3, 5.7 Hz), 1.94-1.82 (1H, m), 1.61 (9H, s). ^{13}C{^{1}H} NMR ($CDCl_3$, 75 MHz) δ_C 173.3 (C), 169.1 (C), 151.3 (C), 150.7 (C), 135.3 (C), 134.0 (C), 133.4 (C), 132.0 (2 CH), 130.6 (CH), 128.8 (CH), 128.7 (CH), 126.4 (CH), 84.5 (C), 52.0 (CH), 43.9 (C), 40.6 (CH_2), 32.3 (CH_2), 30.2 (CH_2), 29.0 (CH_2), 28.0 (CH_3). HRMS (ESI+): calcd m/z for $C_{21}H_{22}ClN_2O_3$ [(M+H-$C_5H_9O_2$)$^+$]: 385.1313; Found: 385.1310. HPLC analysis (24:76 er): CHIRALPAK AD-H, hexane/iPrOH = 9:1, flow rate 1.00 mL/min, 20°C, UV 254 nm, t_{min} = 14.2 min, t_{major} = 16.8 min.

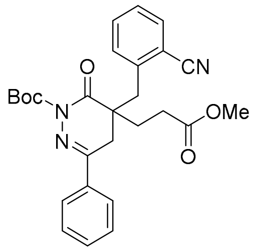

***tert*-butyl 5-(2-cyanobenzyl)-5-(3-methoxy-3-oxopropyl)-6-oxo-3-phenyl-5,6-dihydropyridazine-1(*4H*)-carboxylate** (**10j**) was synthesized following the general procedure using *N*-Boc dihydropyridazinone derivative **7g** (39.0 mg, 0.1 mmol) and methyl acrylate **9a** (27 µL, 0.3 mmol). The crude mixture was purified by silica gel column chromatography using DCM/AcOEt (98:2) as eluent giving the compound **10j** (37.6 mg, 79% yield) as a colorless oil. $[\alpha]_D^{20}$ -24.3 (*c* 1.0, $CHCl_3$). ^{1}H NMR ($CDCl_3$, 300 MHz) δ_H 7.75-7.72 (2H, m), 7.60-7.48 (3H, m), 7.45-7.31 (4H, m), 3.74-3.58 (4H, m), 3.07 (2H, dd, *J* = 15.3, 13.6 Hz), 2.72 (1H, d, *J* = 17.0 Hz), 2.57-2.41 (2H, m), 2.14-1.97 (2H, m), 1.61 (9H, s). ^{13}C{^{1}H} NMR ($CDCl_3$, 75 MHz) δ_C 172.9 (C), 168.5 (C), 151.5 (C), 150.6 (C), 140.3 (C), 135.0 (C), 133.2 (CH), 132.9 (CH), 131.6 (CH), 130.7 (CH), 128.8 (CH), 128.0 (CH), 126.4 (CH), 118.7 (C), 114.3 (C), 84.6 (C), 52.0 (CH_3), 45.1 (C), 38.7 (CH_2), 32.0 (CH_2), 31.3 (CH_2), 28.9 (CH_2), 28.0 (CH_3). HRMS (ESI+): calcd m/z for $C_{22}H_{22}N_3O_3$ [(M+H-$C_5H_9O_2$)$^+$]: 376.1656; Found: 376.1654. HPLC analysis (15:85 er): CHIRALPAK AD-H, hexane/*i*PrOH = 9:1, flow rate 1.00 mL/min, 20°C, UV 254 nm, t_{min} = 11.9 min, t_{major} = 16.9 min.

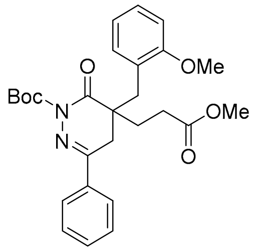

***tert*-butyl 5-(3-methoxy-3-oxopropyl)-5-(2-methoxybenzyl)-6-oxo-3-phenyl-5,6-dihydropyridazine-1(*4H*)-carboxylate** (**10k**) was synthesized following the general procedure using *N*-Boc dihydropyridazinone derivative **7h** (39.5 mg, 0.1 mmol) and methyl acrylate **9a** (27 µL, 0.3 mmol). The crude mixture was purified by silica gel column chromatography using DCM/AcOEt (98:2) as eluent giving the compound **10k** (11.6 mg, 24% yield) as a colorless oil. ^{1}H NMR ($CDCl_3$, 300 MHz) δ_H 7.71-7.64 (2H, m), 7.42-7.34 (3H, m), 7.22-7.10 (2H, m), 6.84 (1H, td, *J* = 7.4, 0.8 Hz), 6.74 (1H, d, *J* = 8.1 Hz), 3.65 (3H, s), 3.53 (3H, s), 3.03 (2H, s), 2.83 (2H, dd, *J* = 17.2, 10.5 Hz, 2H), 2.58 (1H, ddd, *J* = 16.2, 10.9, 5.4 Hz), 2.40 (1H, ddd, *J* = 16.1, 10.7, 5.4 Hz), 2.13 (1H, ddd, *J* = 14.2, 10.8, 5.4 Hz), 1.89 (1H, ddd, *J* = 14.1, 10.9, 5.4 Hz), 1.61 (9H, s). $^{13}C\{^{1}H\}$ NMR ($CDCl_3$, 75 MHz) δ_C 173.7 (C), 169.8 (C), 157.9 (C), 151.5 (C), 150.9 (C), 135.9 (C), 132.4 (CH), 130.2 (CH), 128.65 (CH), 128.56 (CH), 126.3 (CH), 124.0 (C), 120.5 (CH), 110.4 (CH), 84.1 (C), 54.9 (CH_3), 51.9 (CH_3), 44.2 (C), 35.0 (CH_2), 32.0 (CH_2), 30.5 (CH_2), 29.2 (CH_2), 28.1 (CH_3). HRMS (ESI+): calcd m/z for $C_{22}H_{25}N_2O_4$ [$(M+H-C_5H_9O_2)^+$]: 381.1809; Found: 381.1791. HPLC analysis (39:61 er): CHIRALPAK AD-H, hexane/*i*PrOH = 9:1, flow rate 1.00 mL/min, 20°C, UV 254 nm, t_{min} = 14.6 min, t_{major} = 17.4 min.

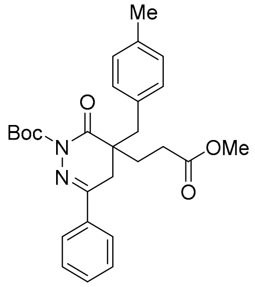

***tert*-butyl 5-(3-methoxy-3-oxopropyl)-5-(4-methylbenzyl)-6-oxo-3-phenyl-5,6-dihydropyridazine-1(*4H*)-carboxylate** (**10l**) was synthesized following the general procedure using *N*-Boc dihydropyridazinone derivative **7i** (37.9 mg, 0.1 mmol) and methyl acrylate **9a** (27 µL, 0.3 mmol). The crude mixture was purified by silica gel column chromatography using DCM/AcOEt (98:2) as eluent giving the compound **10l** (15.4 mg, 33% yield) as a colorless oil. ^{1}H NMR ($CDCl_3$, 300 MHz) δ_H 7.75-7.72 (2H, m), 7.43-7.36 (3H, m), 7.06-6.98 (4H, m); 3.64 (3H, s), 3.10 (1H, d, *J* = 13.7 Hz), 2.83-2.70 (3H, m), 2.55 (1H, ddd, *J* = 16.3, 10.8, 5.5 Hz), 2.37 (1H, ddd, *J* = 16.3, 10.6, 5.7 Hz), 2.30 (3H, s), 2.05 (1H, ddd, *J* = 14.3, 10.7, 5.6 Hz), 1.88 (1H, ddd, *J* = 14.3, 10.8, 5.7 Hz), 1.62 (9H, s). $^{13}C\{^{1}H\}$ NMR ($CDCl_3$, 75 MHz) δ_C 173.4 (C), 169.6 (C), 151.5 (C), 150.8 (C), 136.9 (C), 135.5 (C), 132.3 (C), 130.6 (CH), 130.4 (CH), 129.2 (CH), 128.7 (CH), 126.5 (CH), 84.3 (C), 51.9 (CH_3), 43.9 (C), 40.7 (CH_2), 32.2 (CH_2), 30.1 (CH_2), 29.1 (CH_2), 28.1 (CH_3), 21.1 (CH_3). HRMS (ESI+): calcd m/z for $C_{22}H_{25}N_2O_3$ [$(M+H-C_5H_9O_2)^+$]: 365.1860; Found: 365.1854. HPLC analysis (36:64 er): CHIRALPAK AD-H, hexane/*i*PrOH = 9:1, flow rate 1.00 mL/min, 20°C, UV 254 nm, t_{min} = 10.8 min, t_{major} = 14.3 min.

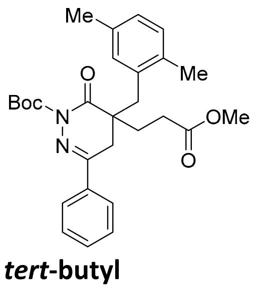

***tert*-butyl 5-(2,5-dimethylbenzyl)-5-(3-methoxy-3-oxopropyl)-6-oxo-3-phenyl-5,6-dihydropyridazine-1(*4H*)-carboxylate** (**10m**) was synthesized following the general procedure using *N*-Boc dihydropyridazinone derivative **7j** (39.3 mg, 0.1 mmol) and methyl acrylate **9a** (27 µL, 0.3 mmol).

The crude mixture was purified by silica gel column chromatography using DCM/AcOEt (98:2) as eluent giving the compound **10m** (19.2 mg, 40% yield) as a colorless oil. ^{1}H NMR ($CDCl_3$, 300 MHz) δ_H 7.74-7.68 (2H, m), 7.45-7.36 (3H, m), 7.00 (1H, d, *J* = 7.7 Hz), 6.91 (1H, d, *J* = 7.8 Hz), 6.84 (1H, s), 3.64 (3H, s), 3.16 (1H, d, *J* = 14.0 Hz), 2.87-2.75 (3H, m), 2.54 (1H, ddd, *J* = 16.0, 10.7, 5.4 Hz), 2.37 (1H, ddd, *J* = 16.0, 10.5, 5.4 Hz), 2.25 (3H, s), 2.23-2.15 (1H, m), 2.12 (3H, s), 1.97-1.83 (1H, m), 1.62 (9H, s). ^{13}C{^{1}H} NMR ($CDCl_3$, 75 MHz) δ_C 173.5 (C), 169.6 (C), 151.7 (C), 150.9 (C), 135.51 (C), 135.49 (C), 133.99 (C), 133.93 (C), 131.8 (CH_3), 130.8 (CH), 130.4 (CH), 128.7 (CH), 128.0 (CH), 126.4 (CH), 84.4 (C), 51.9 (CH_3), 44.9 (C), 37.7 (CH_2), 31.5 (CH_2), 31.2 (CH_2), 29.2 (CH_2), 28.1 (CH_3), 20.9 (CH_3), 20.0 (CH_3). HRMS (ESI+): calcd m/z for $C_{23}H_{27}N_2O_3$ [(M+H-$C_5H_9O_2$)$^+$]: 379.2016; Found: 379.2016. HPLC analysis (33:67 er): CHIRALPAK AD-H, hexane/*i*PrOH = 9:1, flow rate 1.00 mL/min, 20°C, UV 254 nm, t_{min} = 7.0 min, t_{major} = 8.9 min.

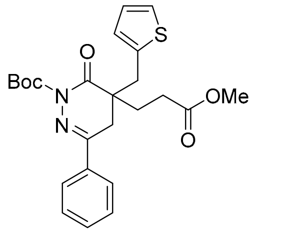

***tert*-butyl 5-(3-methoxy-3-oxopropyl)-6-oxo-3-phenyl-5-(thiophen-2-ylmethyl)-5,6-dihydropyridazine-1(*4H*)-carboxylate** (**10n**) was synthesized following the general procedure using *N*-Boc dihydropyridazinone derivative **7k** (37.1 mg, 0.1 mmol) and methyl acrylate **9a** (27 µL, 0.3 mmol). The crude mixture was purified by silica gel column chromatography using DCM/AcOEt (98:2) as eluent giving the compound **10n** (34.3 mg, 75% yield) as a colorless oil. ^{1}H NMR ($CDCl_3$, 300 MHz) δ_H 7.77-7.71 (2H, m), 7.45-7.36 (3H, m), 7.17 (1H, dd, *J* = 5.2, 1.0 Hz), 6.93 (1H, dd, *J* = 5.1, 3.5 Hz), 6.82 (1H, d, *J* = 2.9 Hz), 3.63 (3H, s), 3.42 (1H, dd, *J* = 14.8 Hz), 3.02 (1H, d, *J* = 14.8 Hz), 2.91-2.79 (2H, m), 2.60-2.34 (2H, m), 2.04-1.94 (2H, m), 1.61 (9H, s). ^{13}C{^{1}H} NMR ($CDCl_3$, 75 MHz) δ_C 173.2 (C), 169.2 (C), 151.5 (C), 150.7 (C), 137.0 (C), 135.4 (C), 130.5 (CH), 128.7 (CH), 128.4 (CH), 127.2 (CH), 126.5 (CH), 125.3 (CH), 84.5 (C), 51.9 (CH_3), 43.8 (C), 34.7 (CH_2), 32.6 (CH_2), 30.1 (CH_2), 29.0 (CH_2), 28.1 (CH_3). HRMS (ESI+): calcd m/z for $C_{19}H_{21}N_2O_3S$ [(M+H-$C_5H_9O_2$)$^+$]: 357.1267; Found: 357.1278. HPLC analysis (33:67 er): CHIRALPAK AD-H, hexane/*i*PrOH = 9:1, flow rate 1.00 mL/min, 20°C, UV 254 nm, t_{min} = 16.2 min, t_{major} = 19.9 min.

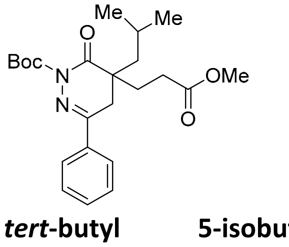

***tert*-butyl 5-isobutyl-5-(3-methoxy-3-oxopropyl)-6-oxo-3-phenyl-5,6-dihydropyridazine-1(*4H*)-carboxylate** (**10o**) was synthesized following the general procedure using *N*-Boc dihydropyridazinone derivative **7m** (33.1 mg, 0.1 mmol) and methyl acrylate **9a** (27 µL, 0.3 mmol). The crude mixture was purified by silica gel column chromatography using DCM/AcOEt (98:2) as eluent giving the compound **10o** (22.5 mg, 54% yield) as a colorless oil. ^{1}H NMR ($CDCl_3$, 300 MHz) δ_H 7.82-7.78 (2H,m), 7.45-7.40 (3H, m), 3.65 (3H, s), 2.88 (2H, dd, *J* = 18.0, 15.0 Hz); 2.45 (1H, ddd, *J* = 16.3, 10.7, 5.8 Hz), 2.33 (1H, ddd, *J* = 16.1, 10.2, 5.9 Hz), 2.07 (1H, ddd, *J* = 16.0, 10.2, 5.8 Hz), 1.95 (1H, ddd, *J* = 14.3, 10.7, 5.9 Hz), 1.77 (1H, m), 1.64 (1H, dd, *J* = 14.4, 5.8 Hz), 1.59 (9H, s), 1.52 (1H, dd, *J* = 14.4, 6.2 Hz), 0.94-0.89 (6H, t, *J* = 6.8 Hz). ^{13}C{^{1}H} NMR ($CDCl_3$, 75 MHz) δ_C 173.6 (C), 170.3 (C), 151.6 (C), 150.9 (C), 135.7 (C), 130.4 (CH), 128.8 (2 CH), 126.4 (2 CH), 84.2 (C), 51.9 (CH_3), 43.6 (CH_2), 42.3 (C), 33.5 (CH_2), 30.4 (CH_2), 29.0 (CH_2), 28.0 (3 CH_3), 24.4 (CH), 24.28 (CH_3), 24.26 (CH_3). HRMS (ESI+): calcd m/z for $C_{18}H_{25}N_2O_3$ [(M+H-$C_5H_9O_2$)$^+$]: 317.1860; Found: 317.1865. HPLC analysis (45:55 er): CHIRALPAK AD-H, hexane/*i*PrOH = 9:1, flow rate 1.00 mL/min, 20°C, UV 254 nm, t_{min} = 8.1 min, t_{major} = 9.7 min.

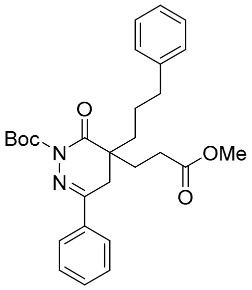

***tert*-butyl 5-(3-methoxy-3-oxopropyl)-6-oxo-3-phenyl-5-(3-phenylpropyl)-5,6-dihydropyridazine-1(*4H*)-carboxylate** (**10p**) was synthesized following the general procedure using *N*-Boc dihydropyridazinone derivative **7l** (39.3 mg, 0.1 mmol) and methyl acrylate **9a** (27 µL, 0.3 mmol). The crude mixture was purified by silica gel column chromatography using DCM/heptane (9:1) as eluent giving the compound **10p** (6.7 mg, 14% yield) as a colorless oil. ^{1}H NMR ($CDCl_3$, 300 MHz) δ_H 7.72-7.66 (2H, m), 7.39-7.30 (3H, m), 7.21-7.03 (5H, m), 3.57 (3H, s), 2.84-2.71 (2H, m), 2.53-2.49 (2H, m), 2.38-2.19 (2H, m), 1.99-1.83 (2H, m), 1.61-1.56 (4H, m), 1.52 (9H, s). $^{13}C\{^{1}H\}$ NMR ($CDCl_3$, 75 MHz) δ_C 173.5 (C), 169.9 (C), 151.5 (C), 150.8 (C), 141.6 (C), 135.6 (C), 130.5 (CH), 128.8 (CH), 128.5 (CH), 128.4 (CH), 126.4 (CH), 126.1 (CH), 84.3 (C), 51.9 (CH_3), 42.3 (C), 36.1 (CH_2), 34.1 (CH_2), 33.1 (CH_2), 29.5 (CH_2), 28.9 (CH_2), 28.0 (CH_3), 25.5 (CH_2). HRMS (ESI+): calcd m/z for $C_{23}H_{27}N_2O_3$ [$(M+H-C_5H_9O_2)^+$]: 379.2016 ; Found: 379.1988. HPLC analysis (59:41 er): CHIRALPAK AD-H, hexane/*i*PrOH = 9:1, flow rate 1.00 mL/min, 20°C, UV 254 nm, t_{major} = 10.9 min, t_{min} = 12.6 min.

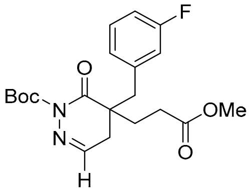

***tert*-butyl 5-(3-fluorobenzyl)-5-(3-methoxy-3-oxopropyl)-6-oxo-5,6-dihydropyridazine-1(*4H*)-carboxylate** (**10q**) was synthesized following the general procedure using *N*-Boc dihydropyridazinone derivative **8** (30.7 mg, 0.1 mmol) and methyl acrylate **9a** (27 µL, 0.3 mmol). The crude mixture was purified by silica gel column chromatography using DCM/AcOEt (95:5) as eluent giving the compound **10q** (18.5 mg, 47% yield) as a colorless oil. $[\alpha]_D^{20}$ -4.9 (*c* 0.5, $CHCl_3$). ^{1}H NMR ($CDCl_3$, 300 MHz) δ_H 7.28-7.18 (2H, m), 7.00-6.85 (3H, m), 3.66 (3H, s), 3.09 (1H, d, *J* = 13.7 Hz), 2.72 (1H, d, *J* = 13.7 Hz), 2.49 (1H, ddd, *J* = 16.2, 10.4, 5.8 Hz), 2.37-2.28 (3H, m), 1.99 (1H, ddd, *J* = 15.9, 10.2, 5.8 Hz), 1.87 (1H, ddd, *J* = 14.3, 10.4, 5.9 Hz), 1.58 (9H, s). $^{13}C\{^{1}H\}$ NMR ($CDCl_3$, 75 MHz) δ_C 173.2 (C), 169.0 (C), 162.8 (C, d, *J* = 244.5 Hz), 150.6 (C), 145.3 (CH), 138.0 (C, d, *J* = 7.5 Hz), 130.0 (CH, d, *J* = 8.2 Hz), 126.3 (CH, d, *J* = 2.2 Hz), 117.4 (CH, d, *J* = 21.0 Hz), 114.4 (CH, d, *J* = 21.0 Hz), 84.7 (C), 52.0 (CH_3), 43.9 (C), 40.7 (CH_2, d, *J* = 1.5 Hz), 32.8 (CH_2), 30.2(CH_2), 28.9 (CH_2), 28.0 (CH_3). $^{19}F\{^{1}H\}$ NMR ($CDCl_3$, 282 MHz) δ_F -112.8. HRMS (ESI+): calcd m/z for $C_{15}H_{18}FN_2O_3$ [$(M+H-C_5H_9O_2)^+$]: 293.1296; Found: 293.1301. HPLC analysis (78:22 er): CHIRALPAK AD-H, hexane/*i*PrOH = 9:1, flow rate 1.00 mL/min, 20°C, UV 254 nm, t_{major} = 17.5 min, t_{min} = 20.4 min.

tert-butyl 5-(3-methoxy-3-oxopropyl)-6-oxo-3,5-diphenyl-5,6-dihydropyridazine-1(*4H*)-carboxylate (10r)

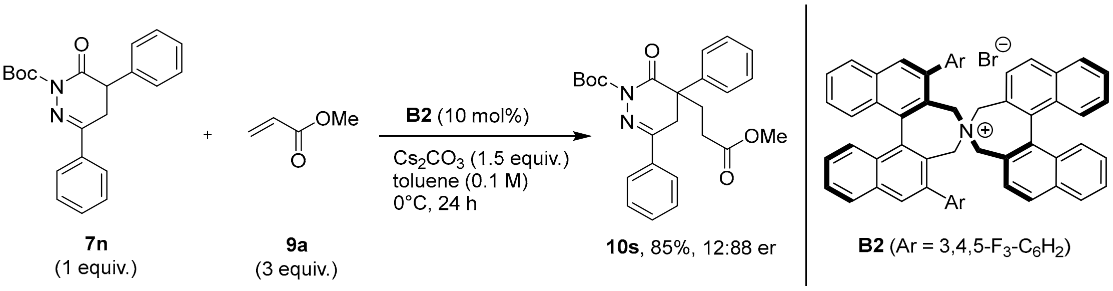

In a 2 mL vial, Maruoka catalyst **B2** (9.2 mg, 0.01 mmol) and Cs_2CO_3 (48.9 mg, 0.15 mmol) were introduced at room temperature. The *N*-Boc dihydropyridazinone derivative **7n** (35.1 mg, 0.1 mmol) was dissolved in toluene (1 mL, 0.1 M) and added in the vial followed with methyl acrylate **9a** (27 µL, 0.3 mmol). The mixture was stirred at 1100 rpm at 0°C for 24 hours. The crude mixture was filtered through a pad of silica gel using AcOEt as eluent. Then, the crude mixture was purified by silica gel column chromatography using DCM/AcOEt (98:2) giving α,α-functionalized pyridazinone **10r** (37.1 mg, 85% yield) as a white solid. $[\alpha]_D^{20}$ -27.6 (*c* 0.2, $CHCl_3$). ^1H NMR ($CDCl_3$, 300 MHz) δ_H 7.78-7.71 (2H, m), 7.41-7.36 (3H, m), 7.28-7.19 (5H, m), 3.67 (1H, d, *J* = 16.9 Hz), 3.60 (3H, s), 2.96 (1H, d, *J* = 17.0 Hz), 2.48-2.16 (4H, m), 1.59 (9H, s). $^{13}C\{^1H\}$ NMR ($CDCl_3$, 75 MHz) δ_C 173.6 (C), 169.3 (C), 151.7 (C), 150.7 (C), 136.9 (C), 135.5 (C), 130.4 (CH), 129.1 (CH), 128.8 (CH), 128.1 (CH), 126.3 (CH), 126.2 (CH), 84.4 (C), 51.8 (CH_3), 47.3 (C), 33.8 (CH_2), 32.9 (CH_2), 29.8 (CH_2), 28.0 (CH_3). HRMS (ESI+): calcd m/z for $C_{20}H_{21}N_2O_3$ $[(M+H-C_5H_9O_2)^+]$: 337.1547; Found: 337.1551. HPLC analysis (12:88 er): CHIRALPAK AD-H, hexane/*i*PrOH = 8:2, flow rate 1.00 mL/min, 20°C, UV 254 nm, t_{min} = 8.0 min, t_{major} = 9.6 min.

tert-butyl 5-((3,5-di-tert-butyl-4-hydroxyphenyl)(phenyl)methyl)-5-(3-fluorobenzyl)-6-oxo-3-phenyl-5,6-dihydropyridazine-1(*4H*)-carboxylate (12)

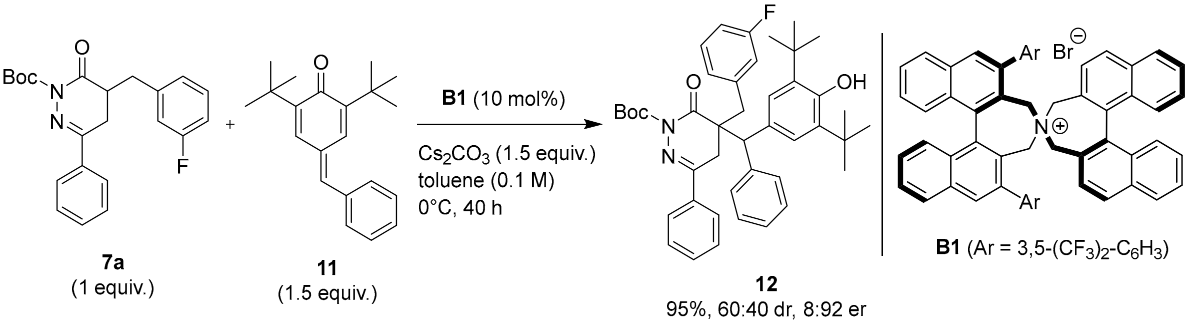

In a 2 mL vial, Maruoka catalyst **B1** (10.8 mg, 0.01 mmol), Cs_2CO_3 (48.9 mg, 0.15 mmol) and *p*-QM **11** (44.2 mg, 0.15 mmol) were introduced at room temperature. The *N*-Boc dihydropyridazinone derivative **7a** (38.3 mg, 0.1 mmol) was dissolved in toluene (1 mL, 0.1 M) and added in the vial. The mixture was stirred at 1100 rpm at 0°C for 40 hours. The crude mixture was filtered through a pad of silica gel using AcOEt as eluent. Then, the crude mixture was purified by silica gel column chromatography using DCM/AcOEt (98:2) giving α,α-functionalized pyridazinone **12** (64.3 mg, 95% yield, 60:40 dr) as a white solid. The main diastereoisomer was separated with a semipreparative HPLC using DCM as eluent. $[\alpha]_D^{20}$ +40.7 (*c* 0.7, $CHCl_3$). ^1H NMR ($CDCl_3$, 300 MHz) δ_H 7.46-7.39 (4H, m), 7.35-7.27 (3H, m), 7.24-7.17 (3H, m), 7.13-7.07 (3H, m), 6.91-6.81 (3H, m), 5.08 (1H, s), 4.33 (1H, s), 3.45 (1H, d, *J* = 13.5 Hz), 3.18 (1H, d, *J* = 17.3 Hz), 2.85 (1H, d, *J* = 17.2 Hz), 2.64 (1H, d, *J* = 13.5 Hz), 1.61 (9H, s), 1.37 (18H, s). $^{13}C\{^1H\}$ NMR ($CDCl_3$, 75 MHz) δ_C 169.1 (C), 162.7 (C, d, *J* = 244.5 Hz), 153.1 (C), 151.0 (C), 140.5 (C), 139.2 (C), 139.1 (C), 135.7 (C), 135.4 (C), 130.3 (CH), 130.0 (CH, d, *J* = 9.0 Hz), 129.7 (CH, d, *J* = 8.2 Hz), 128.5 (CH), 128.4 (CH), 127.0 (CH), 126.55 (CH), 126.49 (CH, d, *J* = 2.2 Hz), 126.0 (CH), 117.6 (CH, d, *J* = 21.0 Hz), 114.1 (CH, d, *J* = 21.0 Hz), 84.2 (C), 59.9 (C), 50.0 (CH), 43.1 (CH_2), 34.4 (2 C),

32.0 (CH_2), 30.4 (CH_3), 28.0 (CH_3). $^{19}F\{^1H\}$ NMR ($CDCl_3$, 282 MHz) δ_F -113.2. HRMS (ESI+): calcd m/z for $C_{38}H_{42}FN_2O_2$ [$(M+H-C_5H_9O_2)^+$]: 577.3225; Found: 577.3220. HPLC analysis (8:92 er): CHIRALPAK AD-H, hexane/*i*PrOH = 100:1, flow rate 1.00 mL/min, 20°C, UV 254 nm, t_{min}(dia$_{major}$) = 7.7 min, t_{major}(dia$_{major}$) = 9.3 min, t_{min}(dia$_{min}$) = 15.3 min, t_{major}(dia$_{min}$) = 16.8 min.

IV. NMR spectra

IV. 1. 1. NMR spectra of C5-monosubstituted Meldrum's acid derivatives

5-(3-fluorobenzyl)-2,2-dimethyl-1,3-dioxane-4,6-dione (**1a**)

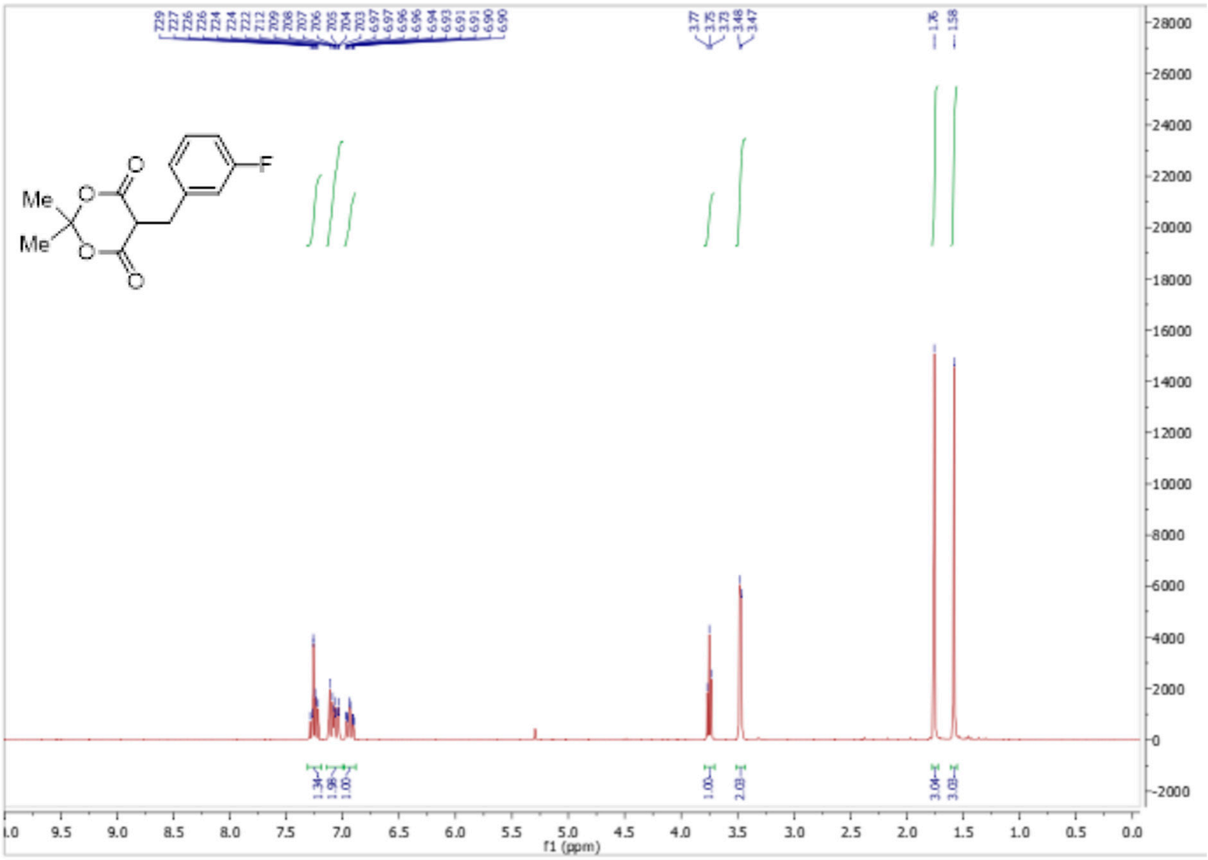

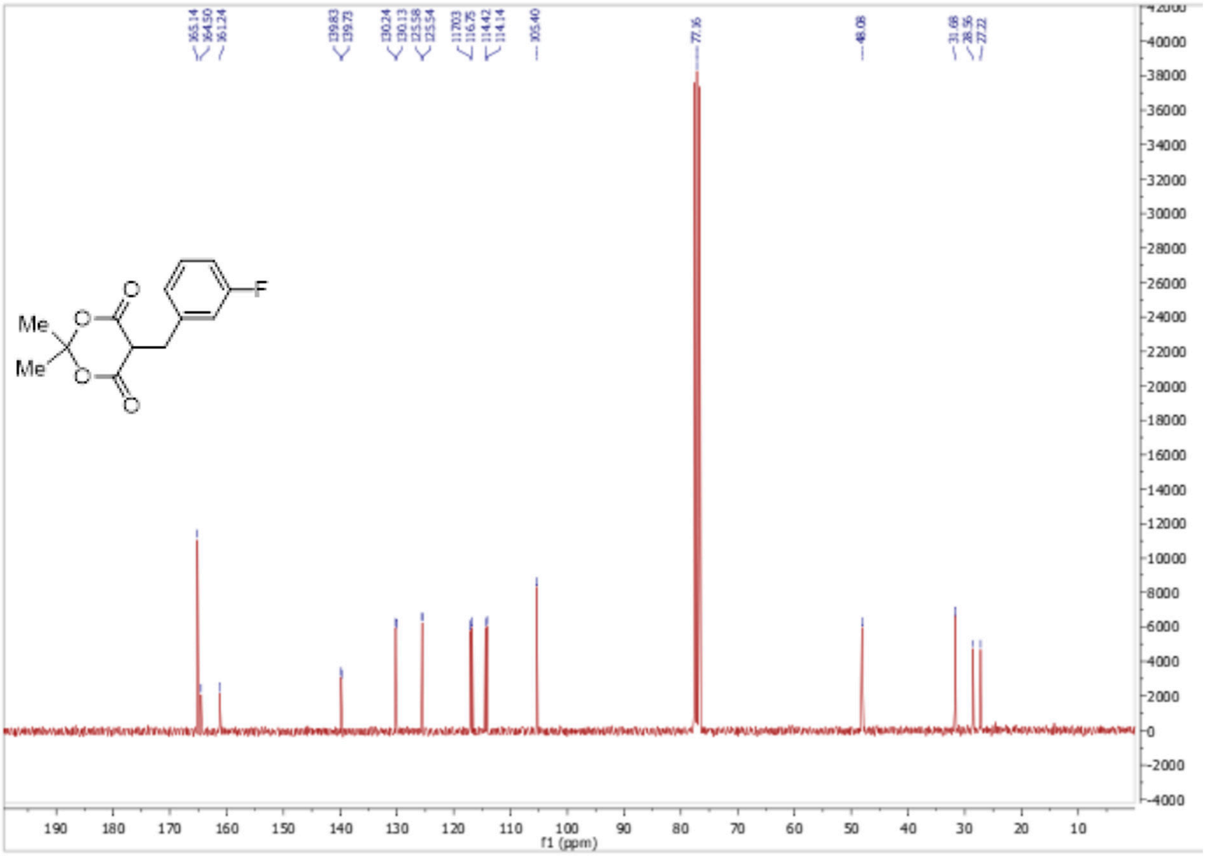

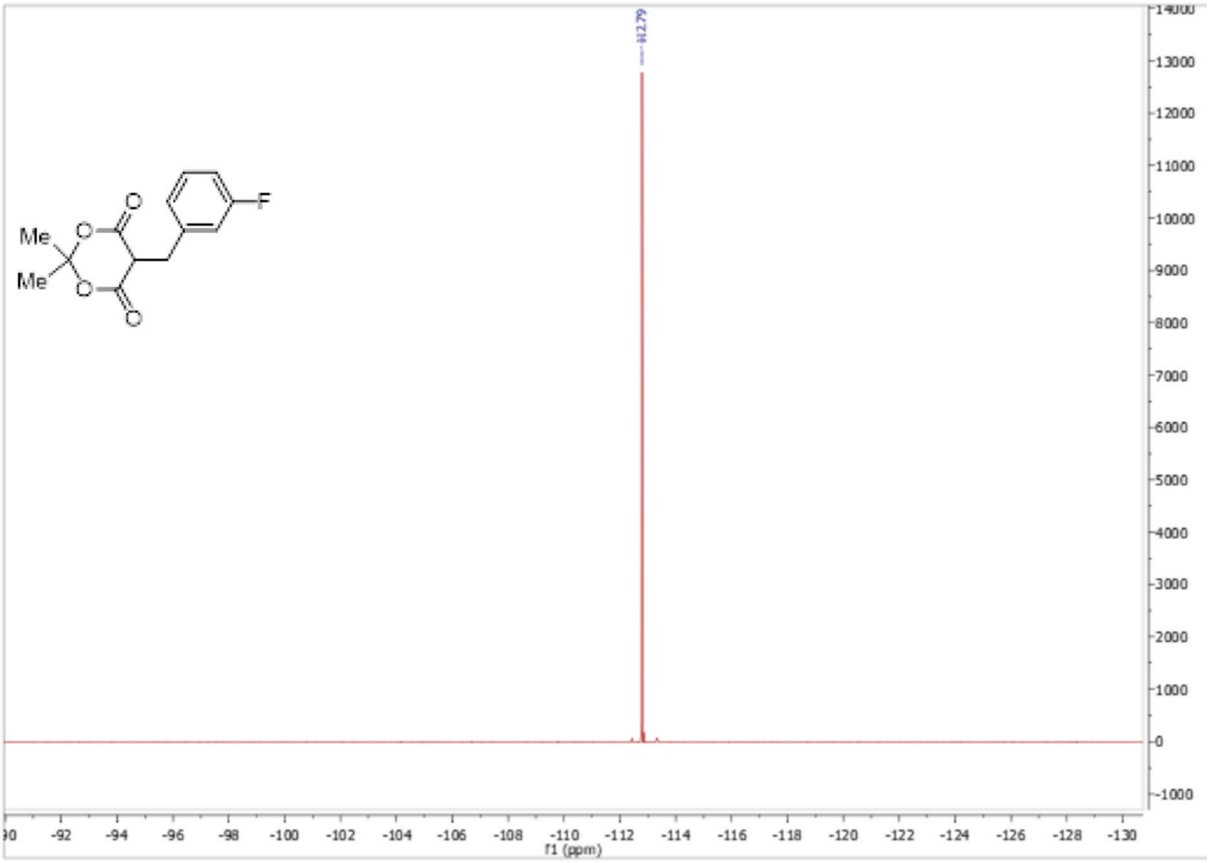

5-(3-fluorobenzyl)-2,2-dimethyl-1,3-dioxane-4,6-dione (1b)

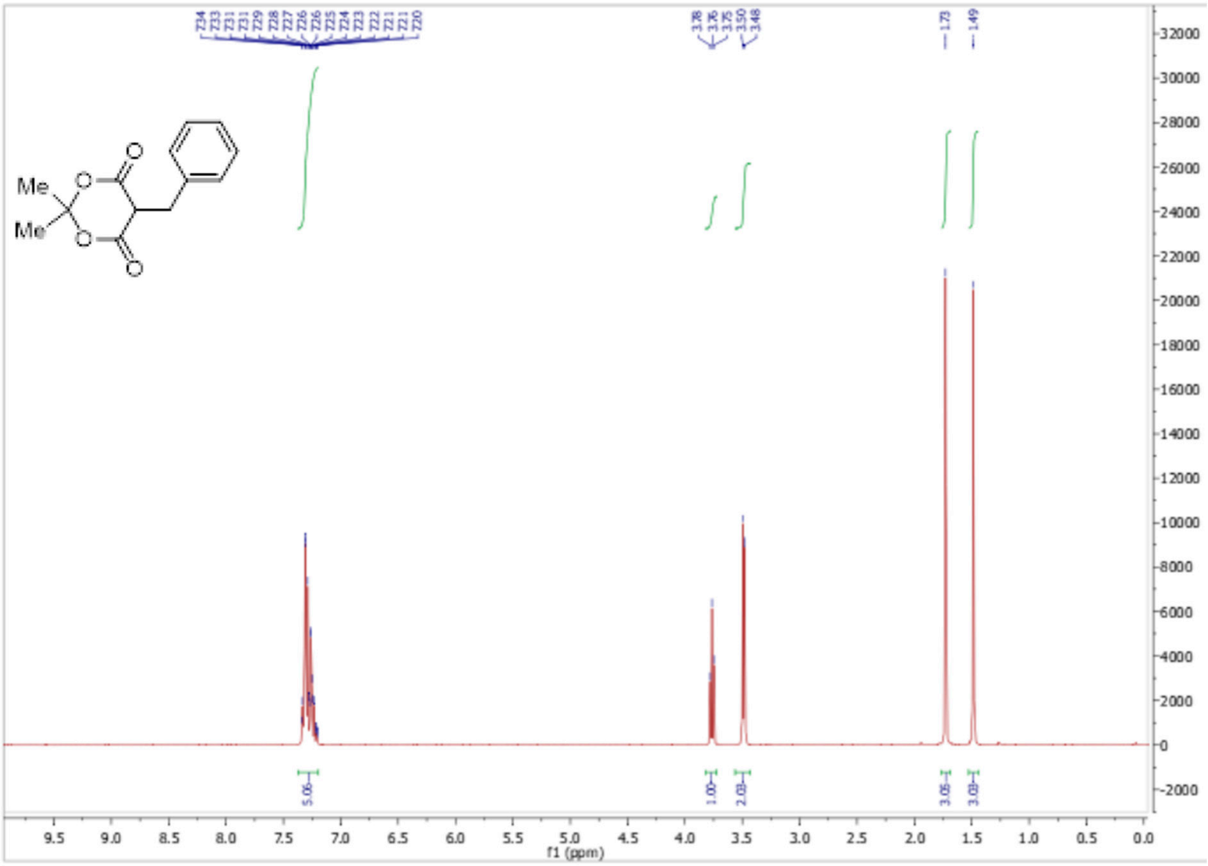

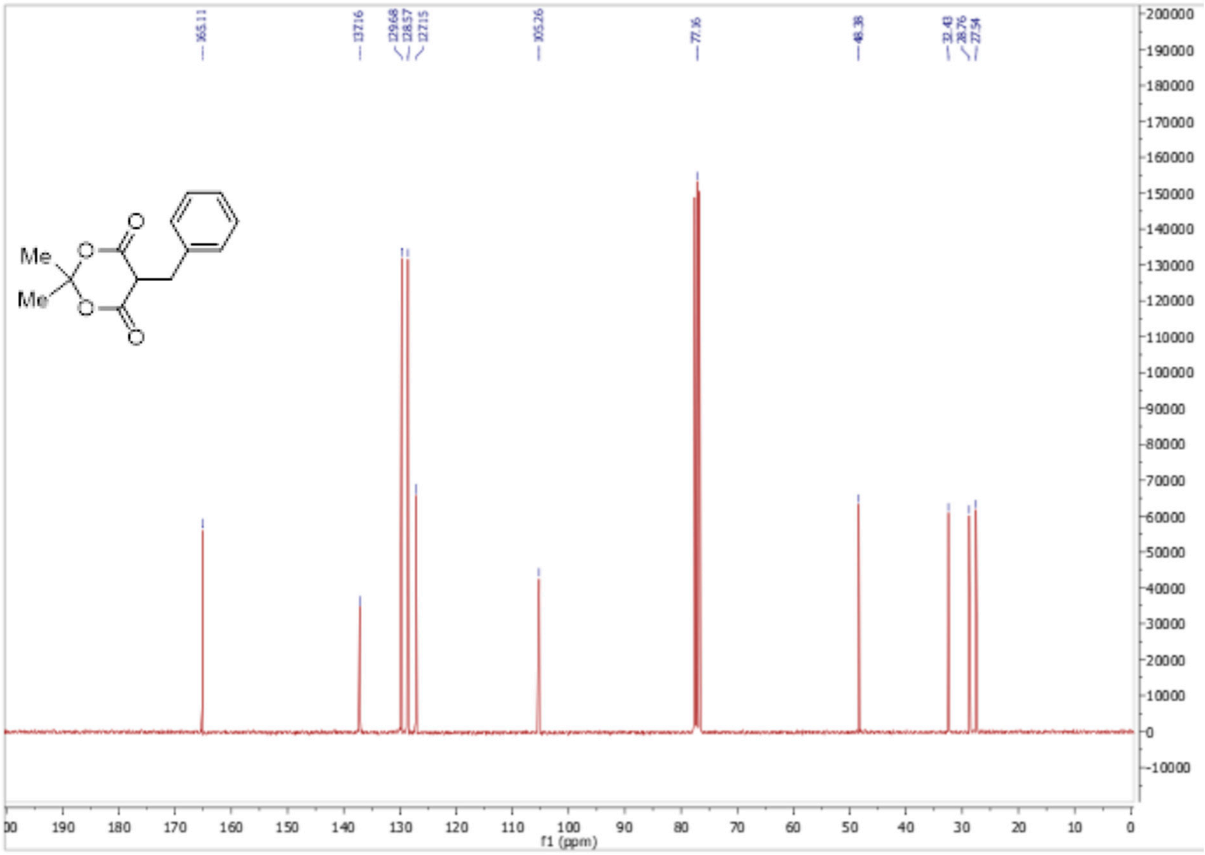

2,2-dimethyl-5-(naphthalen-1-ylmethyl)-1,3-dioxane-4,6-dione (1c)

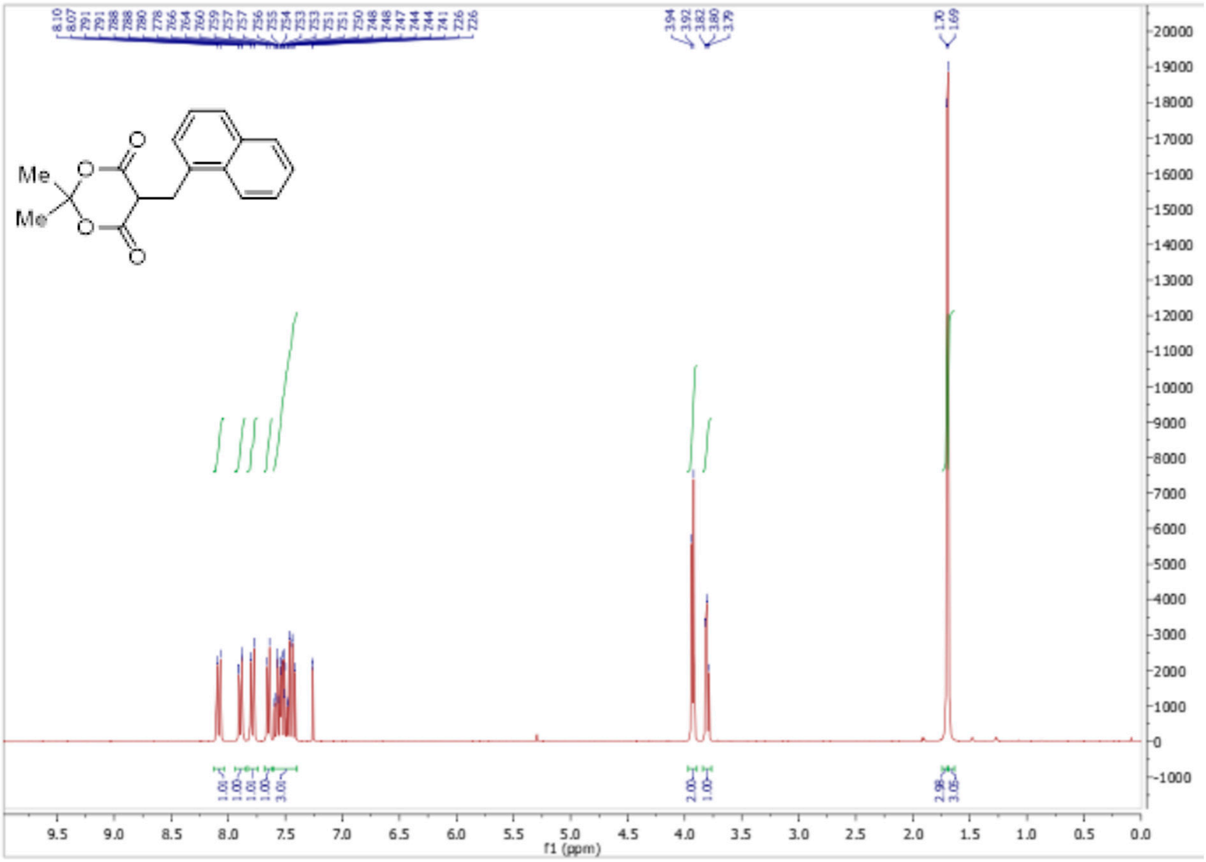

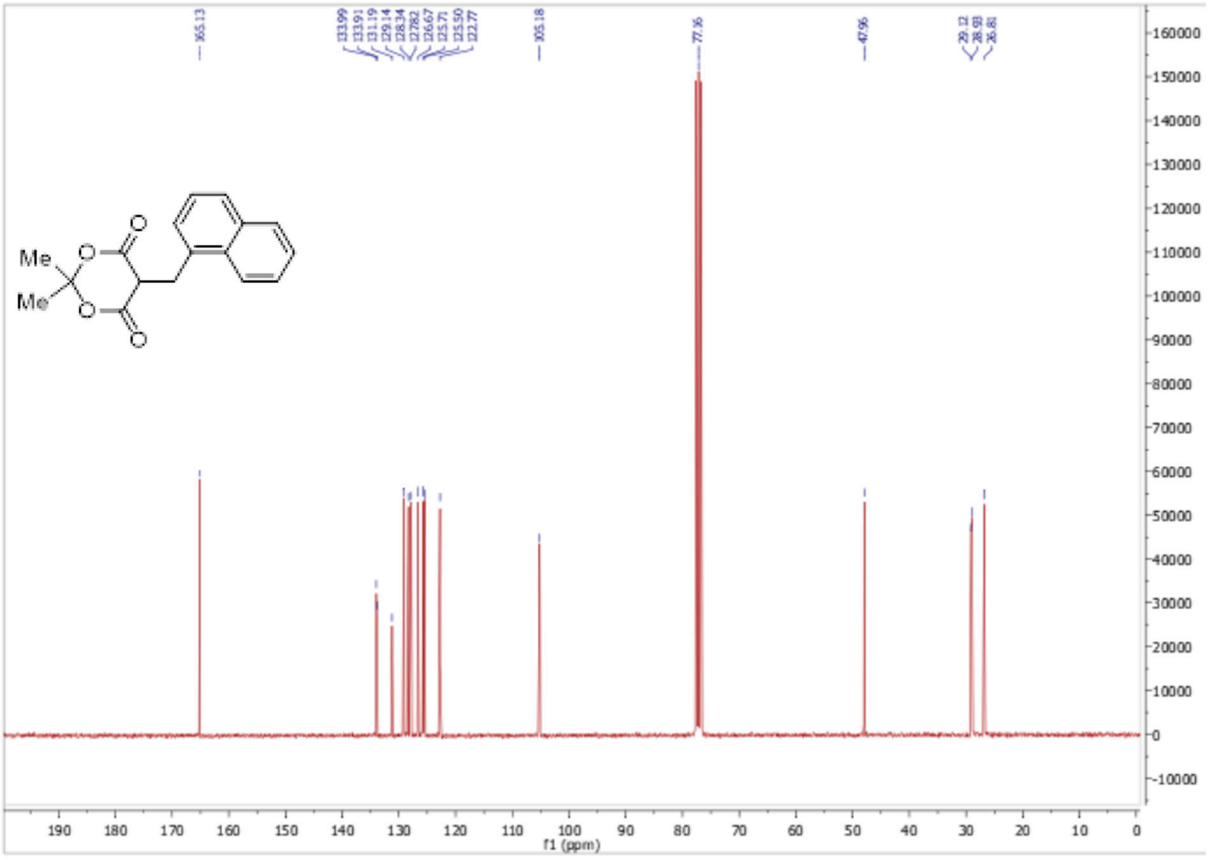

5-(4-fluorobenzyl)-2,2-dimethyl-1,3-dioxane-4,6-dione (1d)

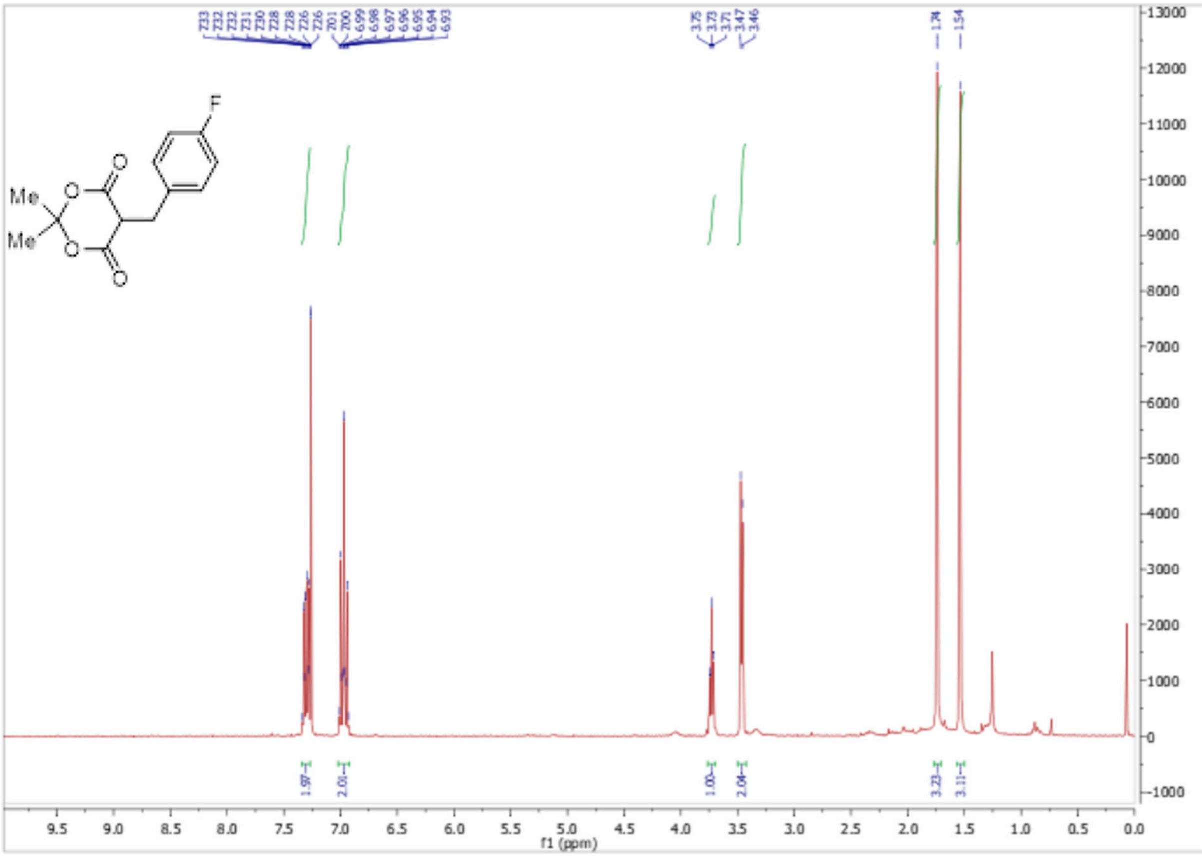

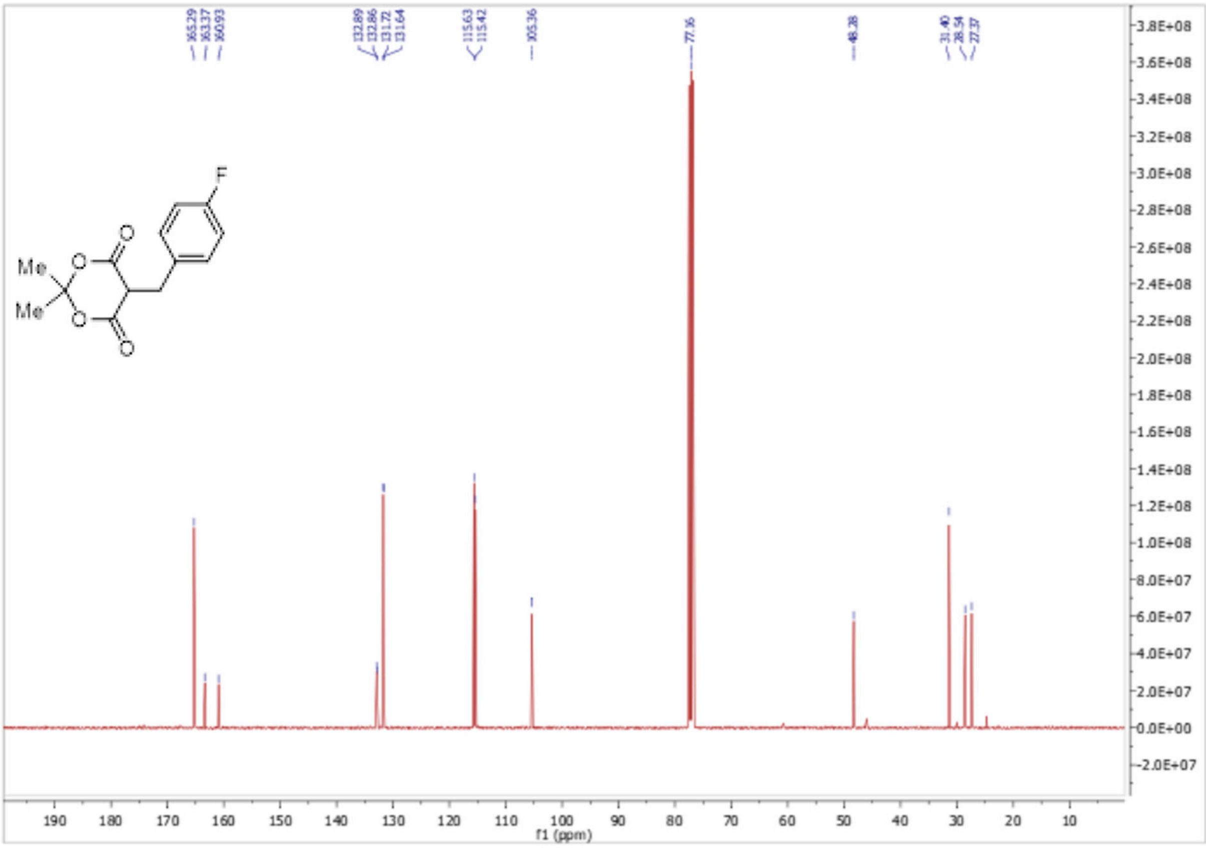

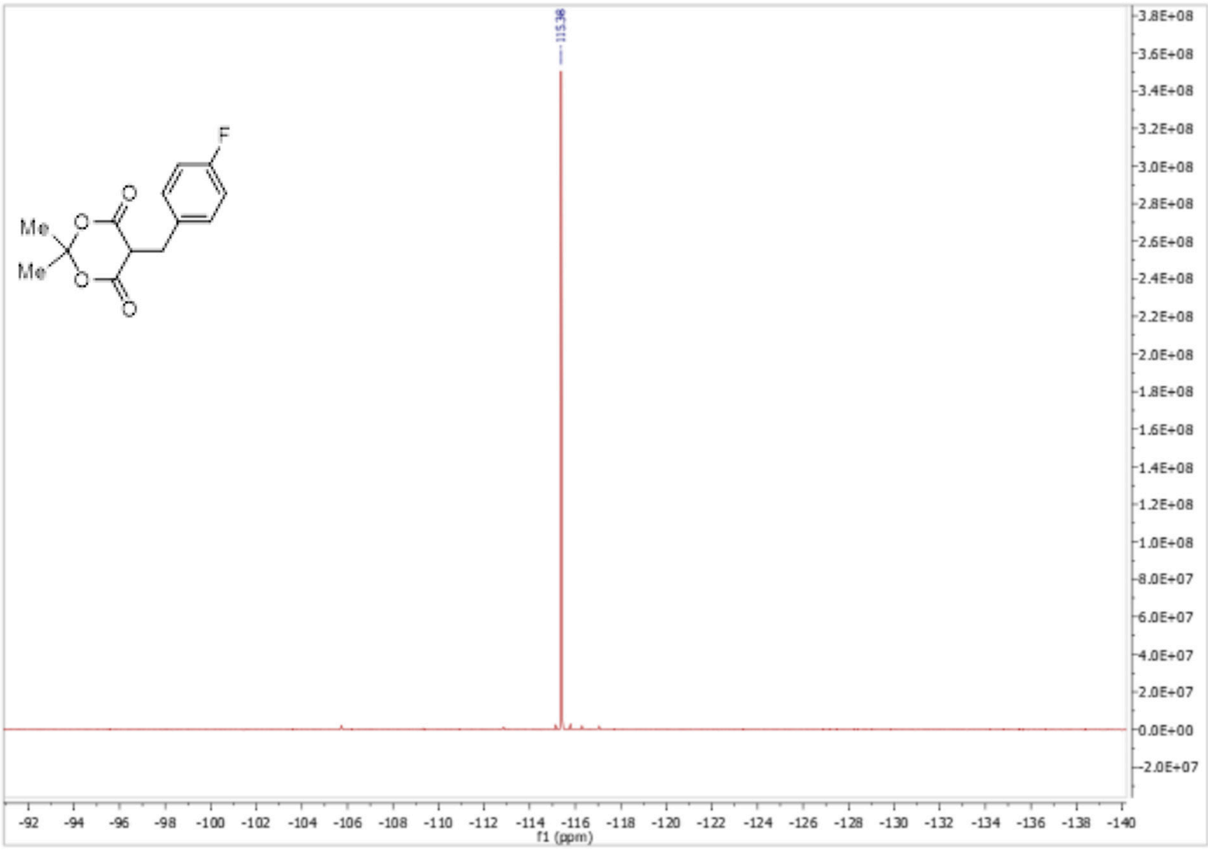
-115.36
F
Me
Me
O
O
O
O
f1 (ppm)

5-(4-bromobenzyl)-2,2-dimethyl-1,3-dioxane-4,6-dione (**1e**)

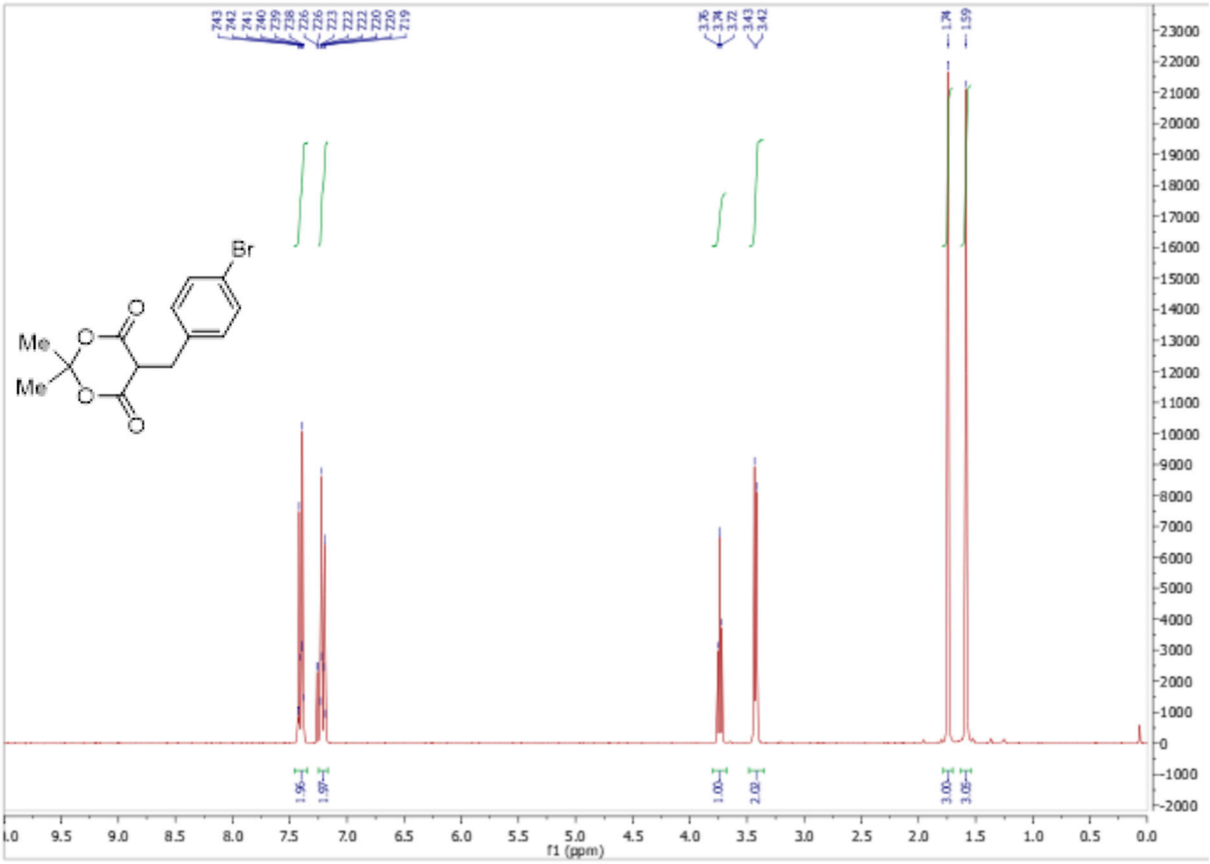

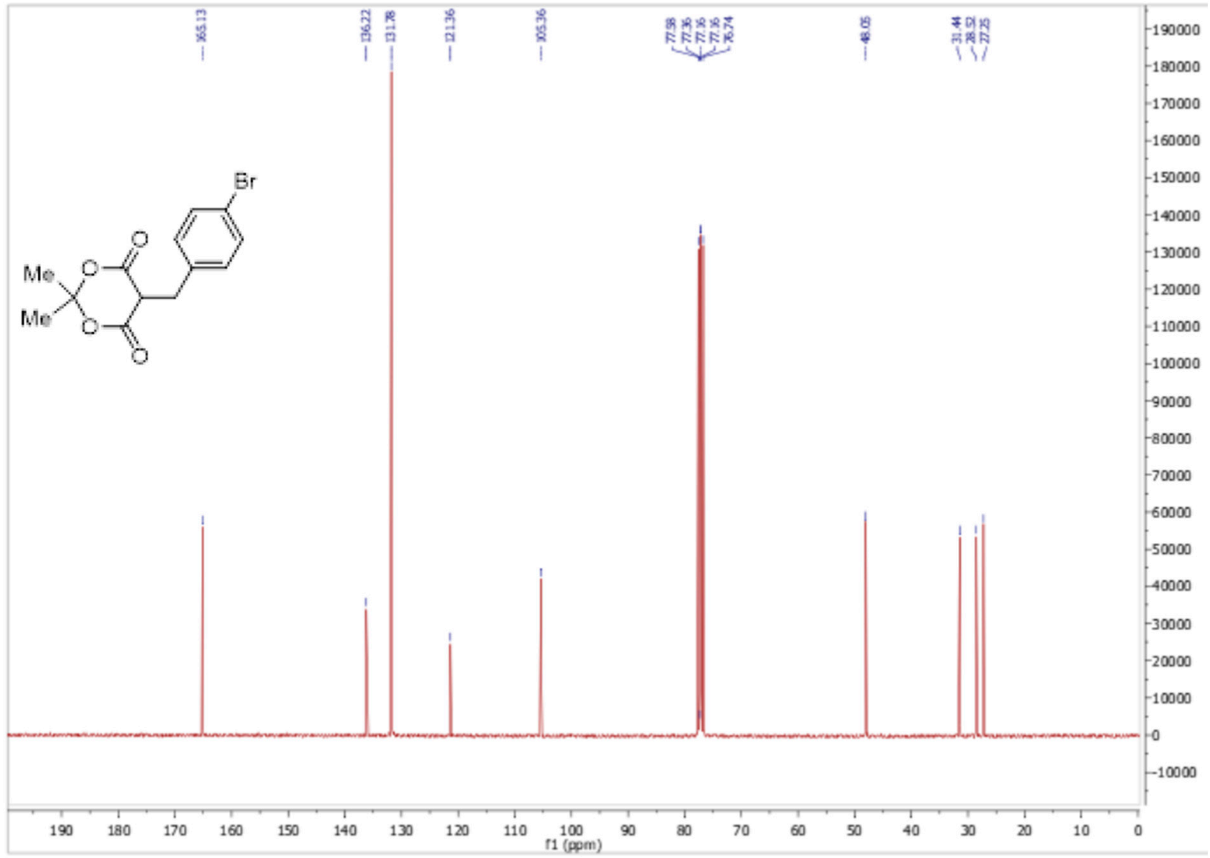

5-(4-chlorobenzyl)-2,2-dimethyl-1,3-dioxane-4,6-dione (1f)

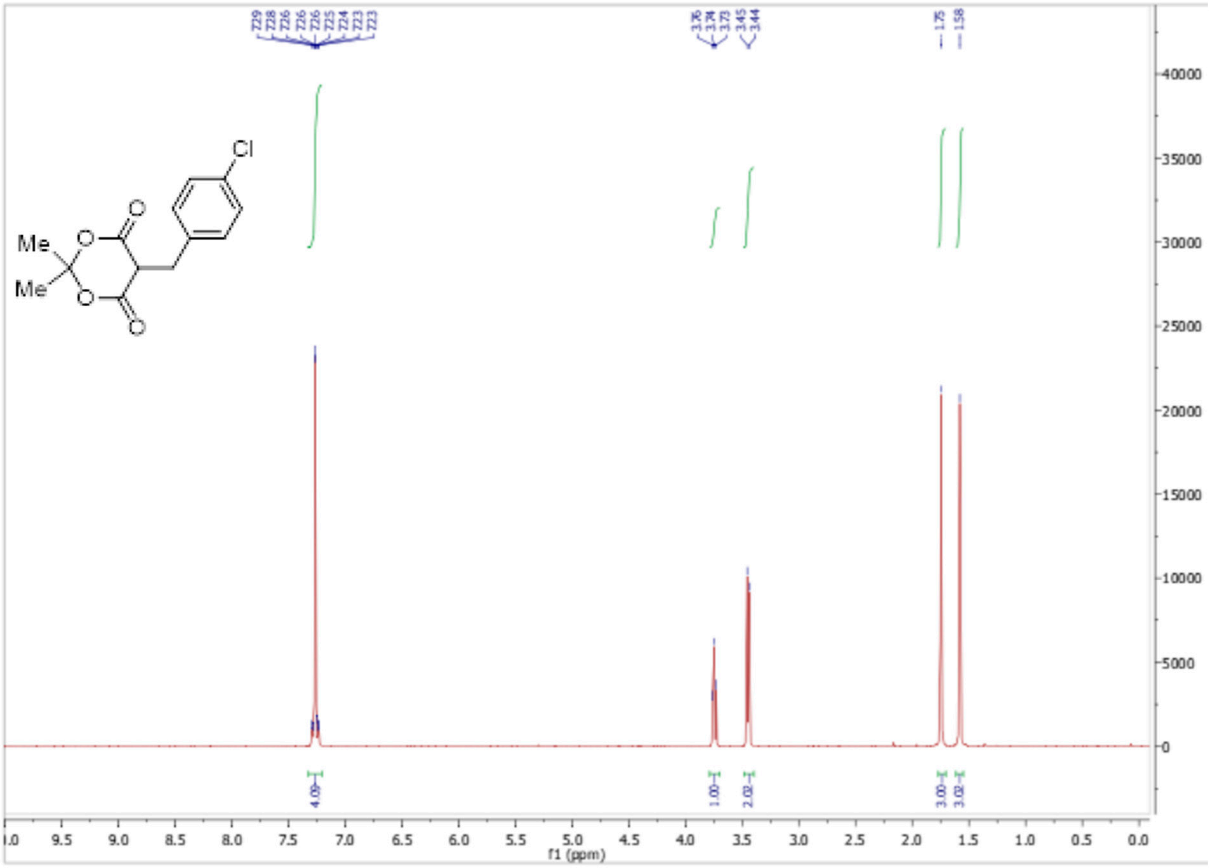

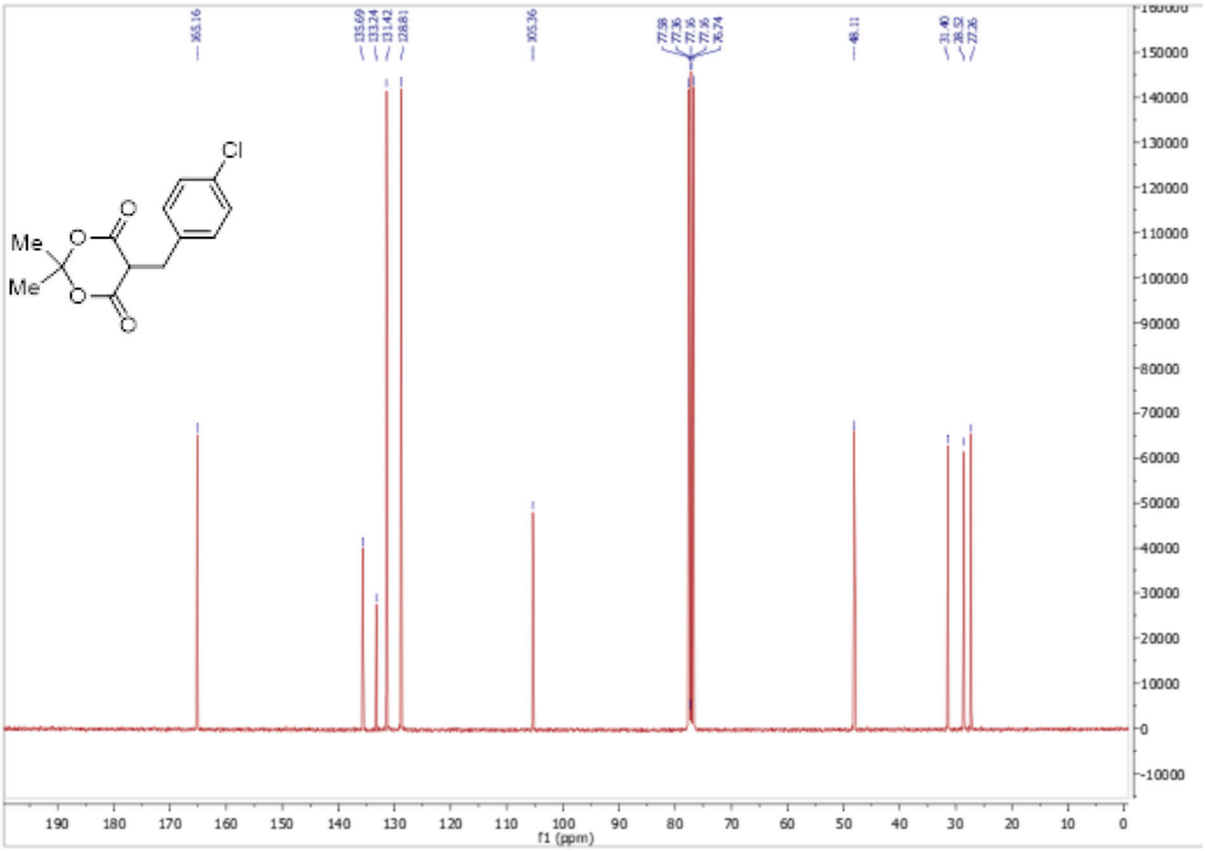

2-((2,2-dimethyl-4,6-dioxo-1,3-dioxan-5-yl)methyl)benzonitrile (1g)

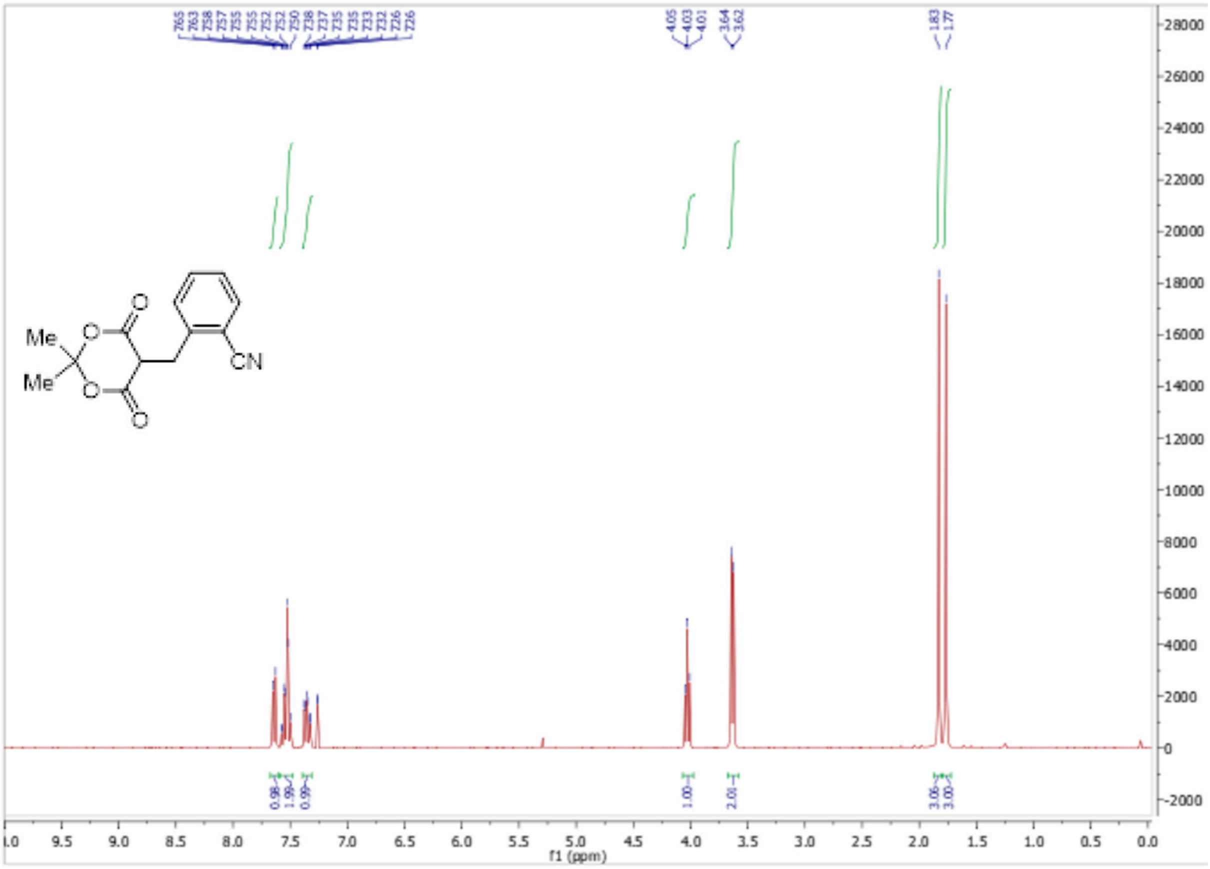

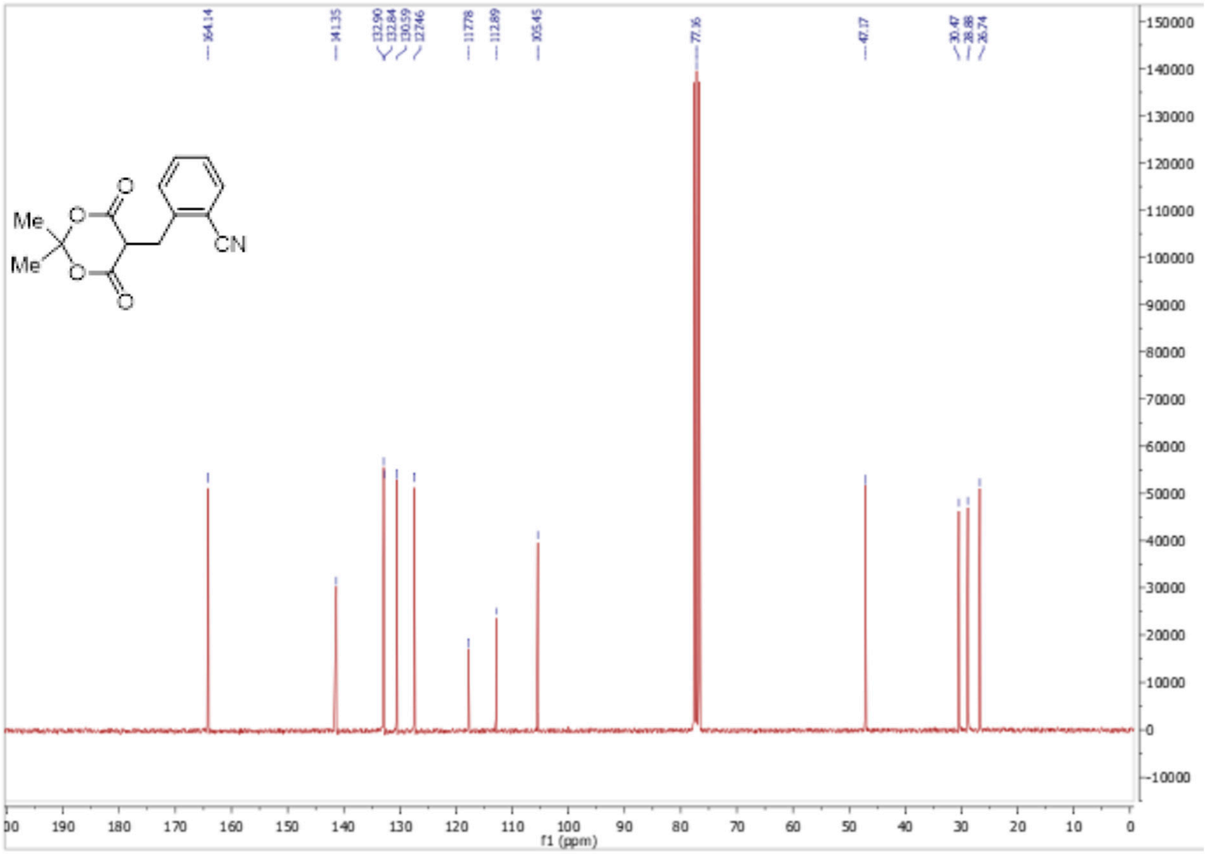

5-(2-methoxybenzyl)-2,2-dimethyl-1,3-dioxane-4,6-dione (1h)

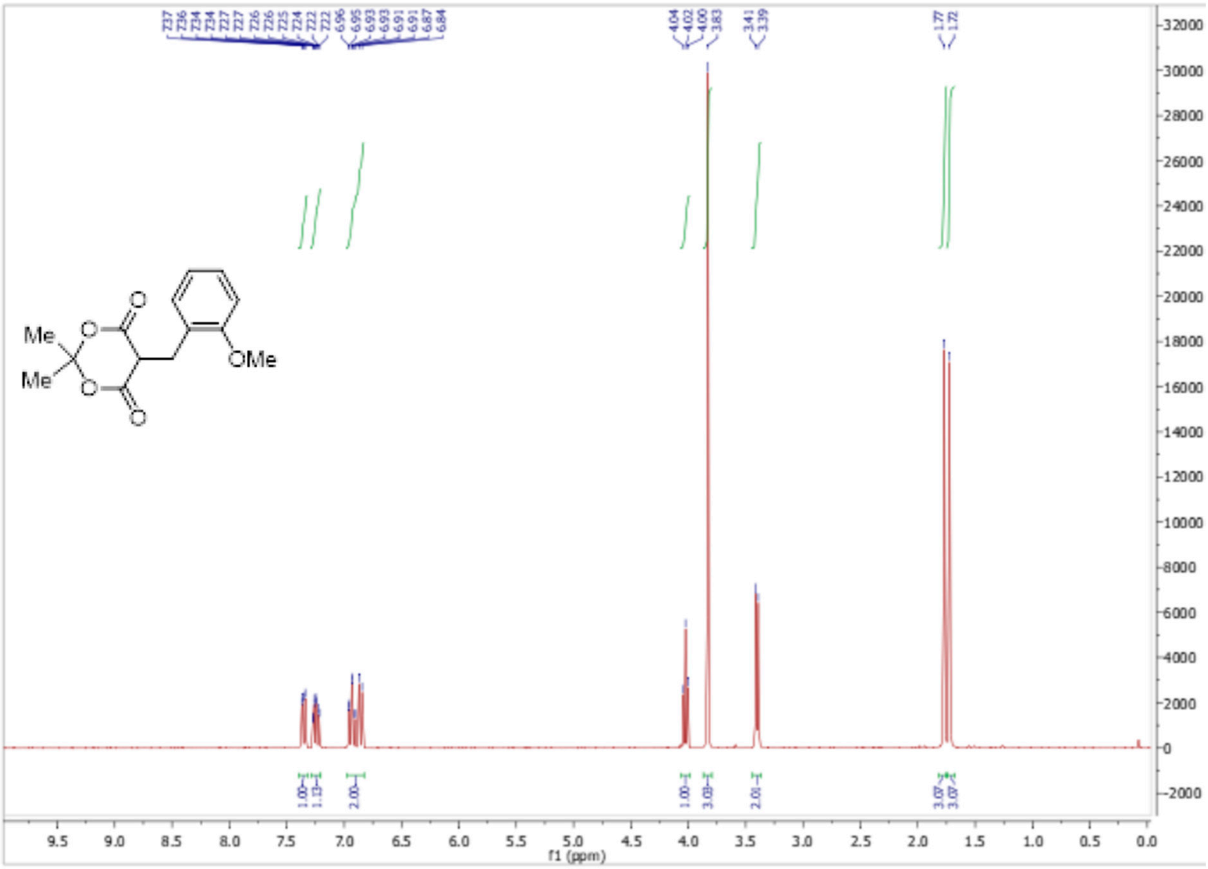

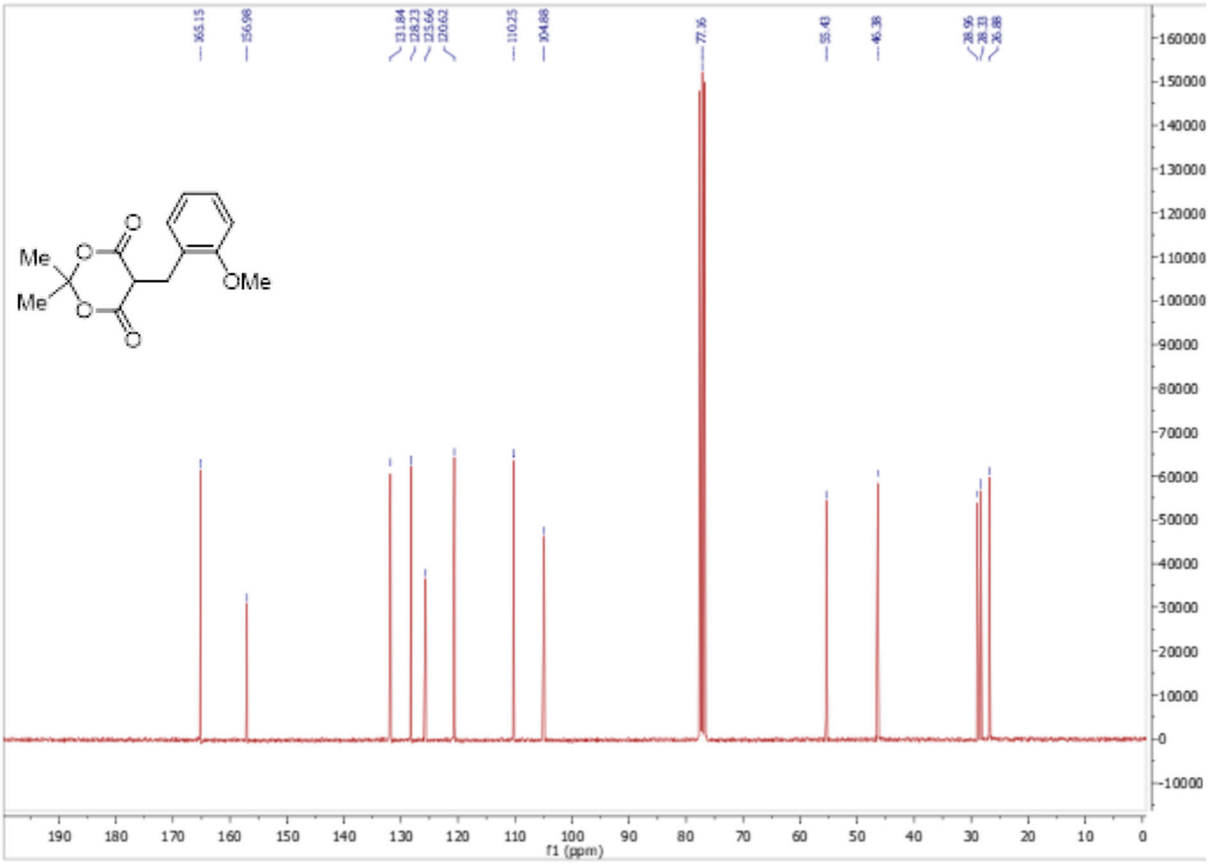

2,2-dimethyl-5-(4-methylbenzyl)-1,3-dioxane-4,6-dione (**1i**)

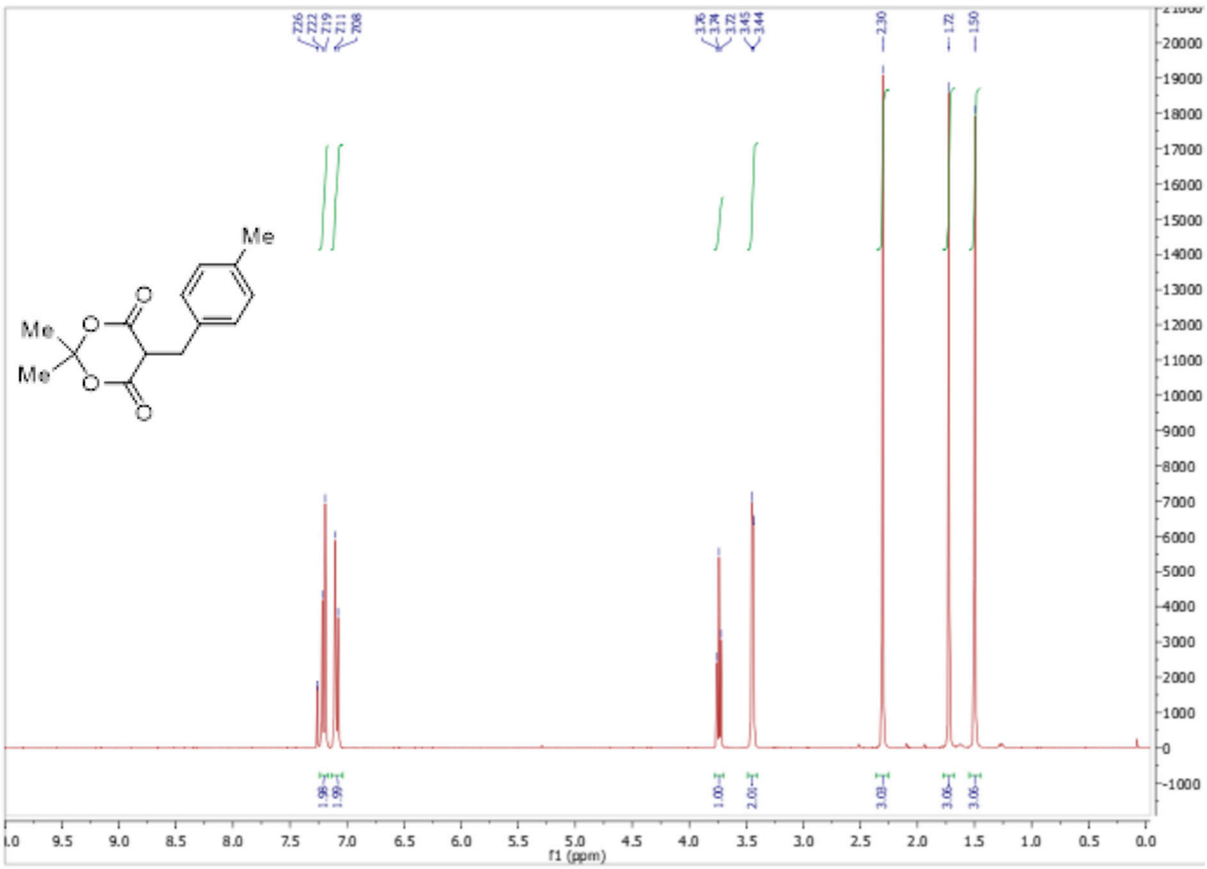

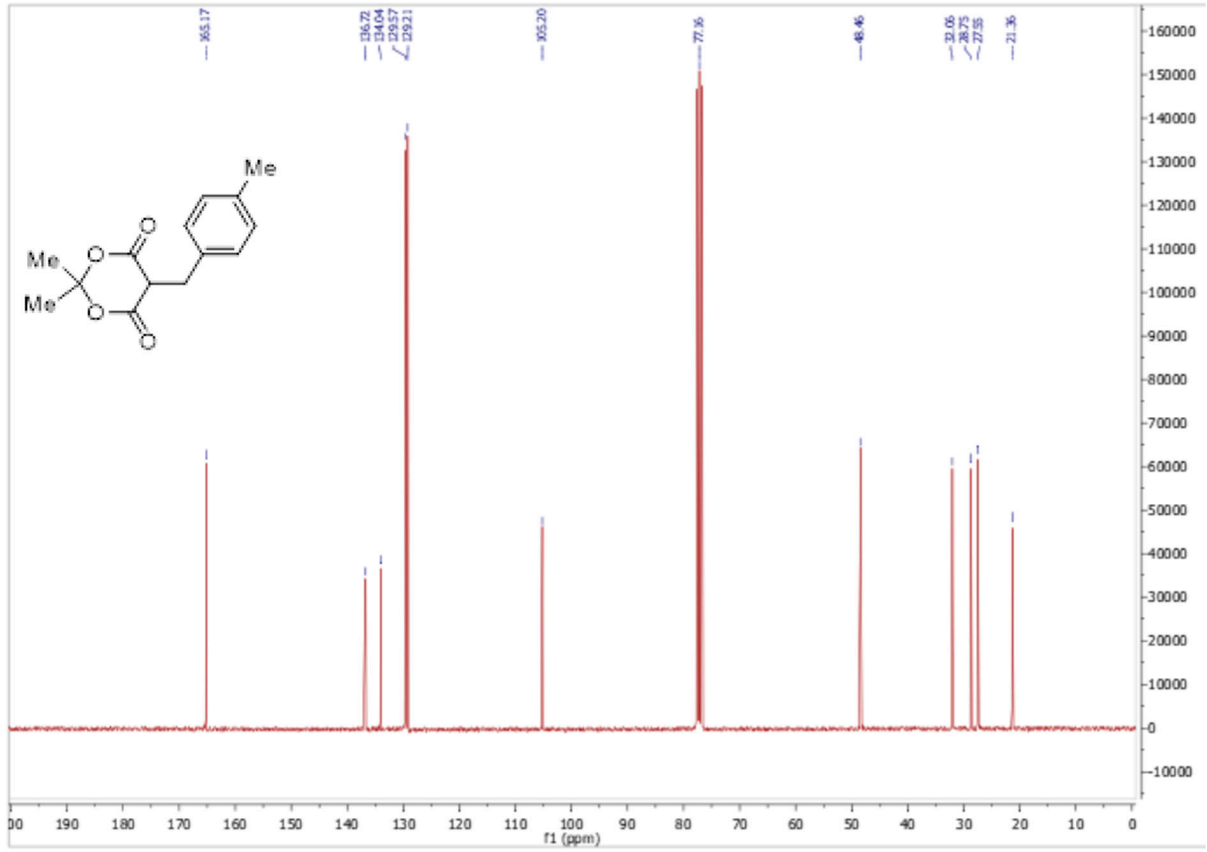

5-(2,5-dimethylbenzyl)-2,2-dimethyl-1,3-dioxane-4,6-dione (1j)

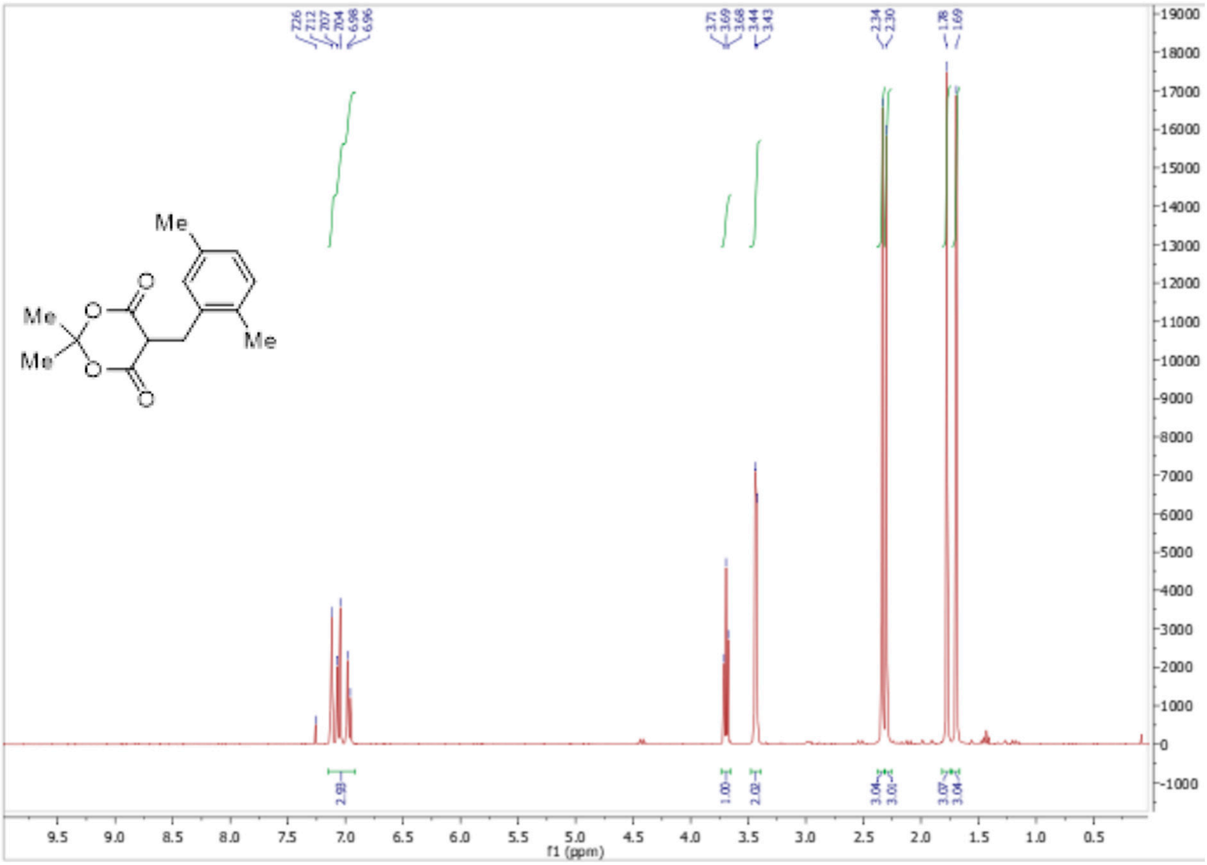

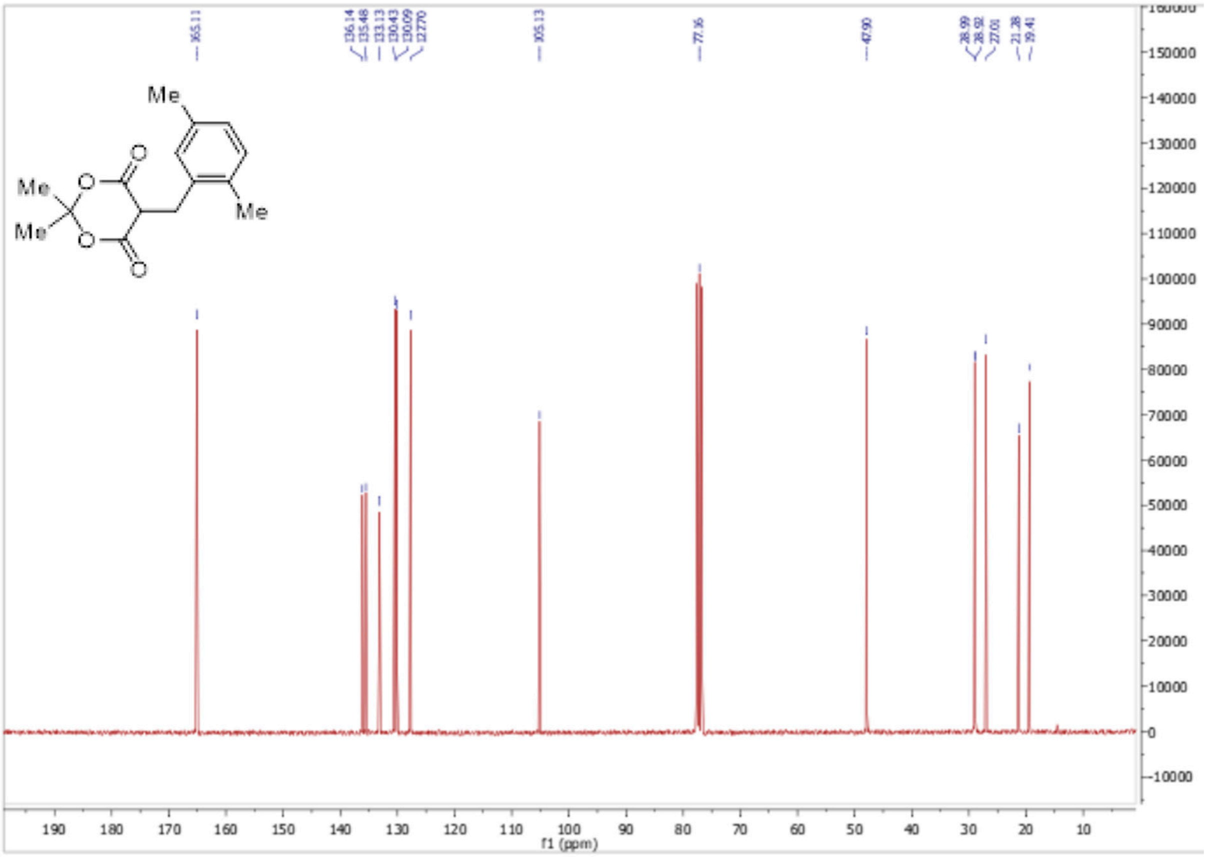

2,2-dimethyl-5-(thiophen-2-ylmethyl)-1,3-dioxane-4,6-dione (1k)

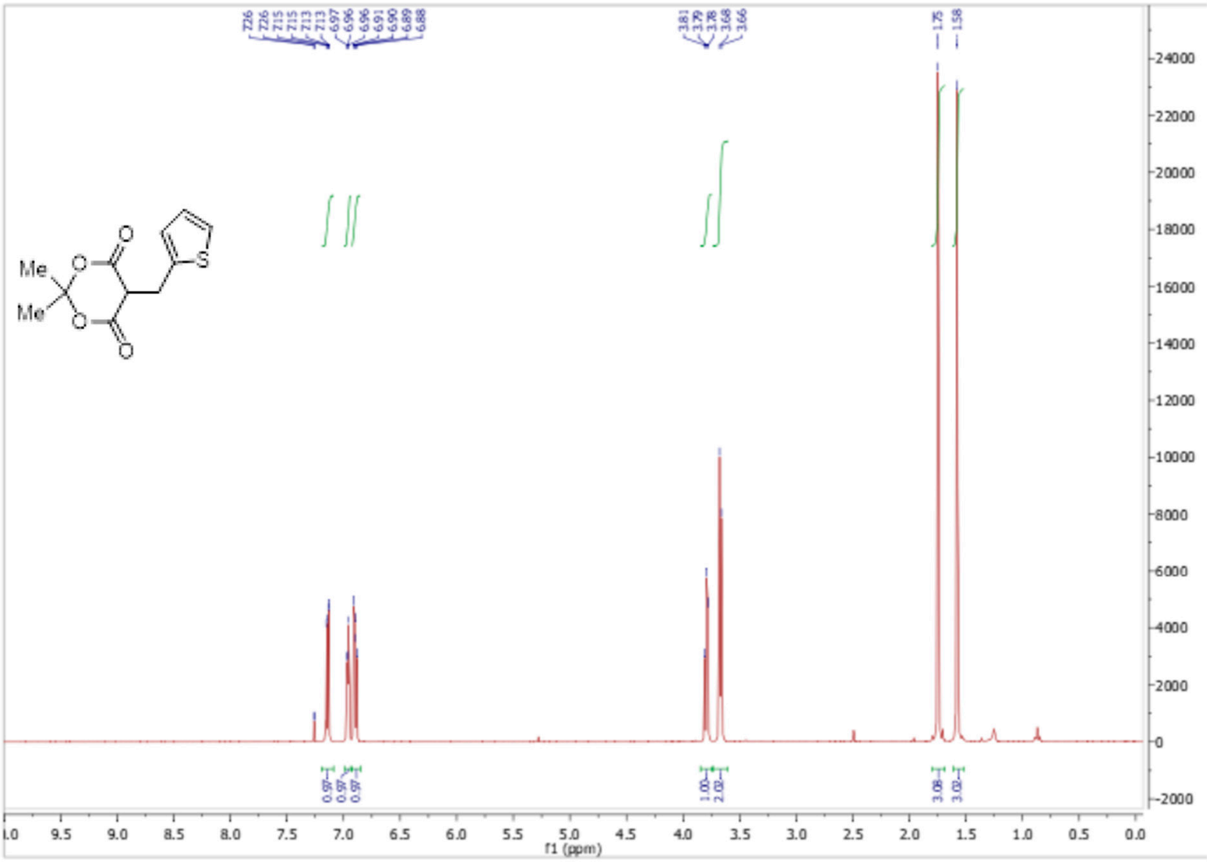

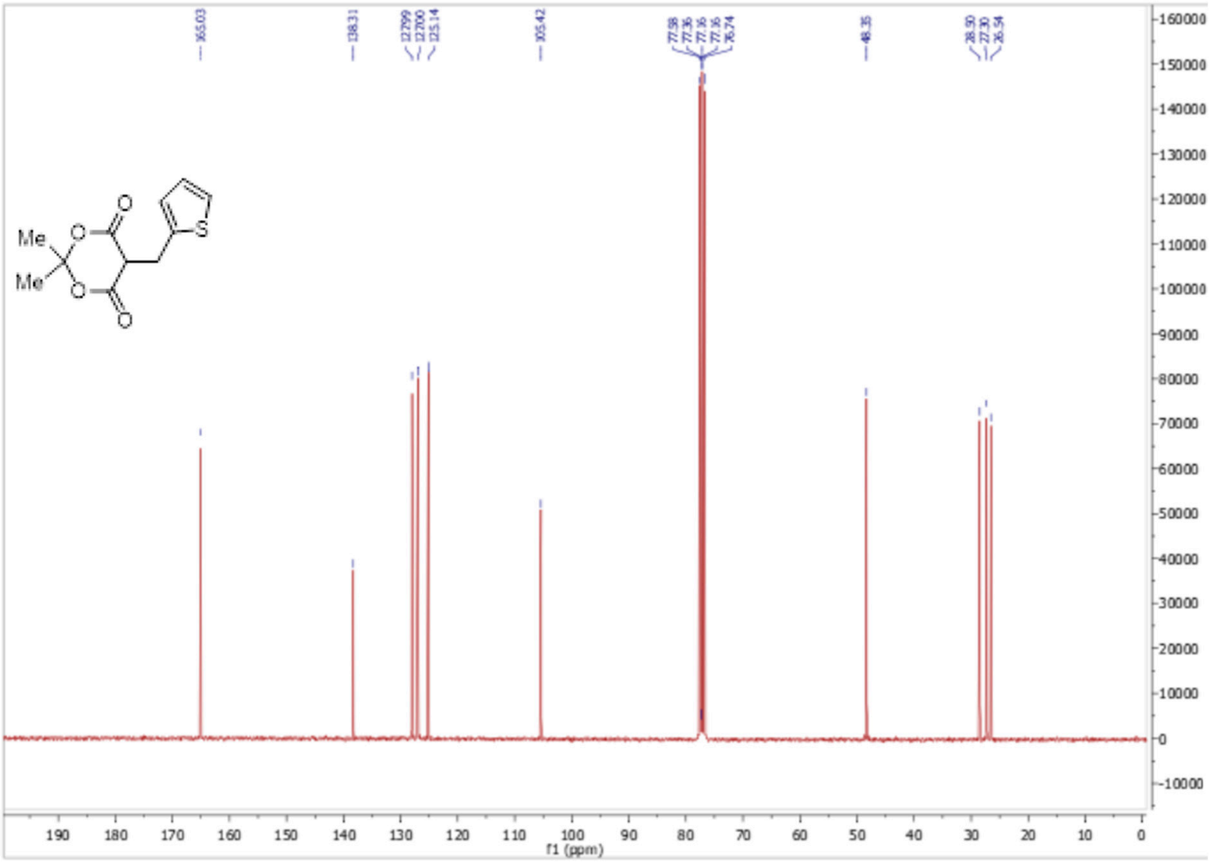

5-isobutyl-2,2-dimethyl-1,3-dioxane-4,6-dione (**1l**)

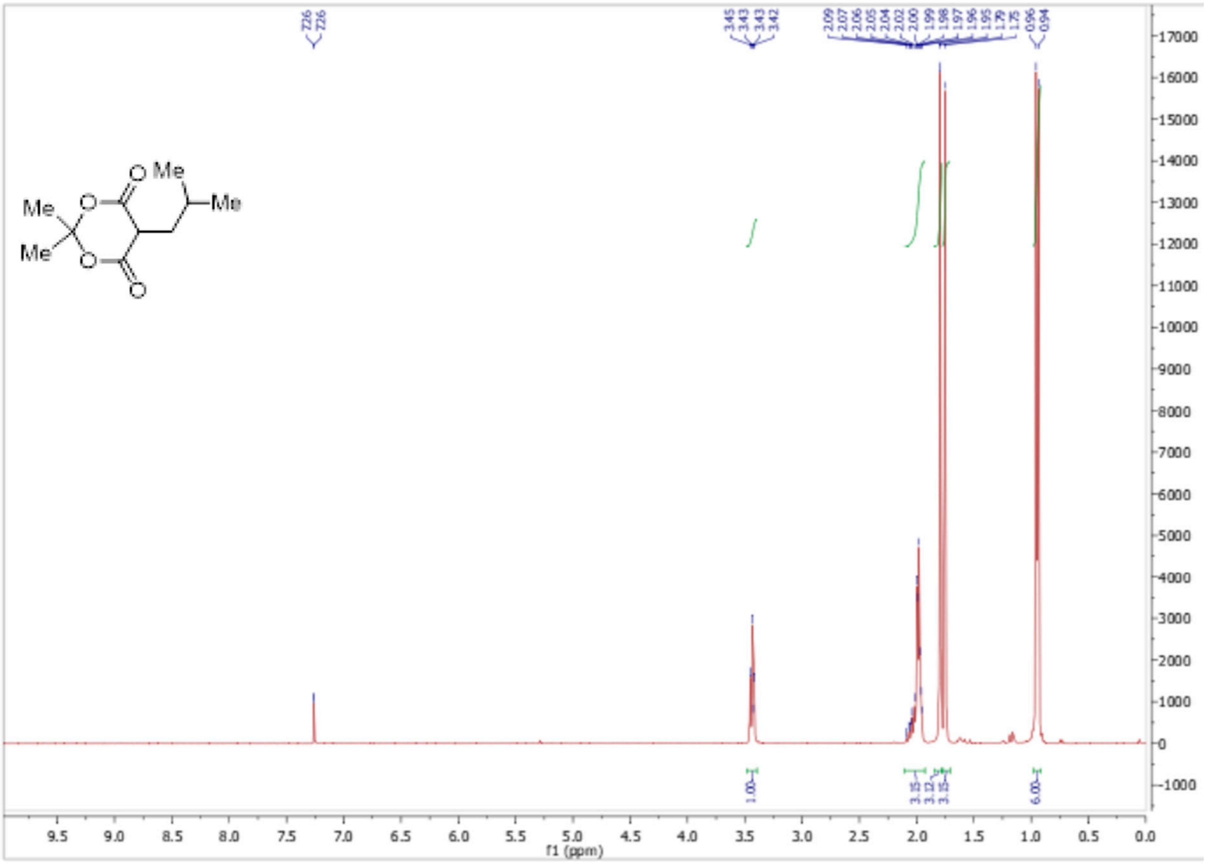

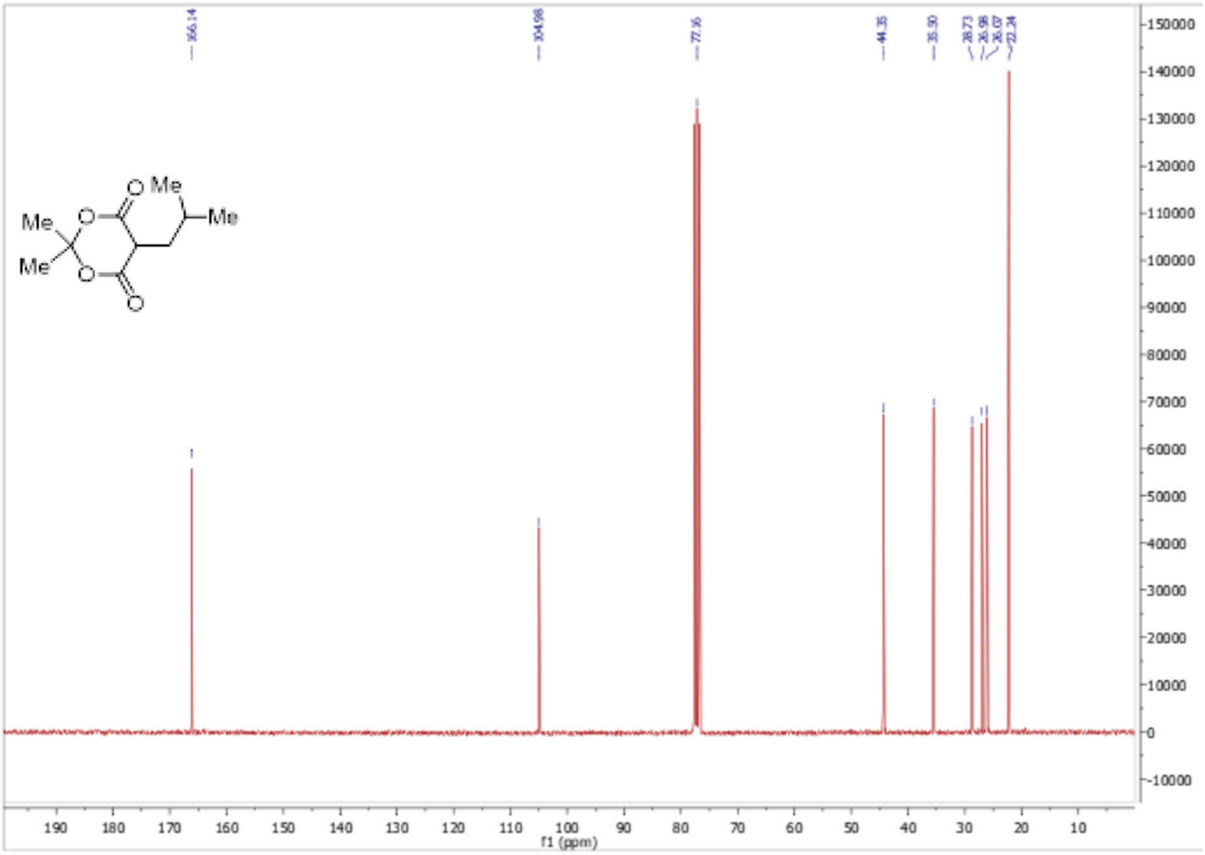

2,2-dimethyl-5-(3-phenylpropyl)-1,3-dioxane-4,6-dione (**1m**)

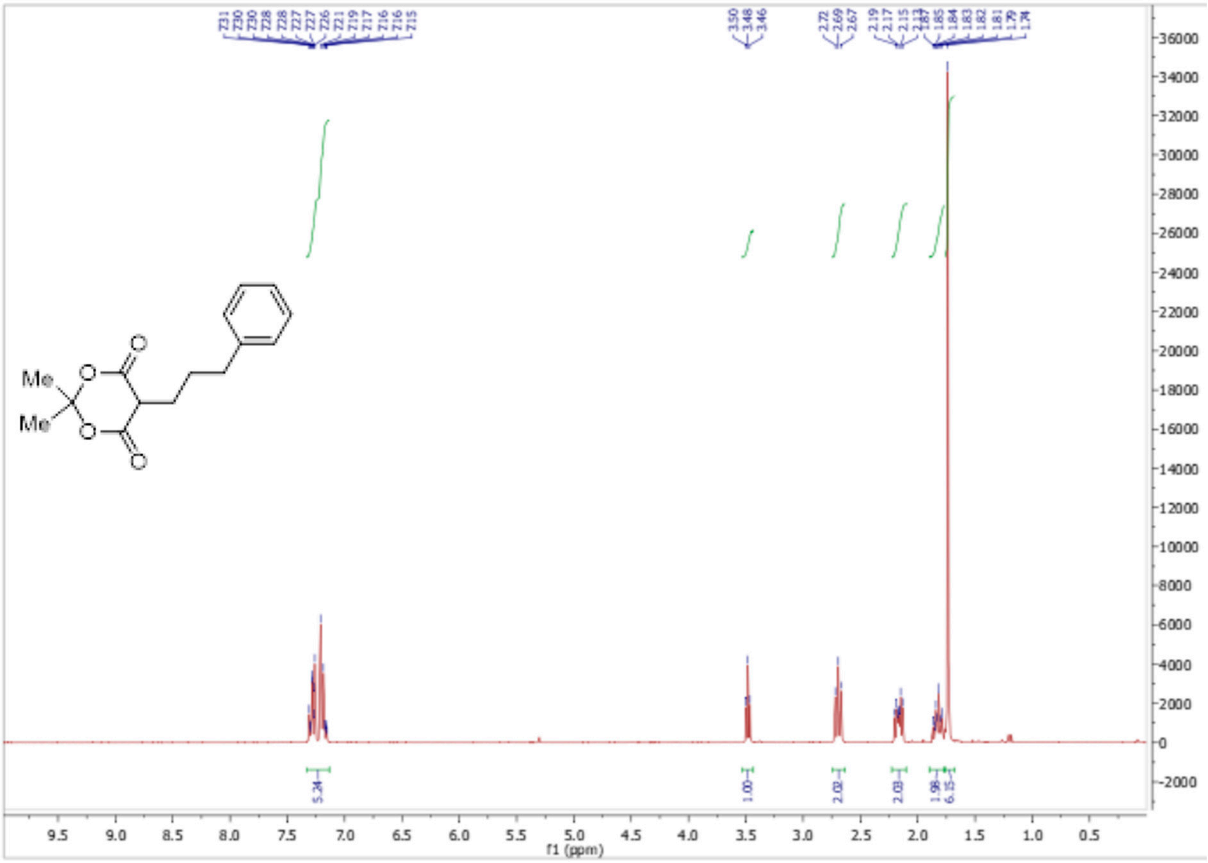

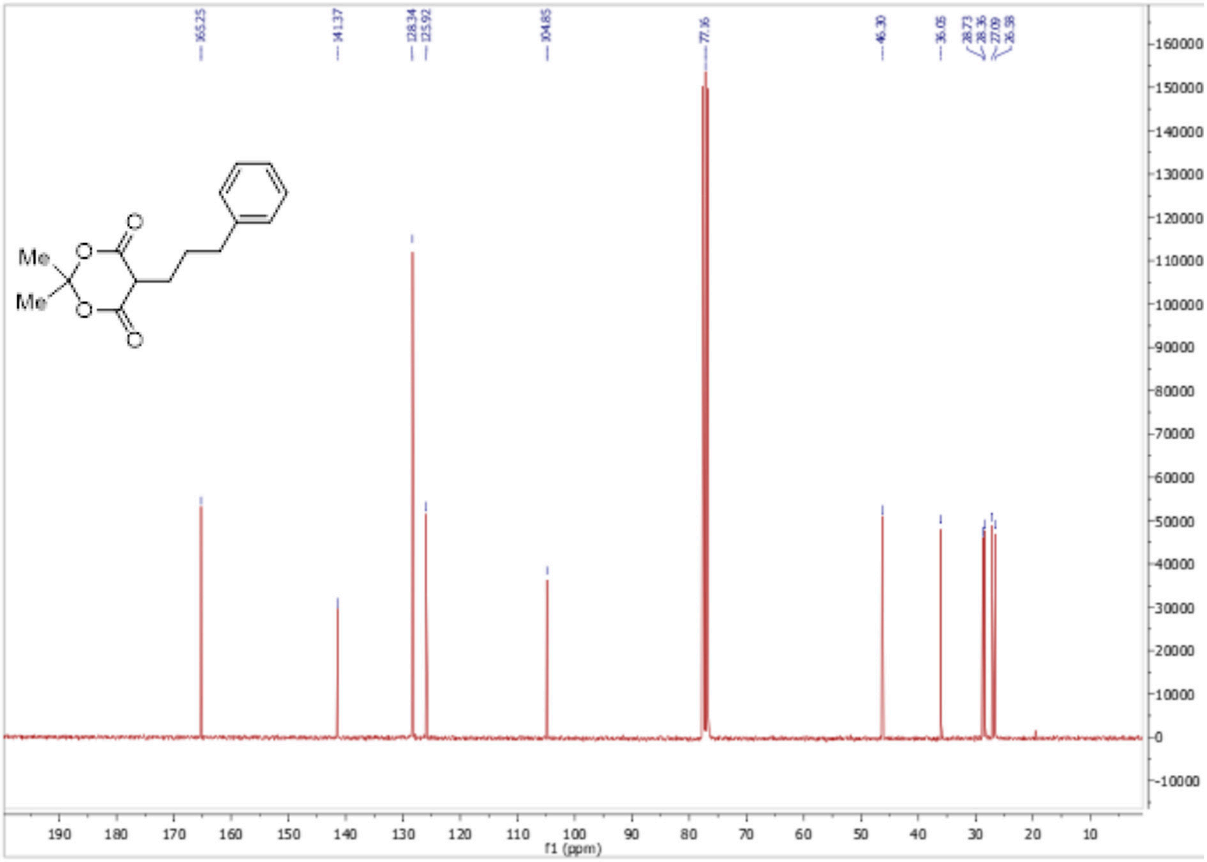

2,2-dimethyl-5-phenyl-1,3-dioxane-4,6-dione (1o)

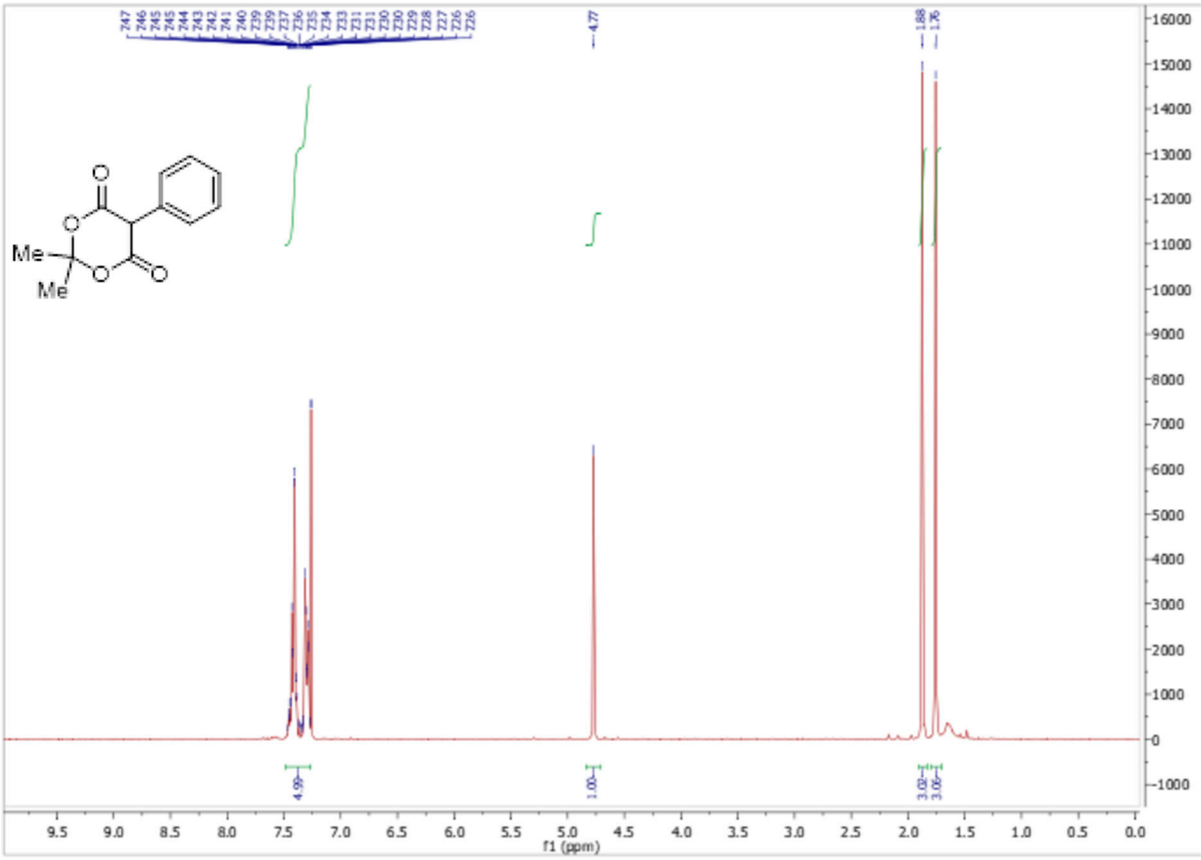

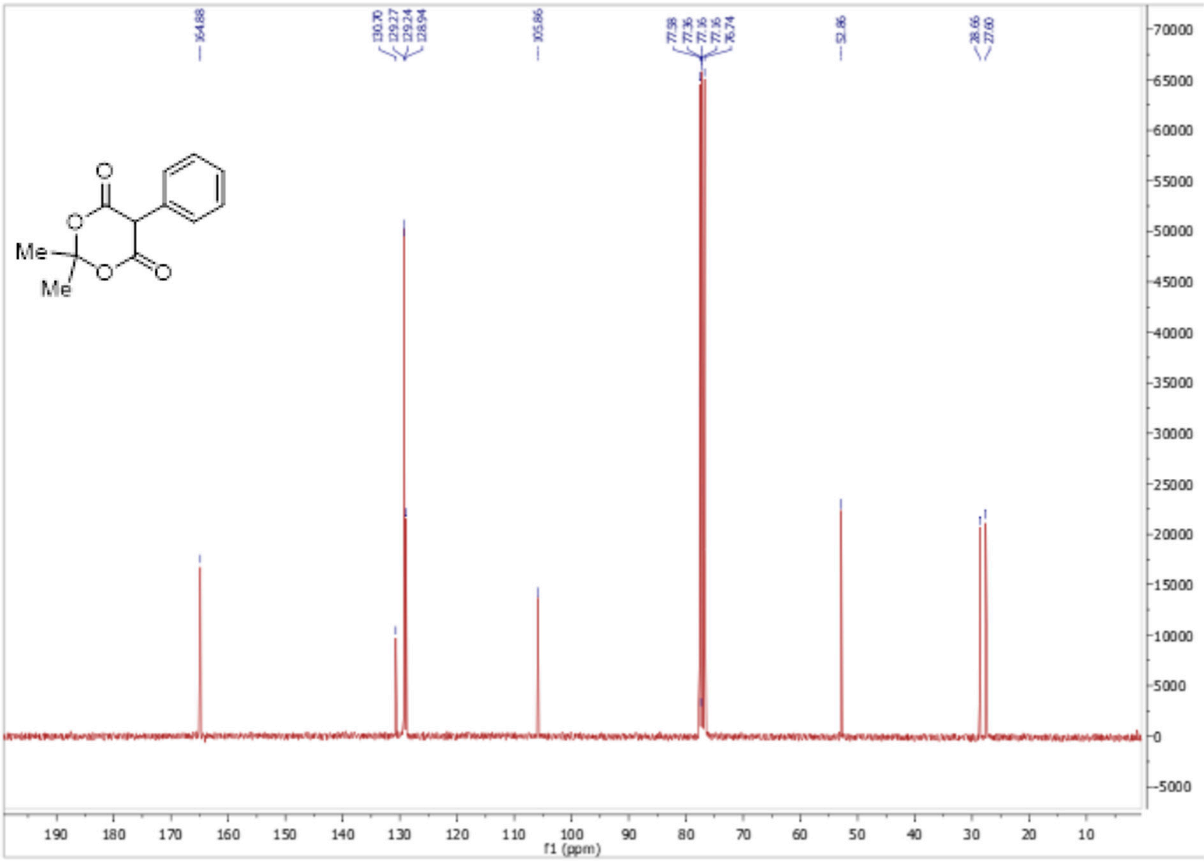

IV. 1. 2. NMR spectra of C5-disubstituted Meldrum's acid derivatives

5-(3-fluorobenzyl)-2,2-dimethyl-5-(2-oxo-2-phenylethyl)-1,3-dioxane-4,6-dione (**2a**)

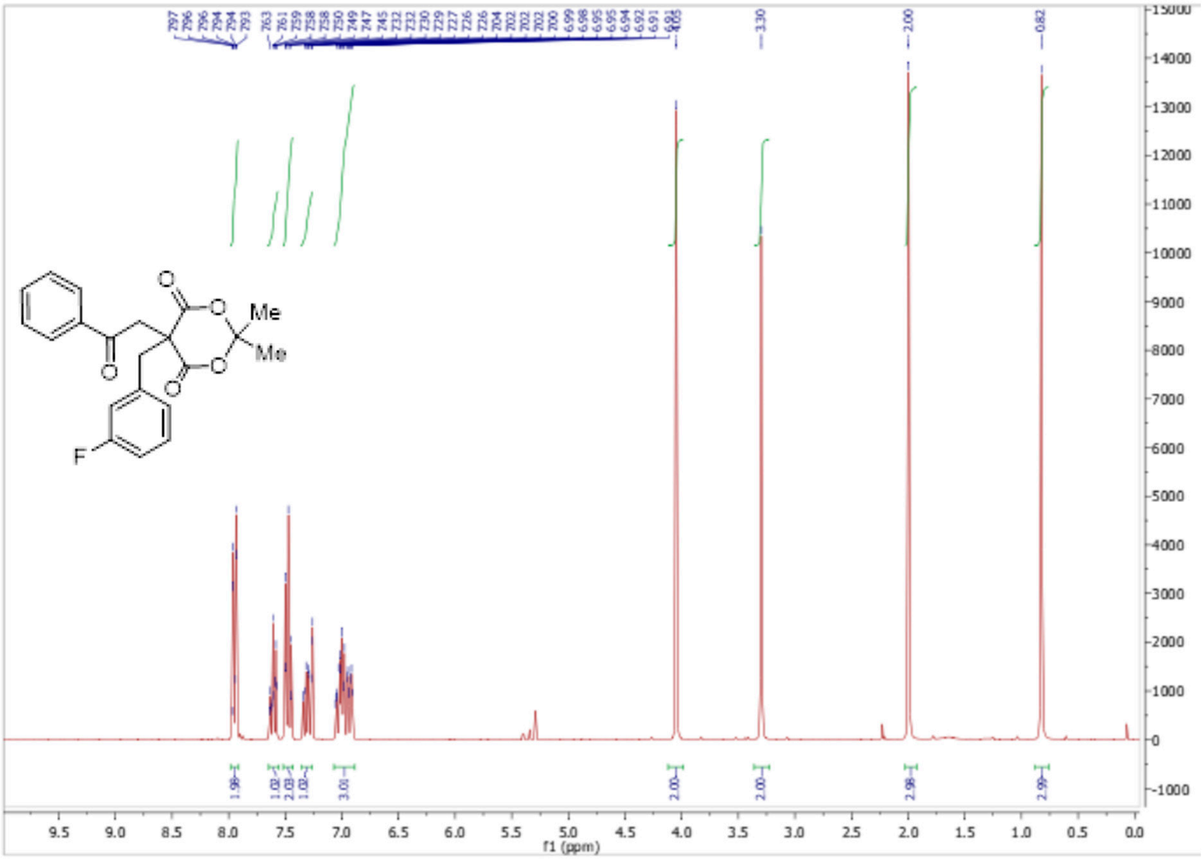

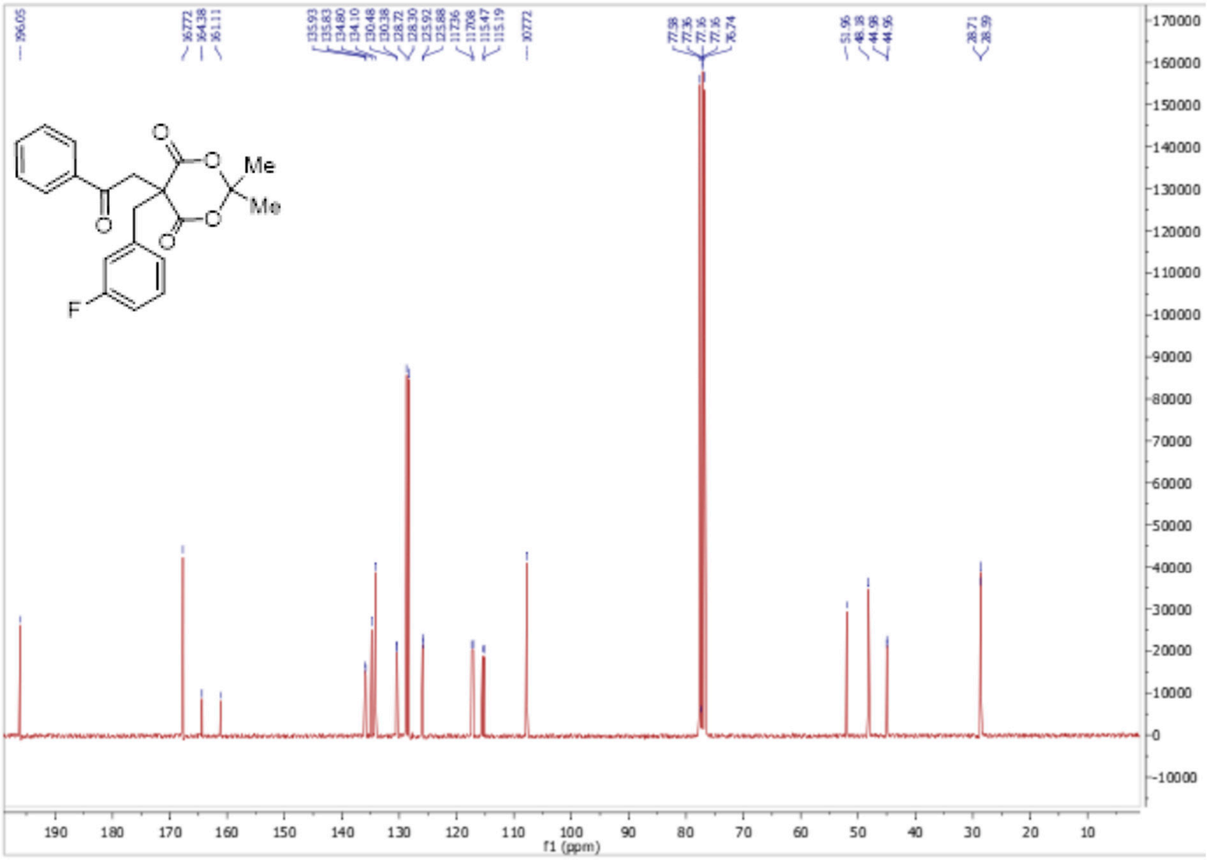

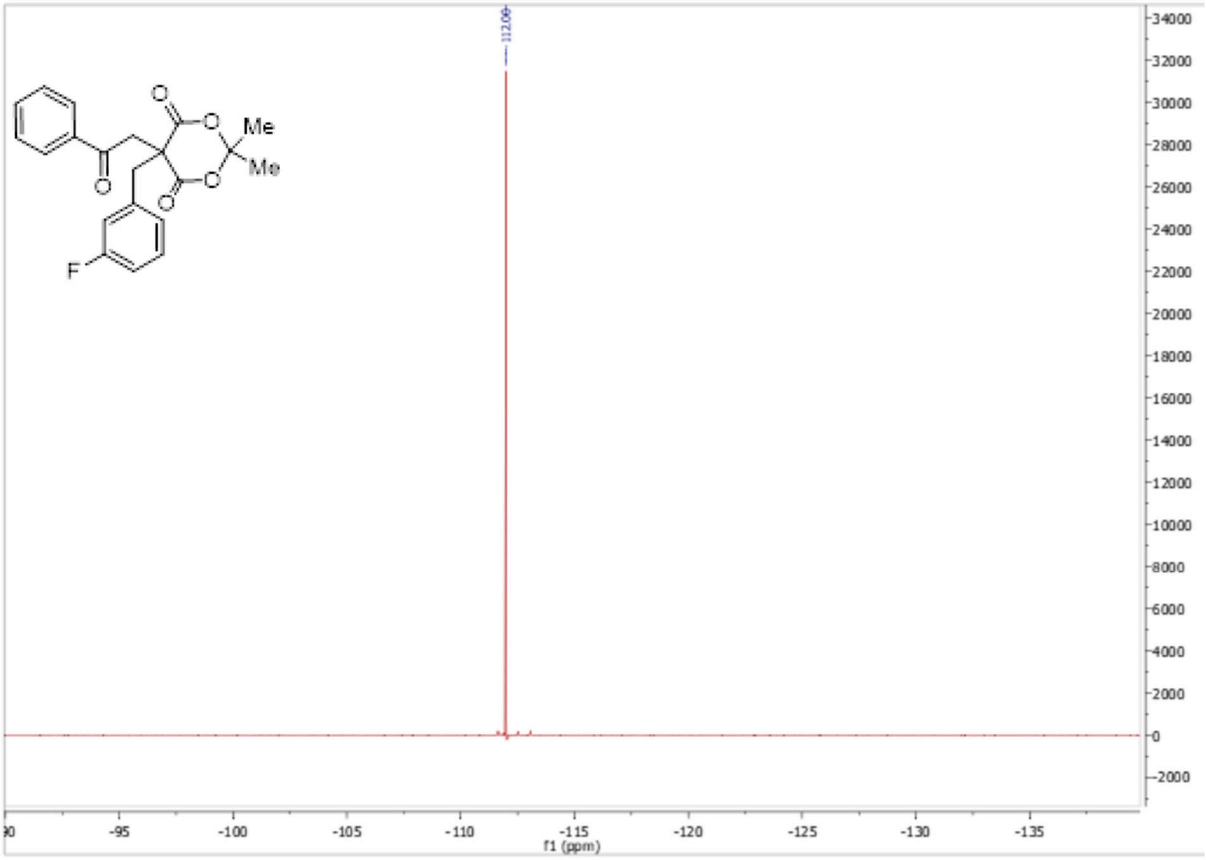

-112.09
Me
Me
F
34000
32000
30000
28000
26000
24000
22000
20000
18000
16000
14000
12000
10000
8000
6000
4000
2000
0
-2000
-95
-100
-105
-110
-115
-120
-125
-130
-135
f1 (ppm)

5-benzyl-2,2-dimethyl-5-(2-oxo-2-phenylethyl)-1,3-dioxane-4,6-dione (2b)

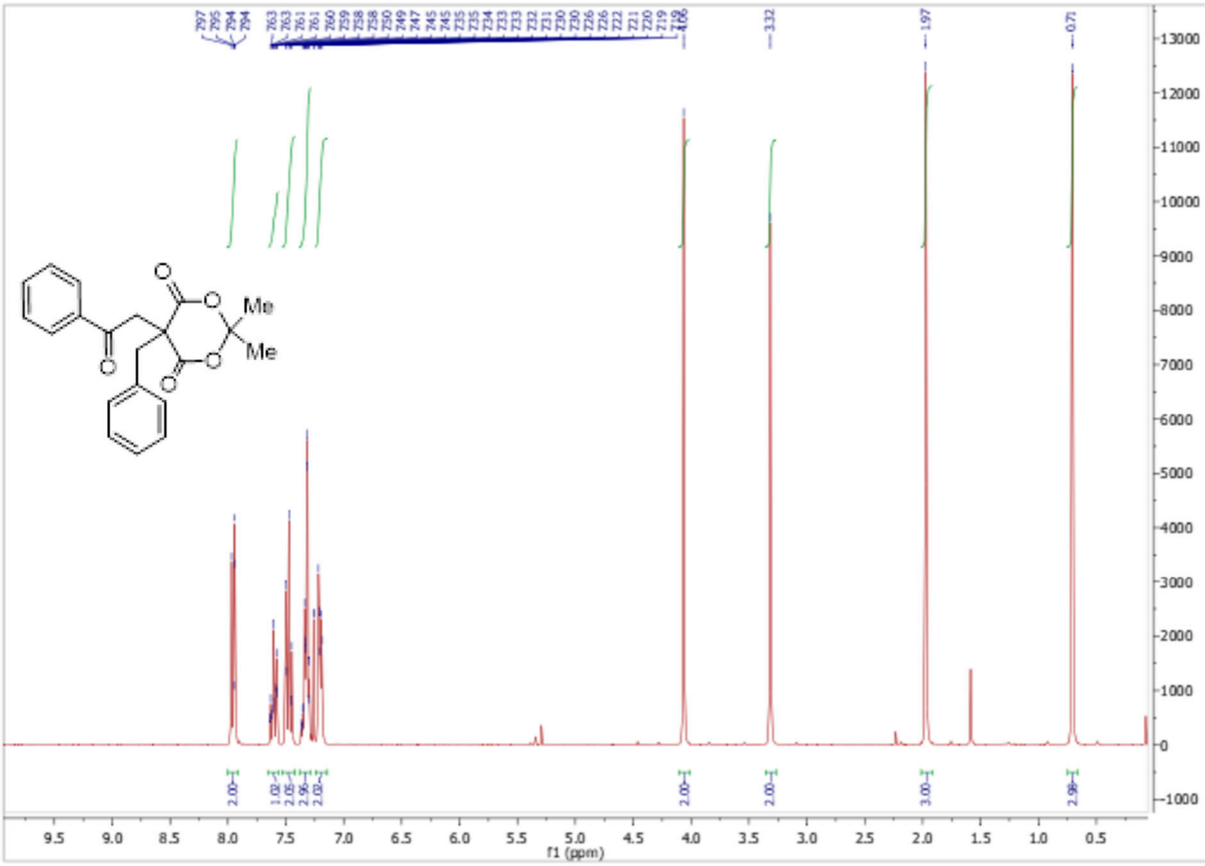

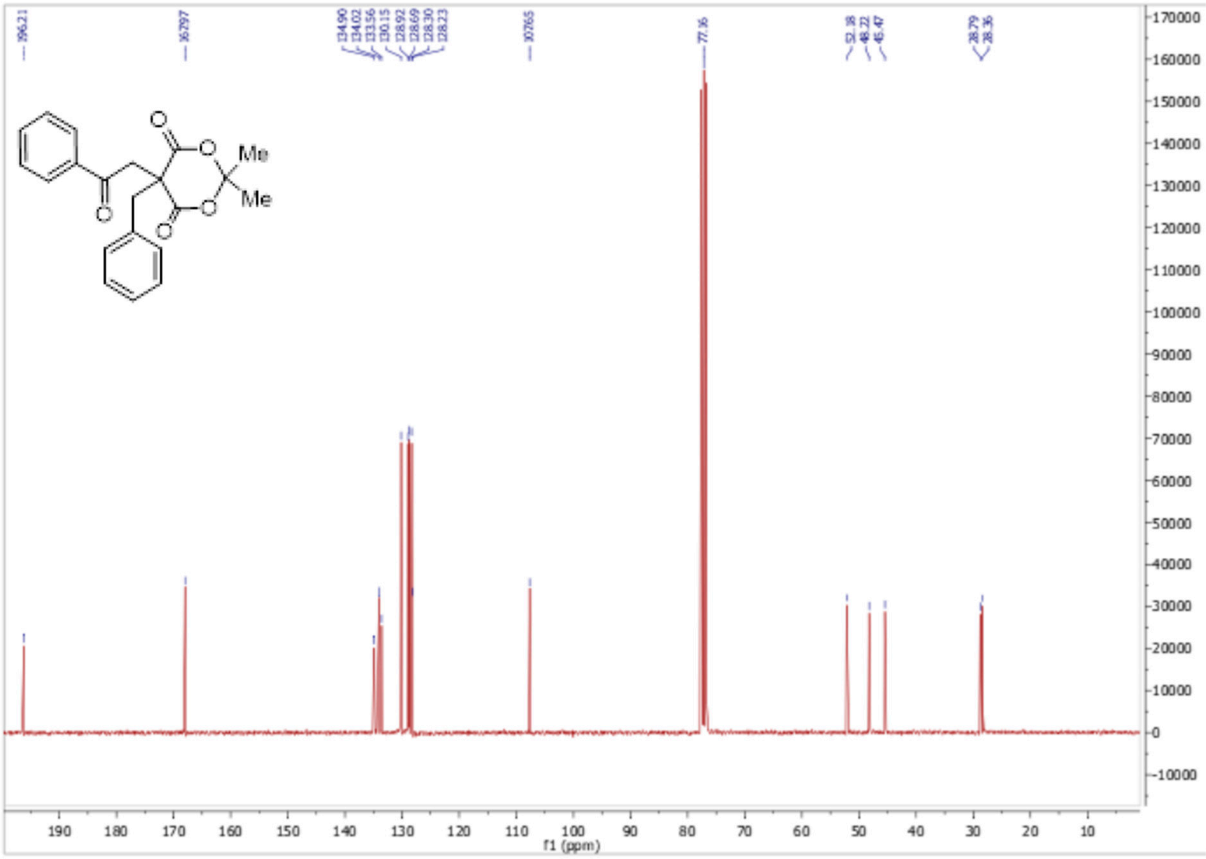

2,2-dimethyl-5-(naphthalen-1-ylmethyl)-5-(2-oxo-2-phenylethyl)-1,3-dioxane-4,6-dione (2c)

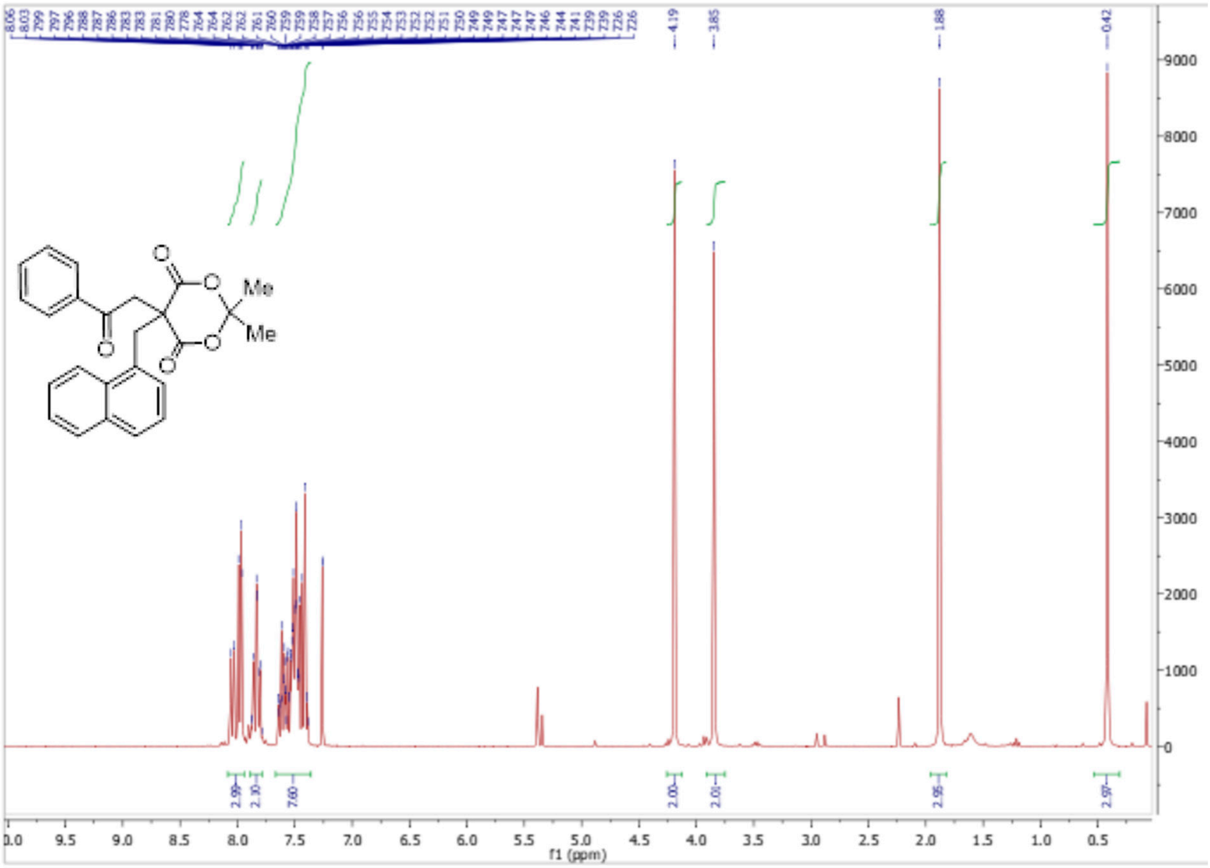

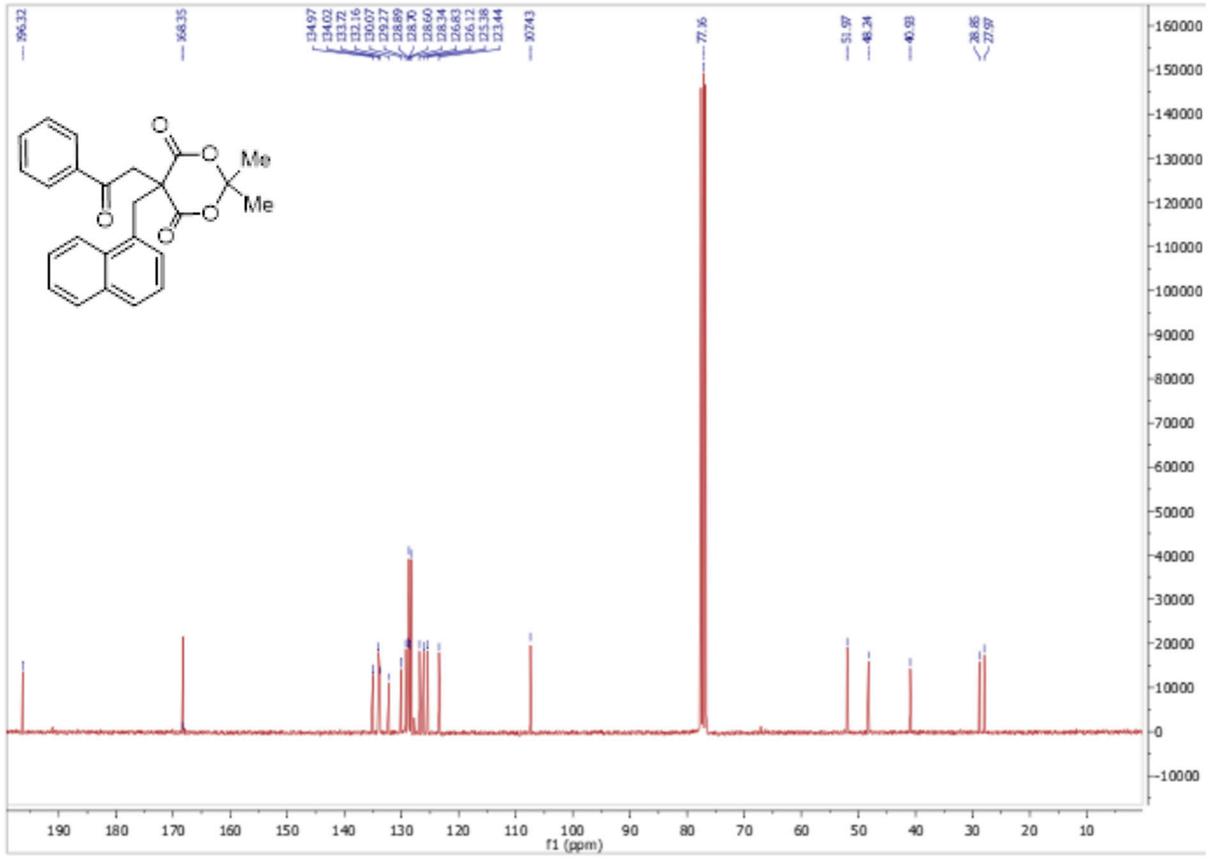

5-(4-fluorobenzyl)-2,2-dimethyl-5-(2-oxo-2-phenylethyl)-1,3-dioxane-4,6-dione (**2d**)

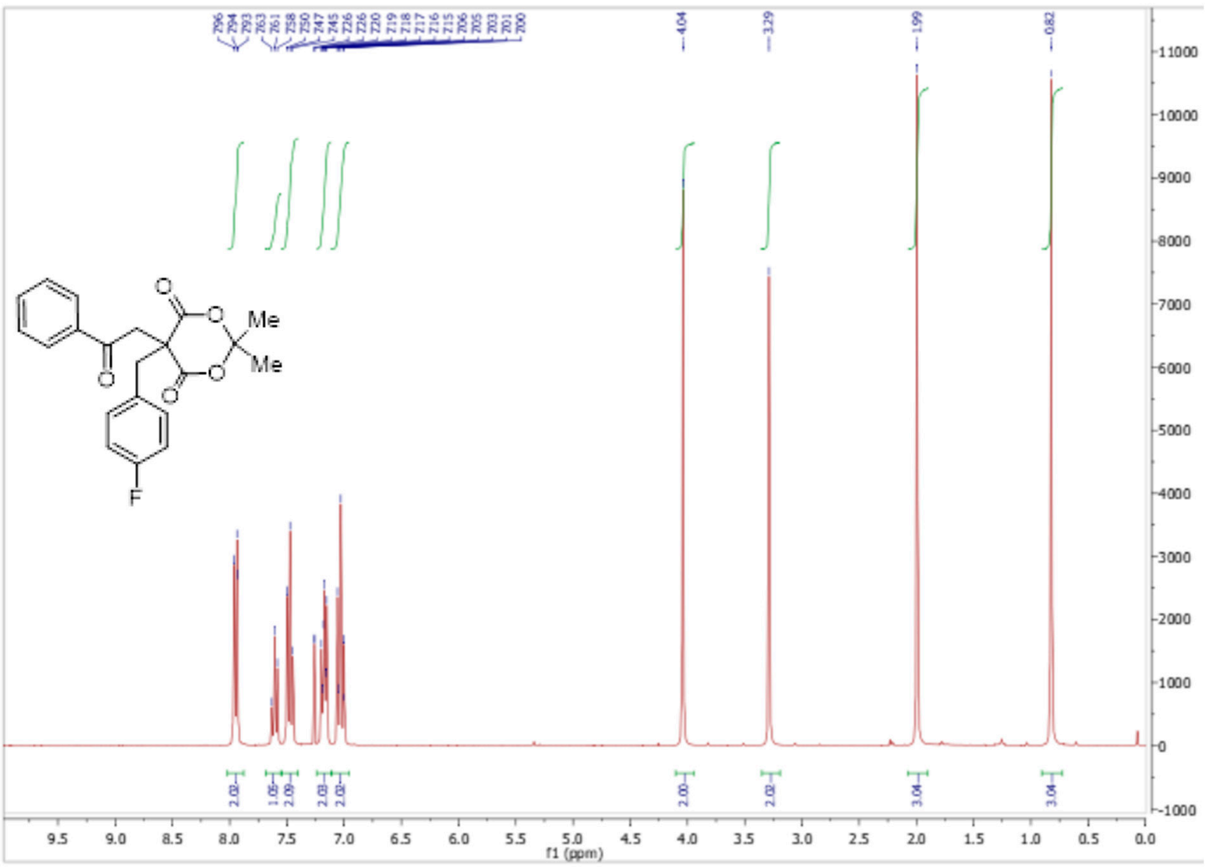

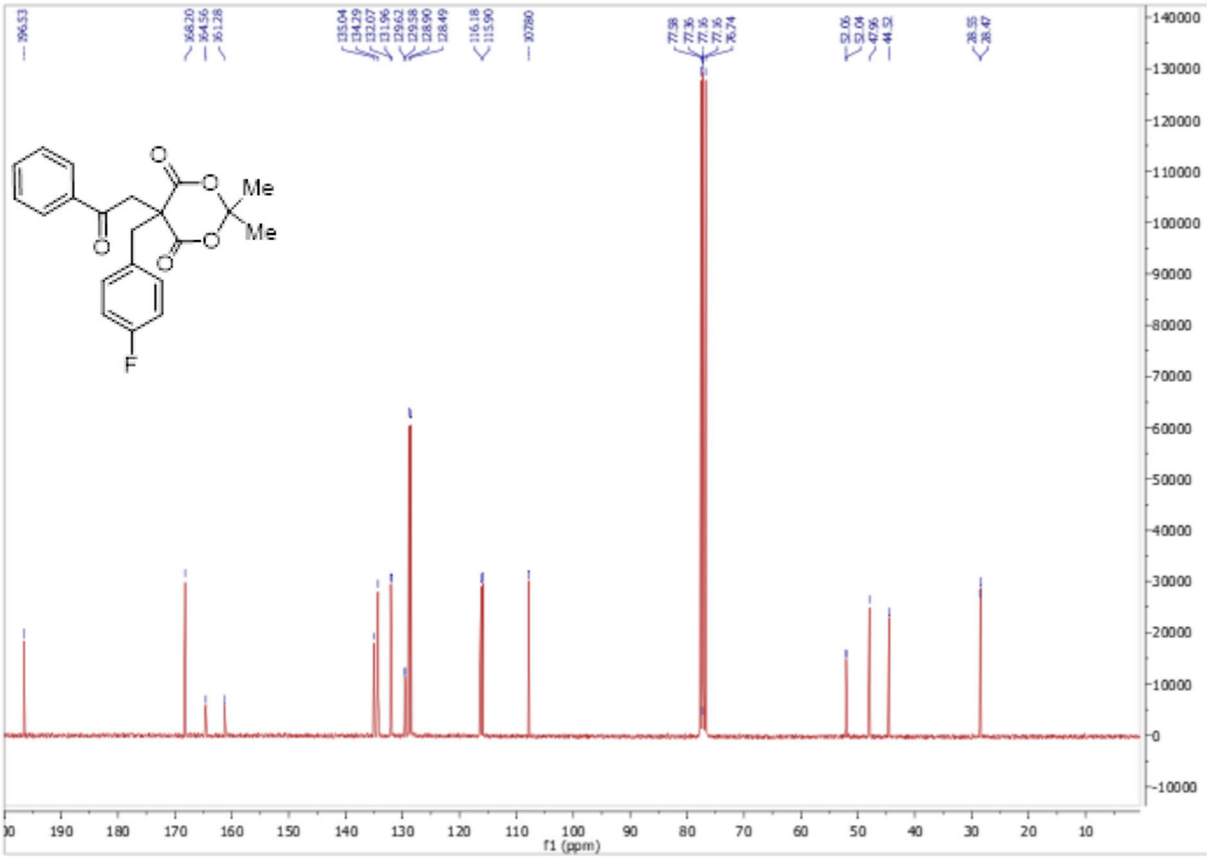

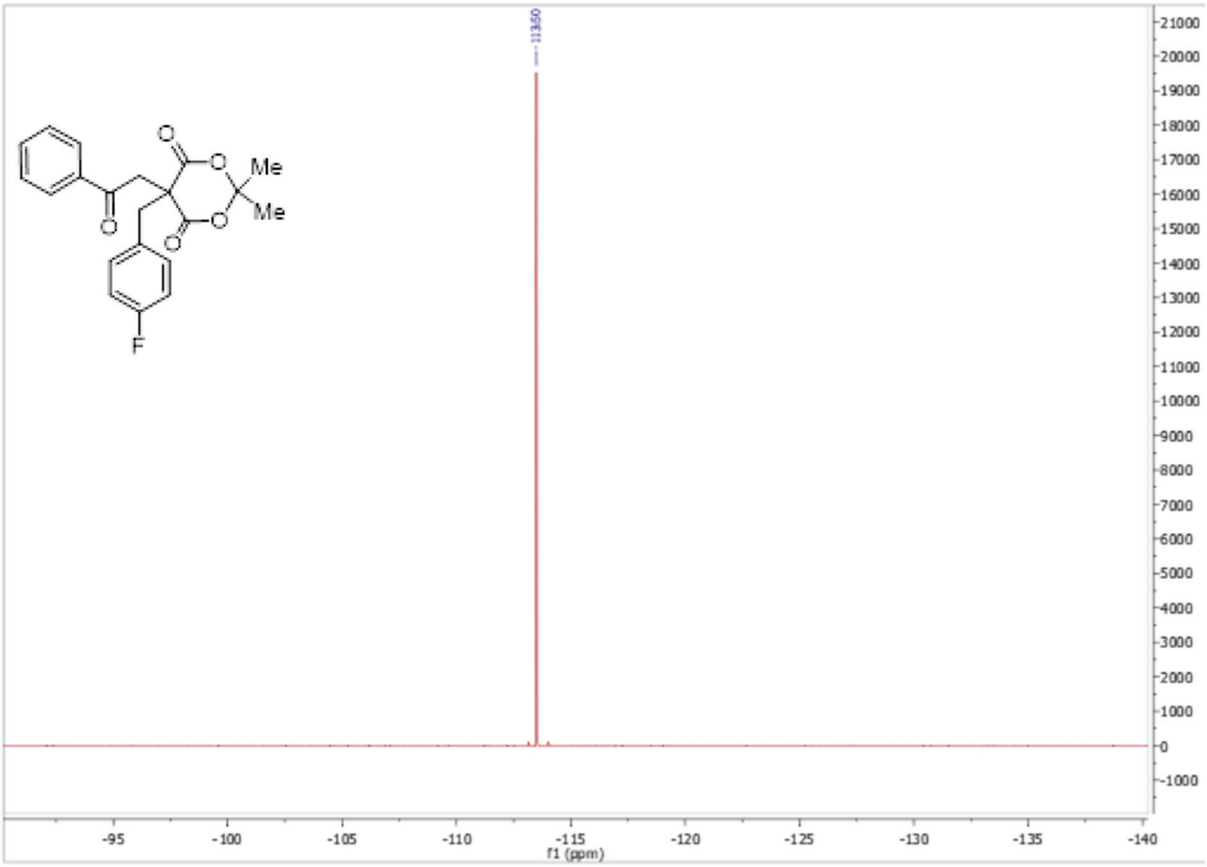

Me
Me
F
O
21000
20000
19000
18000
17000
16000
15000
14000
13000
12000
11000
10000
9000
8000
7000
6000
5000
4000
3000
2000
1000
0
-1000
-95
-100
-105
-110
-115
-120
-125
-130
-135
-140
f1 (ppm)

5-(4-bromobenzyl)-2,2-dimethyl-5-(2-oxo-2-phenylethyl)-1,3-dioxane-4,6-dione (2e)

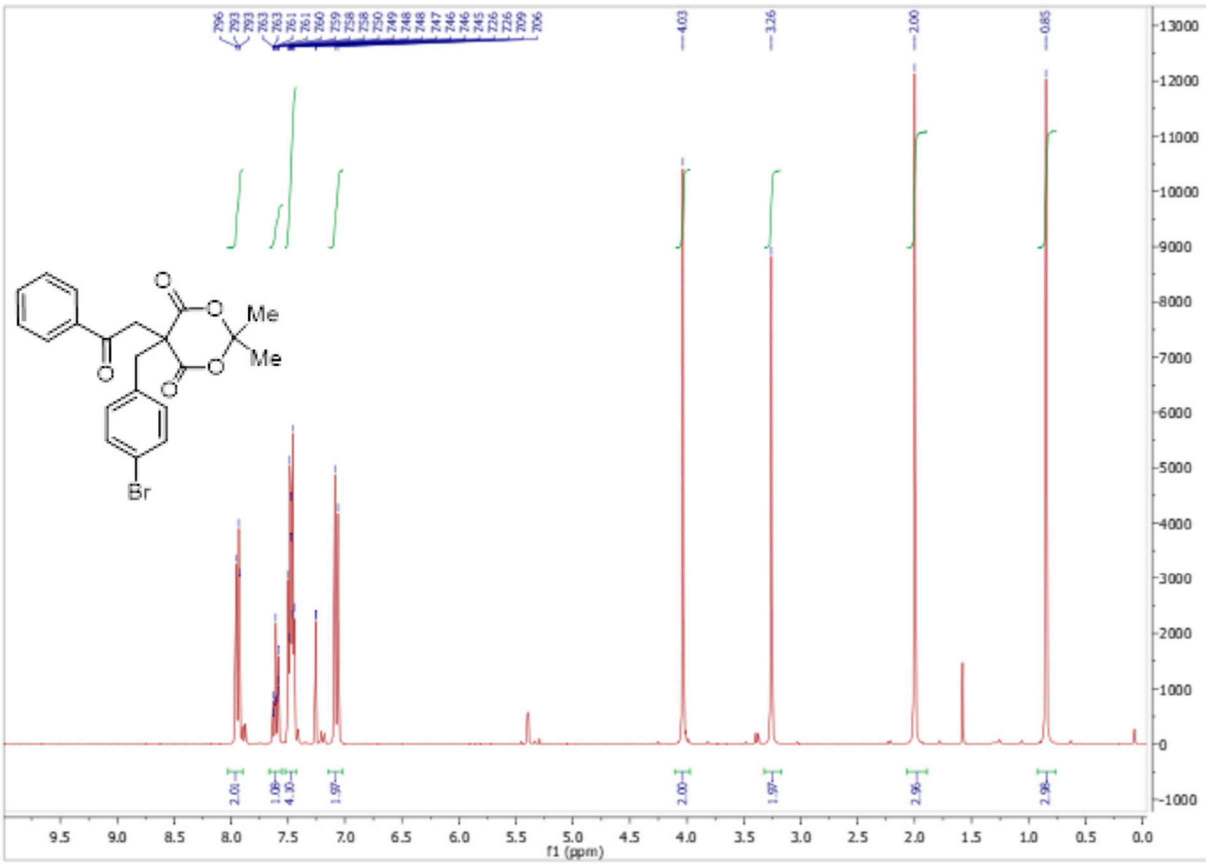

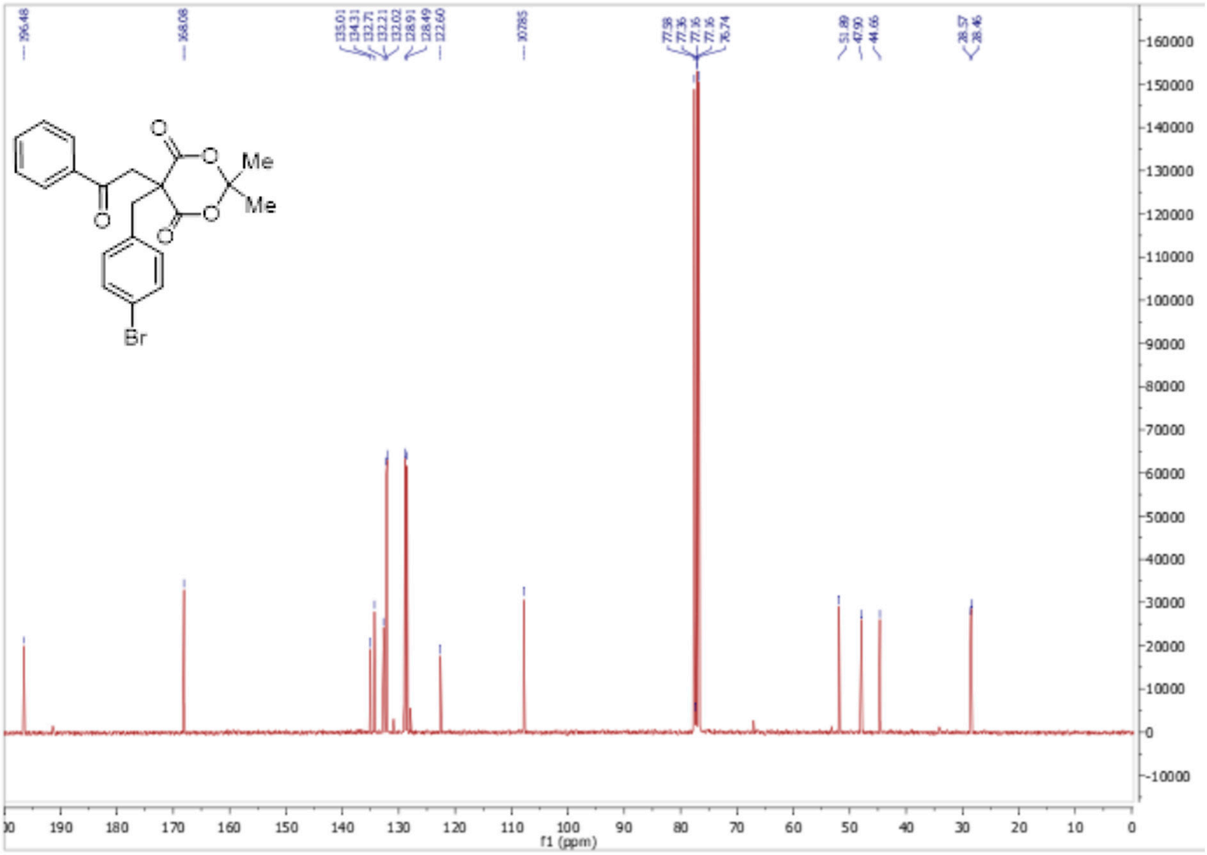

5-(4-chlorobenzyl)-2,2-dimethyl-5-(2-oxo-2-phenylethyl)-1,3-dioxane-4,6-dione (2f)

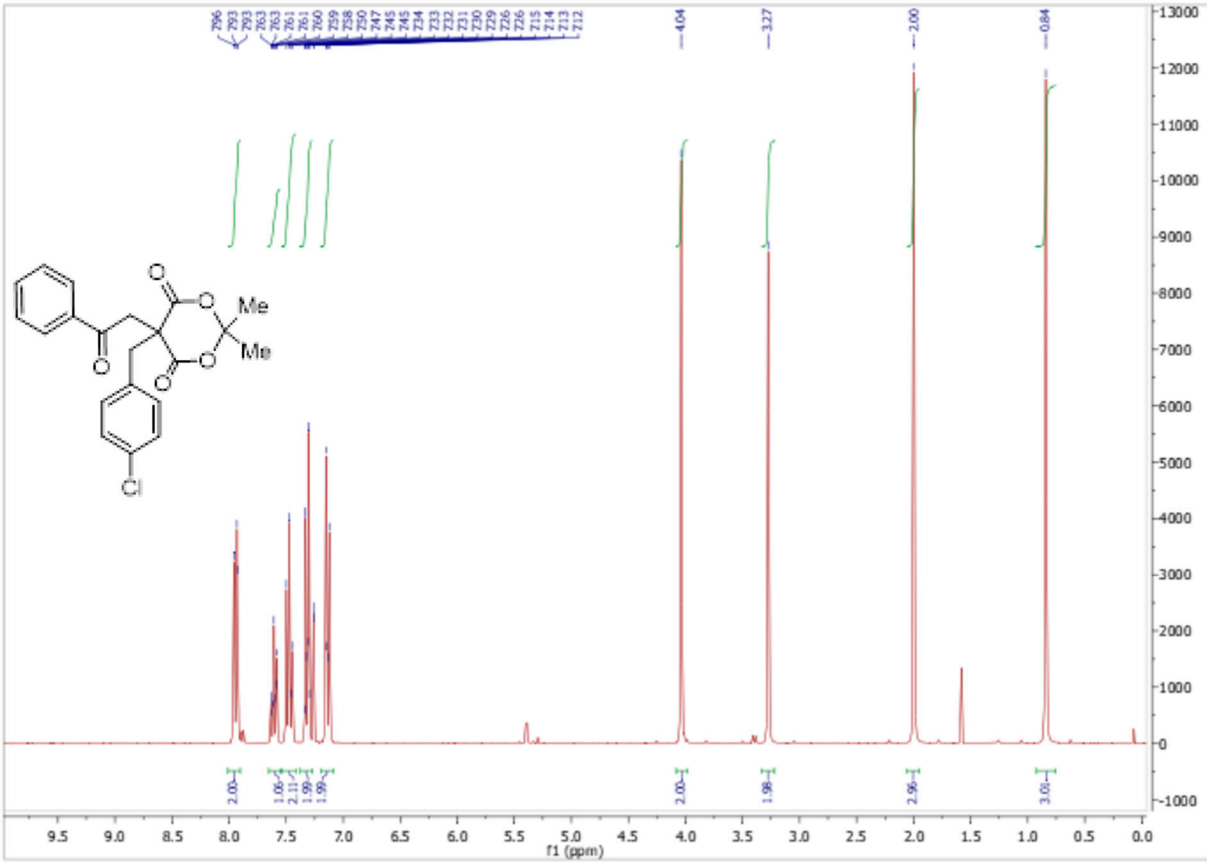

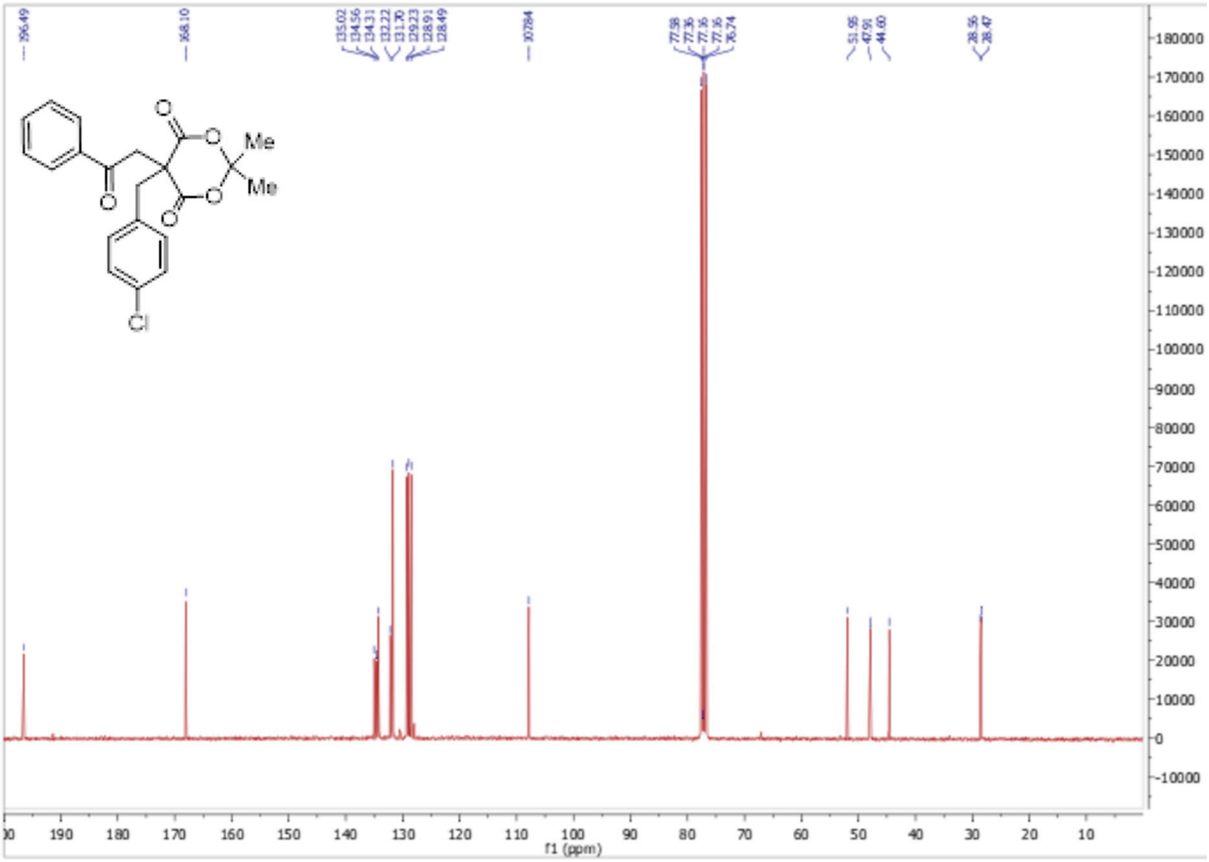

2-((2,2-dimethyl-4,6-dioxo-5-(2-oxo-2-phenylethyl)-1,3-dioxan-5-yl)methyl)benzonitrile (2g)

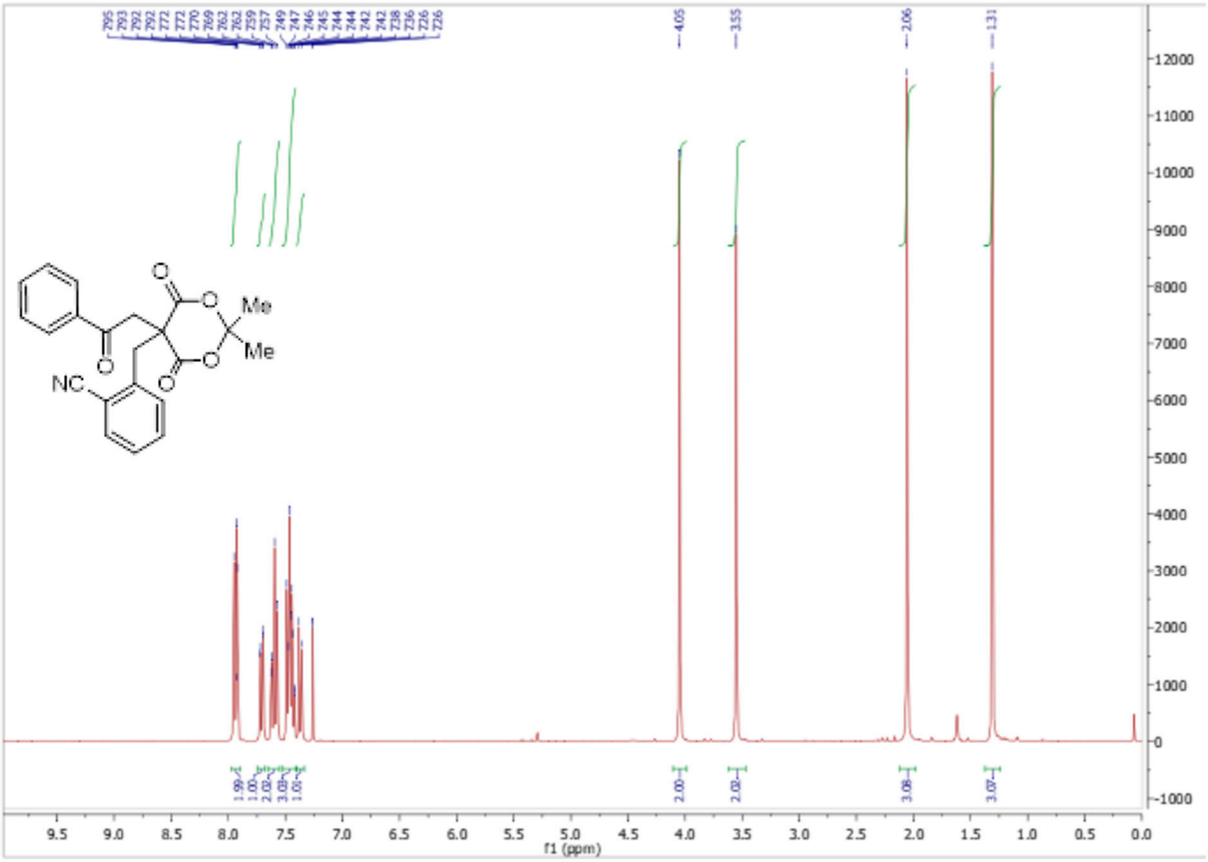

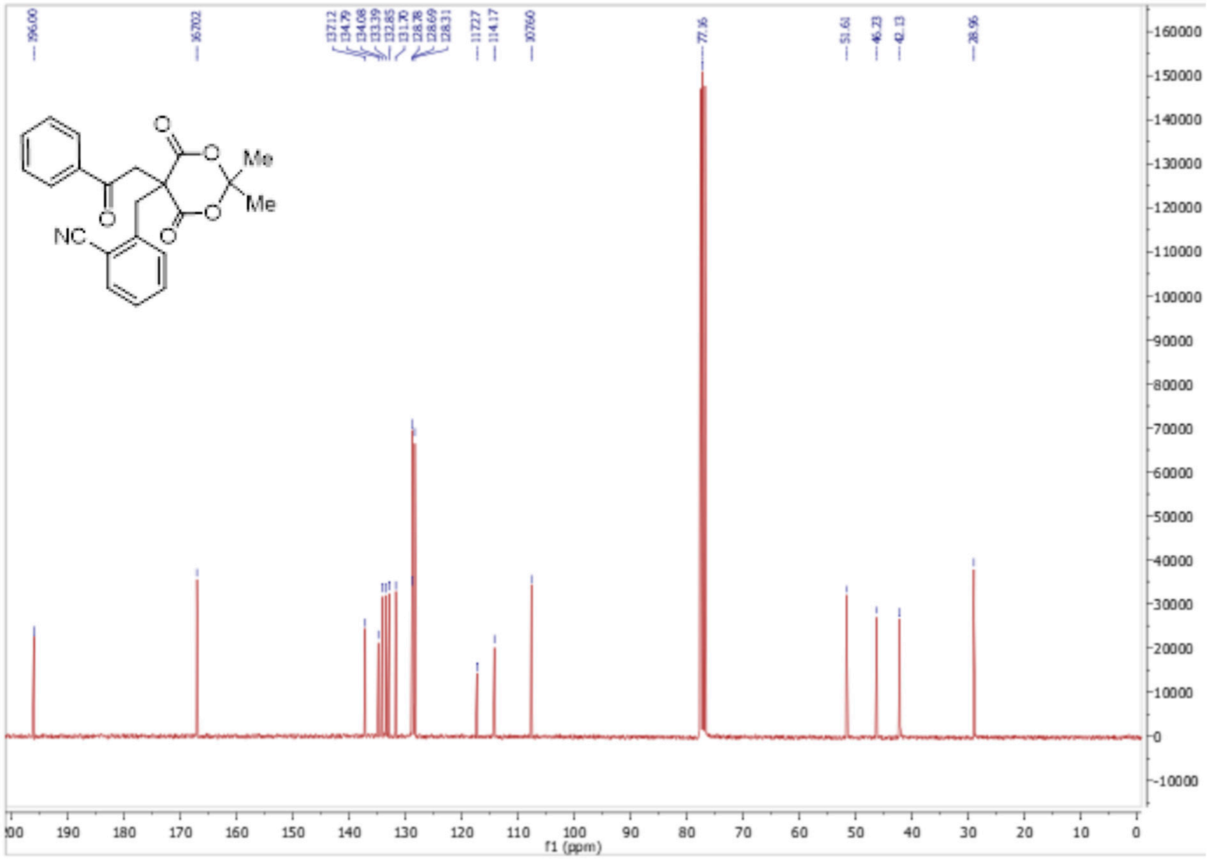

5-(2-methoxybenzyl)-2,2-dimethyl-5-(2-oxo-2-phenylethyl)-1,3-dioxane-4,6-dione (2h)

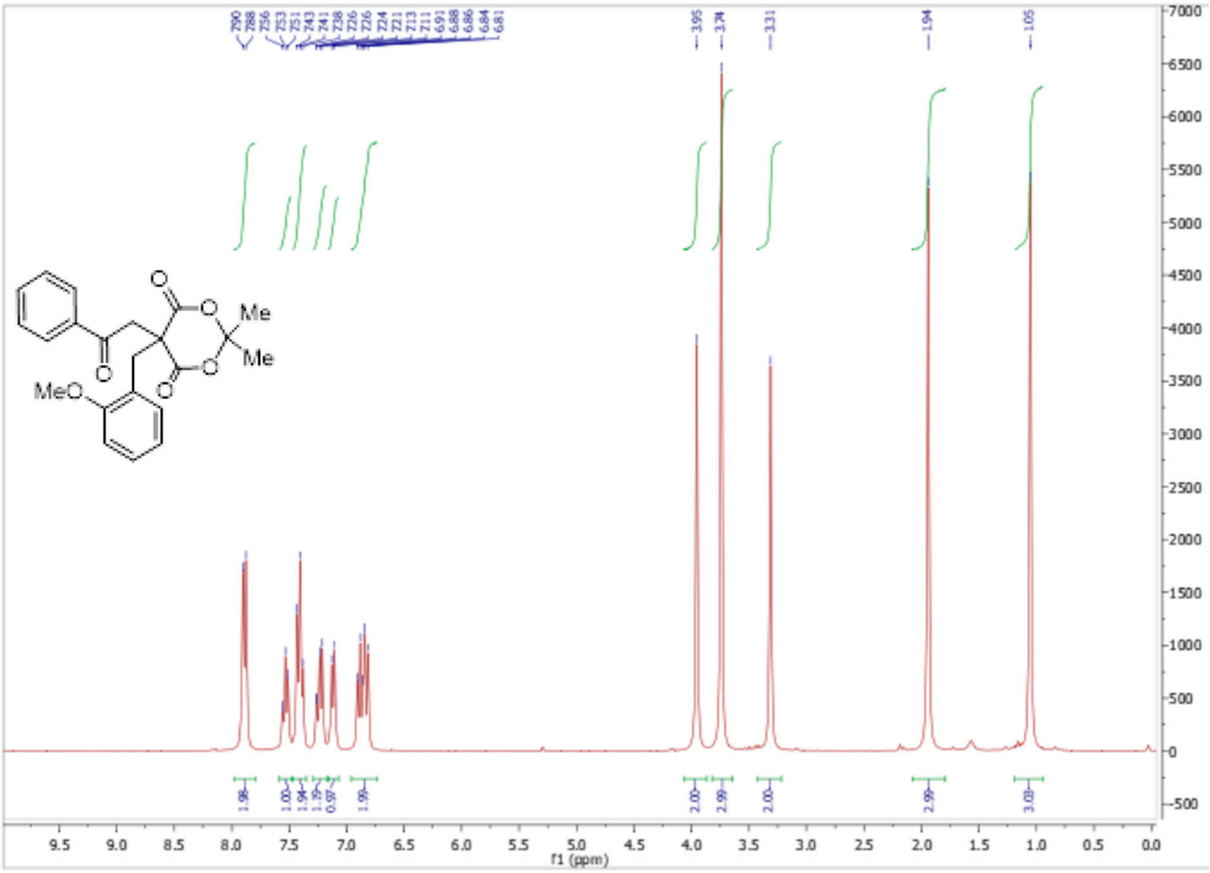

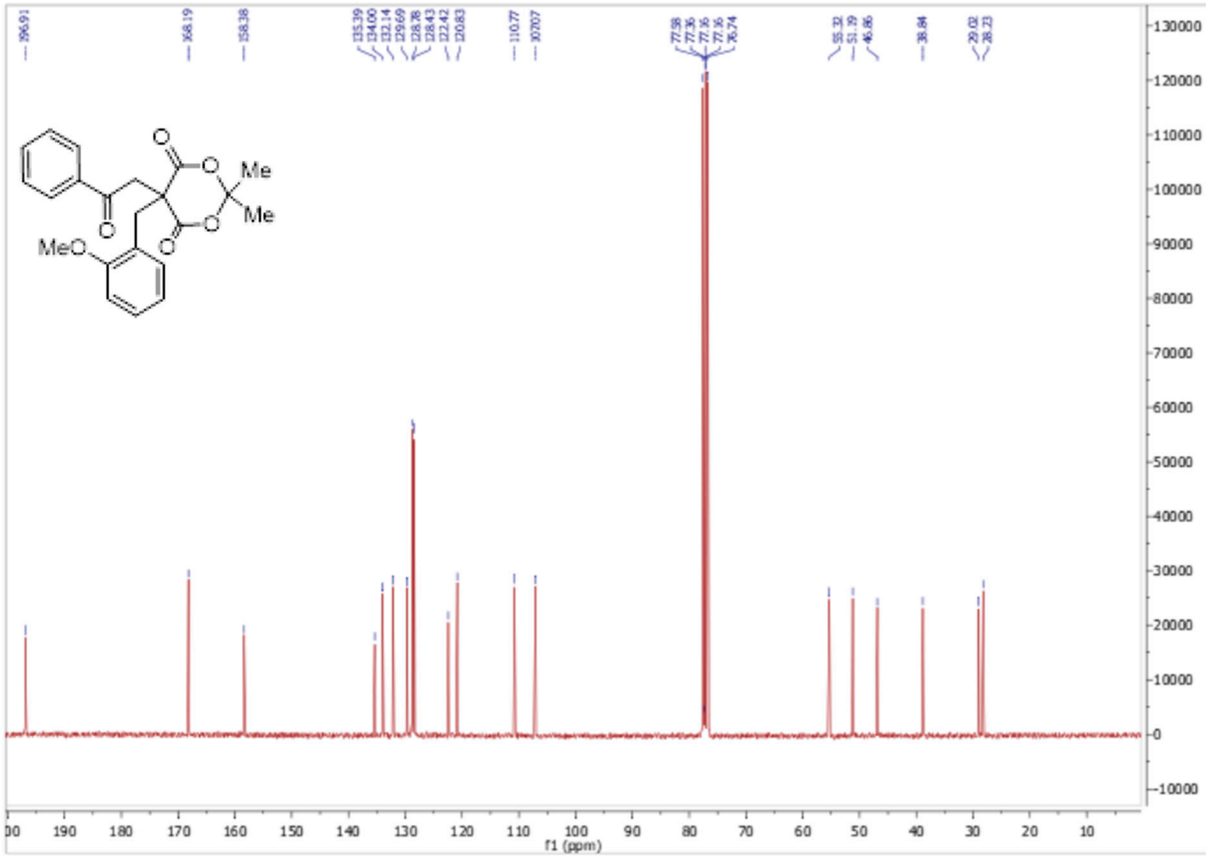

2,2-dimethyl-5-(4-methylbenzyl)-5-(2-oxo-2-phenylethyl)-1,3-dioxane-4,6-dione (2i)

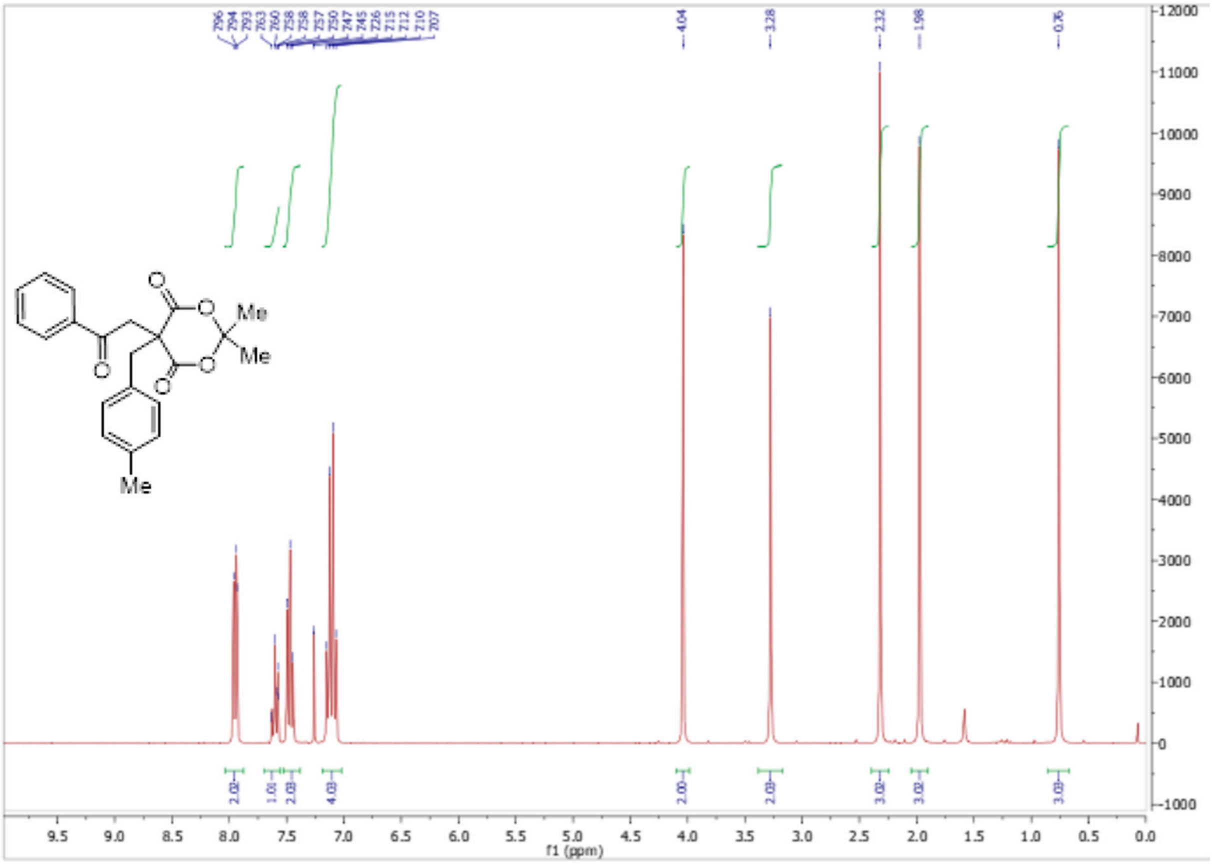

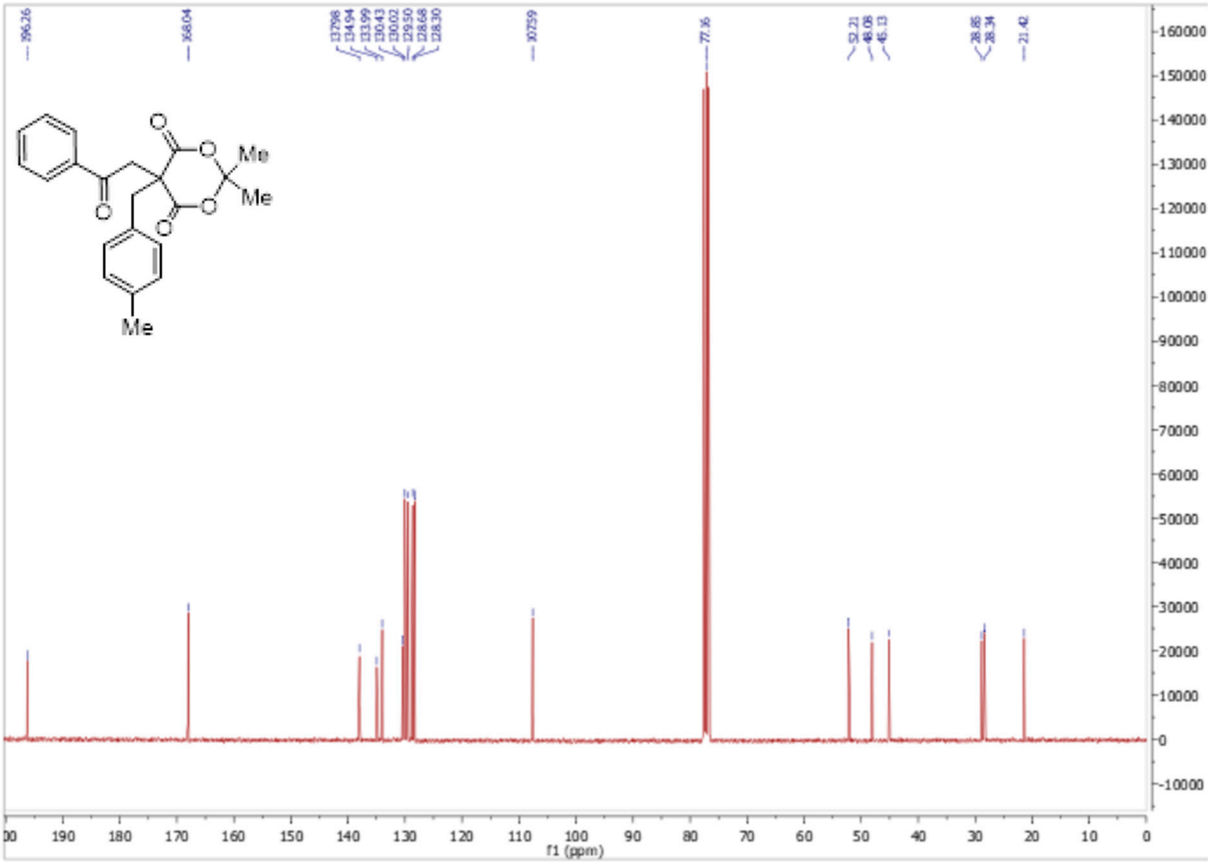

5-(2,5-dimethylbenzyl)-2,2-dimethyl-5-(2-oxo-2-phenylethyl)-1,3-dioxane-4,6-dione (2j)

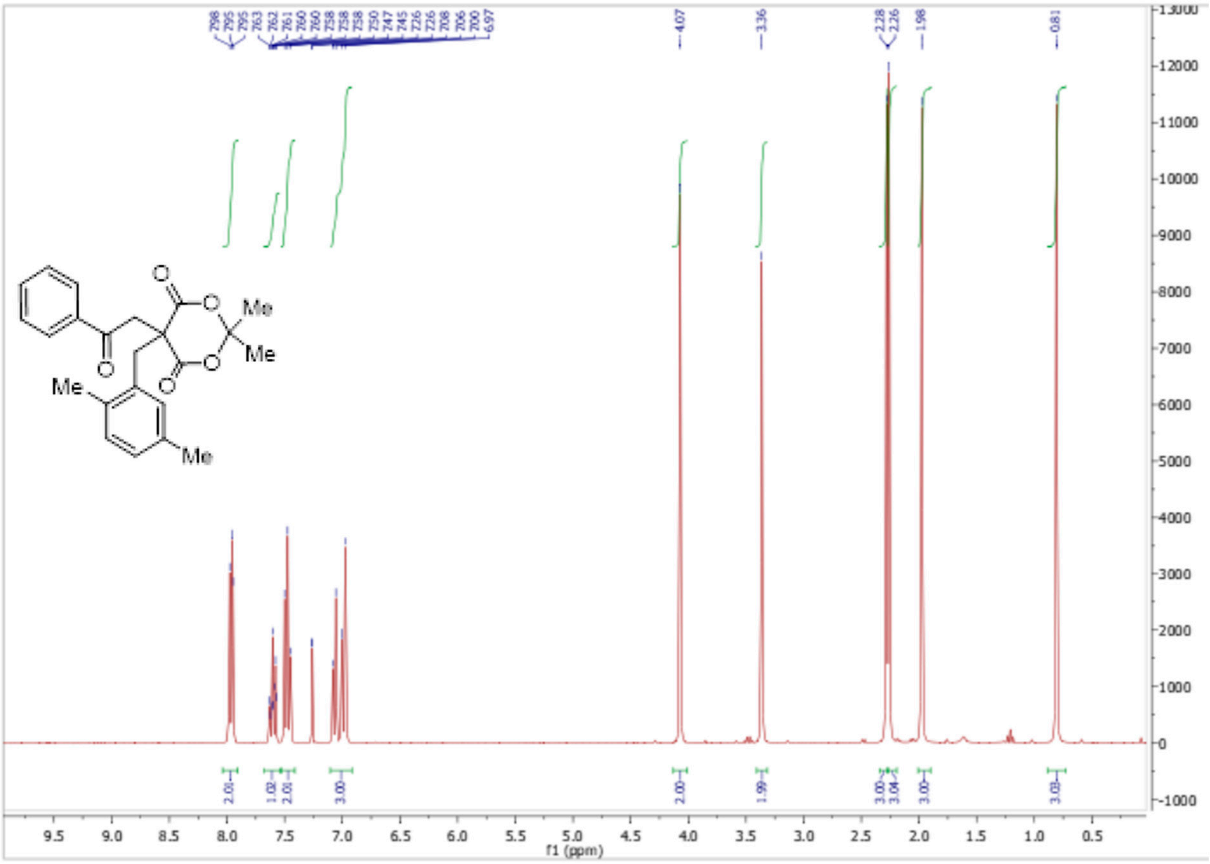

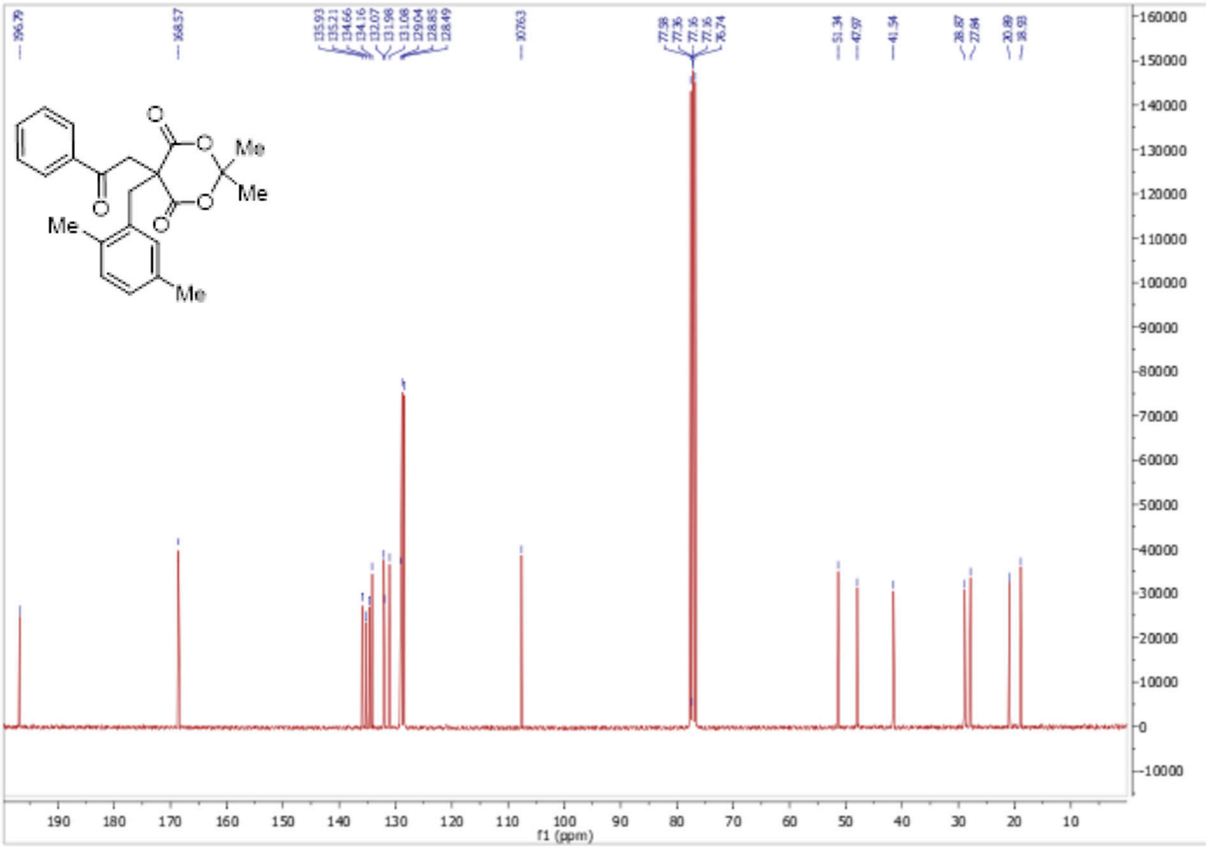

2,2-dimethyl-5-(2-oxo-2-phenylethyl)-5-(thiophen-2-ylmethyl)-1,3-dioxane-4,6-dione (2k)

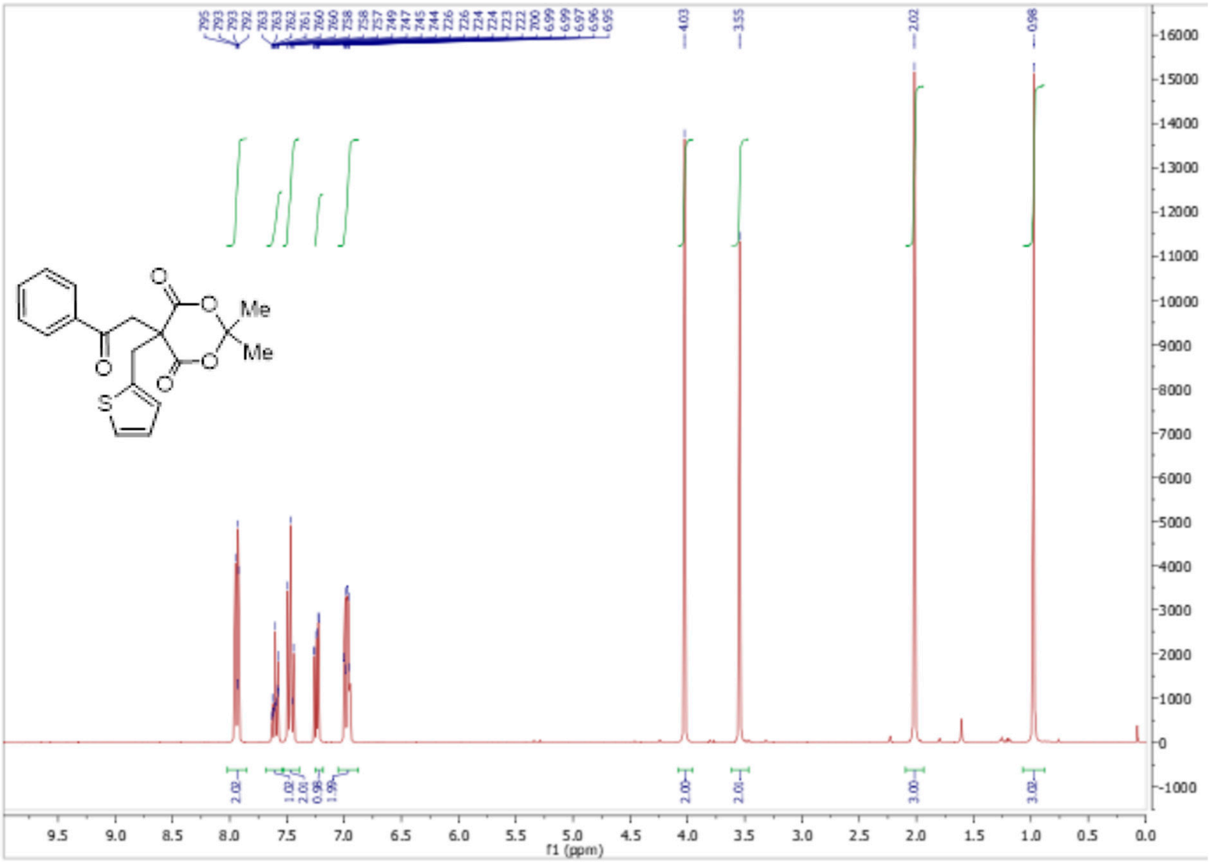

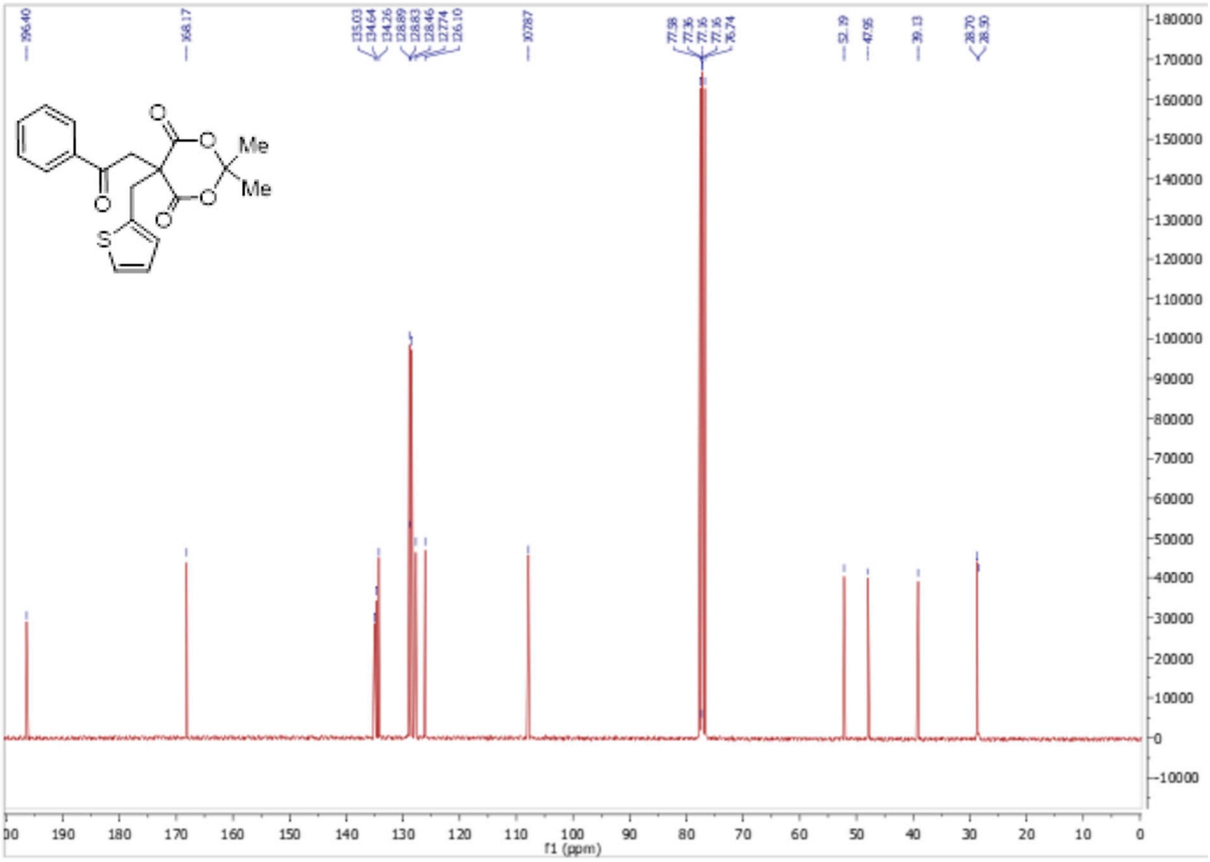

5-isobutyl-2,2-dimethyl-5-(2-oxo-2-phenylethyl)-1,3-dioxane-4,6-dione (**2l**)

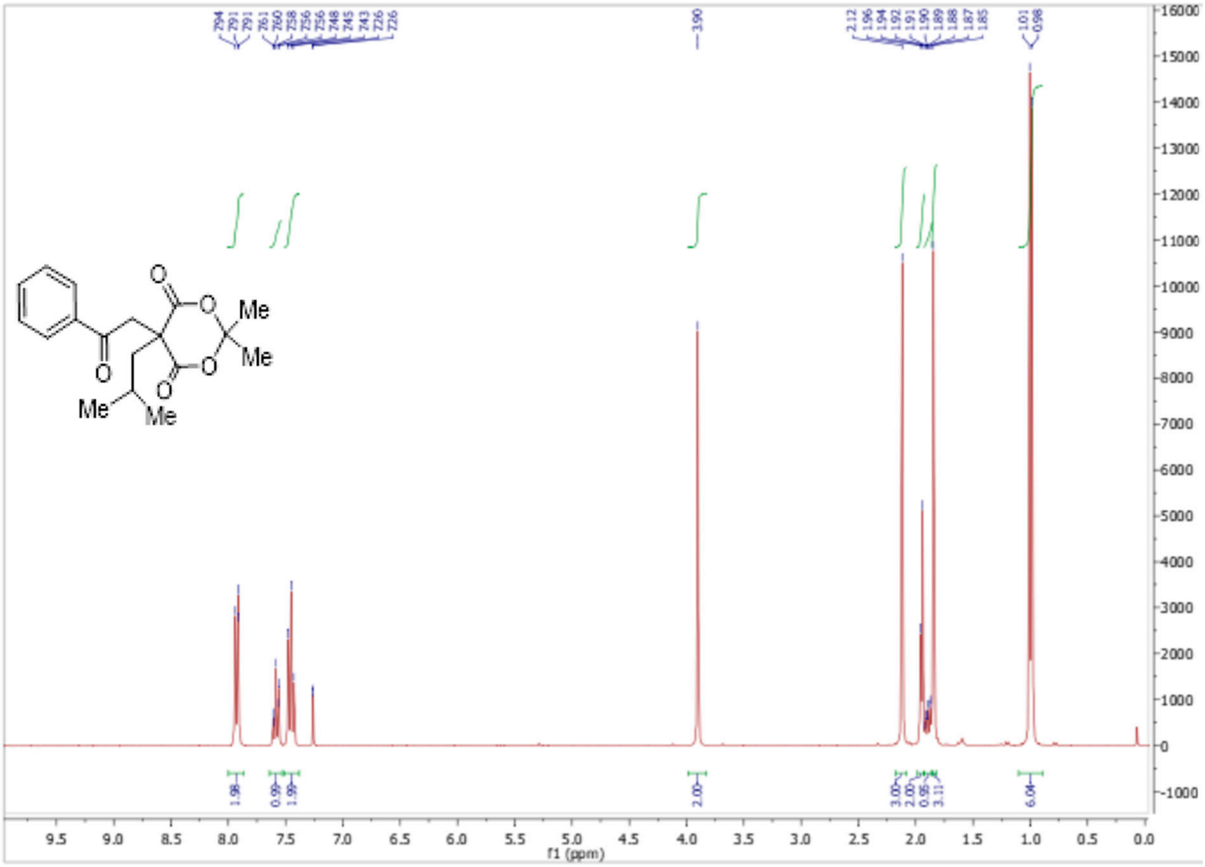

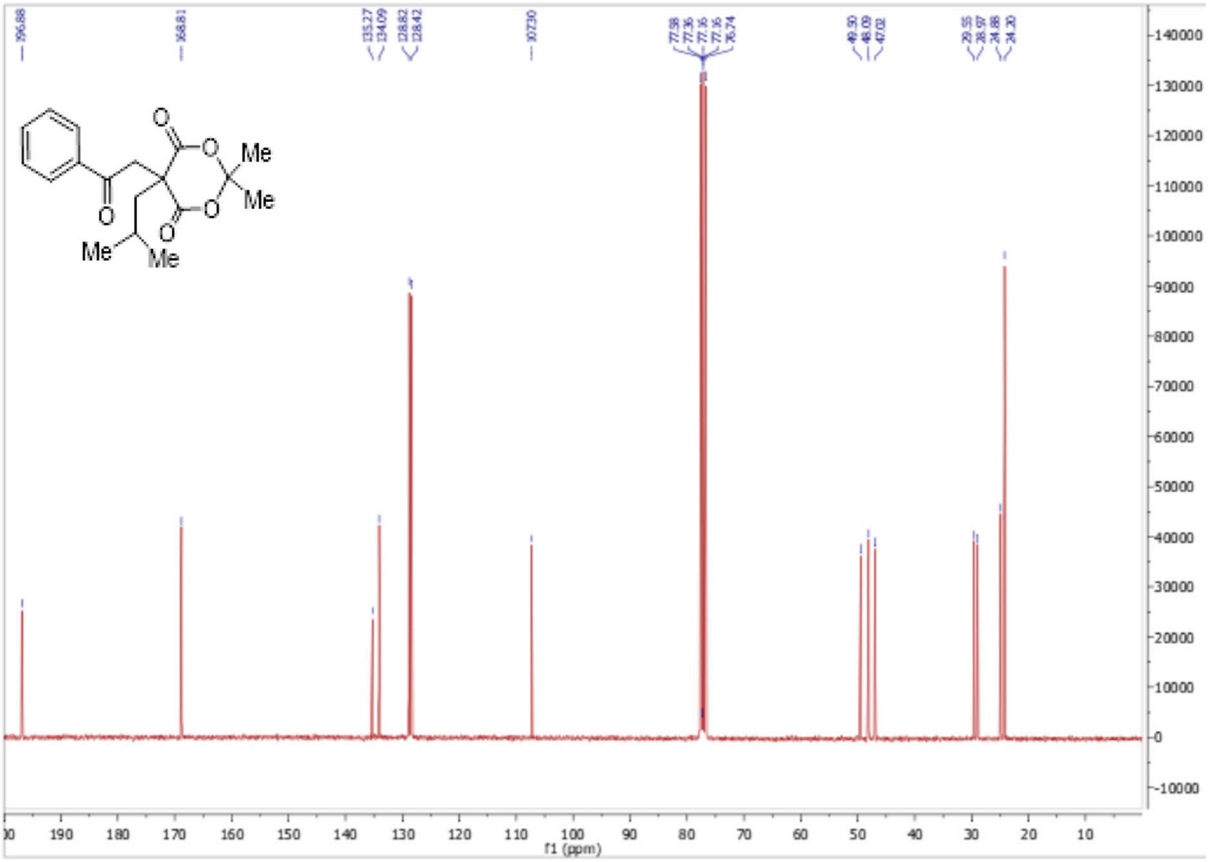

2,2-dimethyl-5-(2-oxo-2-phenylethyl)-5-(3-phenylpropyl)-1,3-dioxane-4,6-dione (2m)

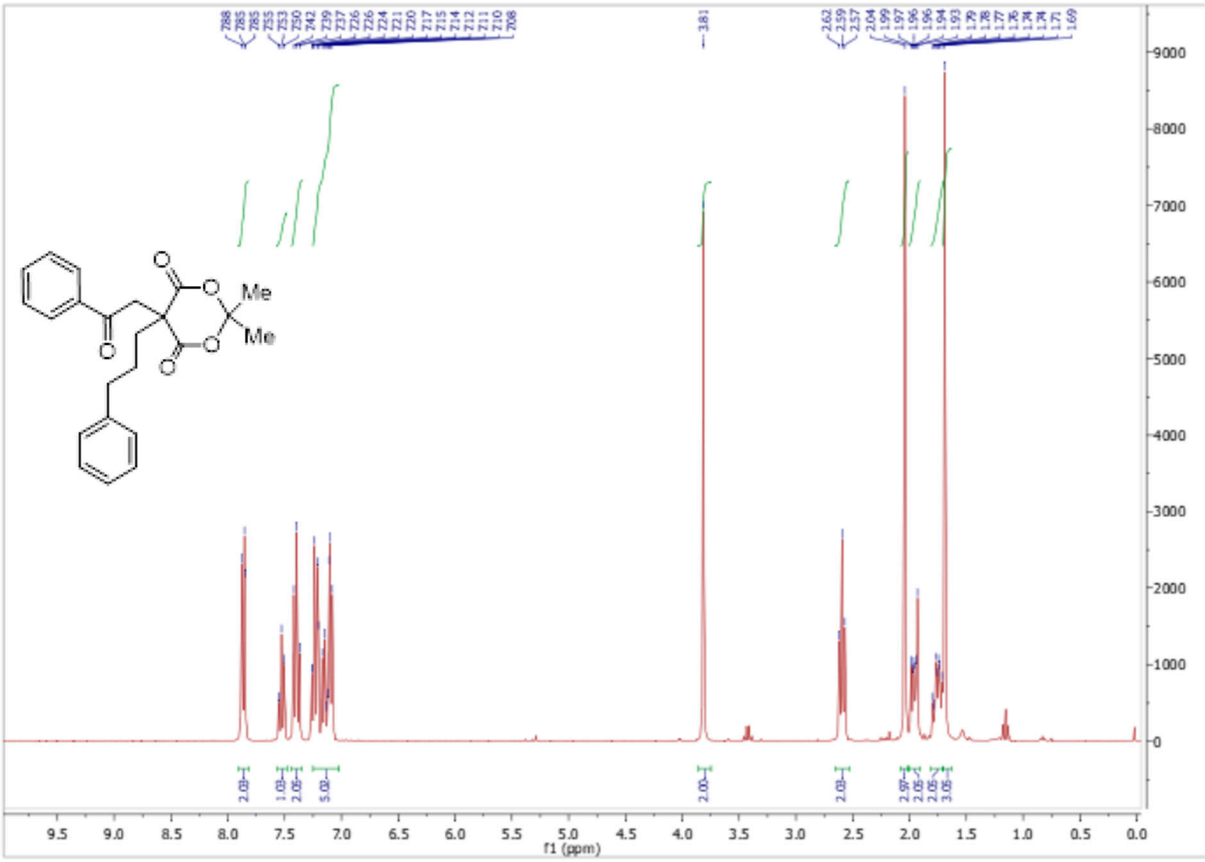

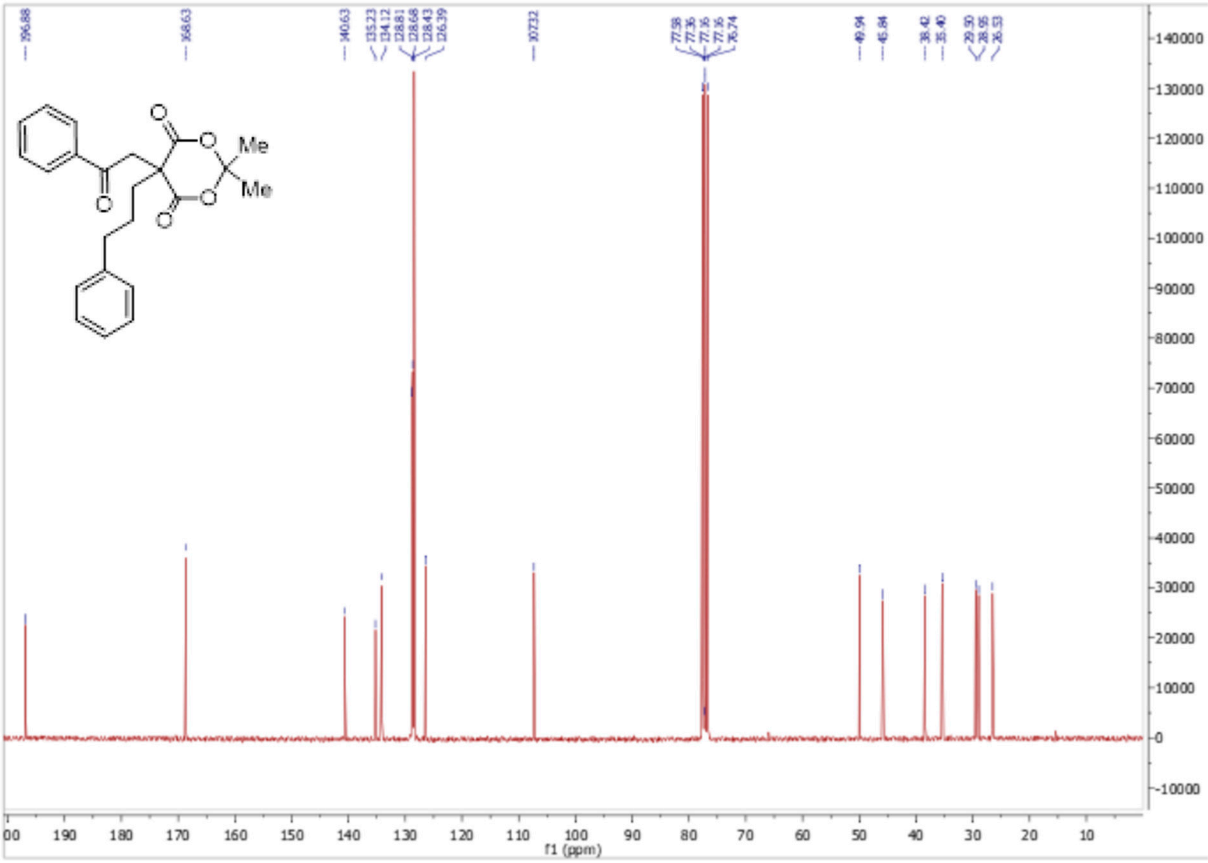

2,2-dimethyl-5-(2-oxo-2-phenylethyl)-5-phenyl-1,3-dioxane-4,6-dione (2n)

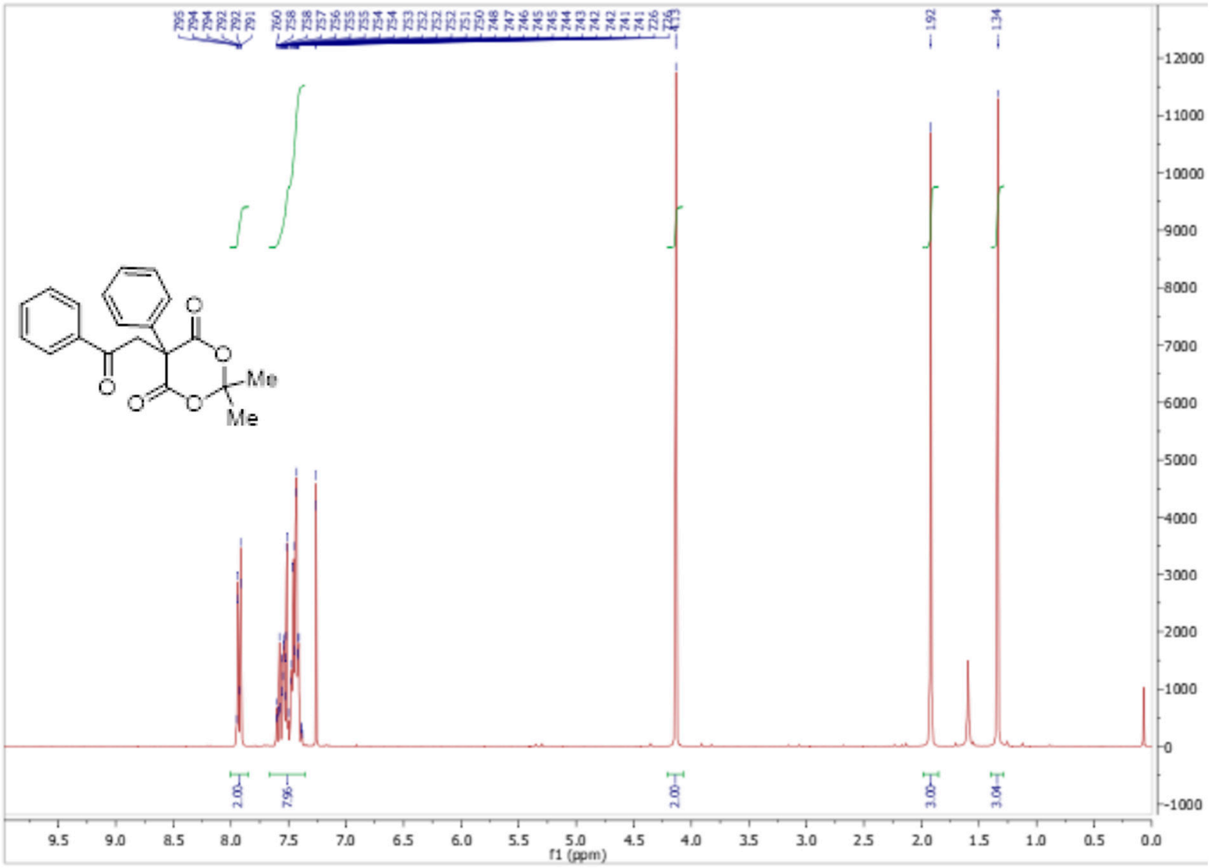

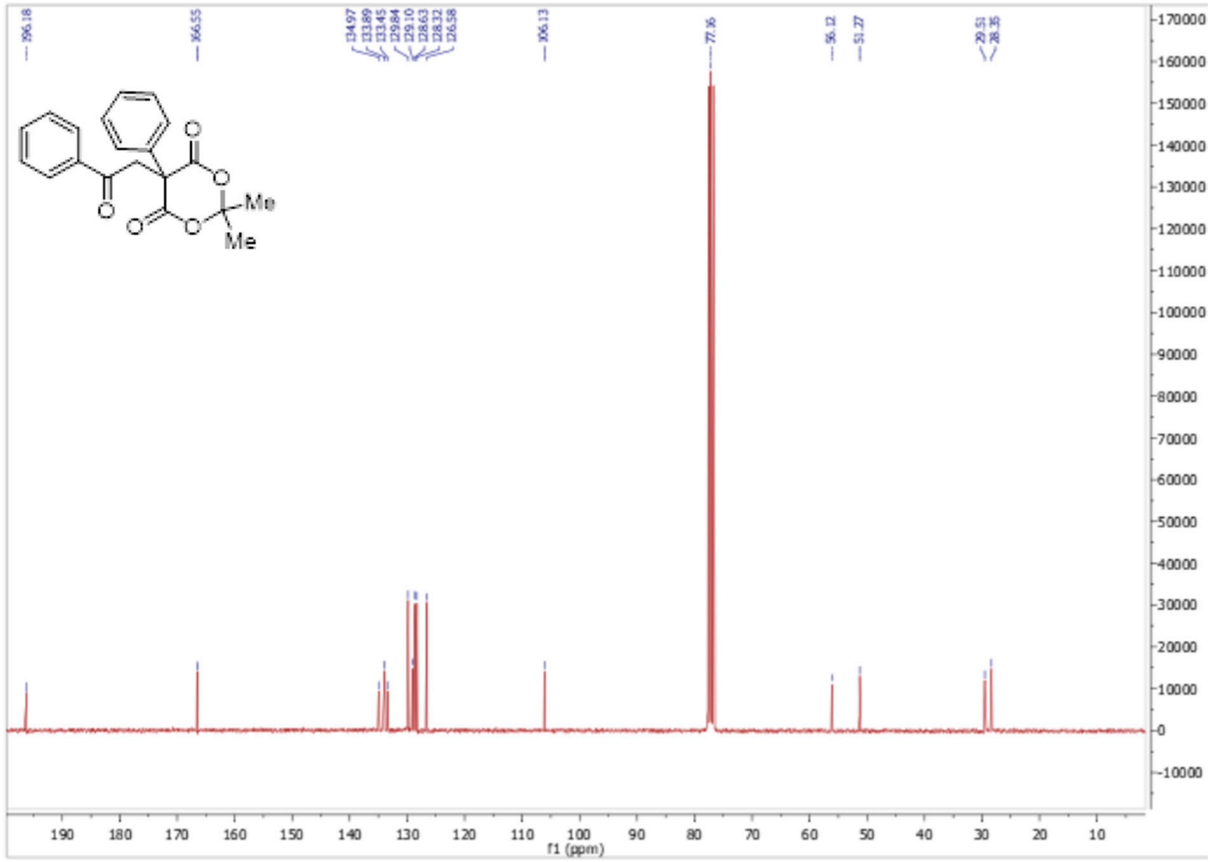

IV. 1. 3. NMR spectra of NH-pyridazinones

4-(3-fluorobenzyl)-6-phenyl-4,5-dihydropyridazin-3(*2H*)-one (**3a**)

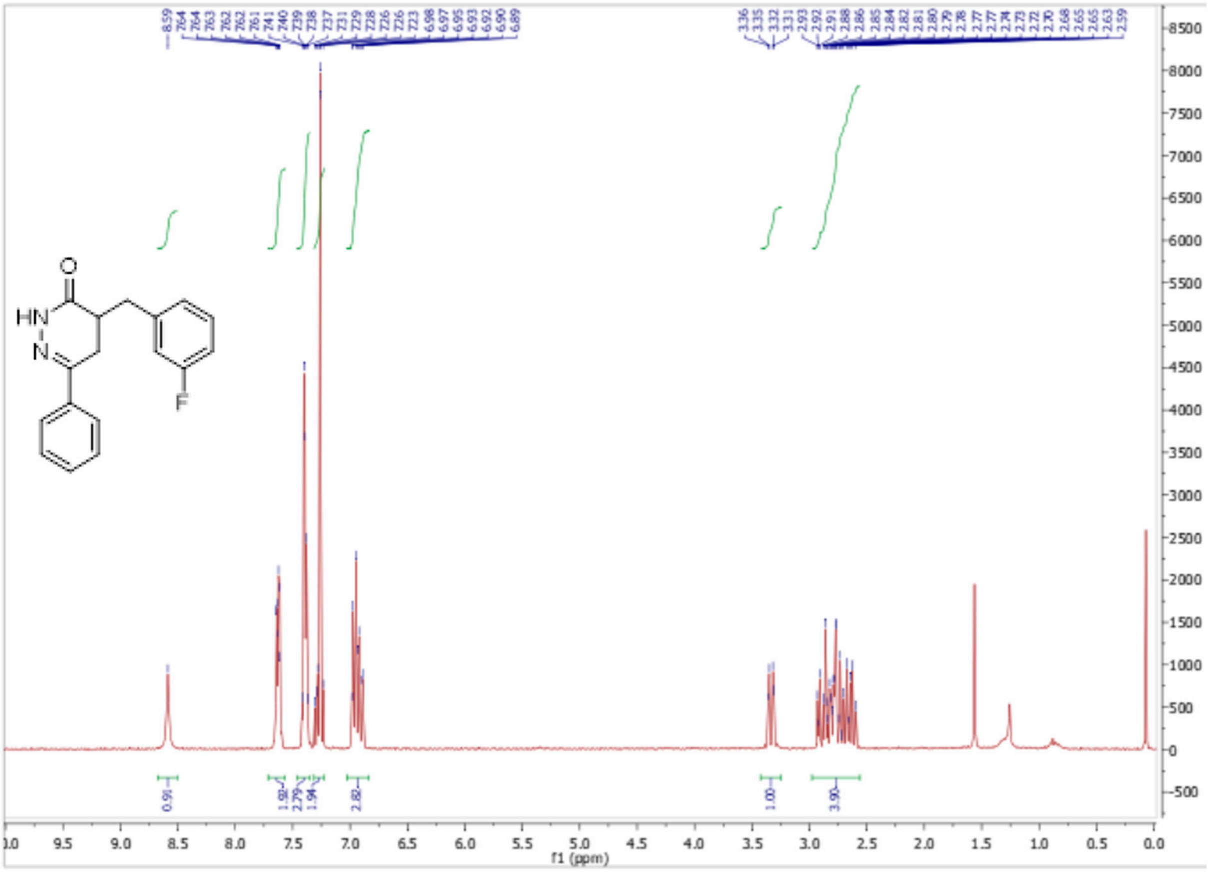

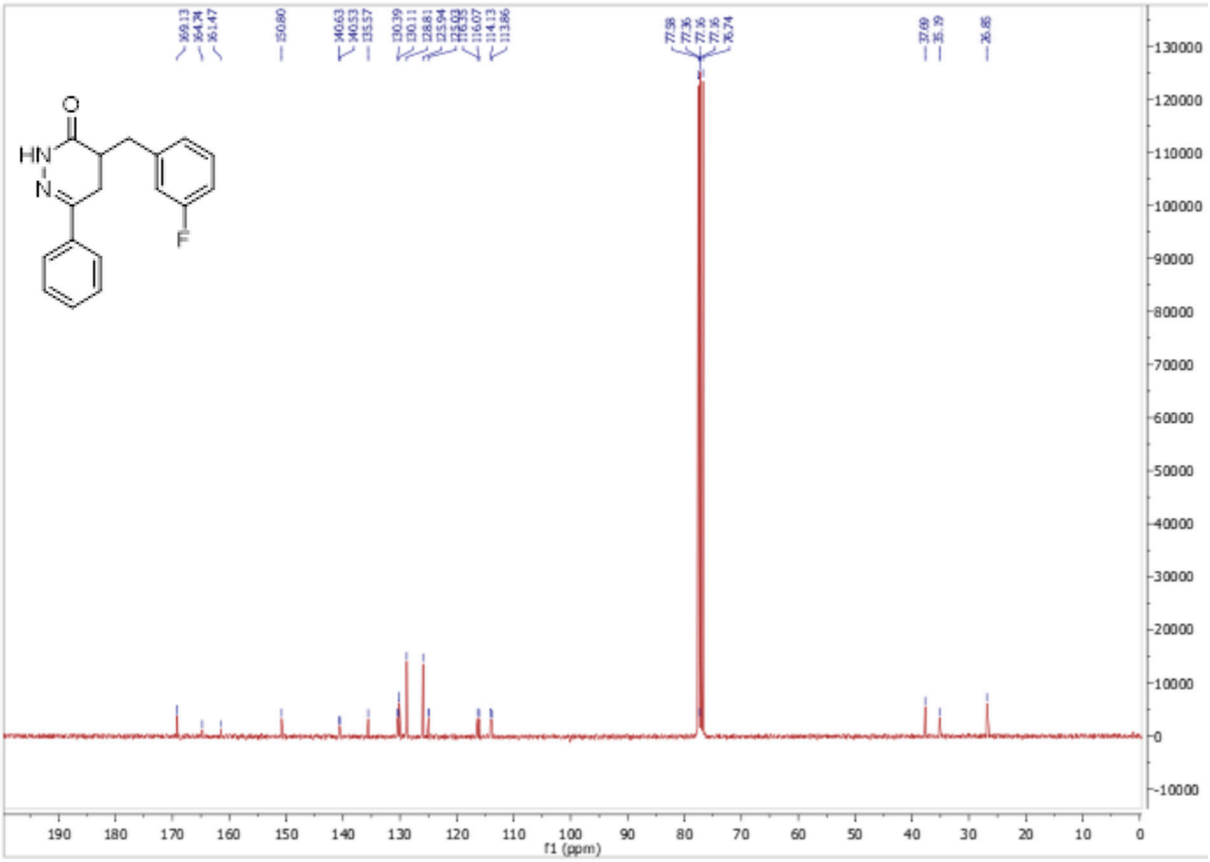

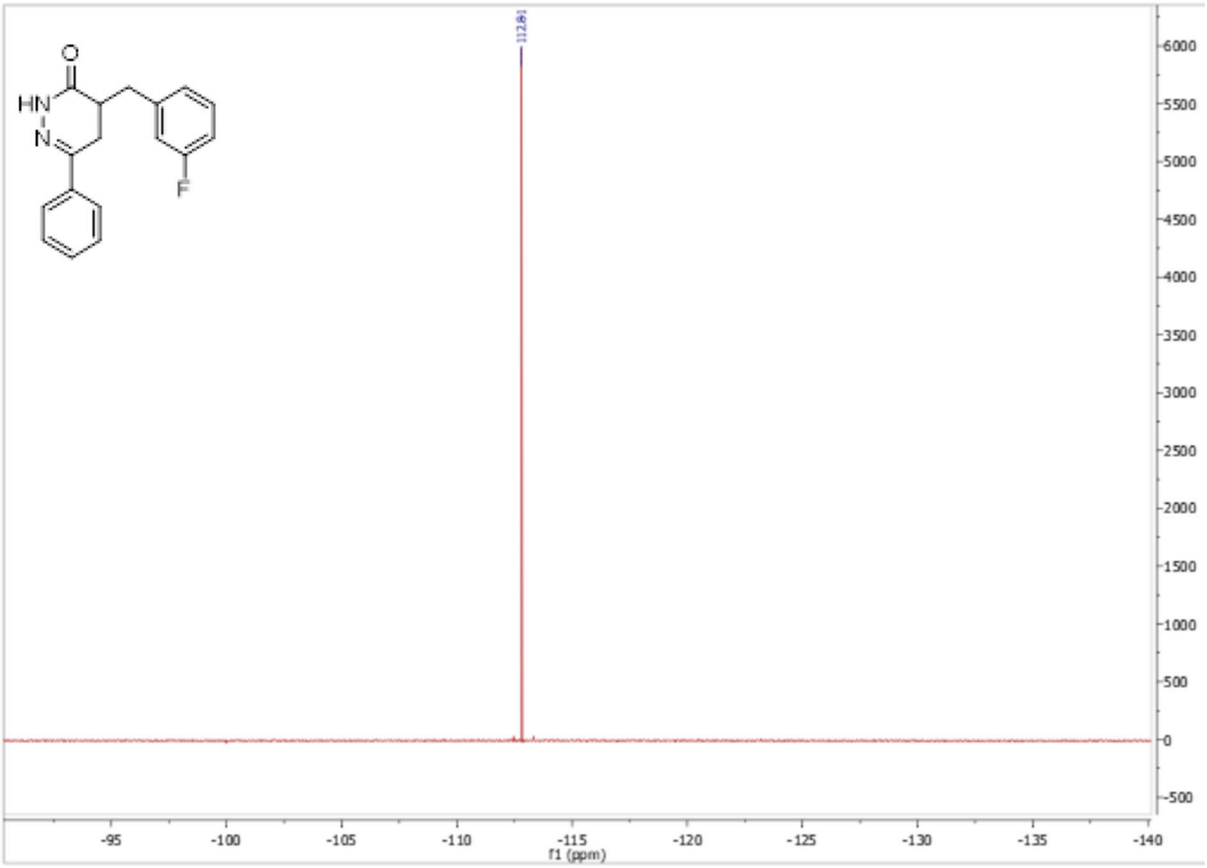

HN
N
O
F
-112.81
6000
5500
5000
4500
4000
3500
3000
2500
2000
1500
1000
500
0
-500
-95
-100
-105
-110
-115
-120
-125
-130
-135
-140
f1 (ppm)

4-benzyl-6-phenyl-4,5-dihydropyridazin-3(*2H*)-one (3b)

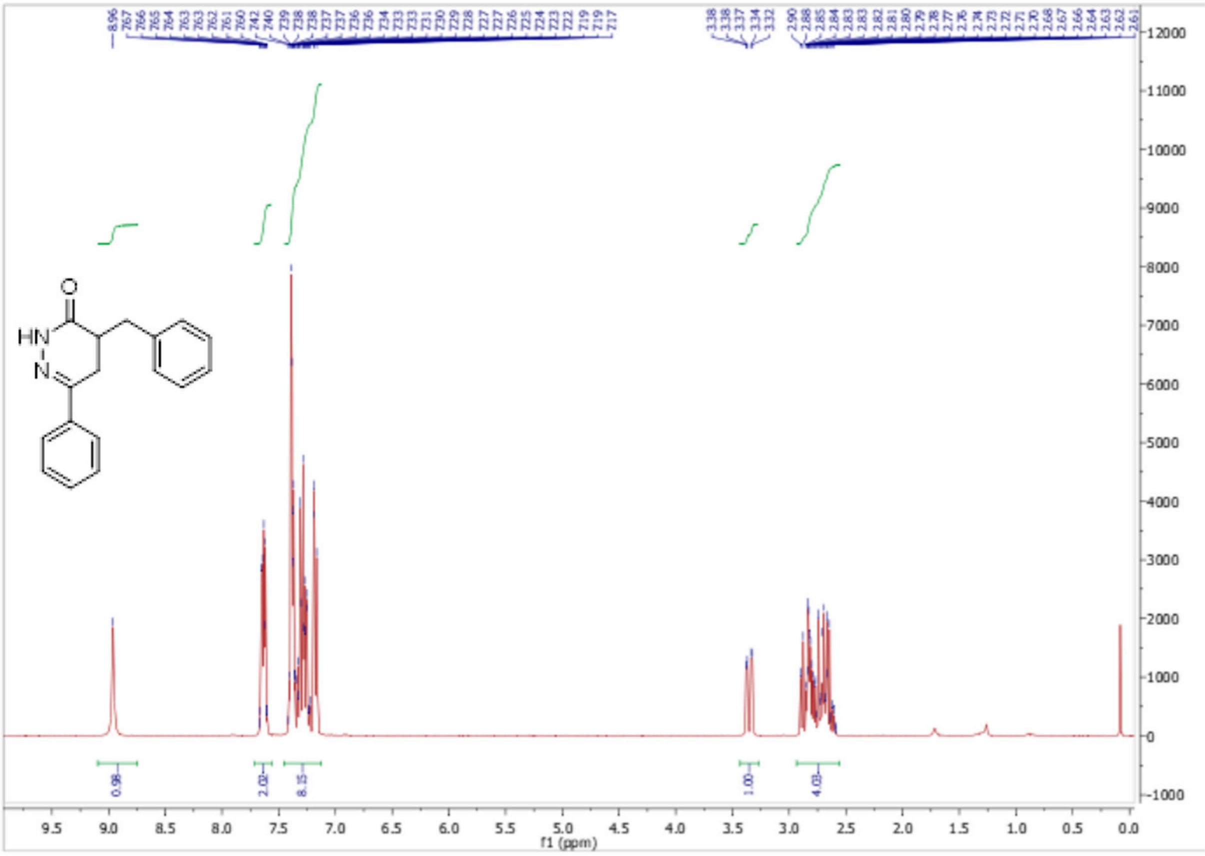

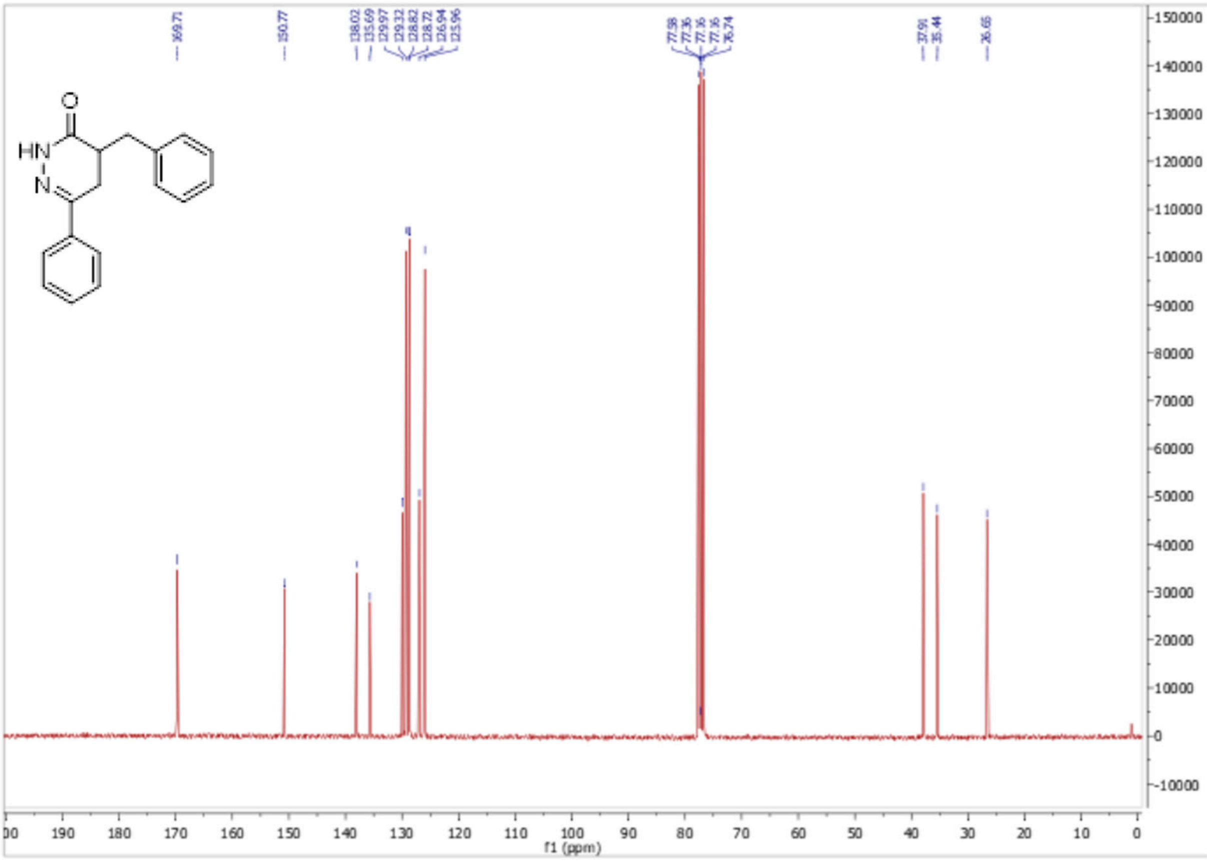

4-(naphthalen-1-ylmethyl)-6-phenyl-4,5-dihydropyridazin-3(*2H*)-one (3c)

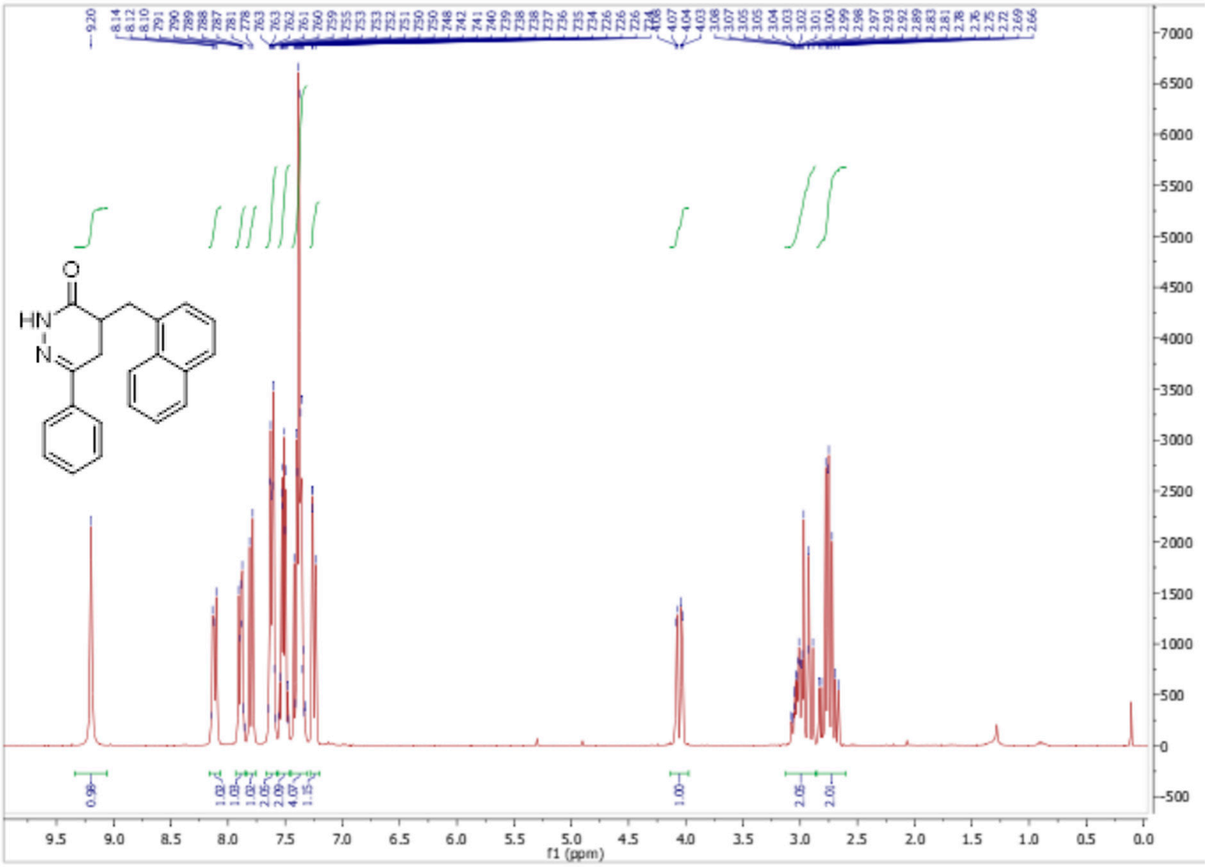

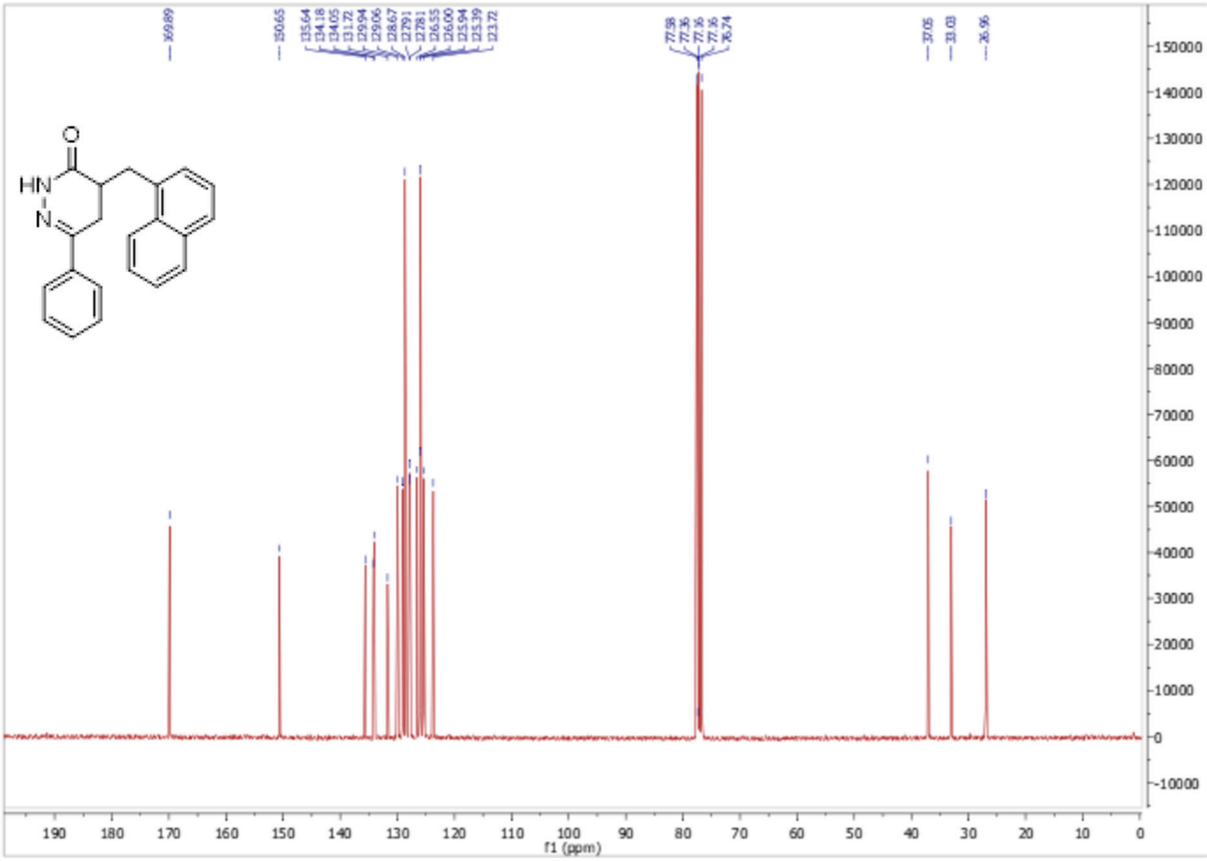

4-(4-fluorobenzyl)-6-phenyl-4,5-dihydropyridazin-3(*2H*)-one (3d)

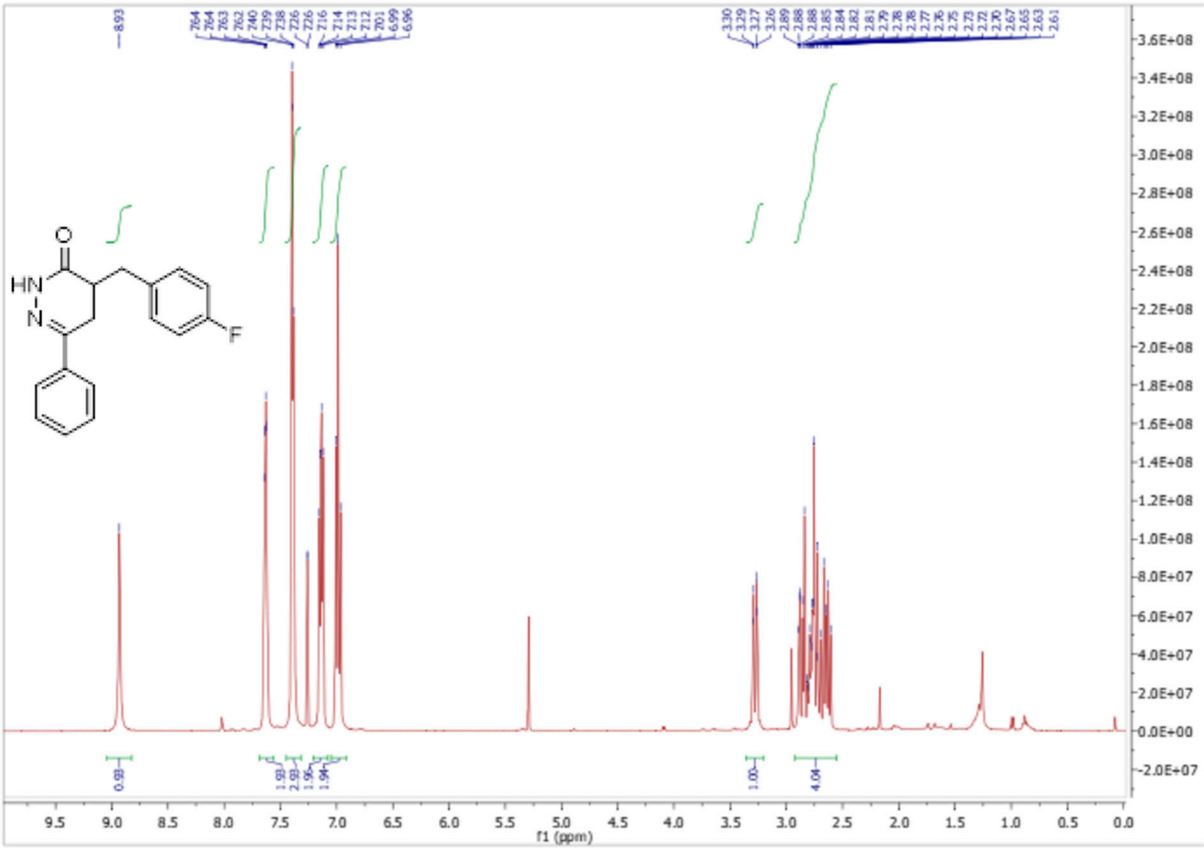

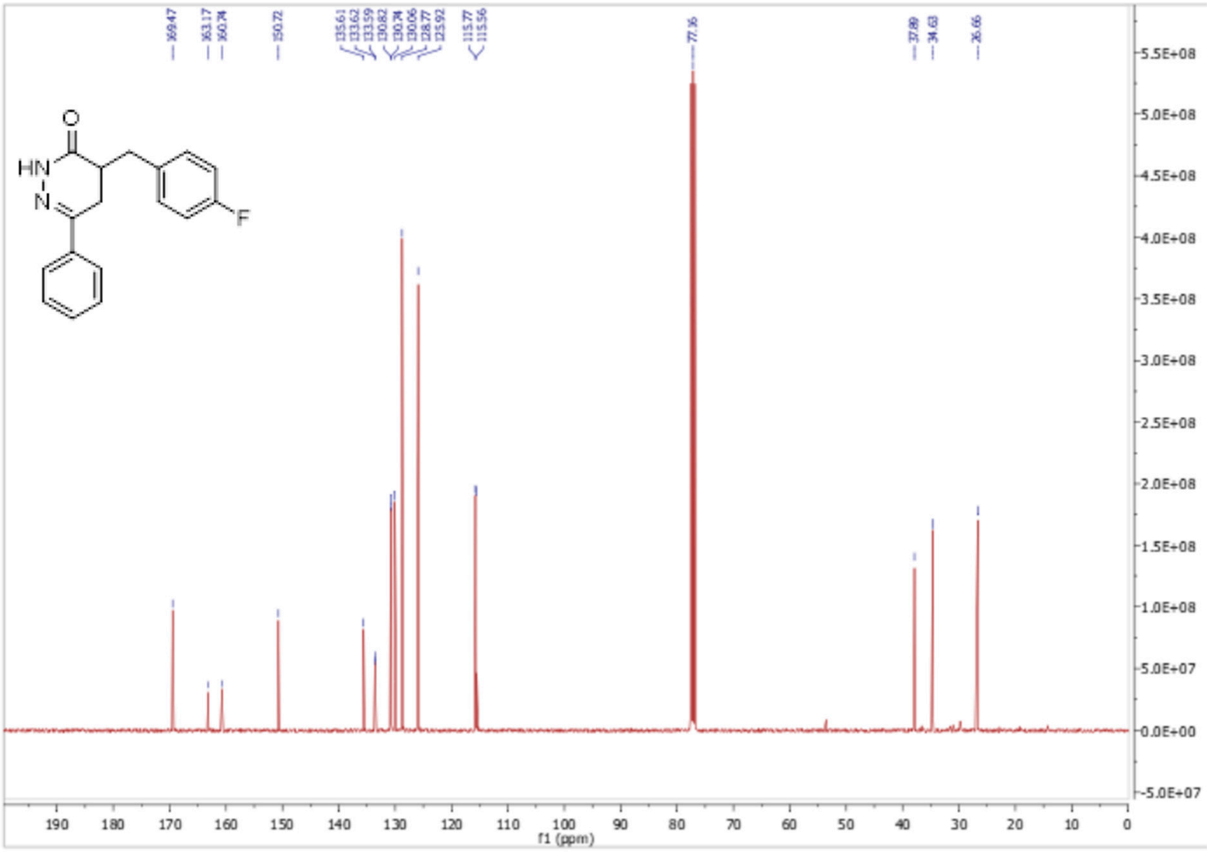

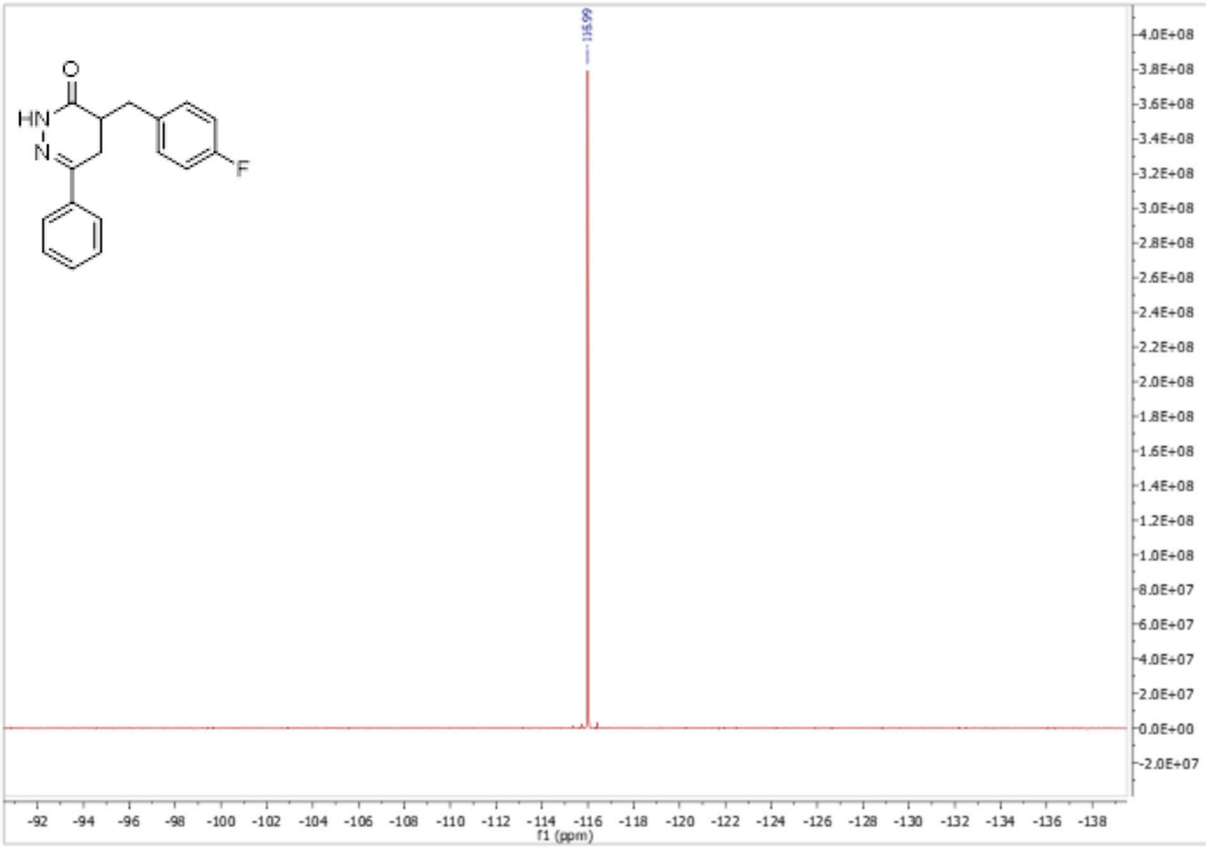
HN
N
O
F
-116.99
4.0E+08
3.8E+08
3.6E+08
3.4E+08
3.2E+08
3.0E+08
2.8E+08
2.6E+08
2.4E+08
2.2E+08
2.0E+08
1.8E+08
1.6E+08
1.4E+08
1.2E+08
1.0E+08
8.0E+07
6.0E+07
4.0E+07
2.0E+07
0.0E+00
-2.0E+07
-92 -94 -96 -98 -100 -102 -104 -106 -108 -110 -112 -114 -116 -118 -120 -122 -124 -126 -128 -130 -132 -134 -136 -138
f1 (ppm)

4-(4-bromobenzyl)-6-phenyl-4,5-dihydropyridazin-3(2H)-one (3e)

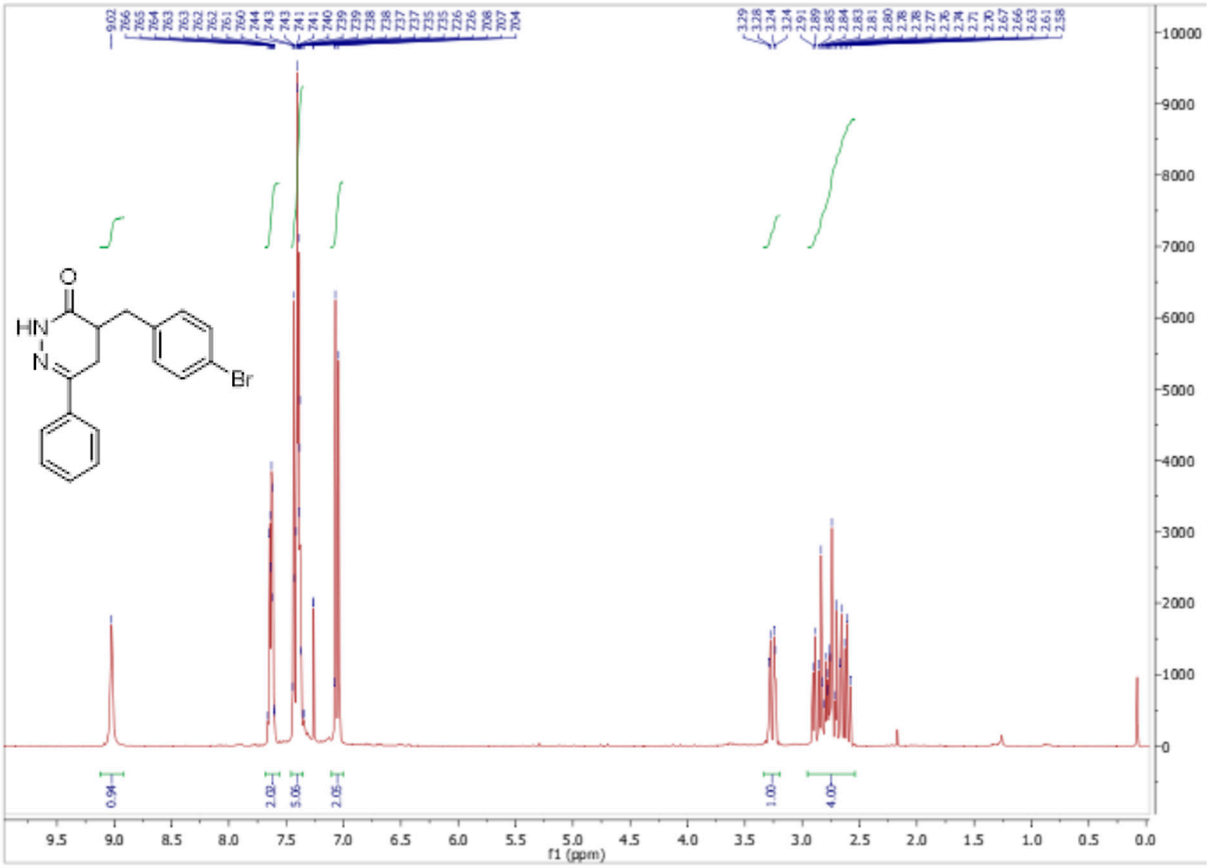

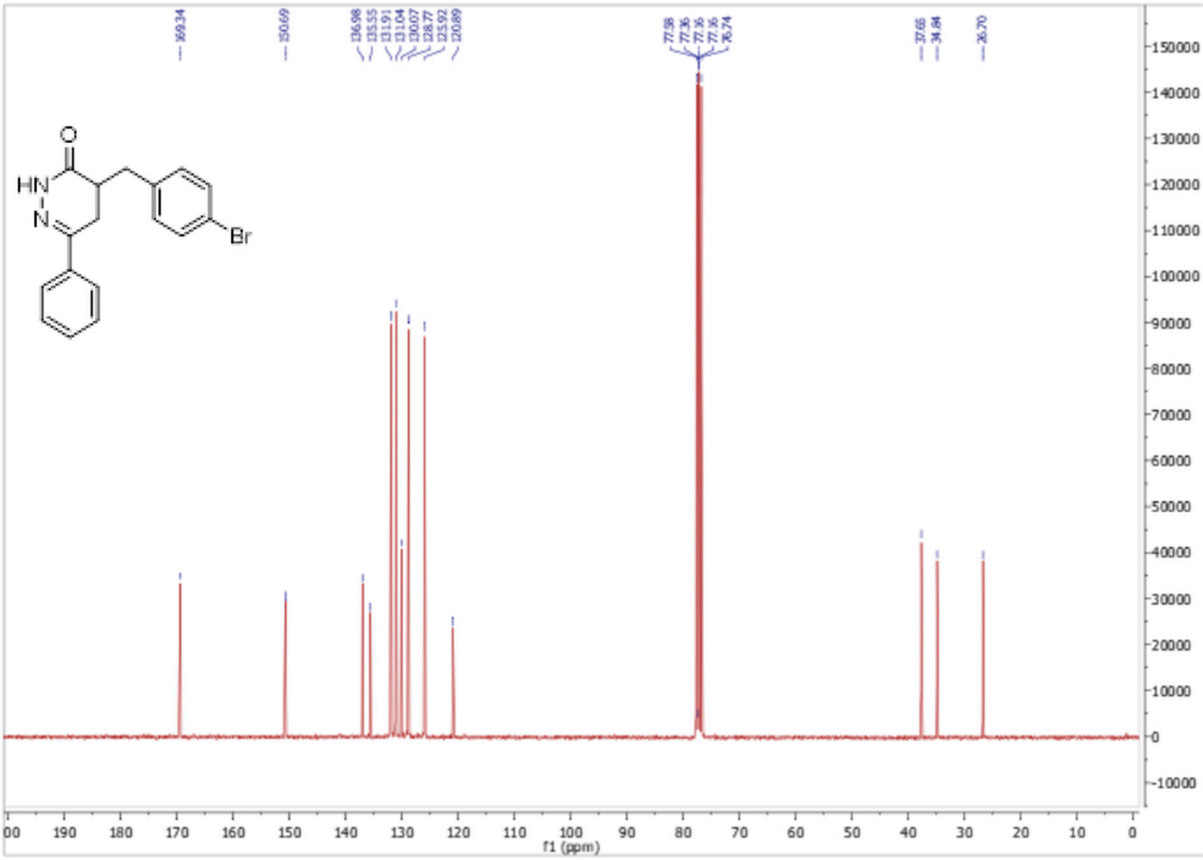

4-(4-chlorobenzyl)-6-phenyl-4,5-dihydropyridazin-3(2H)-one (3f)

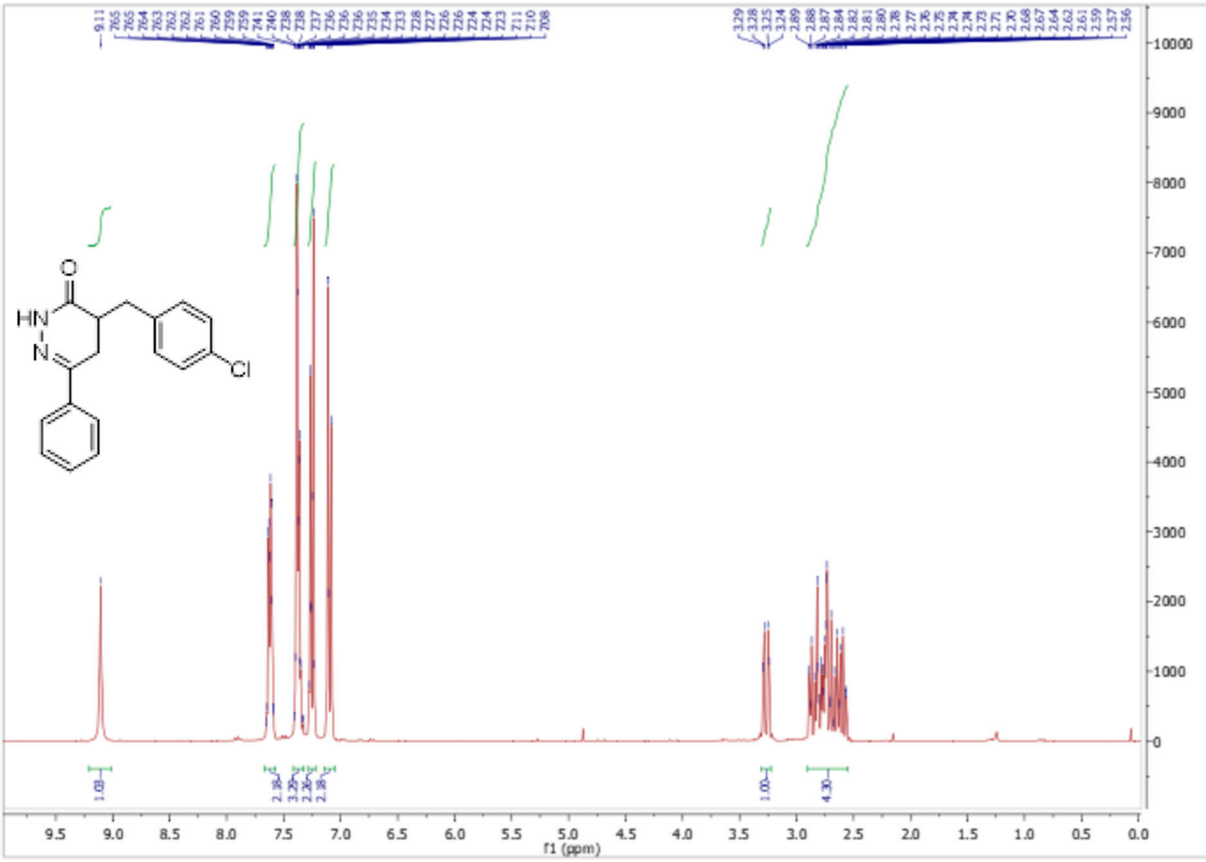

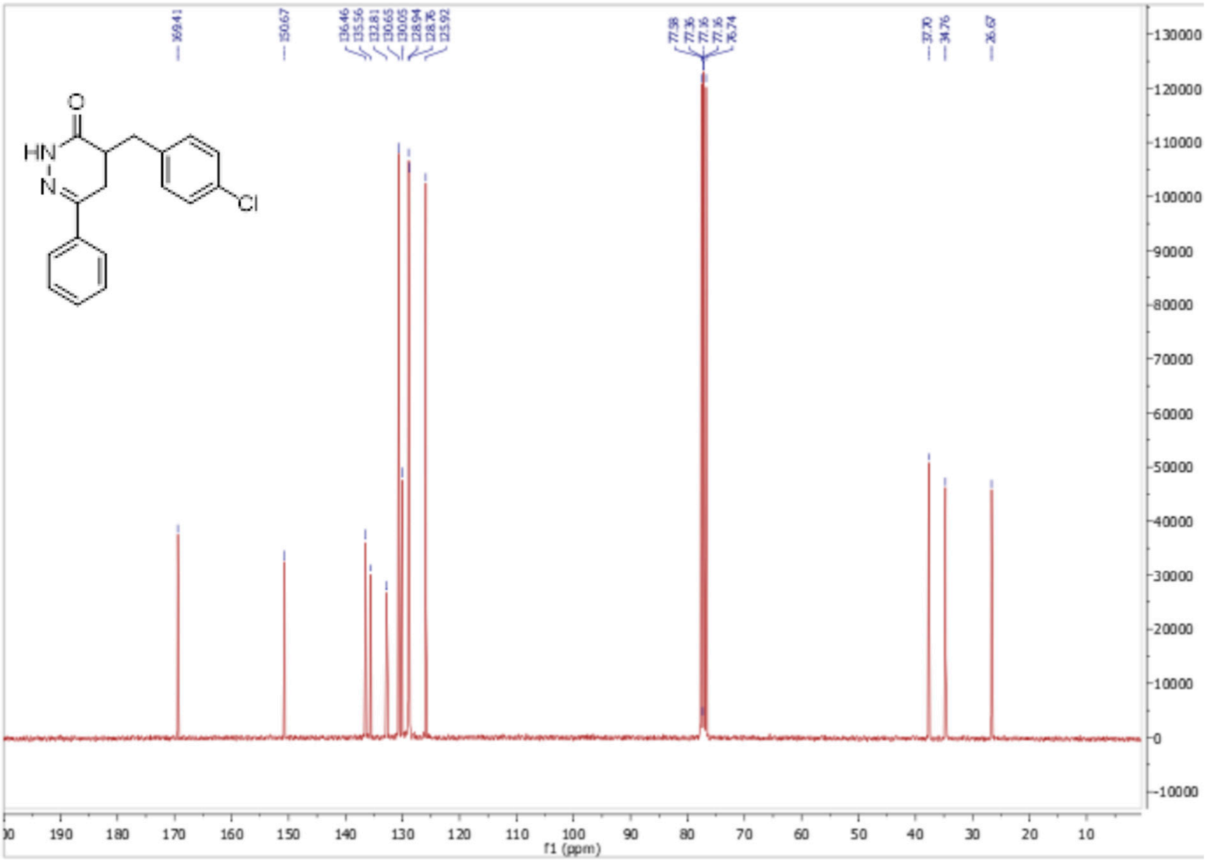

2-((3-oxo-6-phenyl-2,3,4,5-tetrahydropyridazin-4-yl)methyl)benzonitrile (3g)

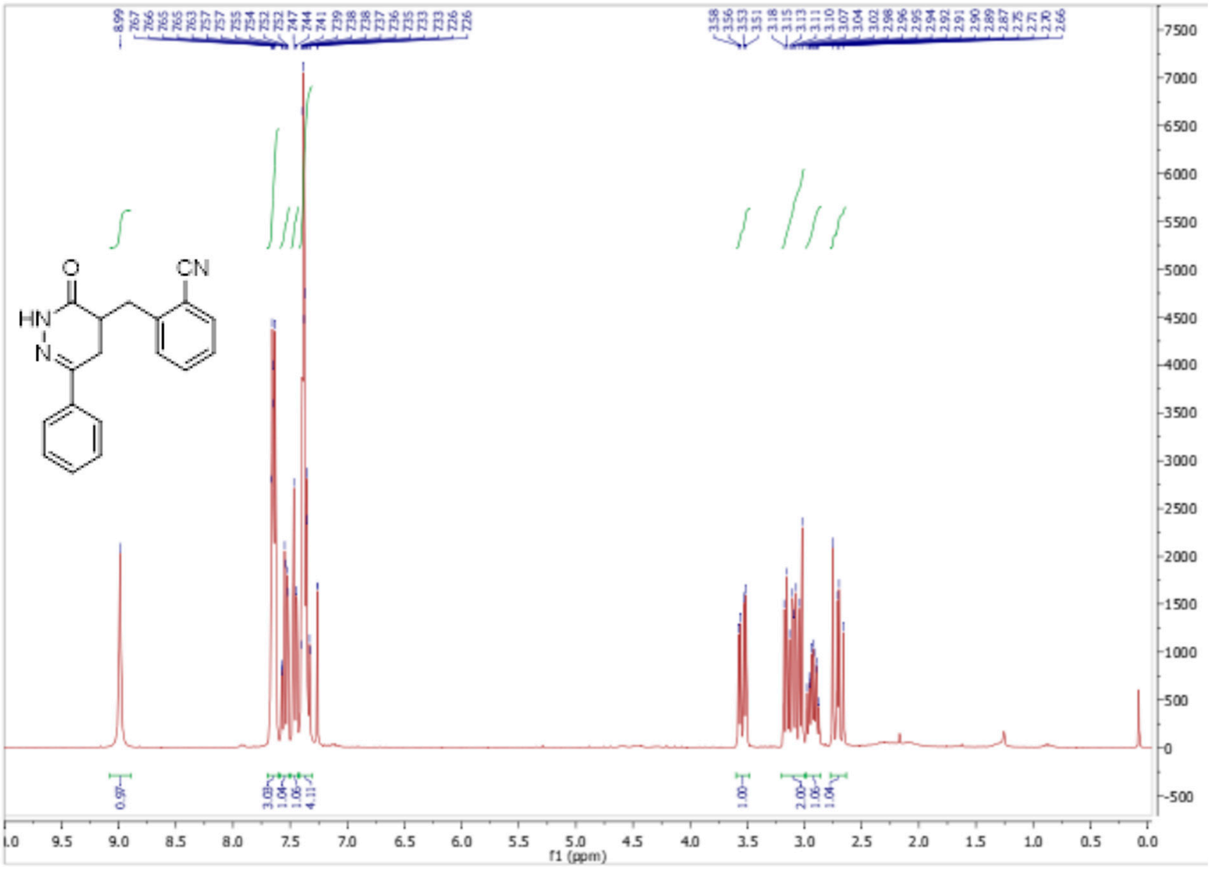

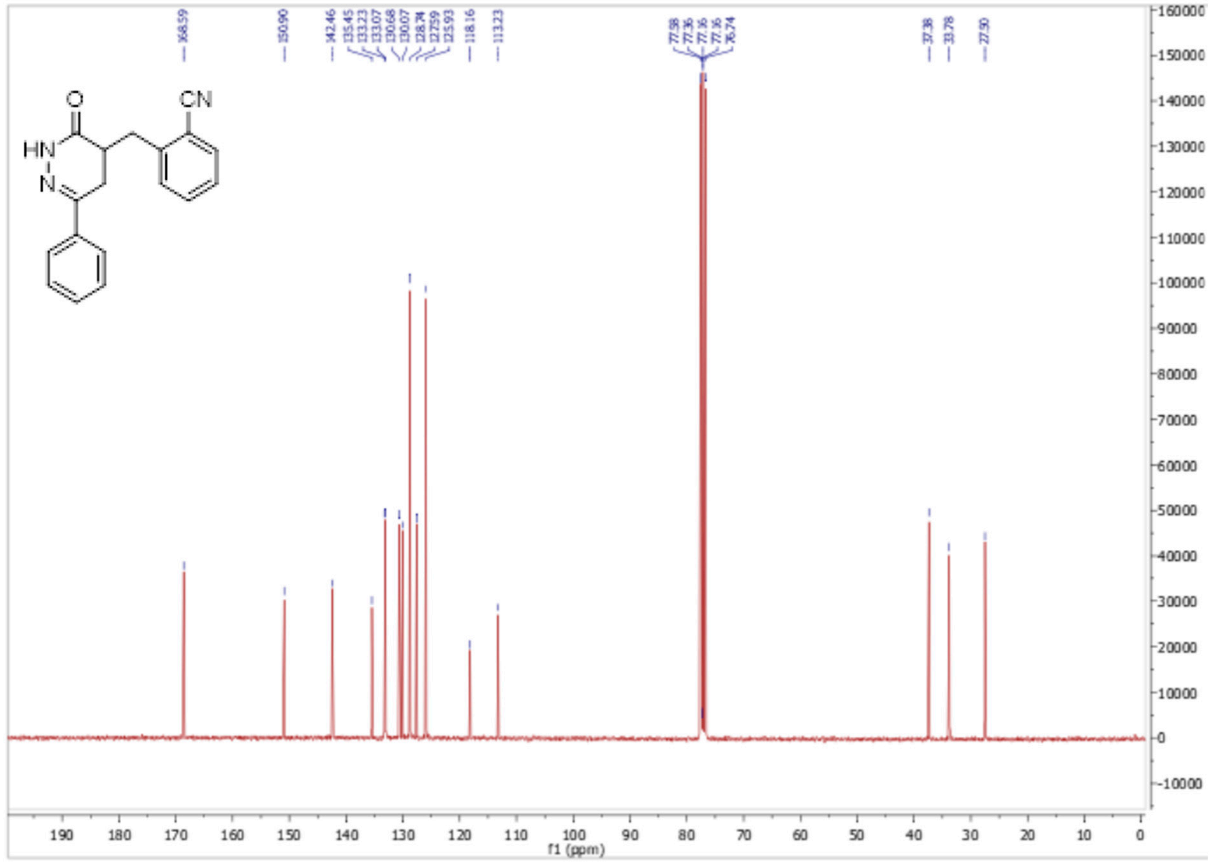

4-(2-methoxybenzyl)-6-phenyl-4,5-dihydropyridazin-3(*2H*)-one (3h)

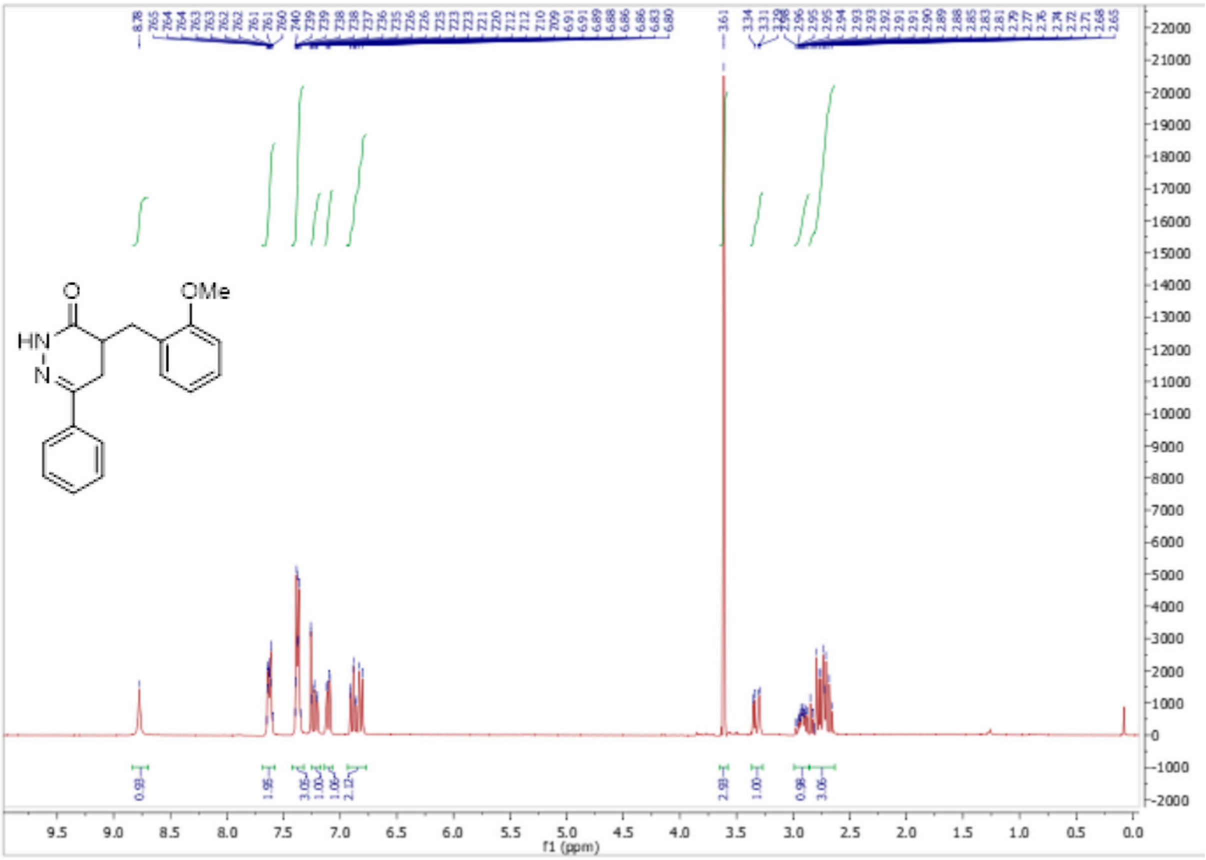

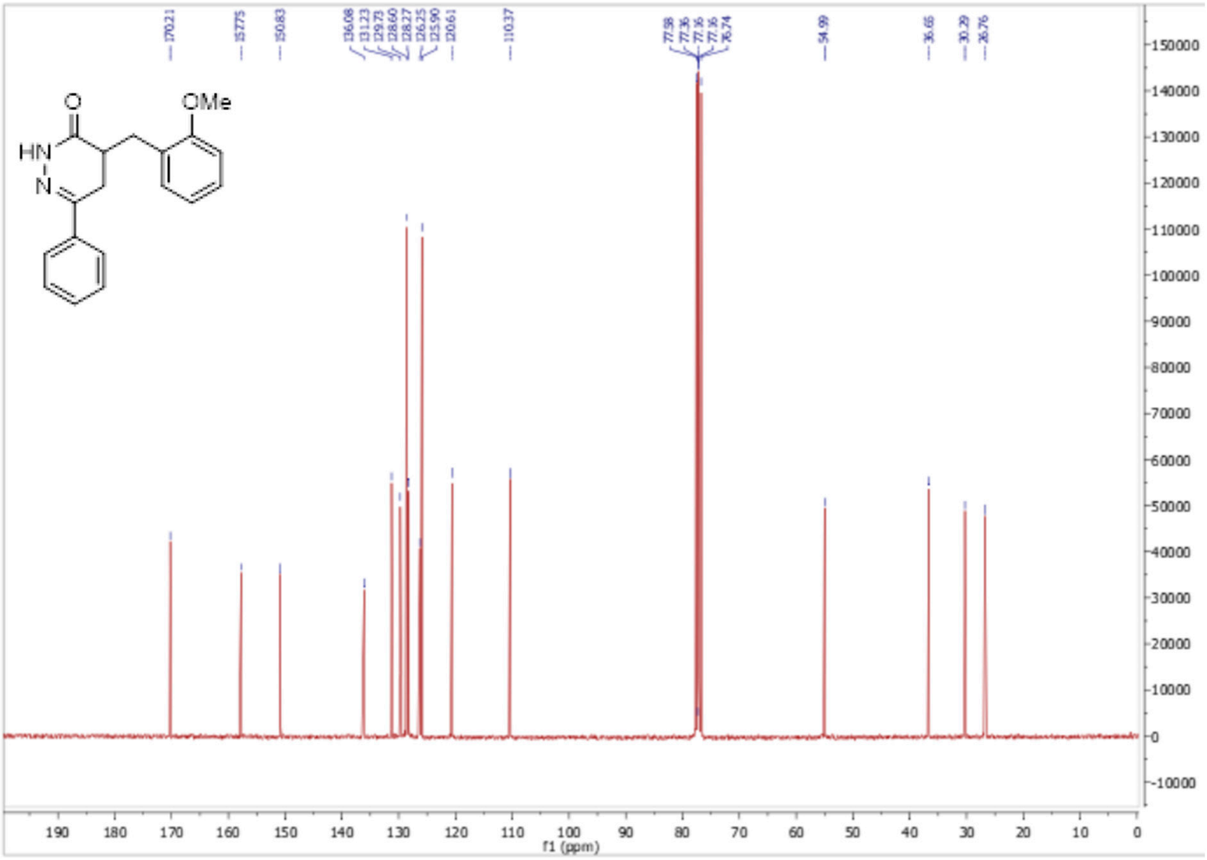

4-(4-methylbenzyl)-6-phenyl-4,5-dihydropyridazin-3(*2H*)-one (3i)

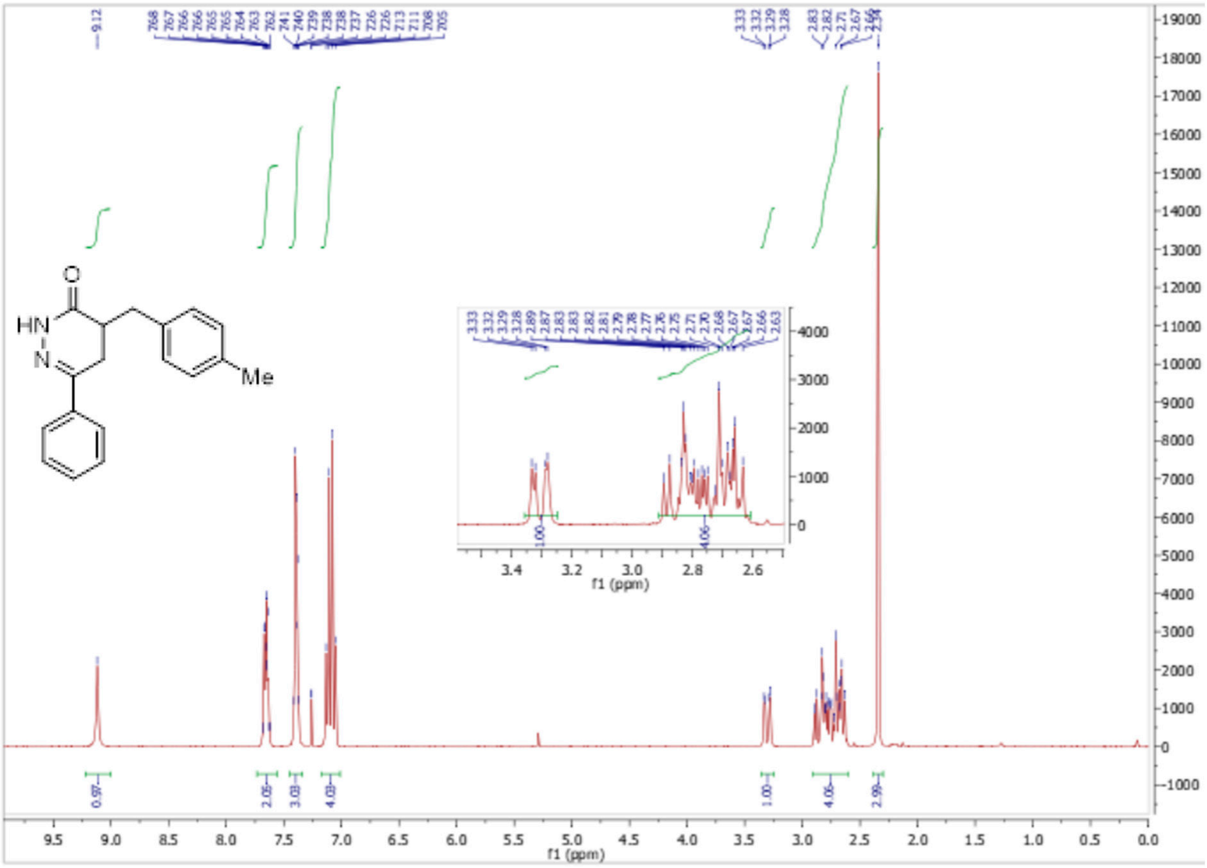

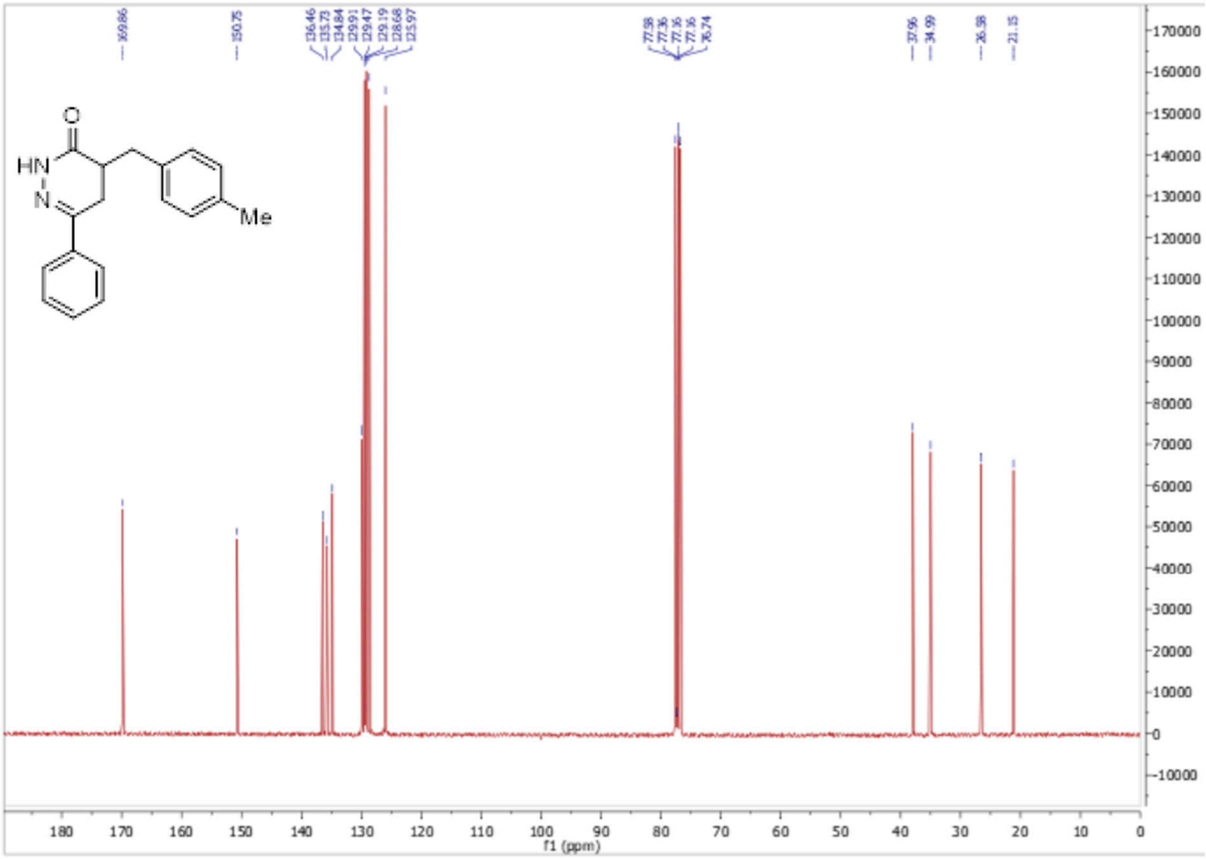

4-(2,5-dimethylbenzyl)-6-phenyl-4,5-dihydropyridazin-3(2H)-one (3j)

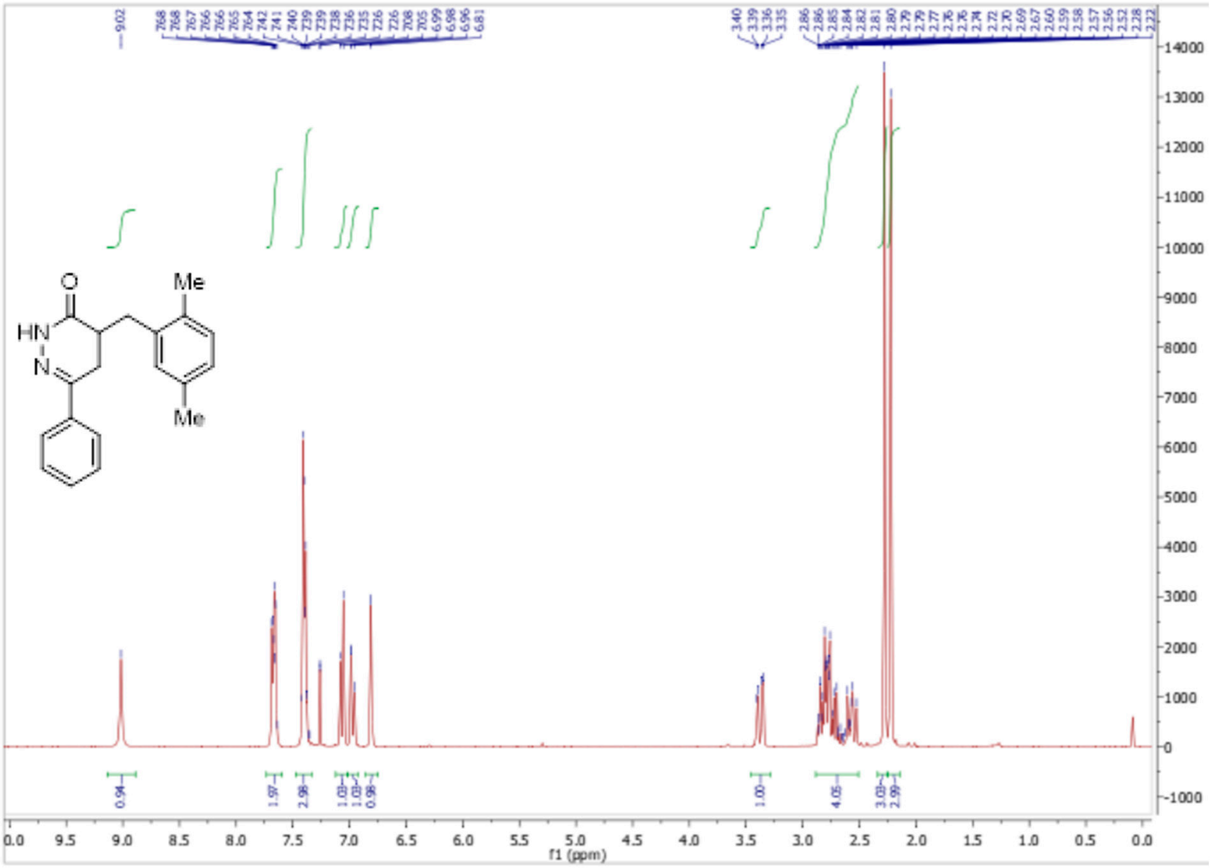

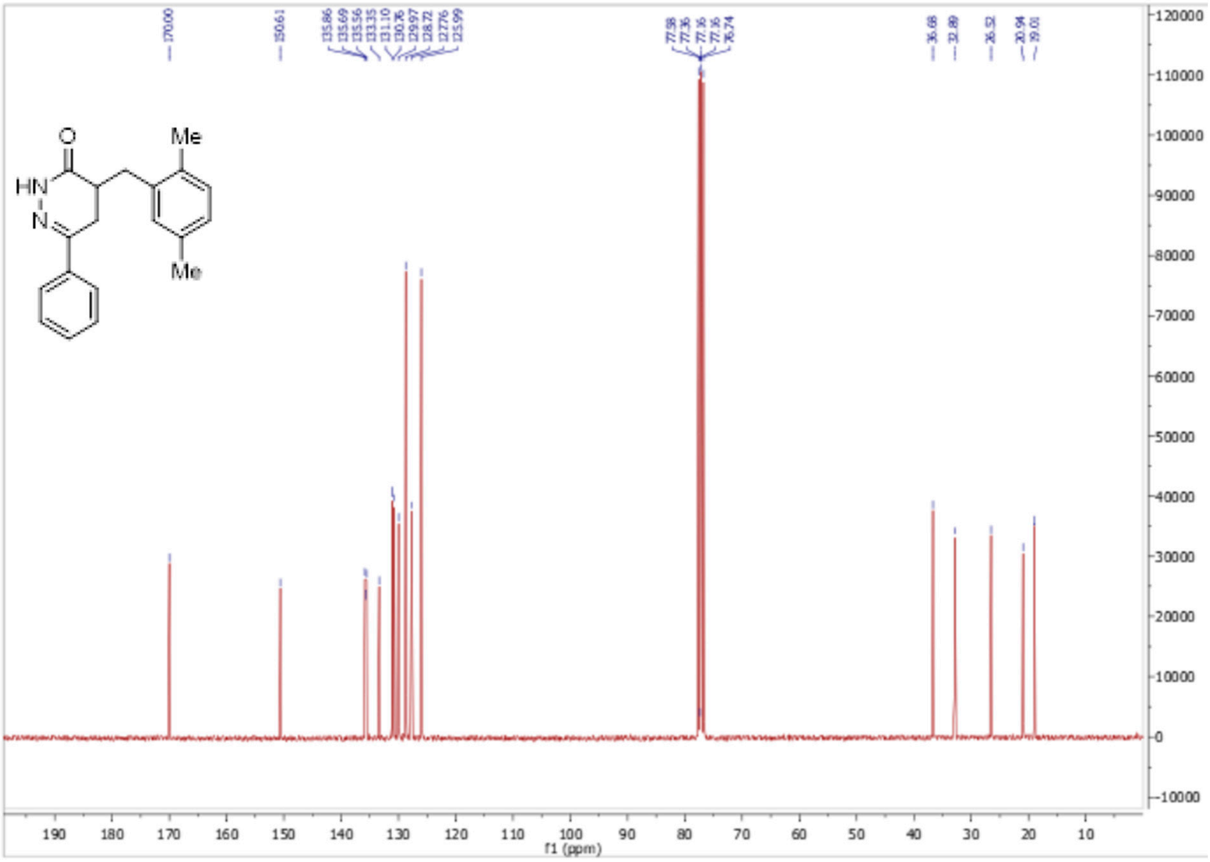

6-phenyl-4-(thiophen-2-ylmethyl)-4,5-dihydropyridazin-3(*2H*)-one (3k)

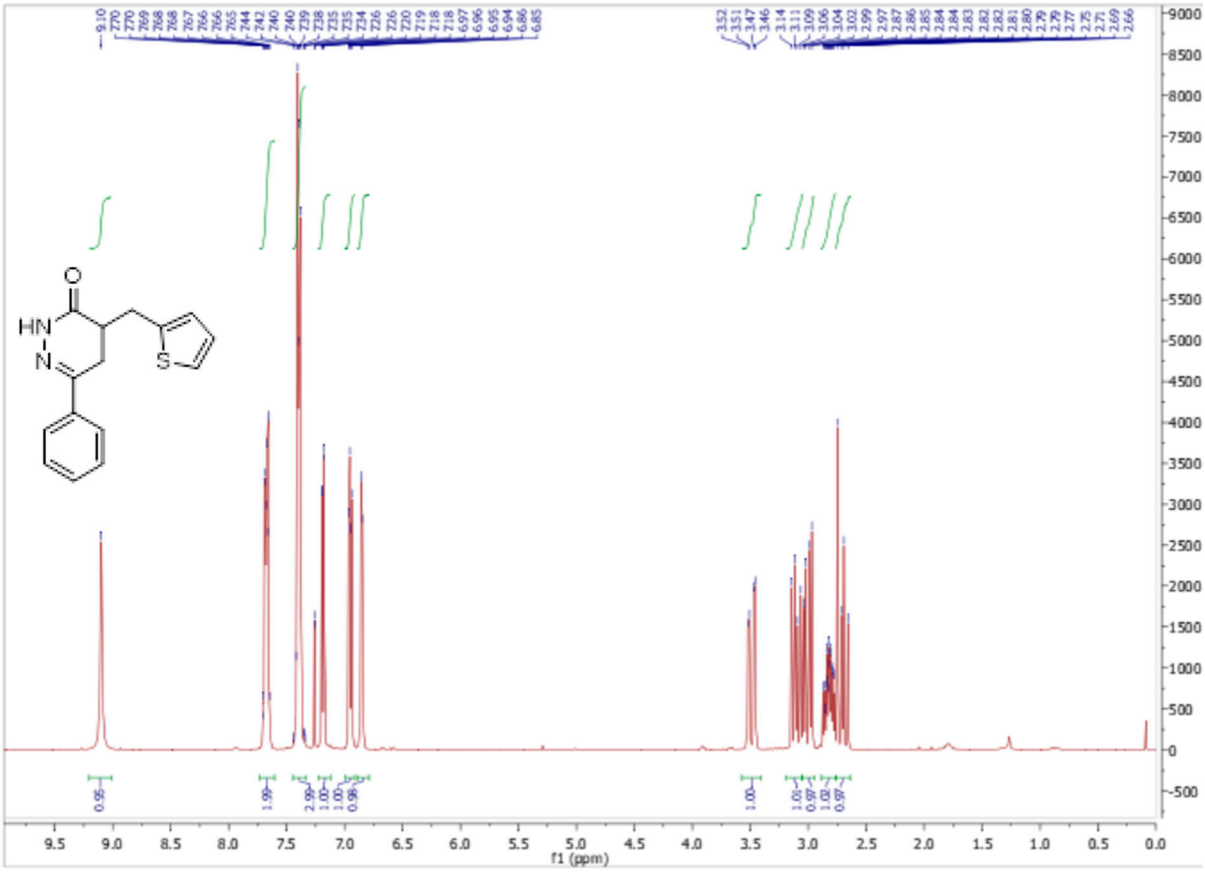

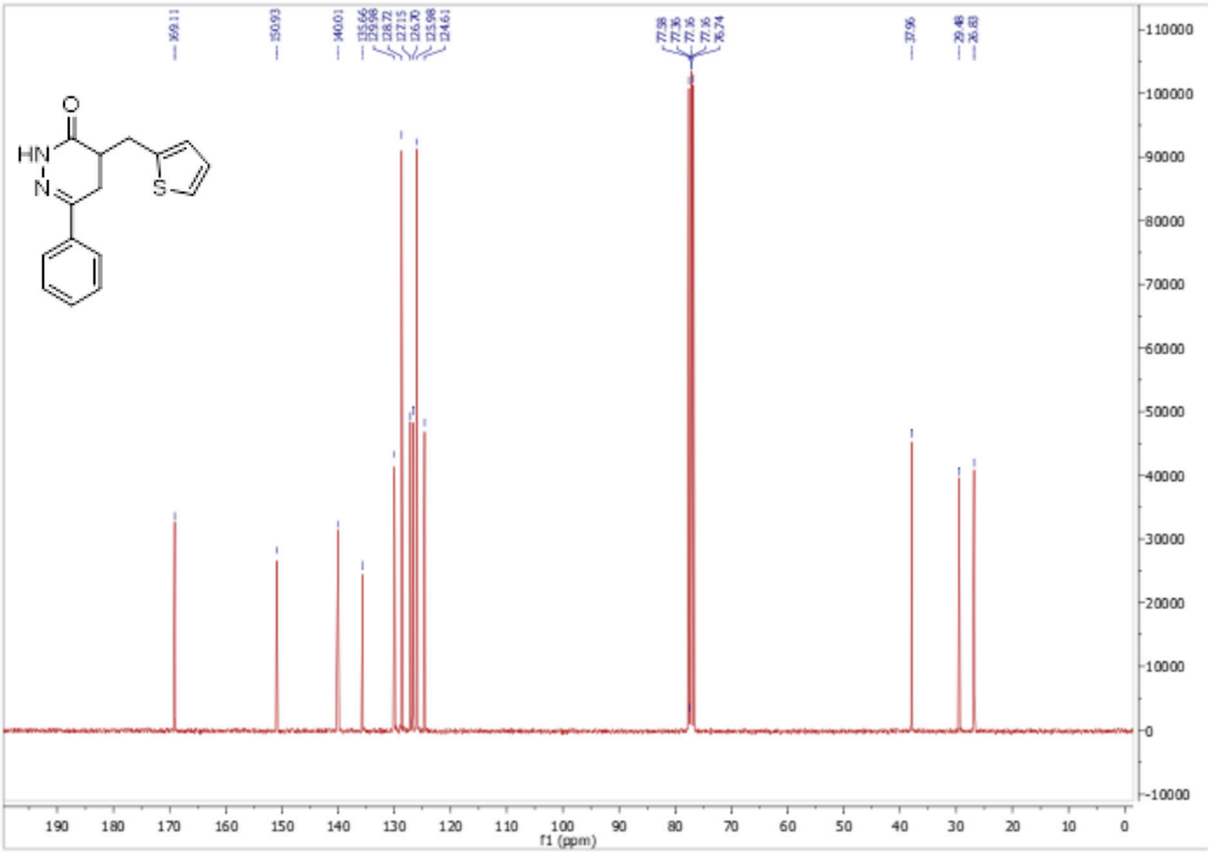

4-isobutyl-6-phenyl-4,5-dihydropyridazin-3(2H)-one (3l)

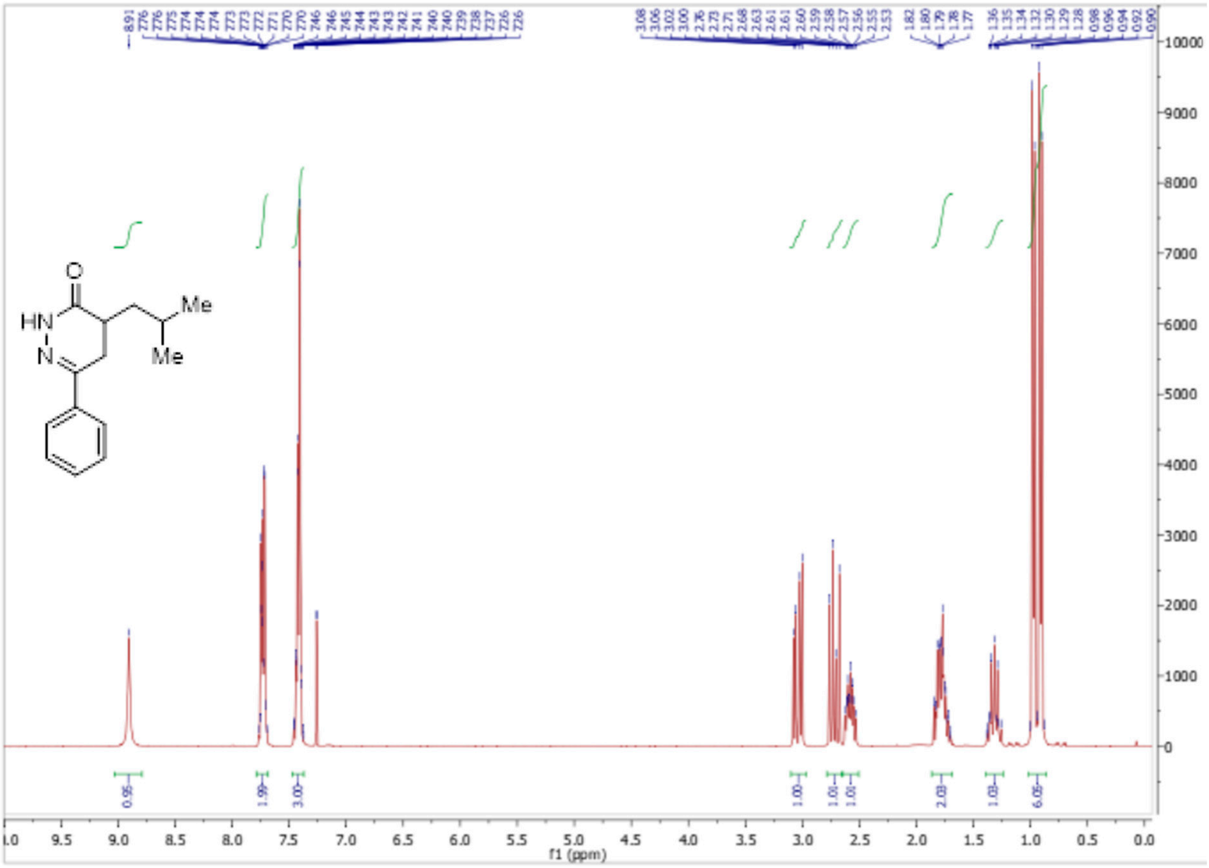

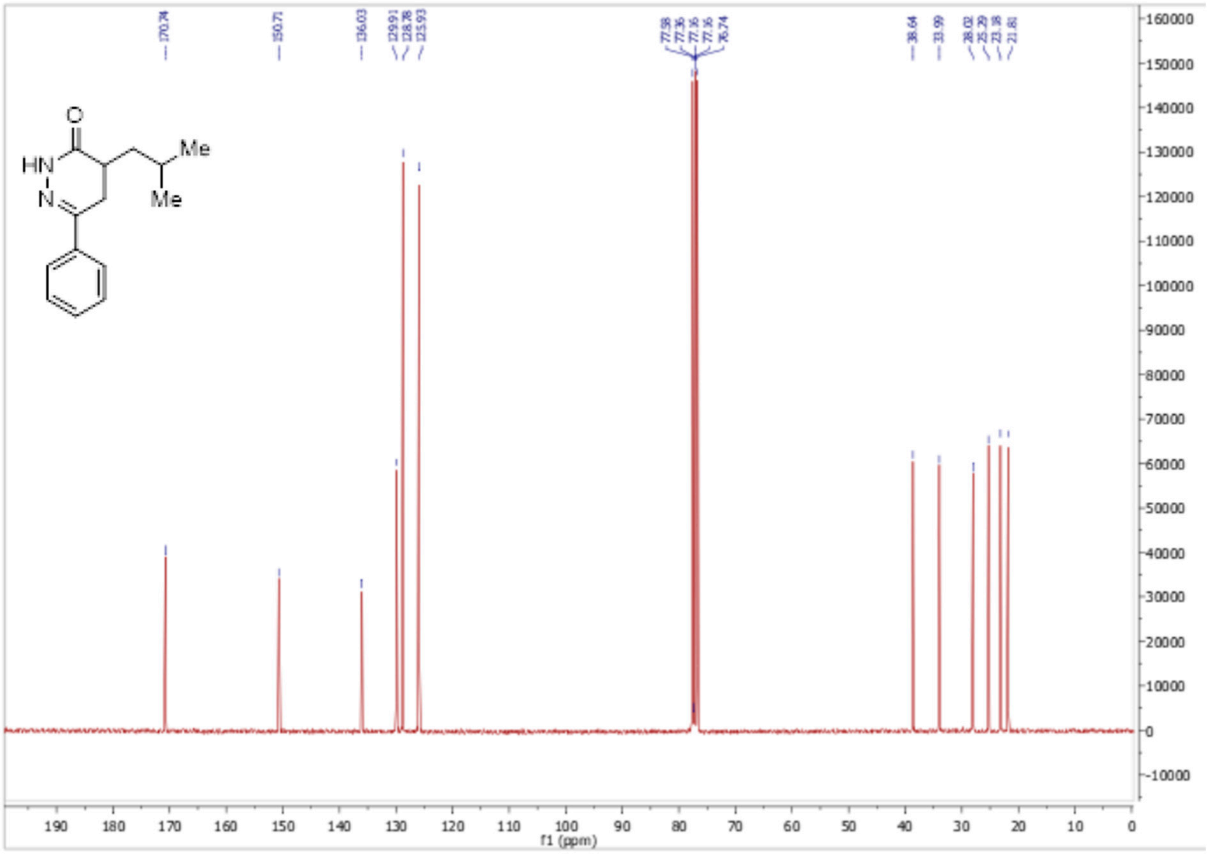

6-phenyl-4-(3-phenylpropyl)-4,5-dihydropyridazin-3(*2H*)-one (**3m**)

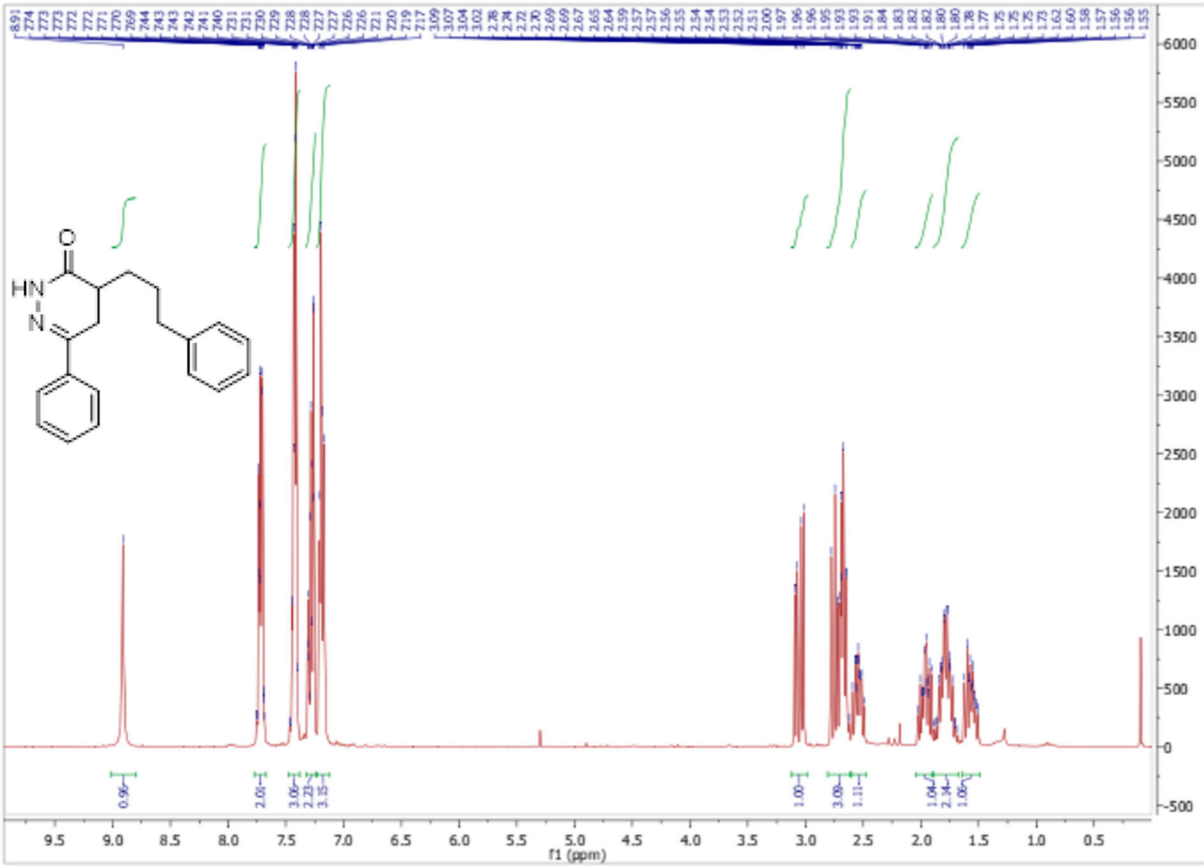

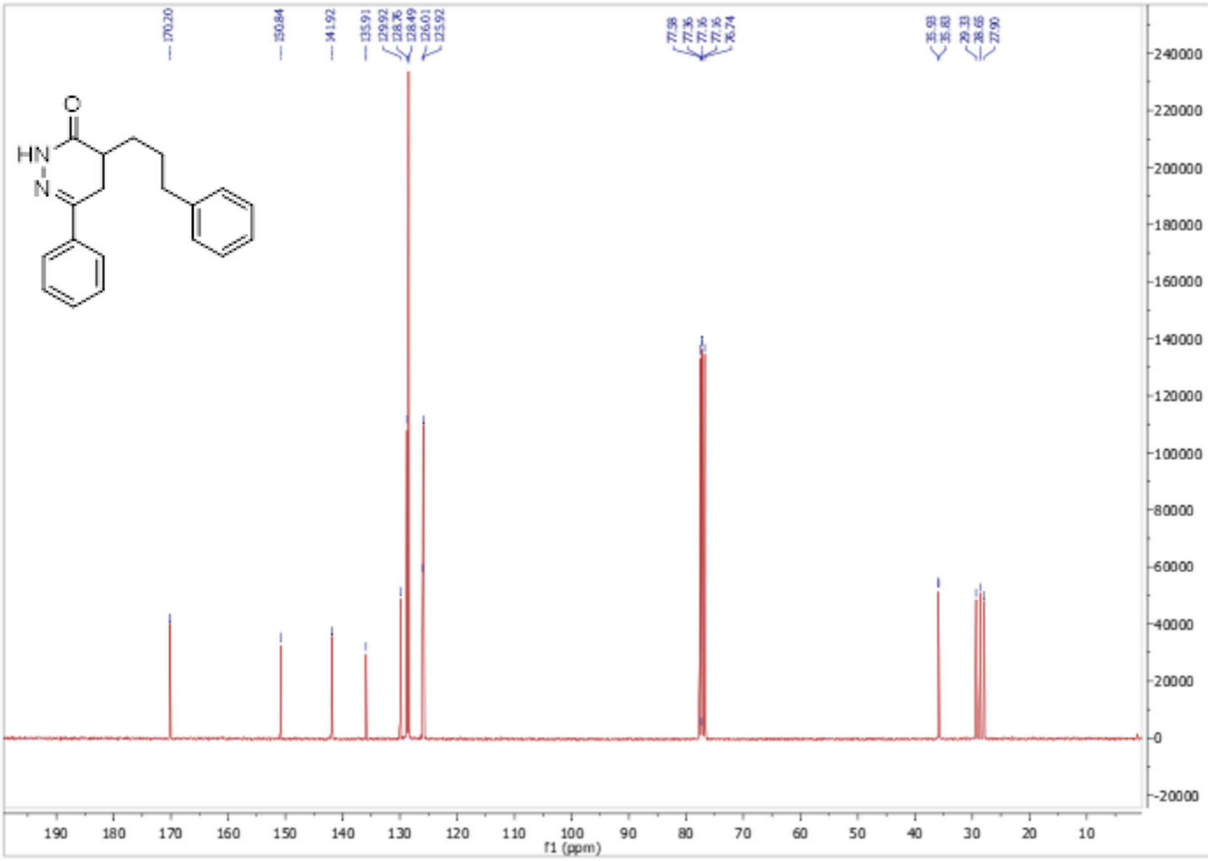

4,6-diphenyl-4,5-dihydropyridazin-3(*2H*)-one (3n)

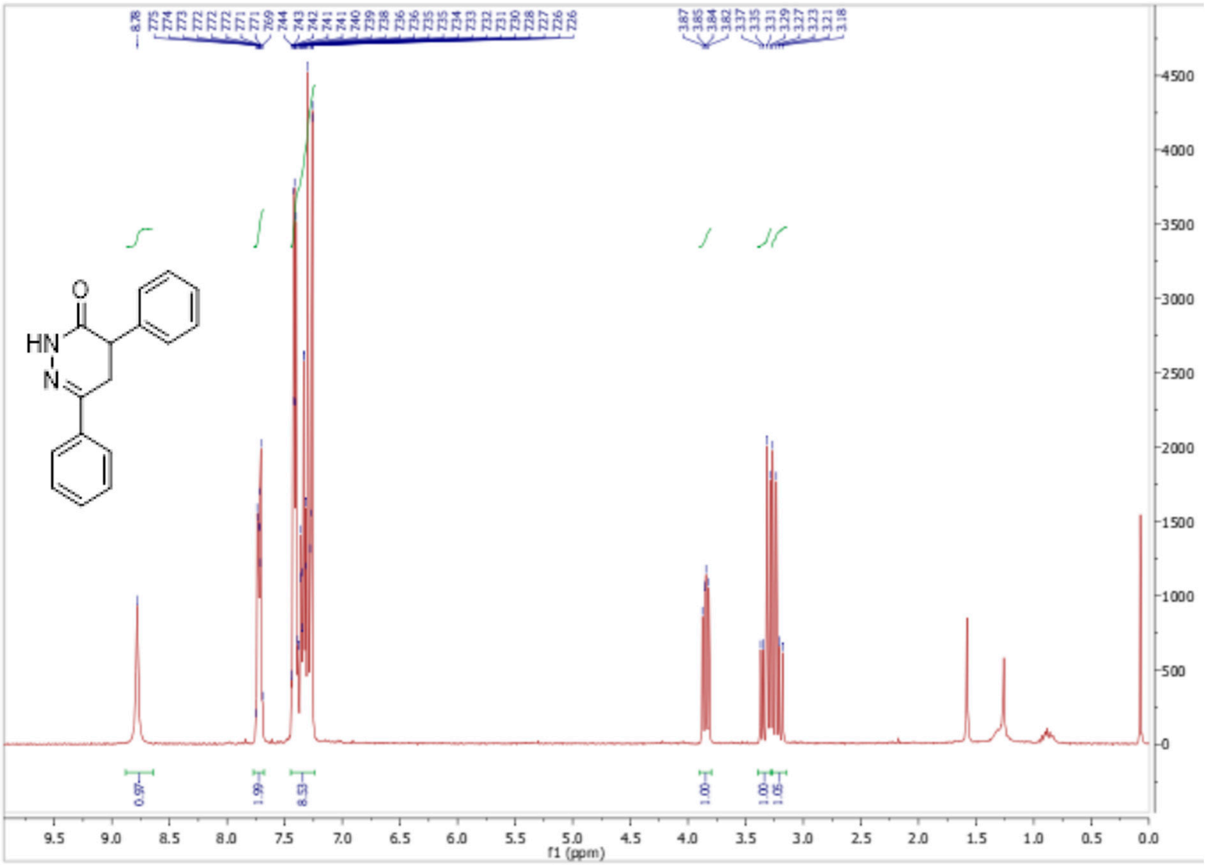

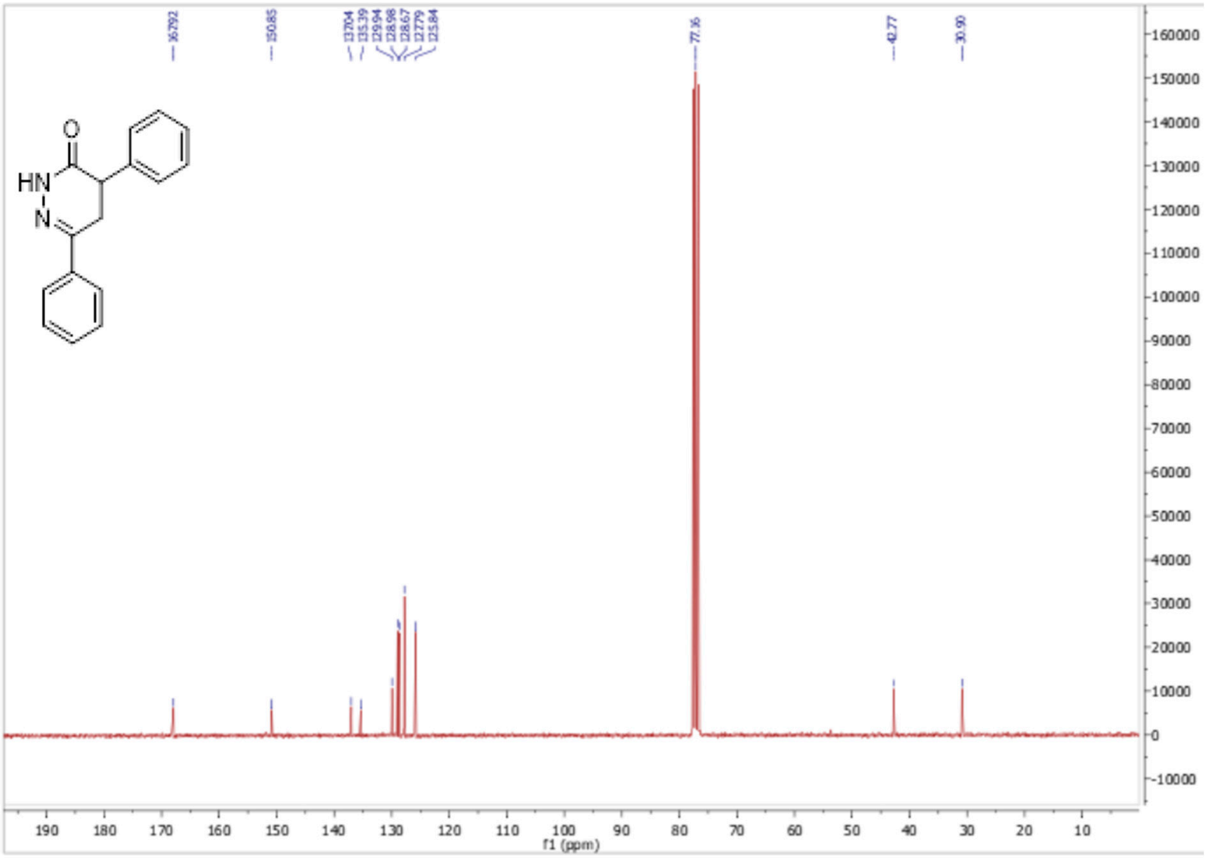

4-(3-fluorobenzyl)-4,5-dihydropyridazin-3(*2H*)-one (6)

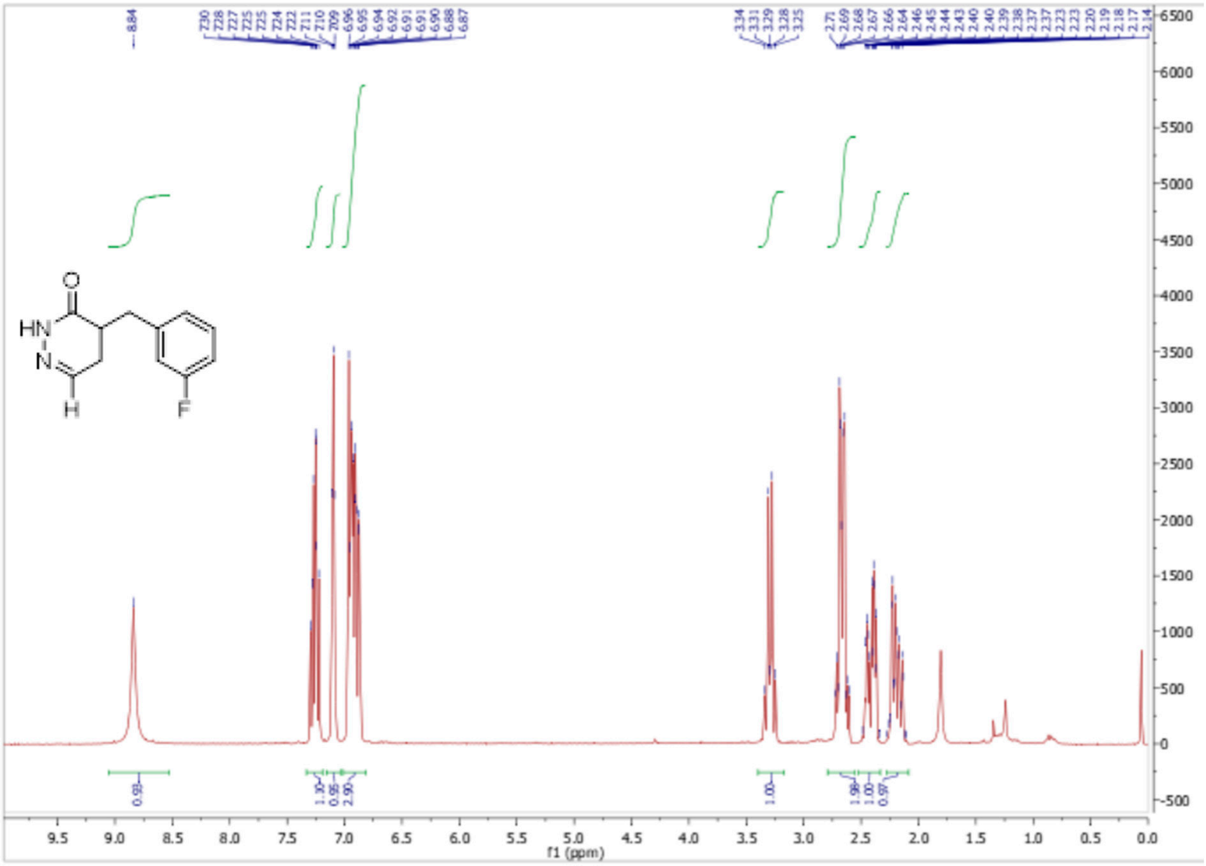

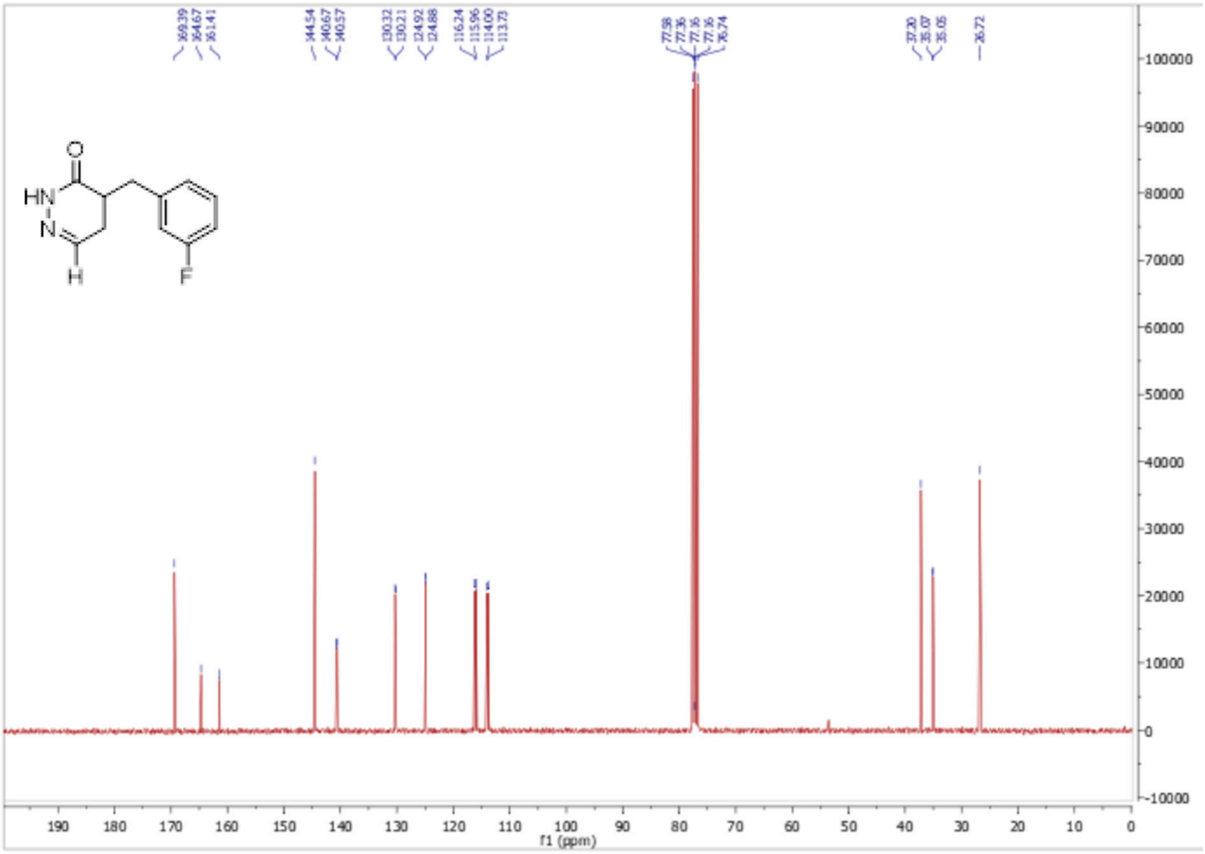

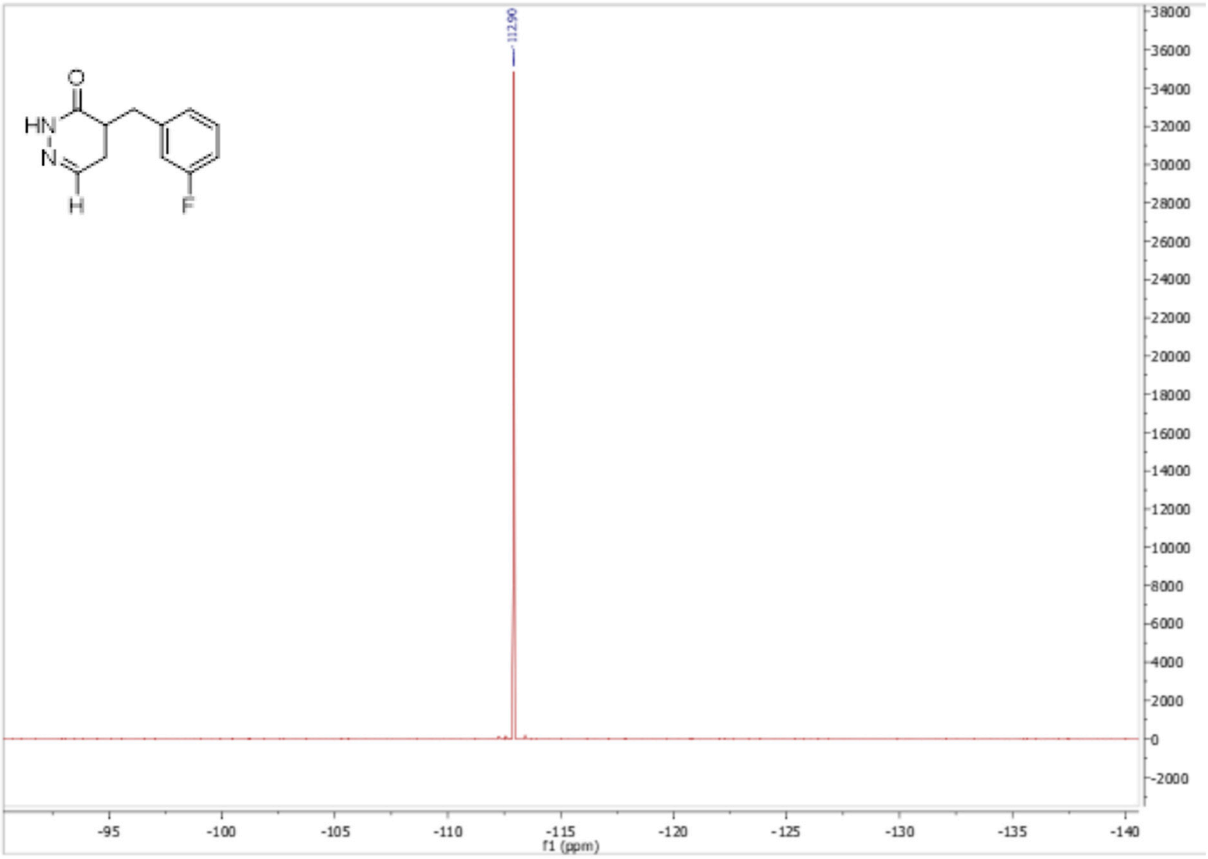

-112.90
HN
N
O
H
F
f1 (ppm)

IV. 1. 4. NMR spectra of *N*-Boc pyridazinones

tert-butyl 5-(3-fluorobenzyl)-6-oxo-3-phenyl-5,6-dihydropyridazine-1(_4H_)-carboxylate (7a)

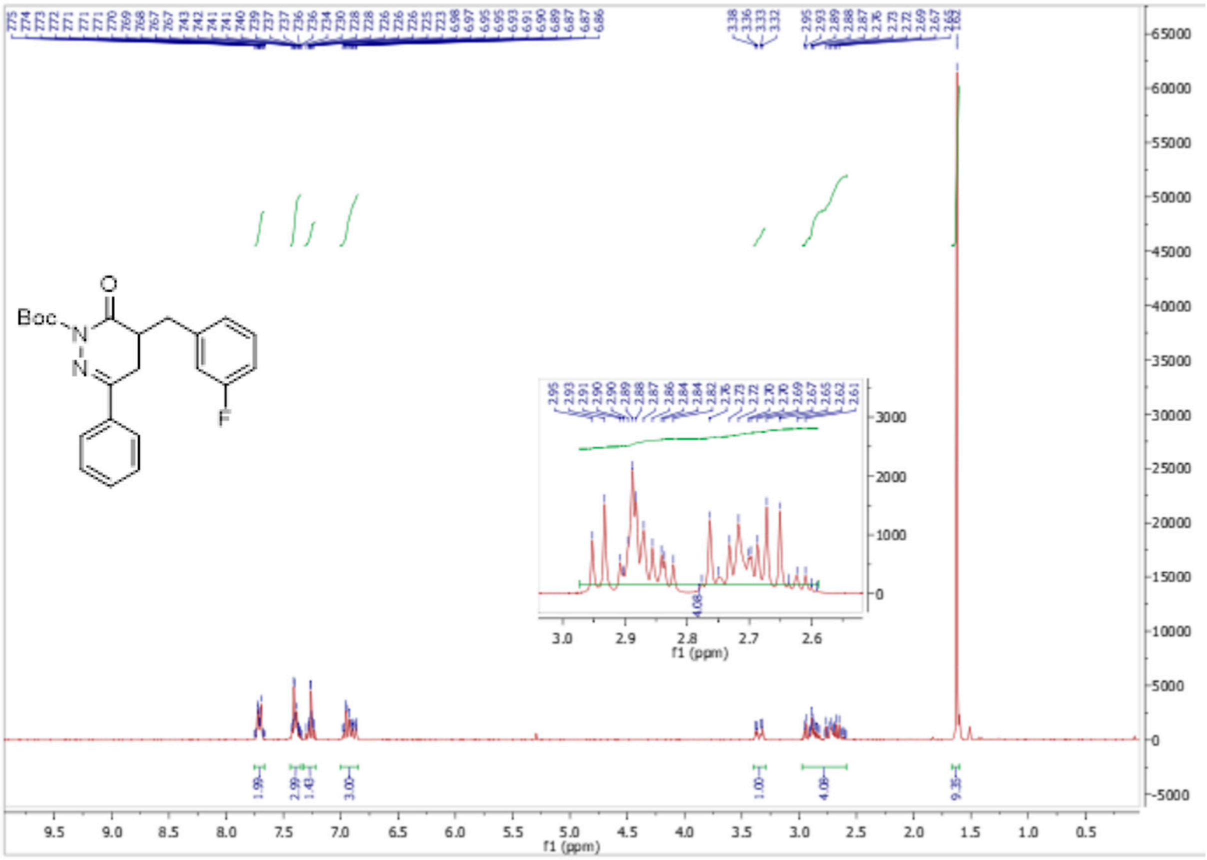

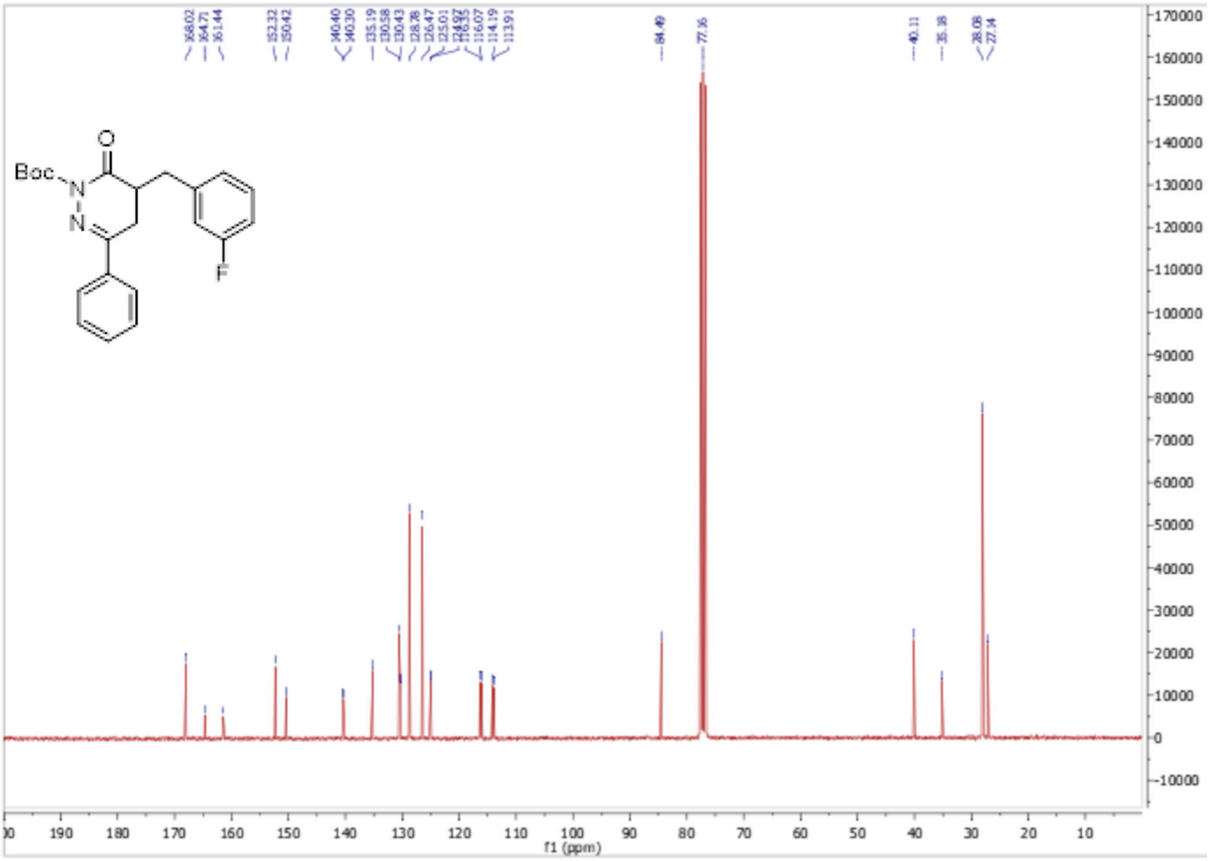

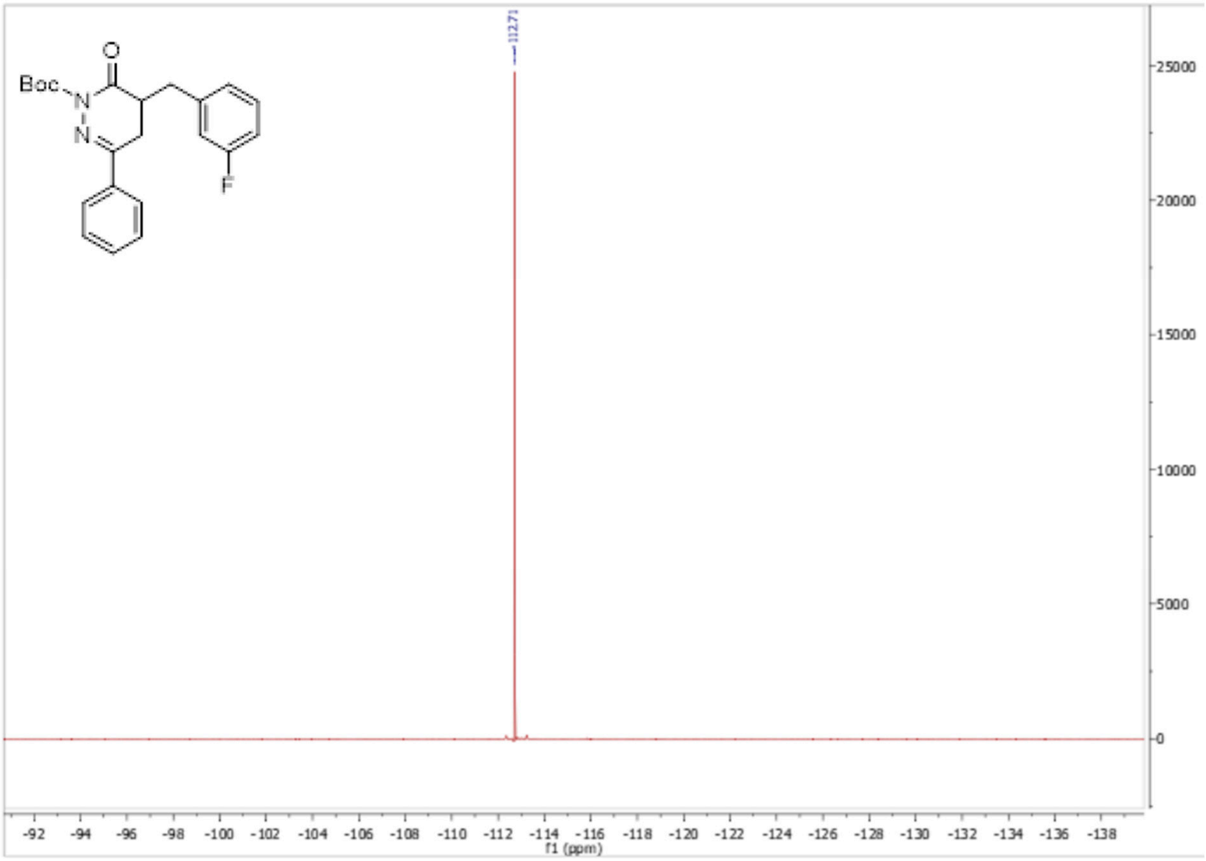
Boc
N
N
O
F
-112.71
25000
20000
15000
10000
5000
0
-92 -94 -96 -98 -100 -102 -104 -106 -108 -110 -112 -114 -116 -118 -120 -122 -124 -126 -128 -130 -132 -134 -136 -138
f1 (ppm)

***tert*-butyl 5-benzyl-6-oxo-3-phenyl-5,6-dihydropyridazine-1(*4H*)-carboxylate (7b)**

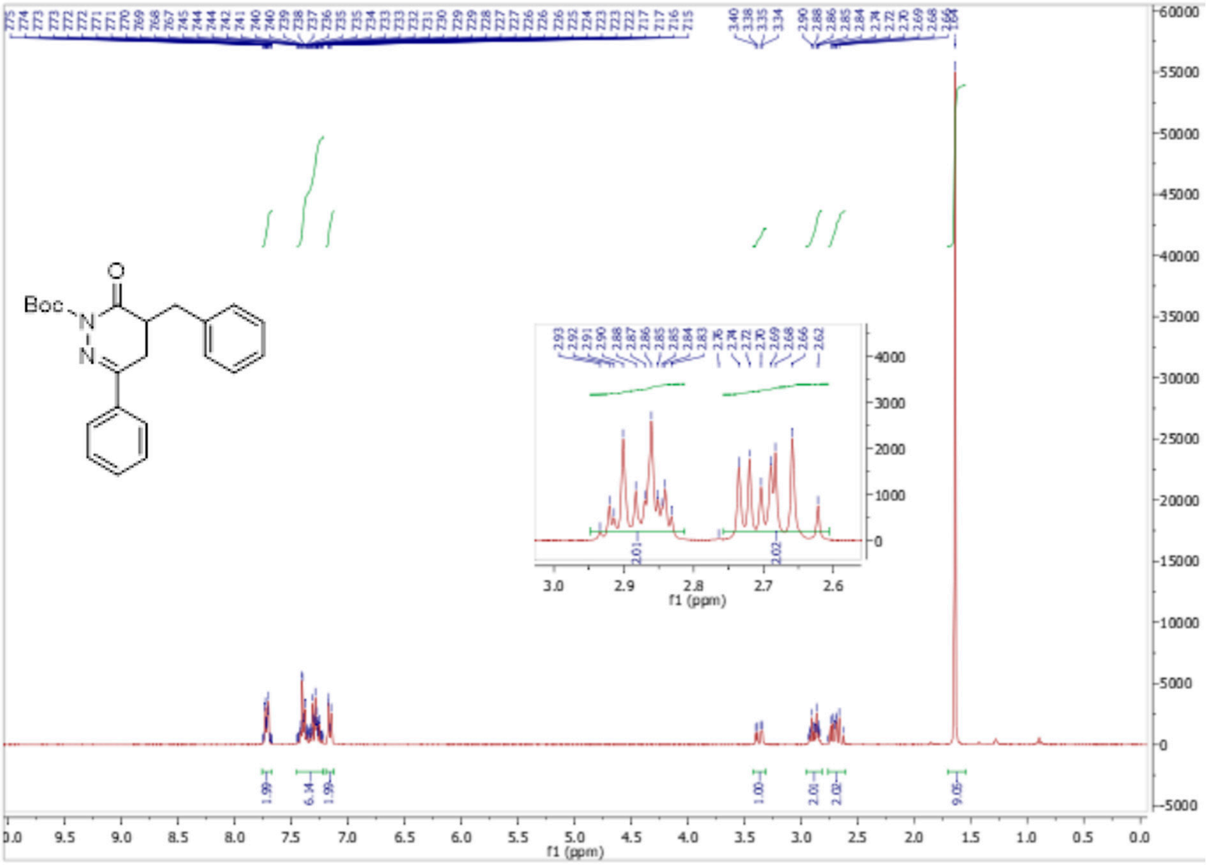

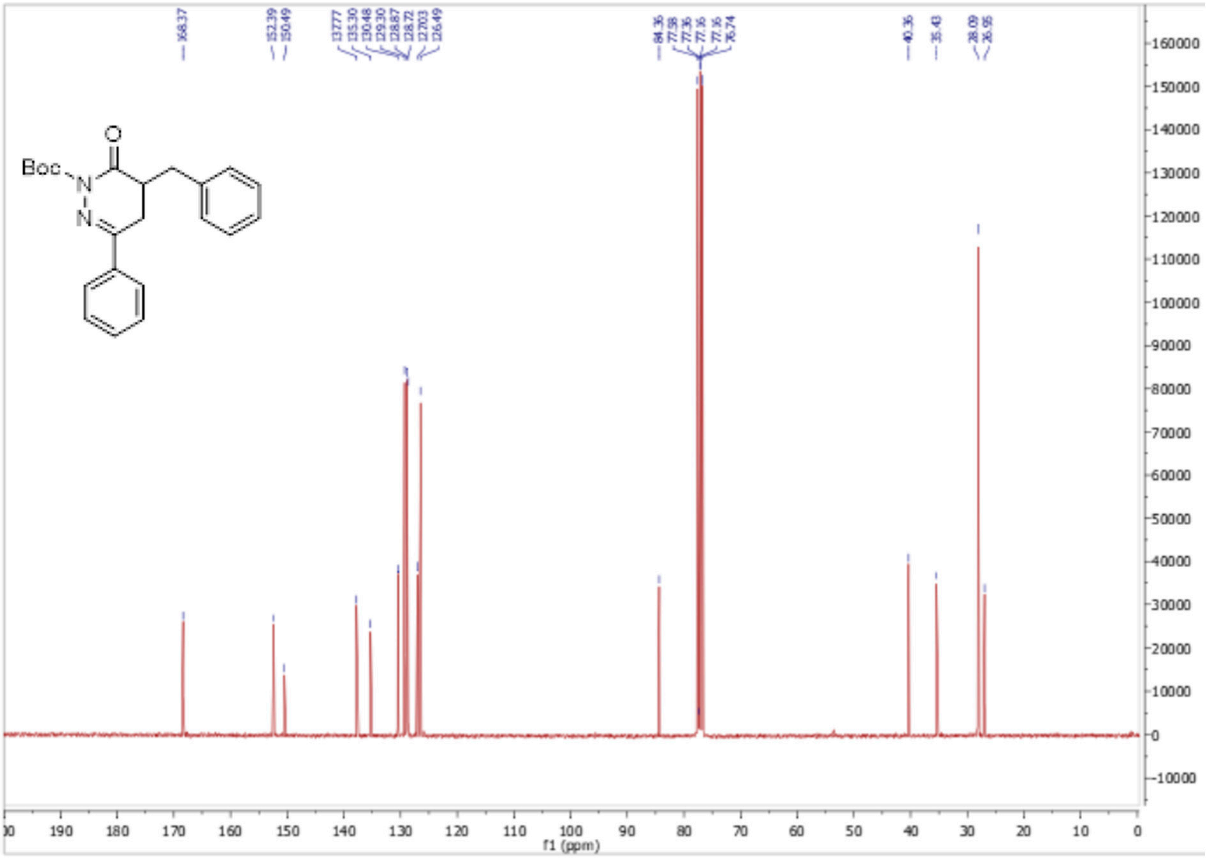

***tert*-butyl 5-(naphthalen-1-ylmethyl)-6-oxo-3-phenyl-5,6-dihydropyridazine-1(*4H*)-carboxylate (7c)**

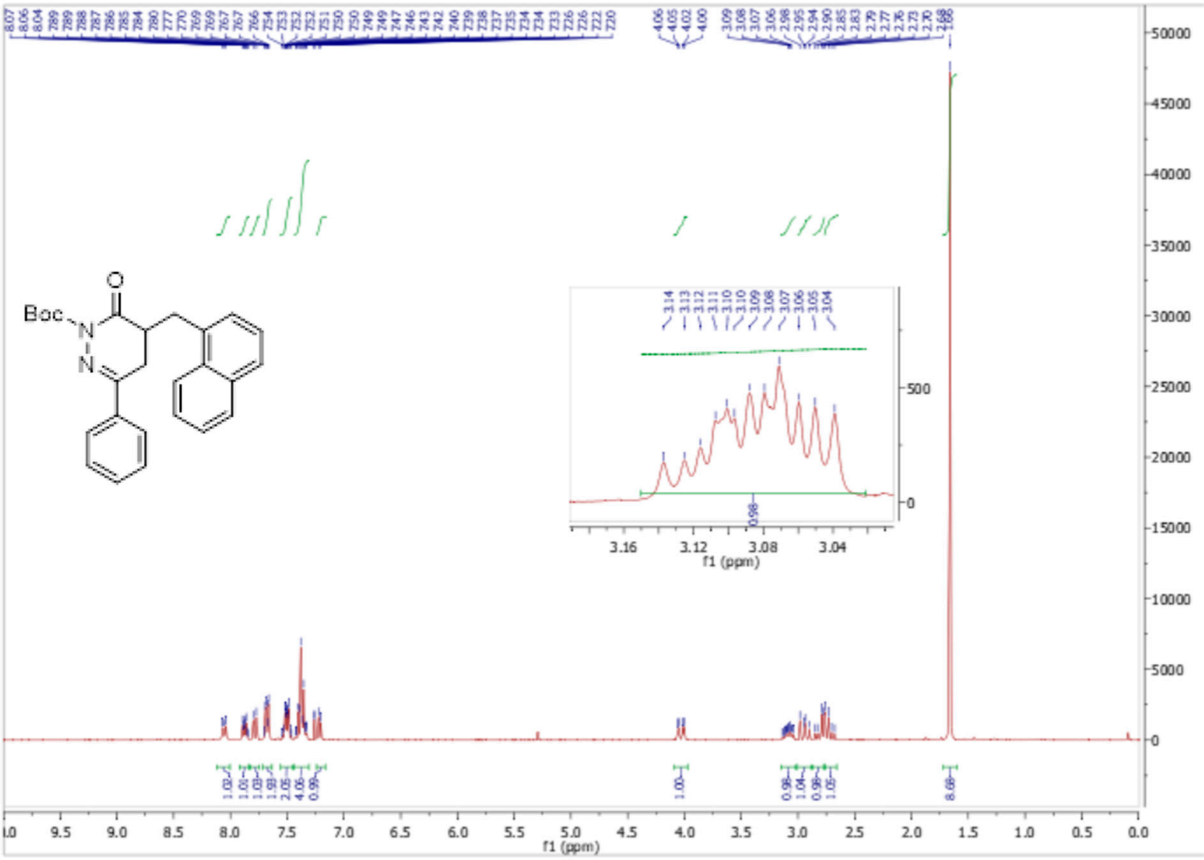

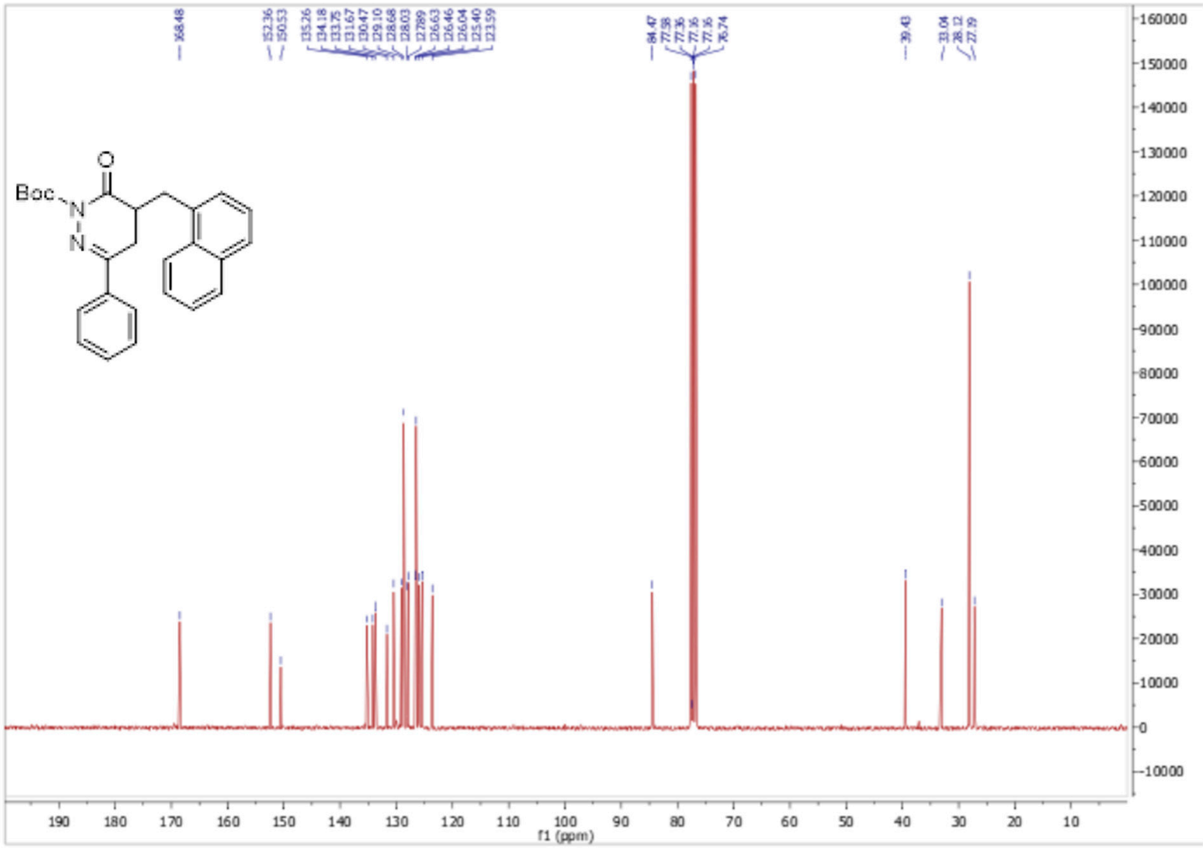

***tert*-butyl 5-(4-fluorobenzyl)-6-oxo-3-phenyl-5,6-dihydropyridazine-1(*4H*)-carboxylate (7d)**

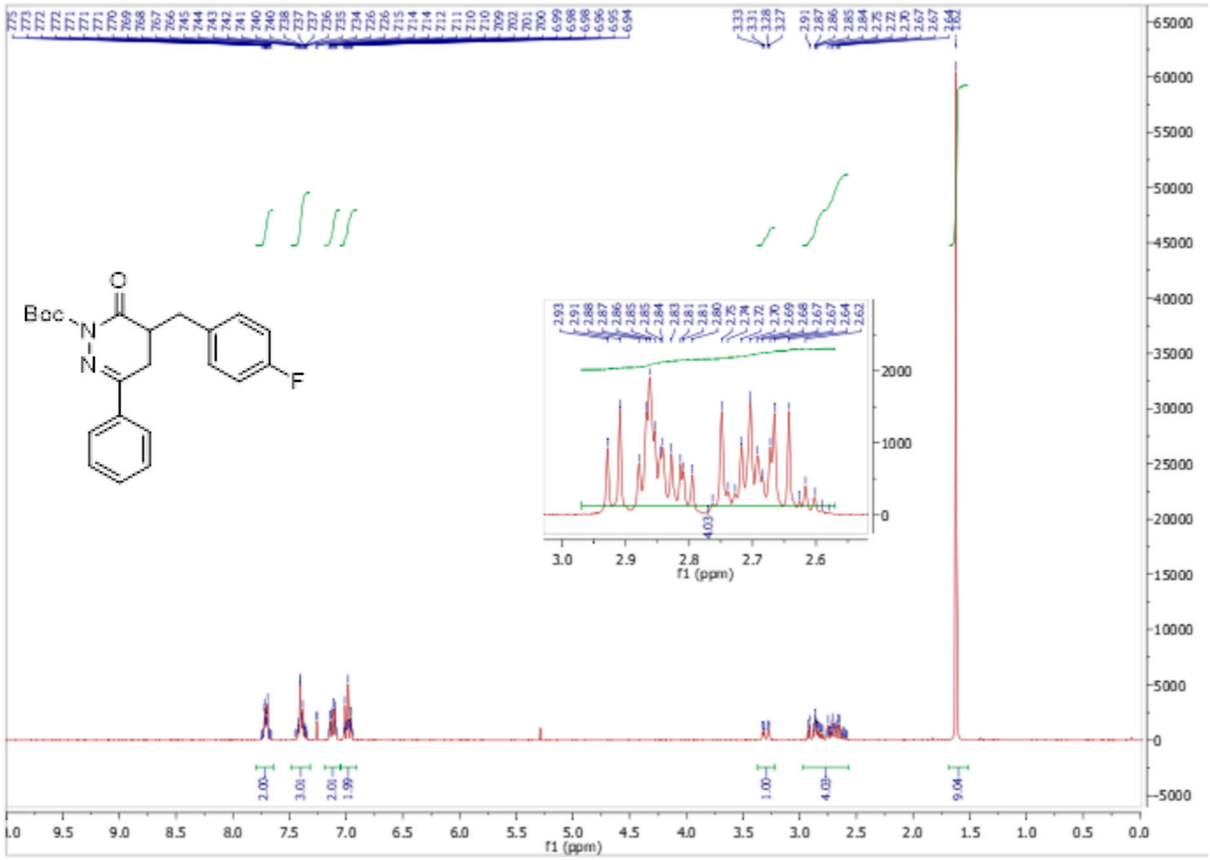

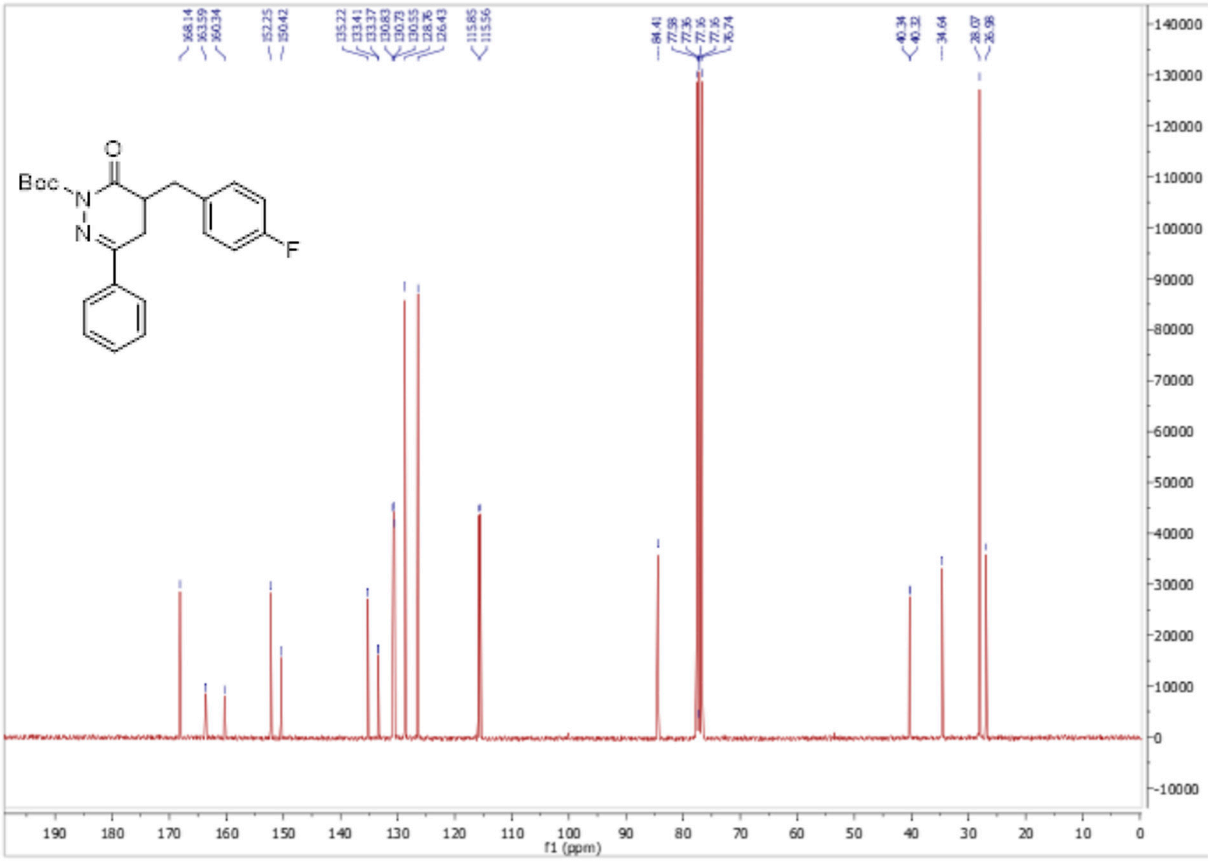

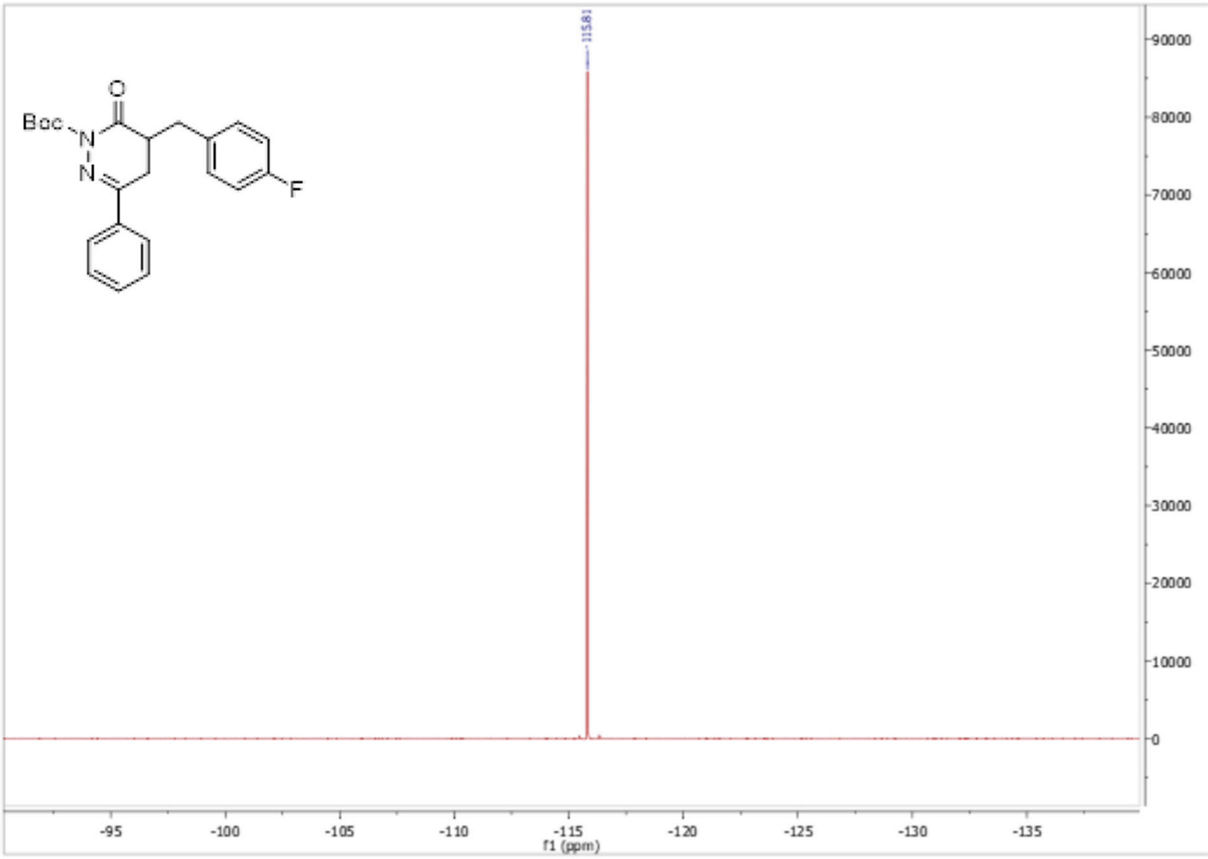

Boc
N
N
O
F
90000
80000
70000
60000
50000
40000
30000
20000
10000
0
-95
-100
-105
-110
-115
-120
-125
-130
-135
f1 (ppm)

***tert*-butyl 5-(4-bromobenzyl)-6-oxo-3-phenyl-5,6-dihydropyridazine-1(*4H*)-carboxylate (7e)**

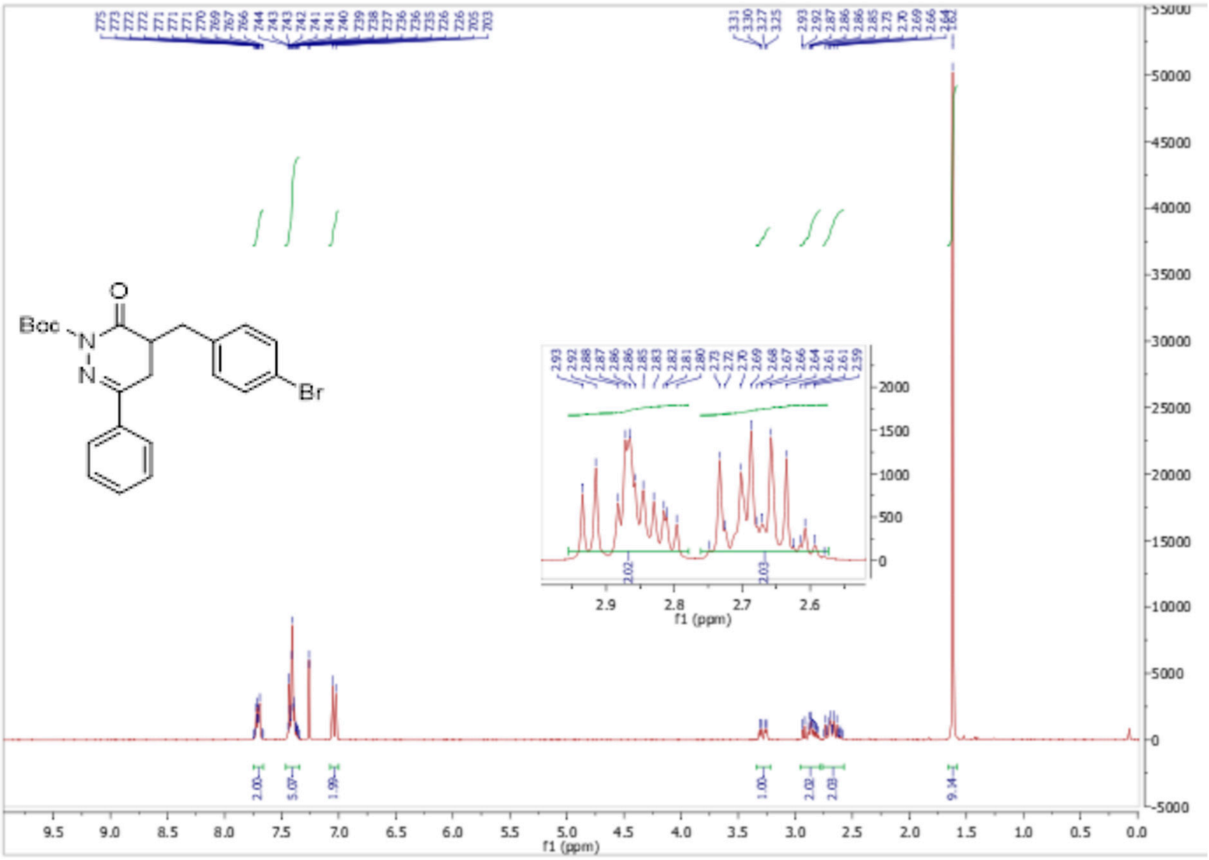

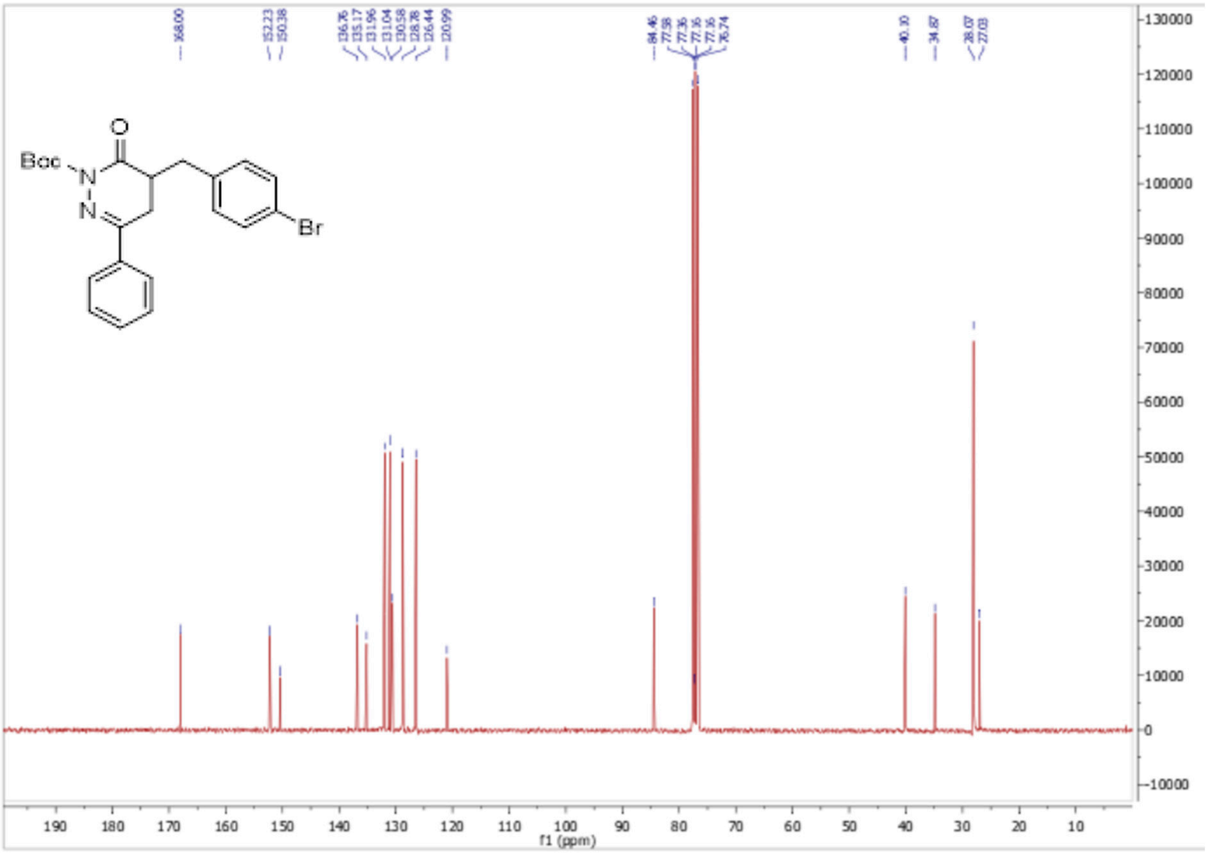

***tert*-butyl 5-(4-chlorobenzyl)-6-oxo-3-phenyl-5,6-dihydropyridazine-1(*4H*)-carboxylate (7f)**

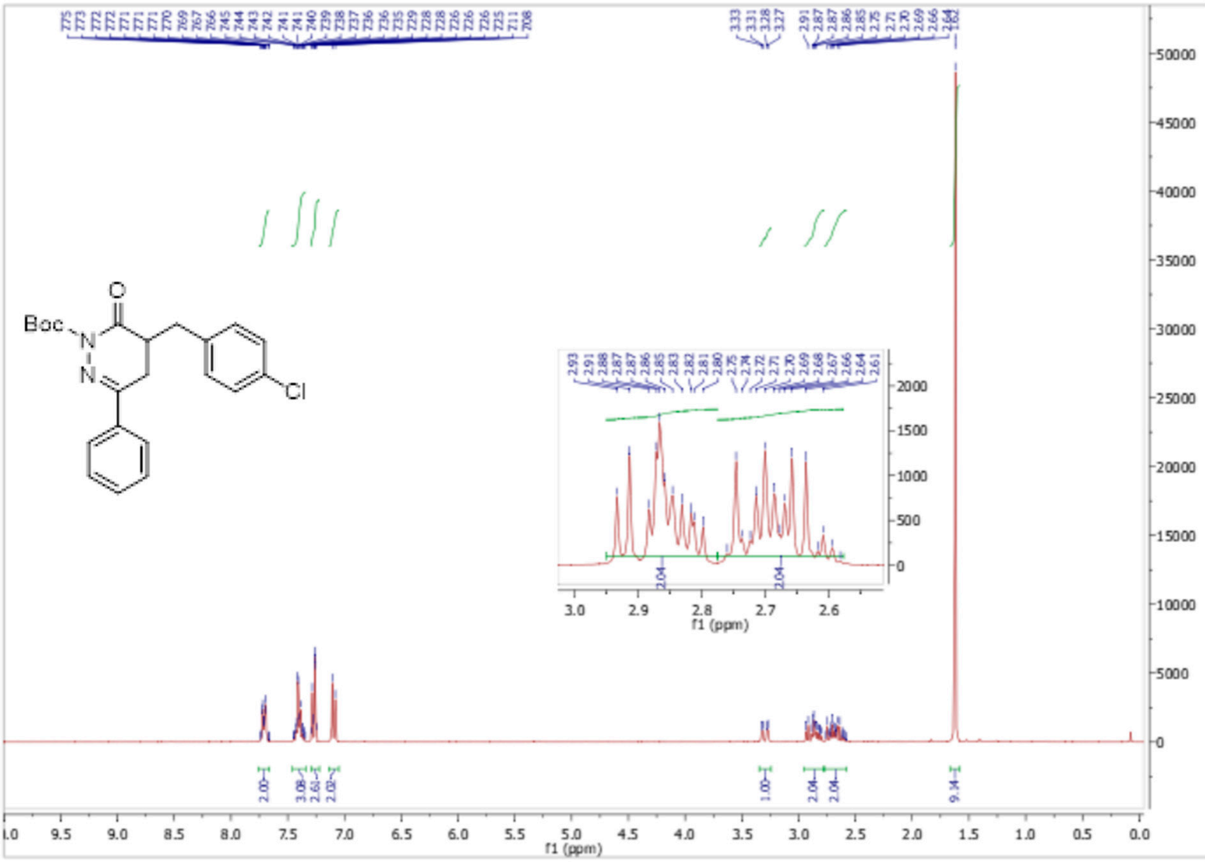

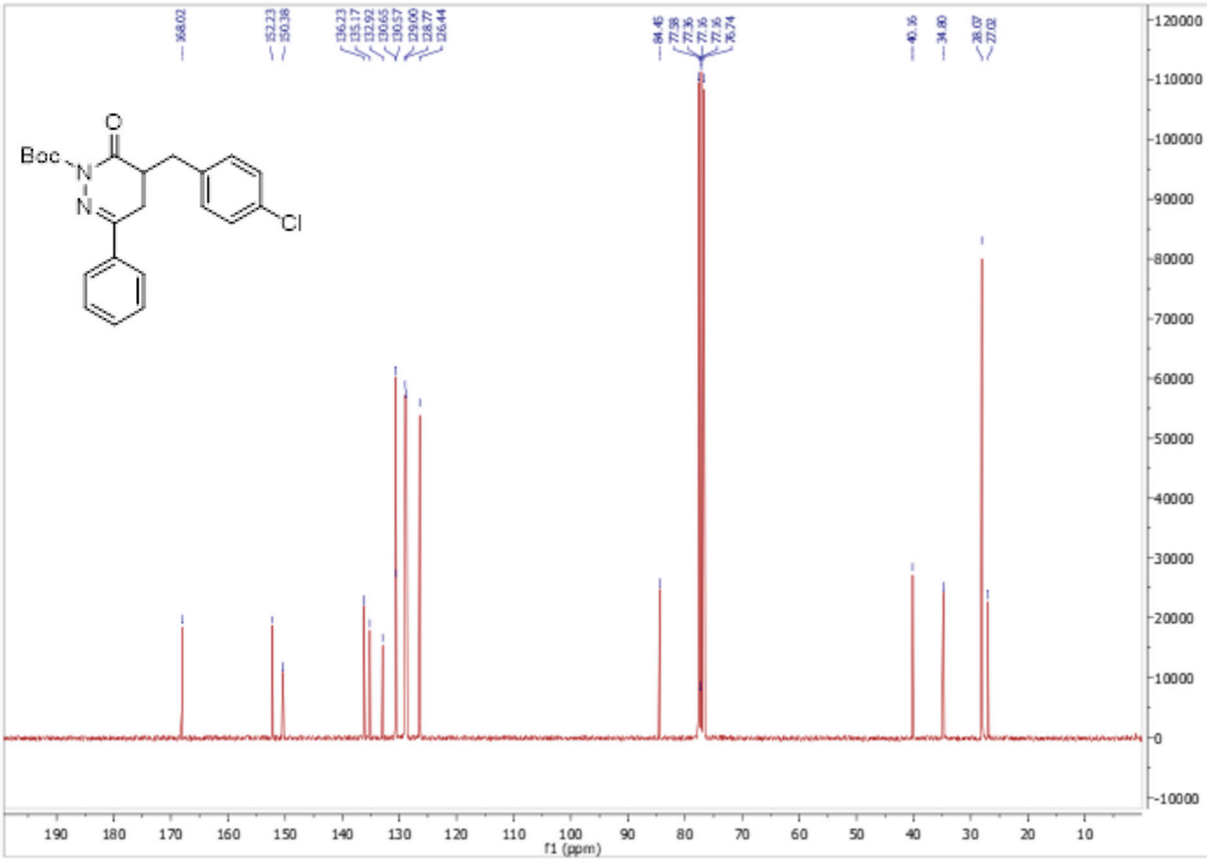

***tert*-butyl 5-(2-cyanobenzyl)-6-oxo-3-phenyl-5,6-dihydropyridazine-1(4H)-carboxylate (7g)**

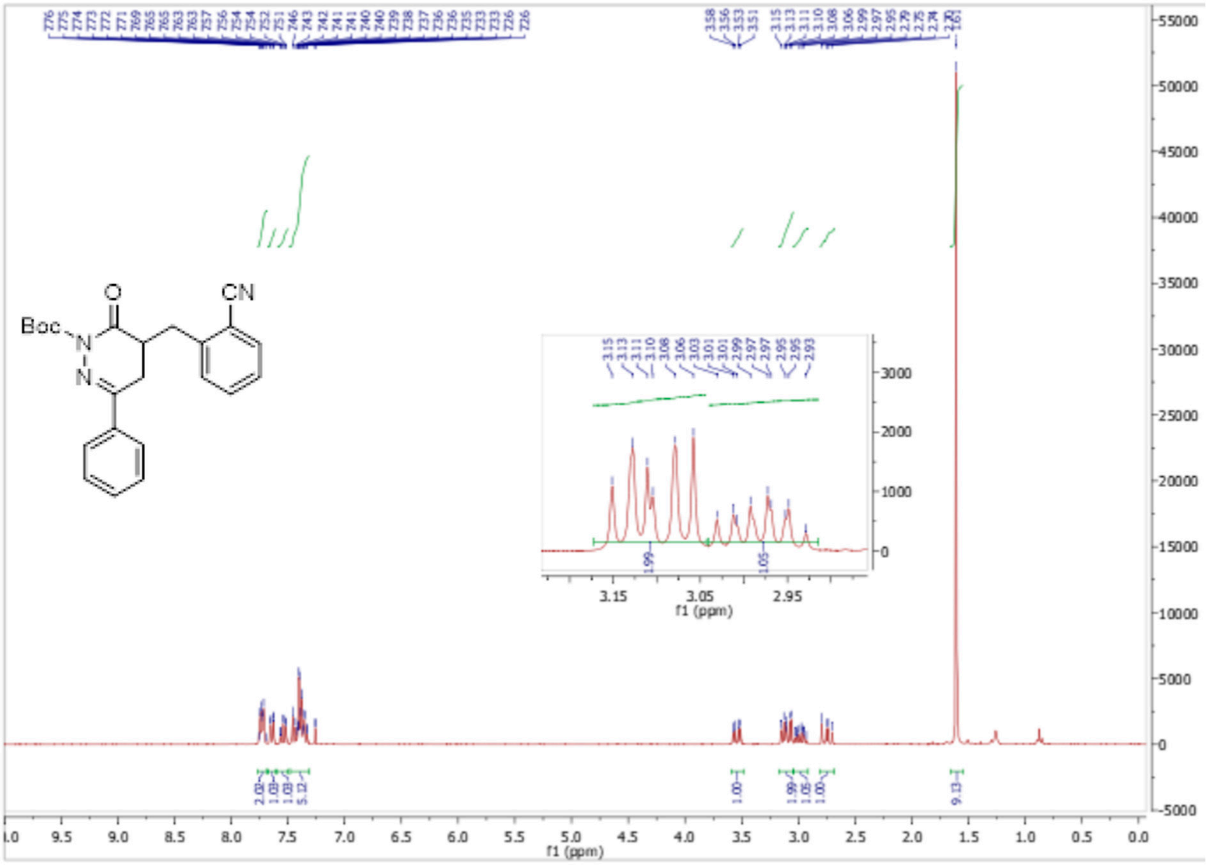

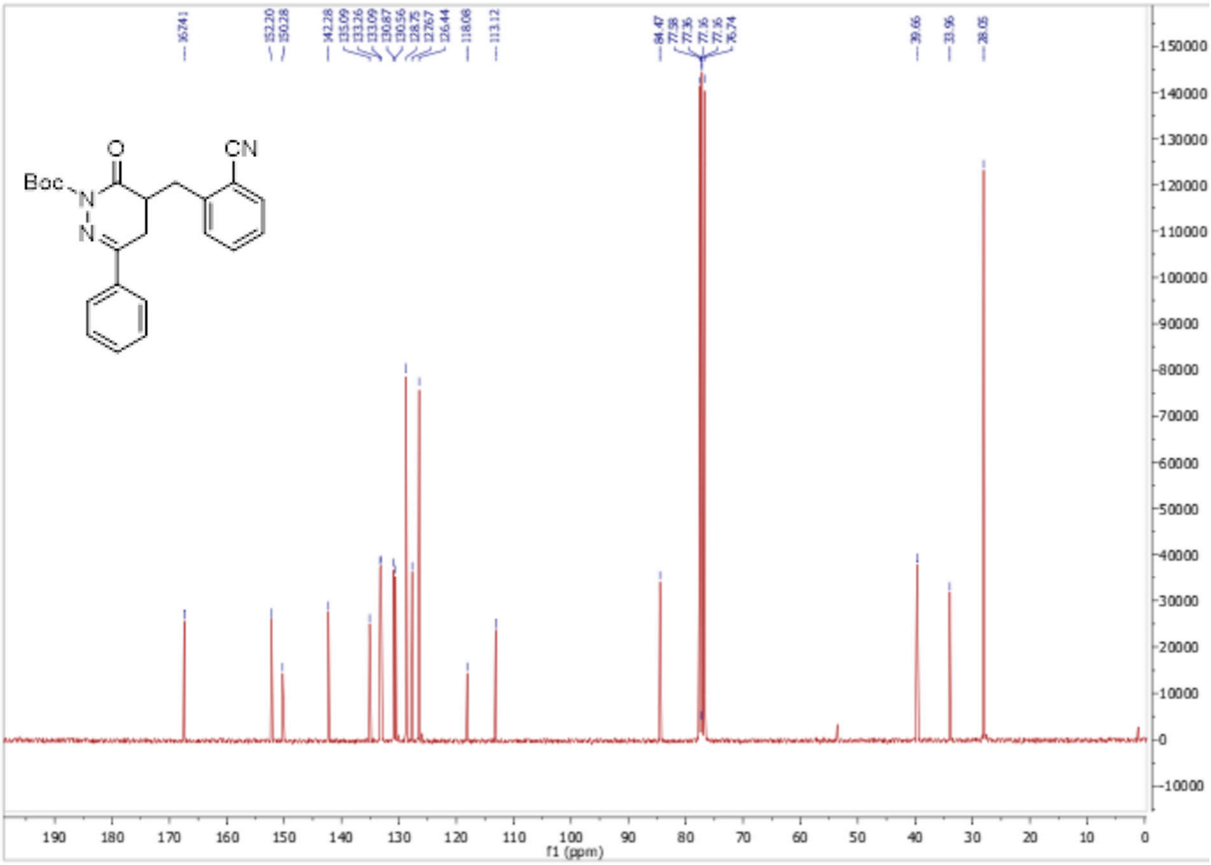

***tert*-butyl 5-(2-methoxybenzyl)-6-oxo-3-phenyl-5,6-dihydropyridazine-1(*4H*)-carboxylate (7h)**

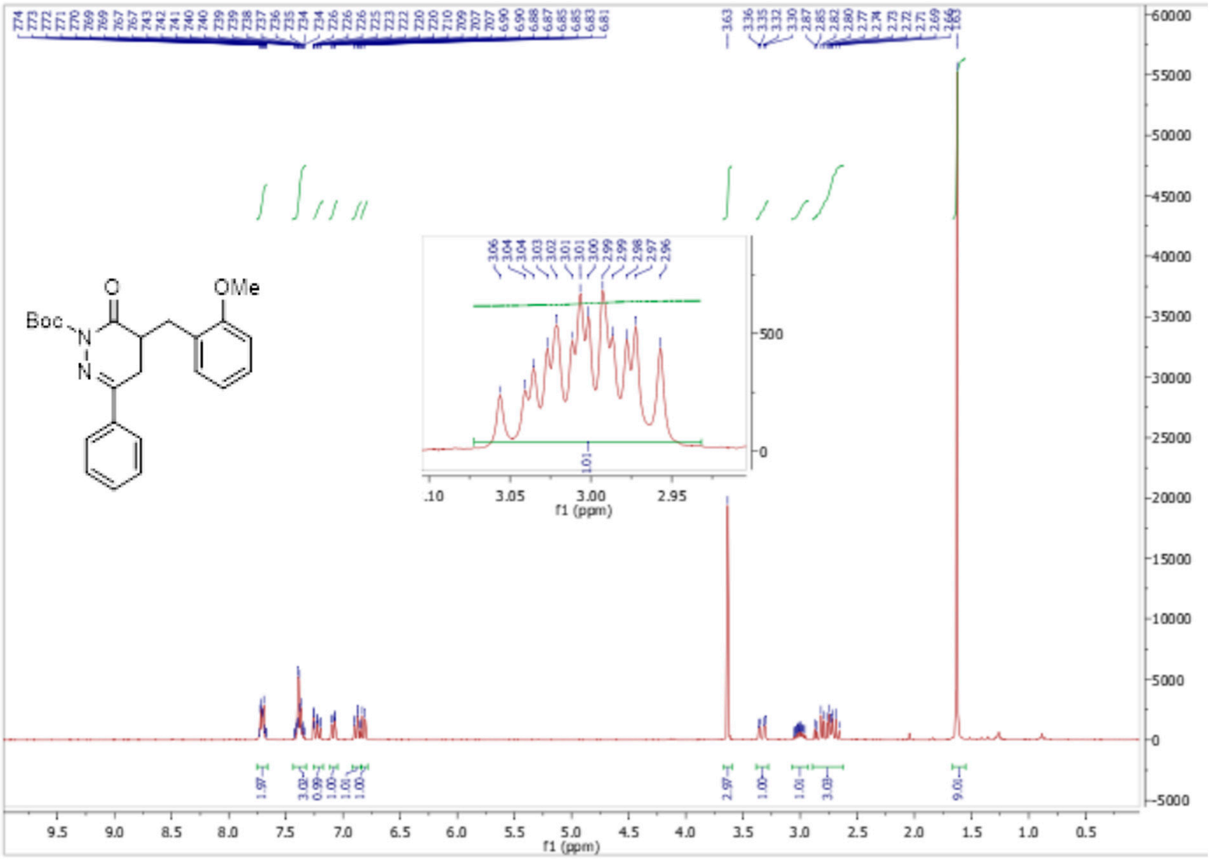

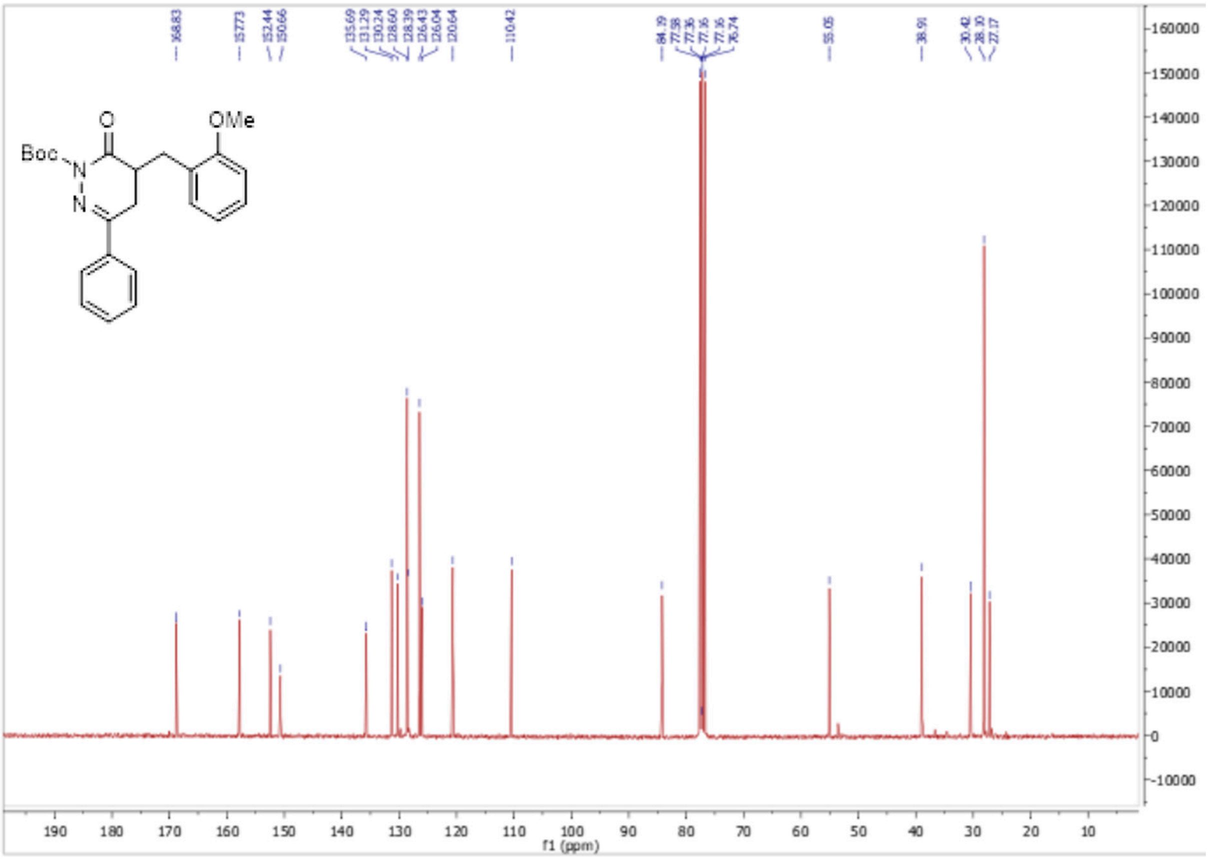

tert-butyl 5-(4-methylbenzyl)-6-oxo-3-phenyl-5,6-dihydropyridazine-1(_4H_)-carboxylate (7i)

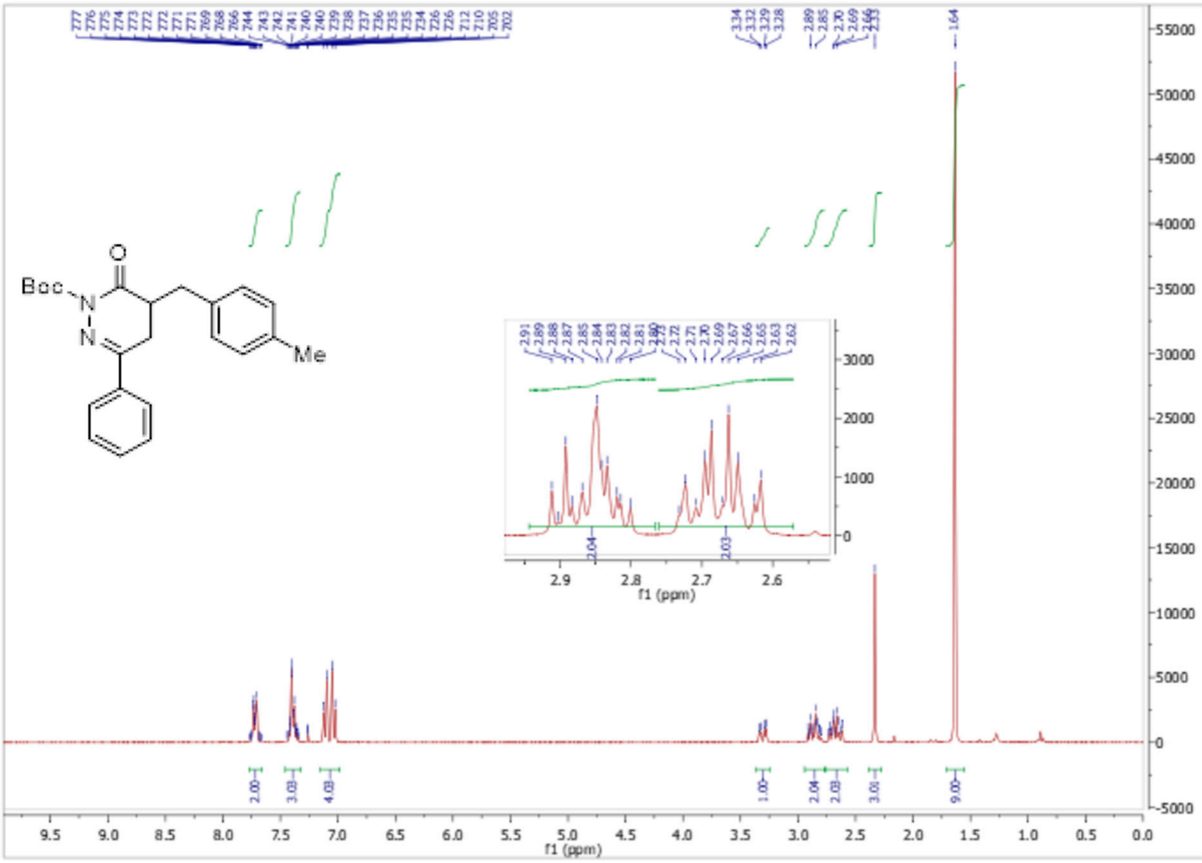

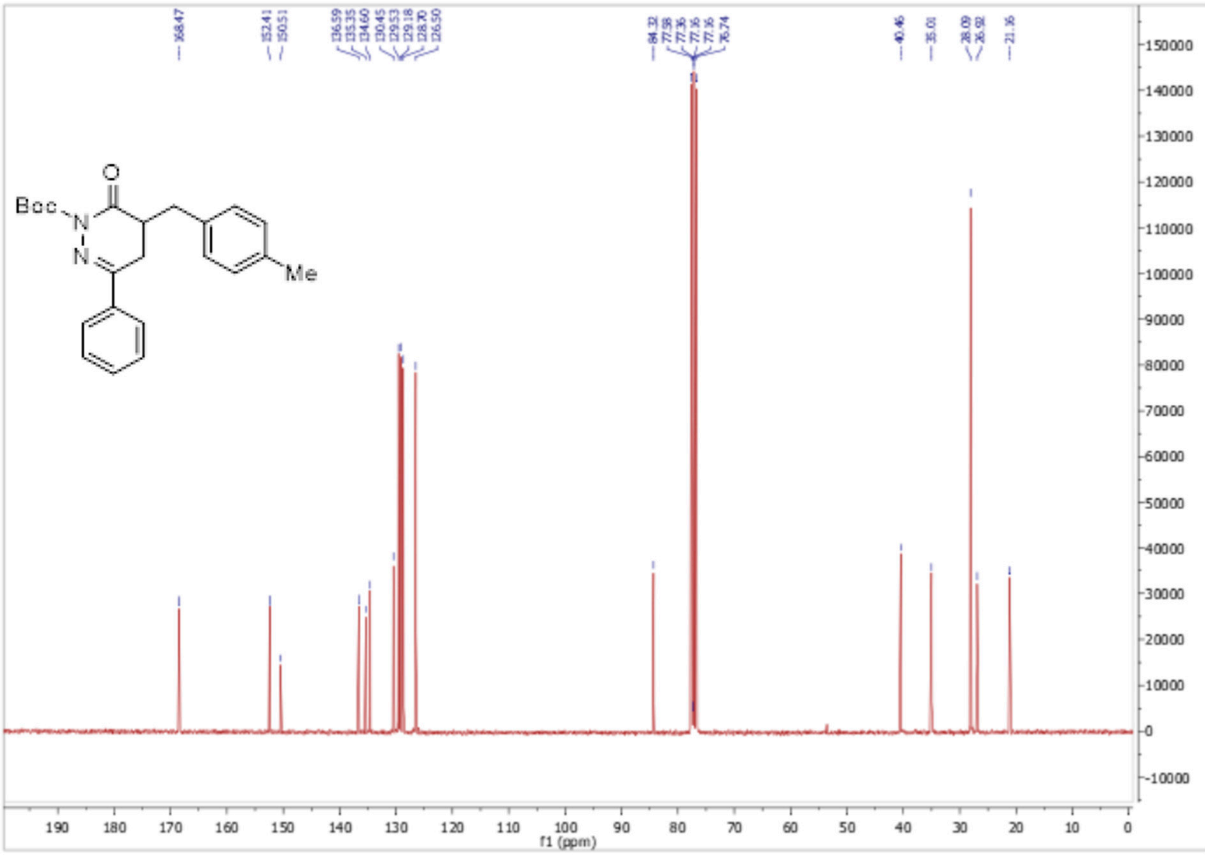

***tert*-butyl 5-(2,5-dimethylbenzyl)-6-oxo-3-phenyl-5,6-dihydropyridazine-1(*4H*)-carboxylate (7j)**

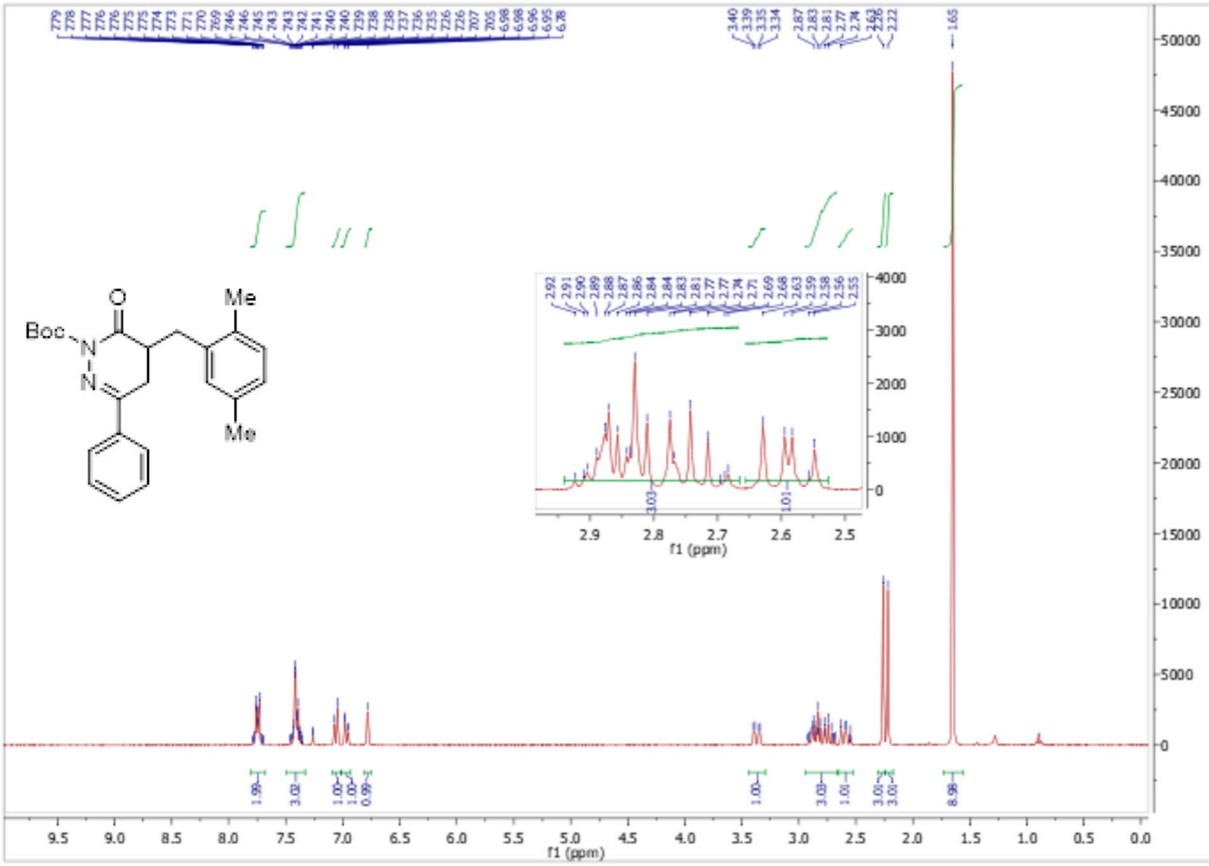

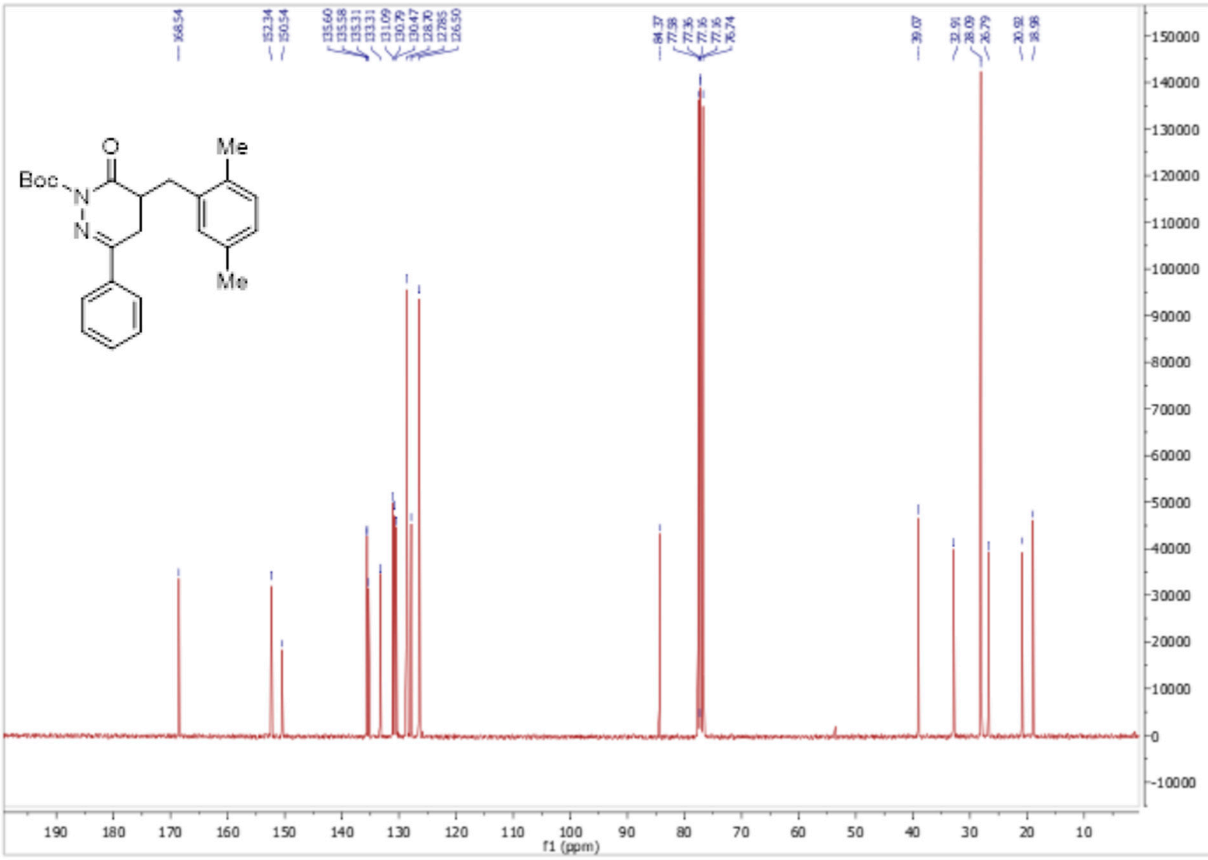

***tert*-butyl 6-oxo-3-phenyl-5-(thiophen-2-ylmethyl)-5,6-dihydropyridazine-1(*4H*)-carboxylate (7k)**

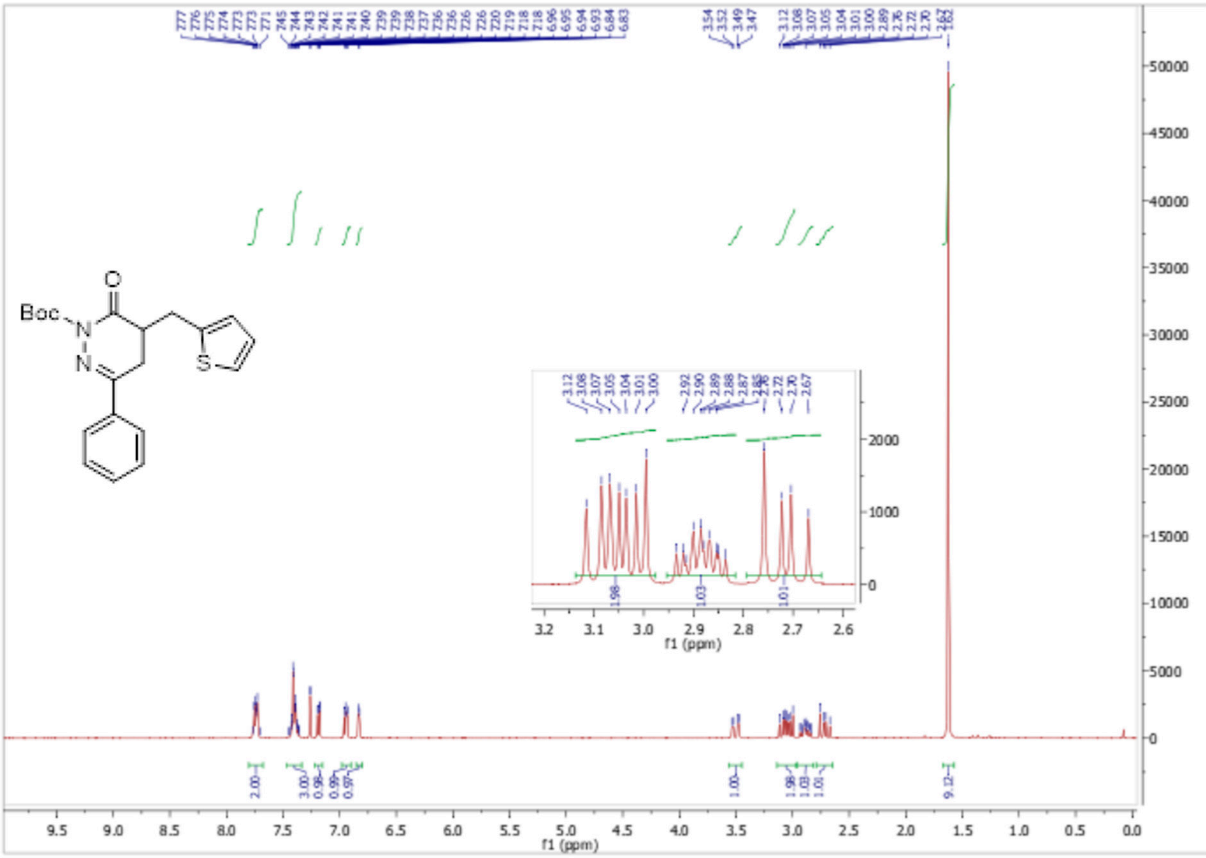

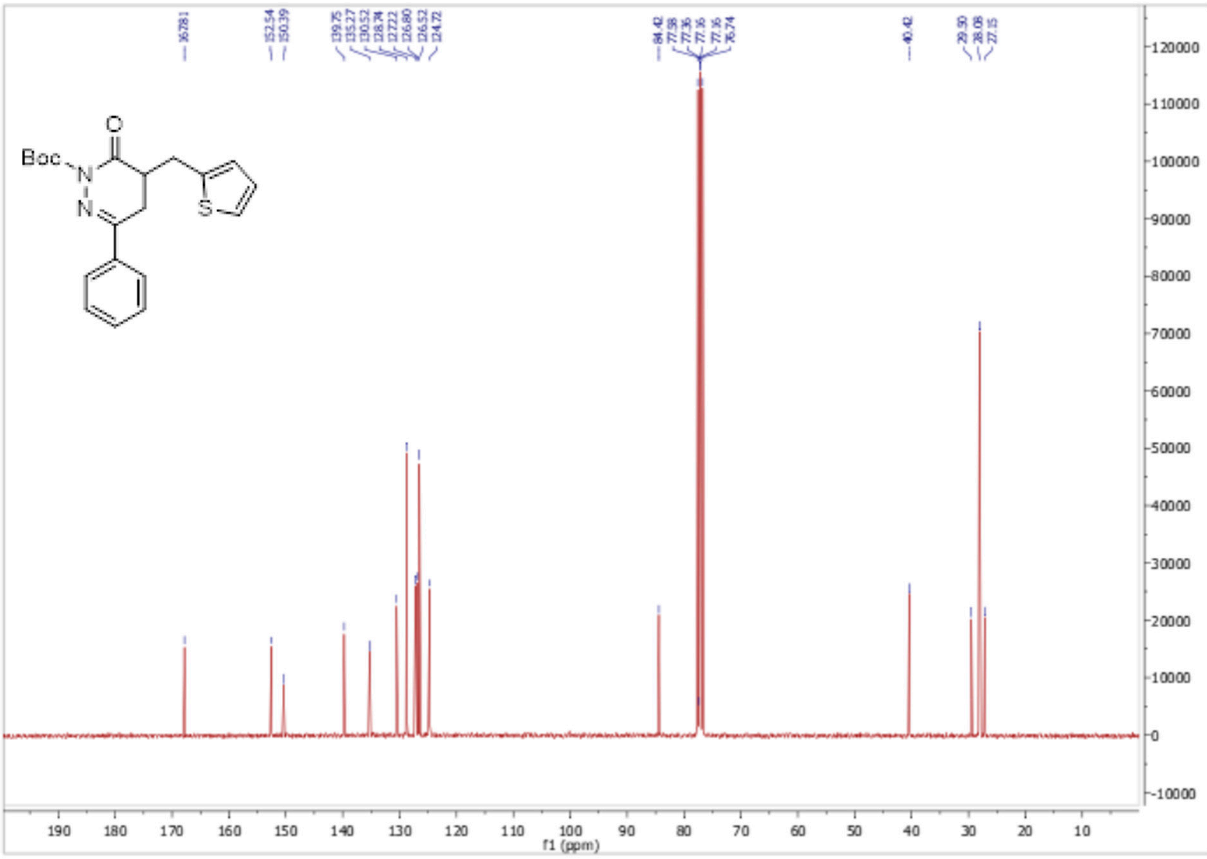

***tert*-butyl 5-isobutyl-6-oxo-3-phenyl-5,6-dihydropyridazine-1(*4H*)-carboxylate (7l)**

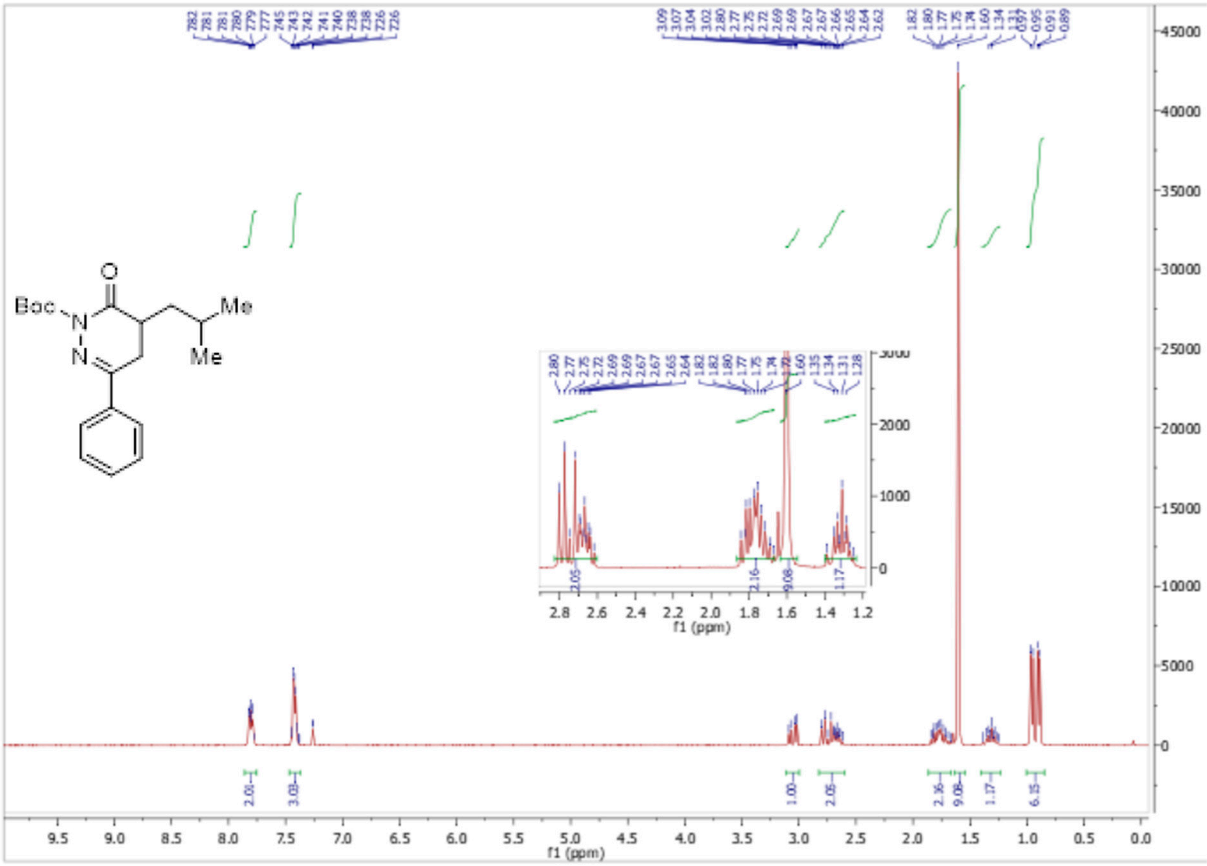

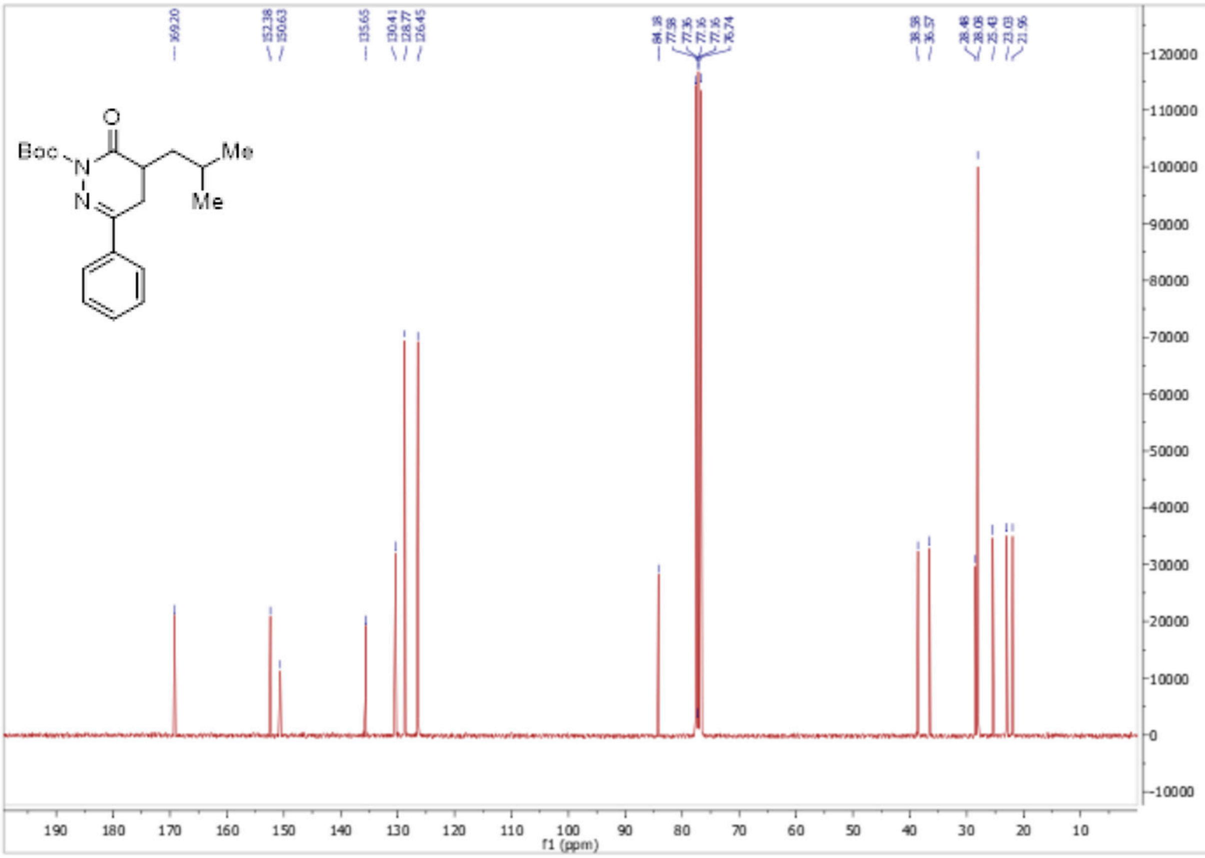

***tert*-butyl 6-oxo-3-phenyl-5-(3-phenylpropyl)-5,6-dihydropyridazine-1(*4H*)-carboxylate (7m)**

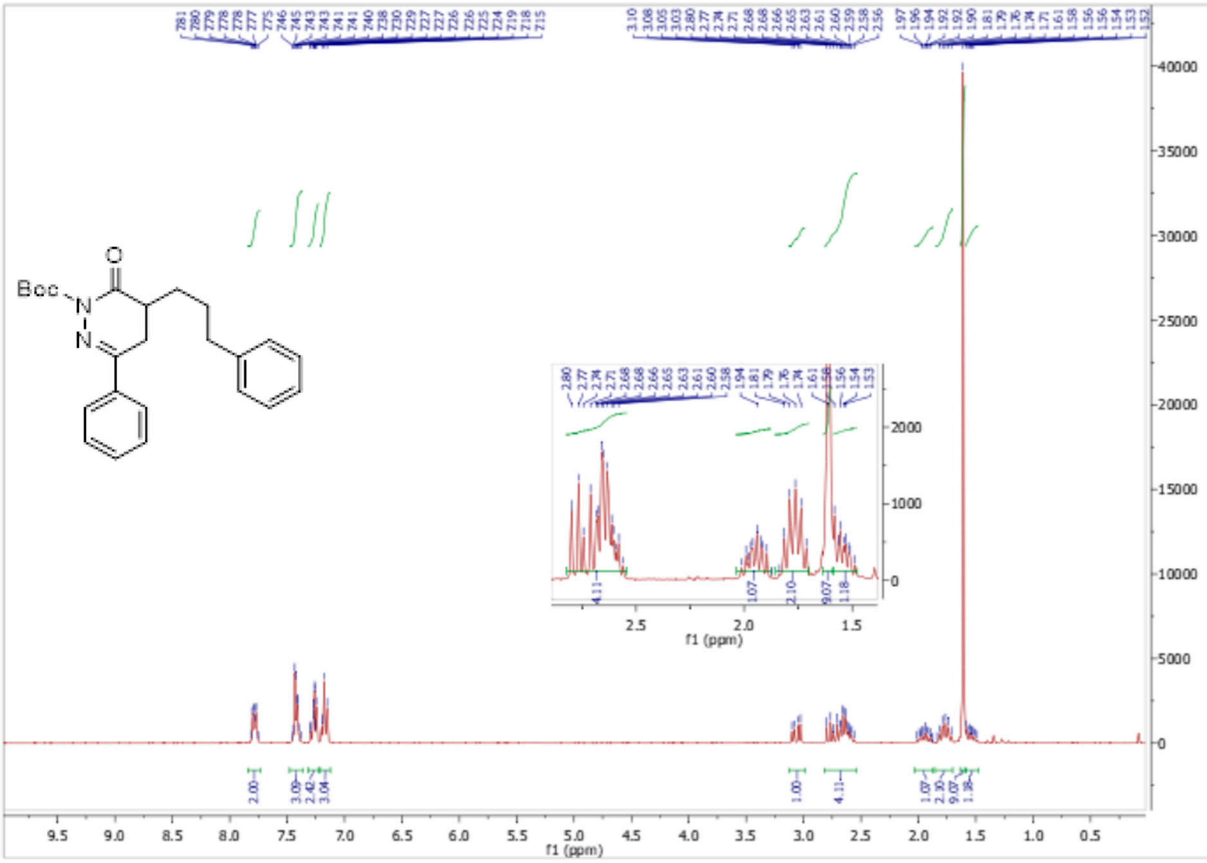

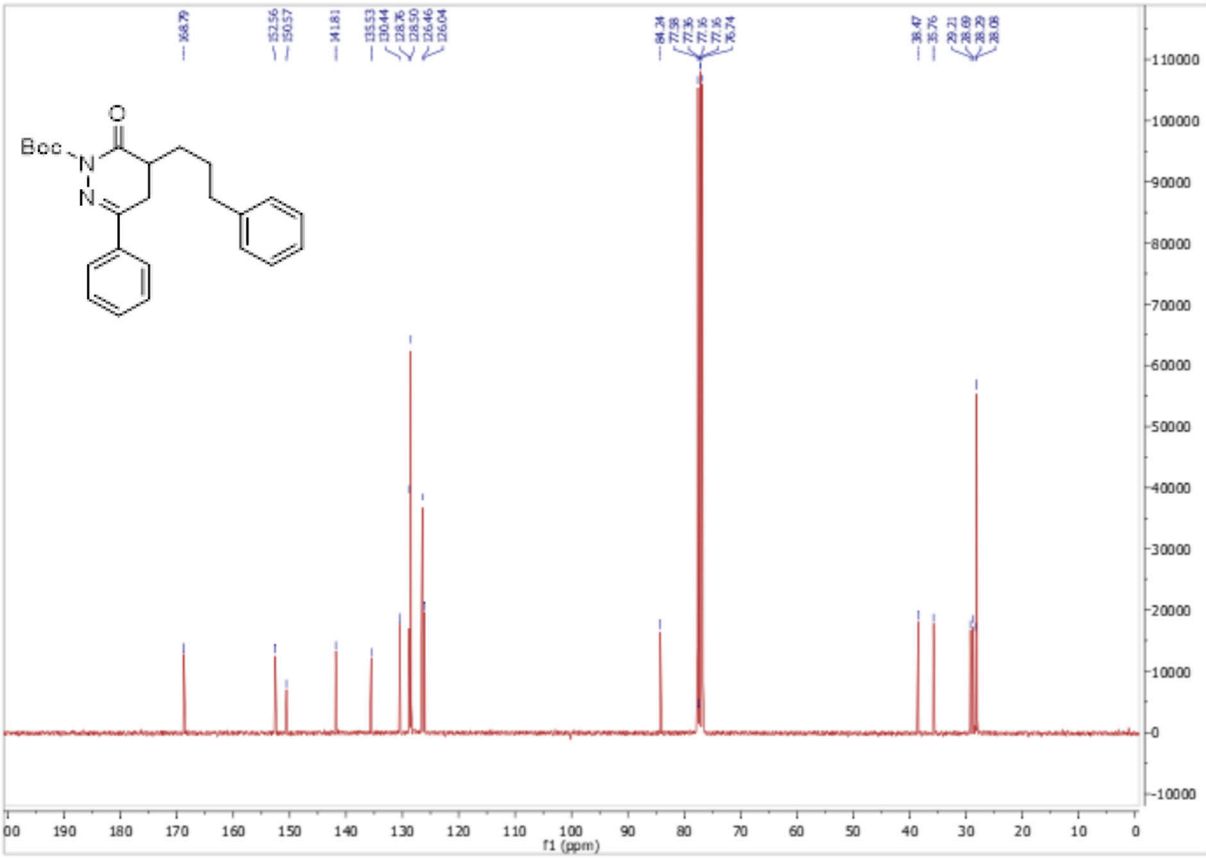

***tert*-butyl 6-oxo-3,5-diphenyl-5,6-dihydropyridazine-1(*4H*)-carboxylate (7n)**

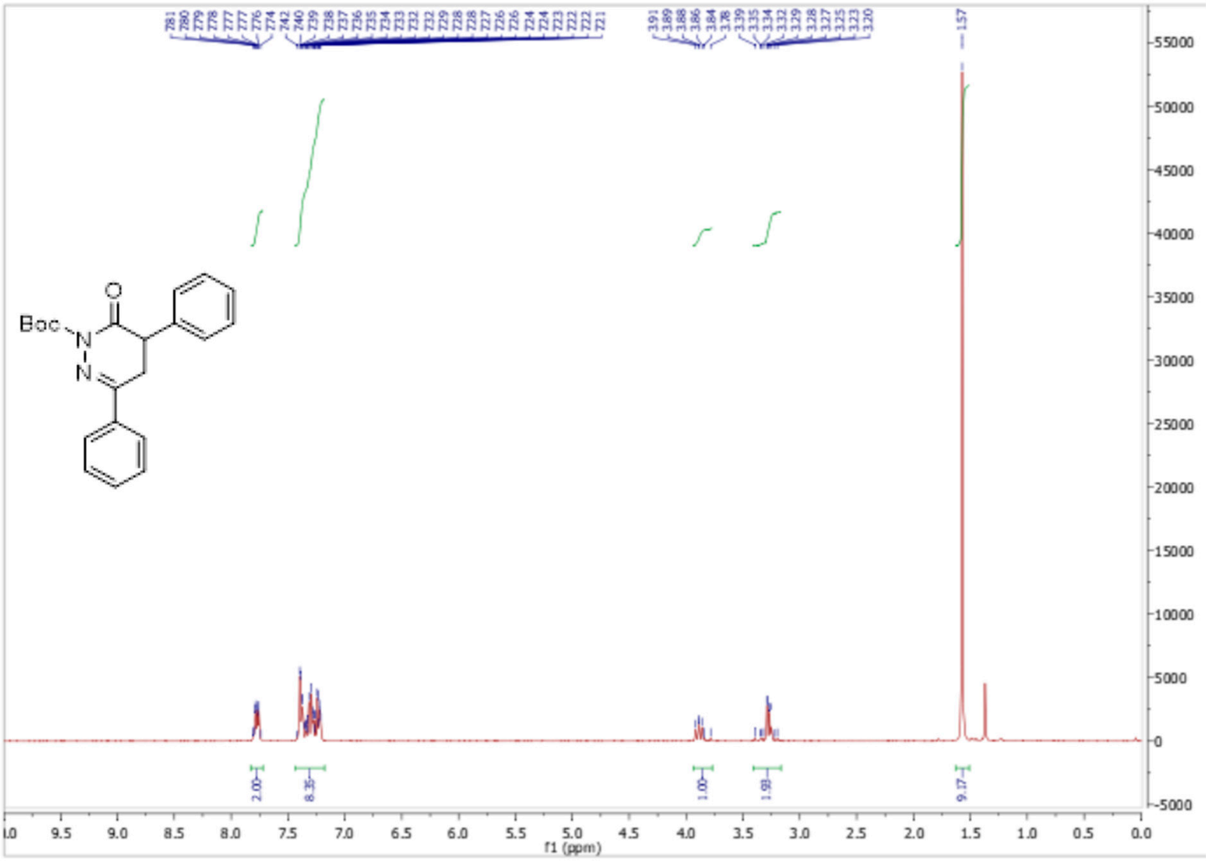

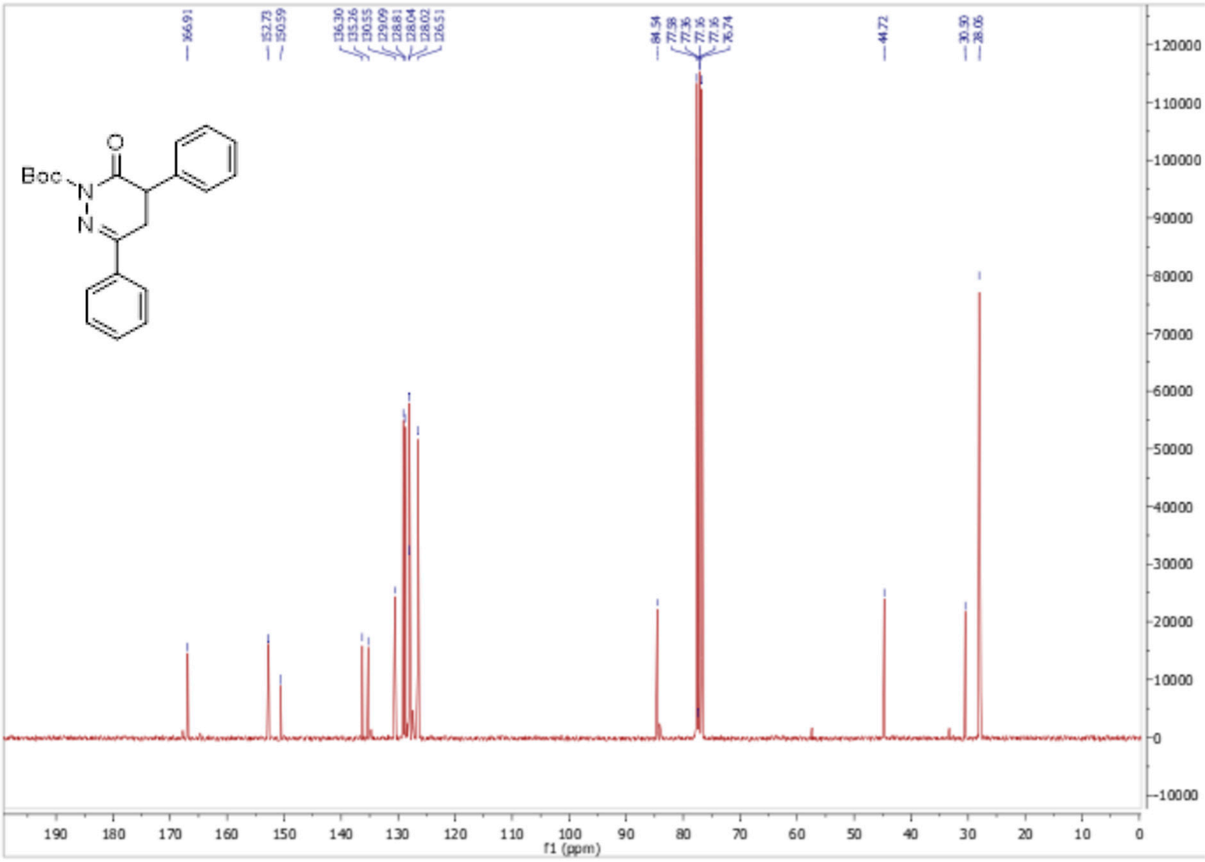

***tert*-butyl 5-(3-fluorobenzyl)-6-oxo-5,6-dihydropyridazine-1(*4H*)-carboxylate (8)**

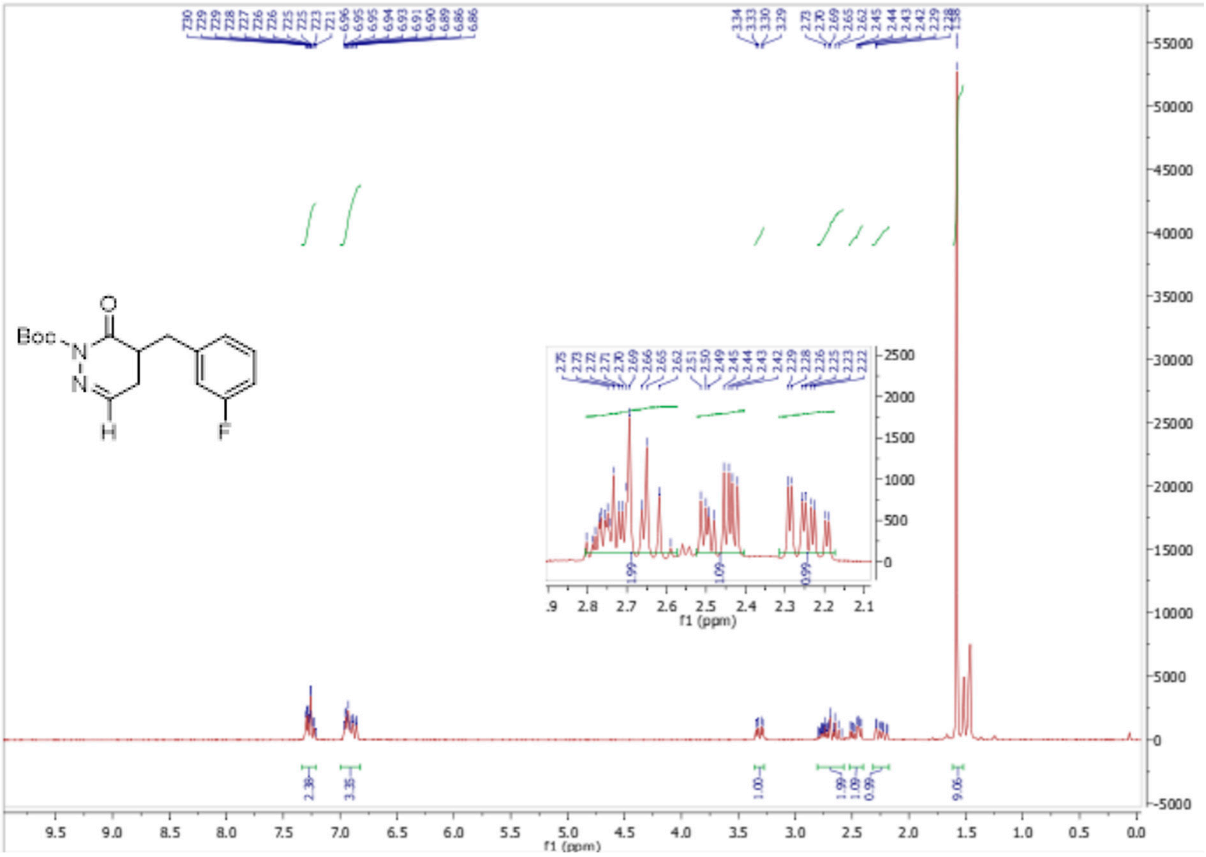

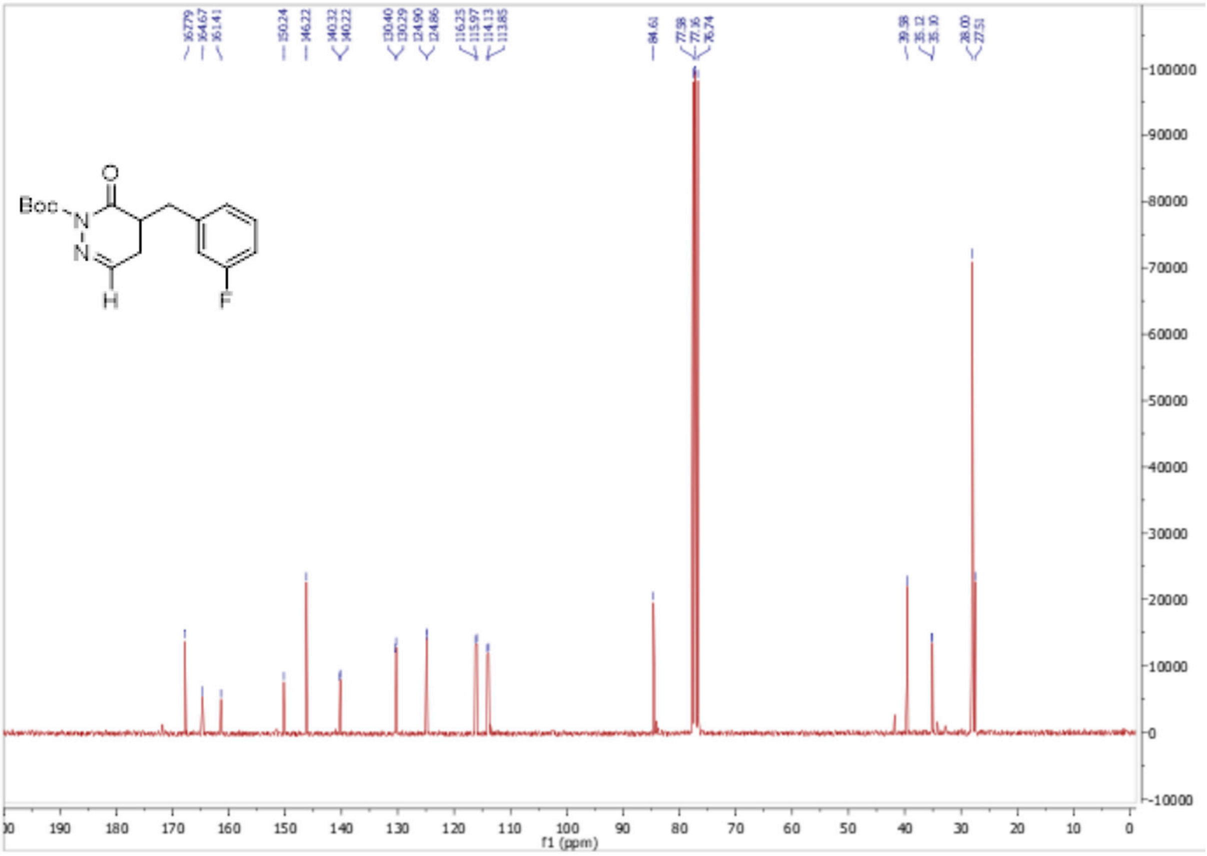

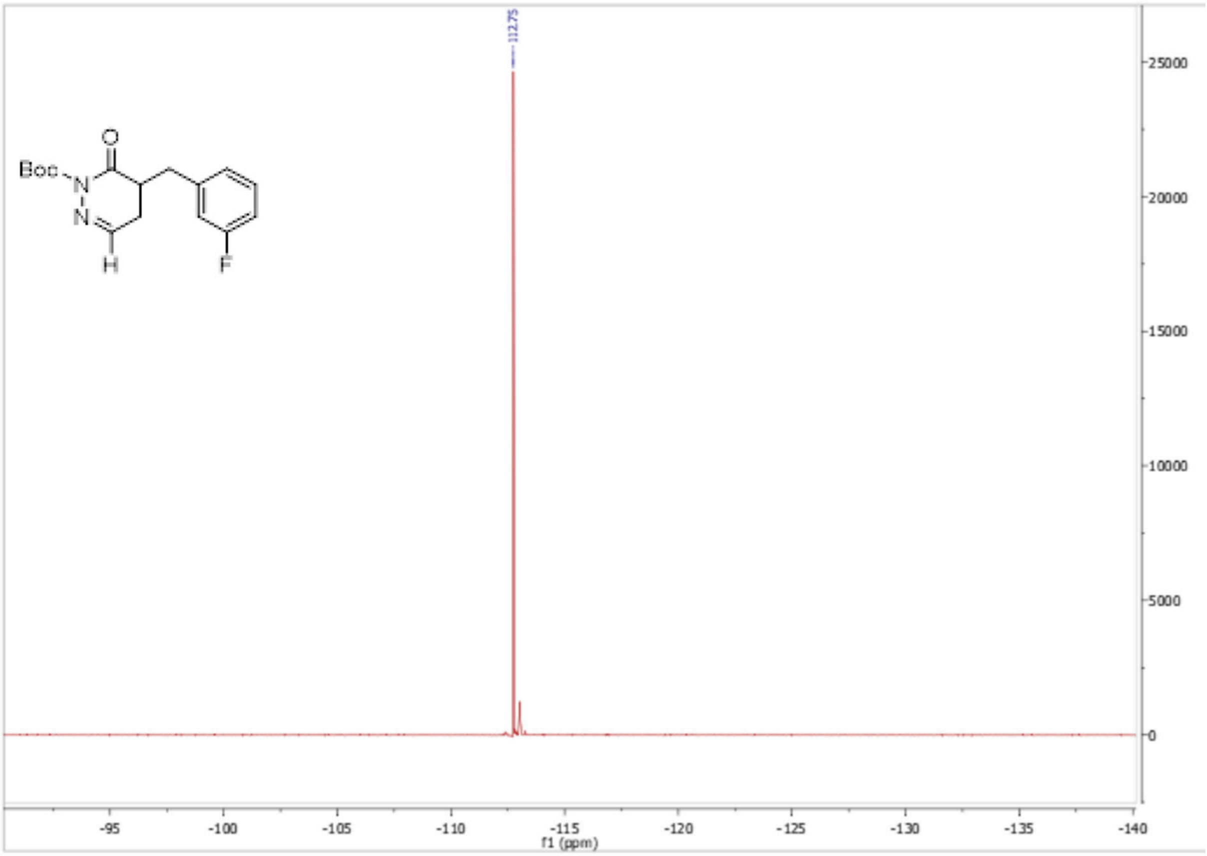

-112.75
Boc
N
N
O
H
F
25000
20000
15000
10000
5000
0
-95
-100
-105
-110
-115
-120
-125
-130
-135
-140
f1 (ppm)

IV. 1. 5. NMR spectra of α-disubstituted pyridazinones

***tert*-butyl 5-(3-fluorobenzyl)-5-(3-methoxy-3-oxopropyl)-6-oxo-3-phenyl-5,6-dihydropyridazine-1(*4H*)-carboxylate (10a)**

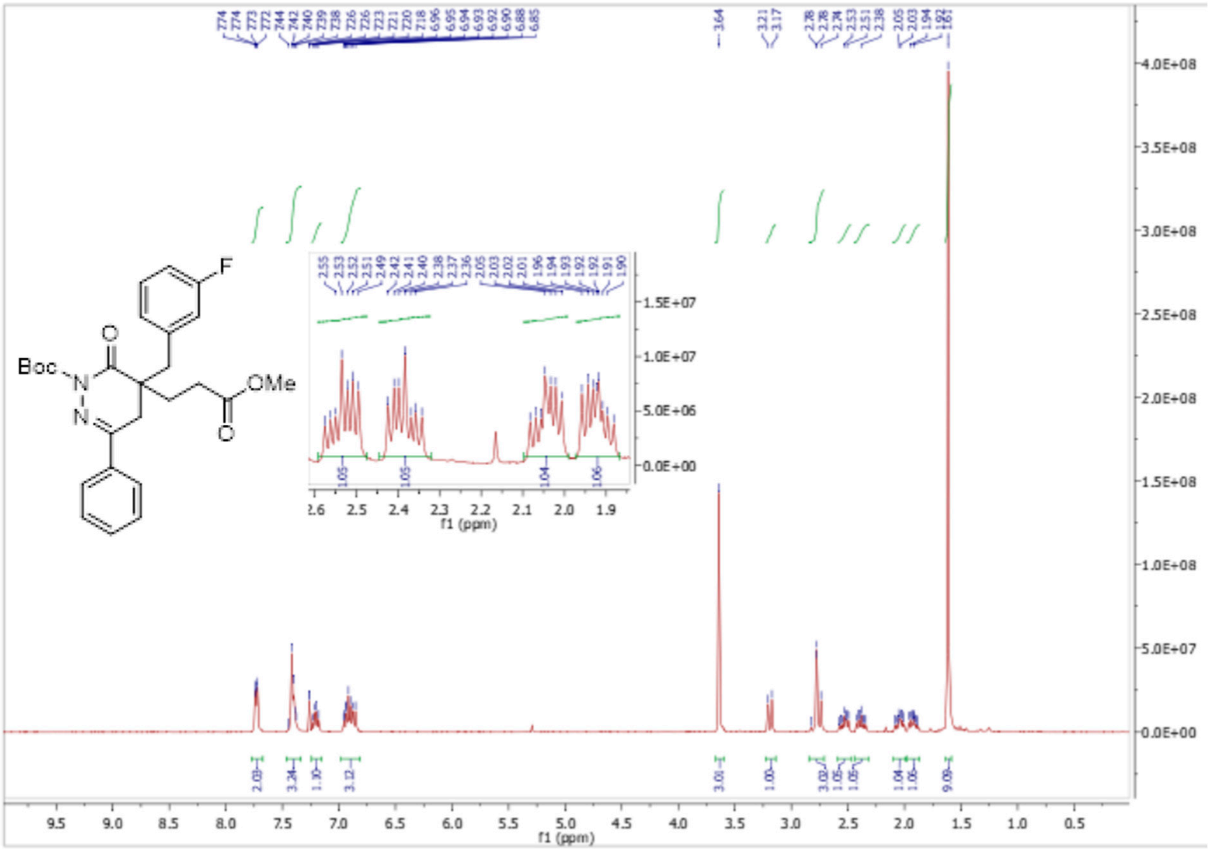

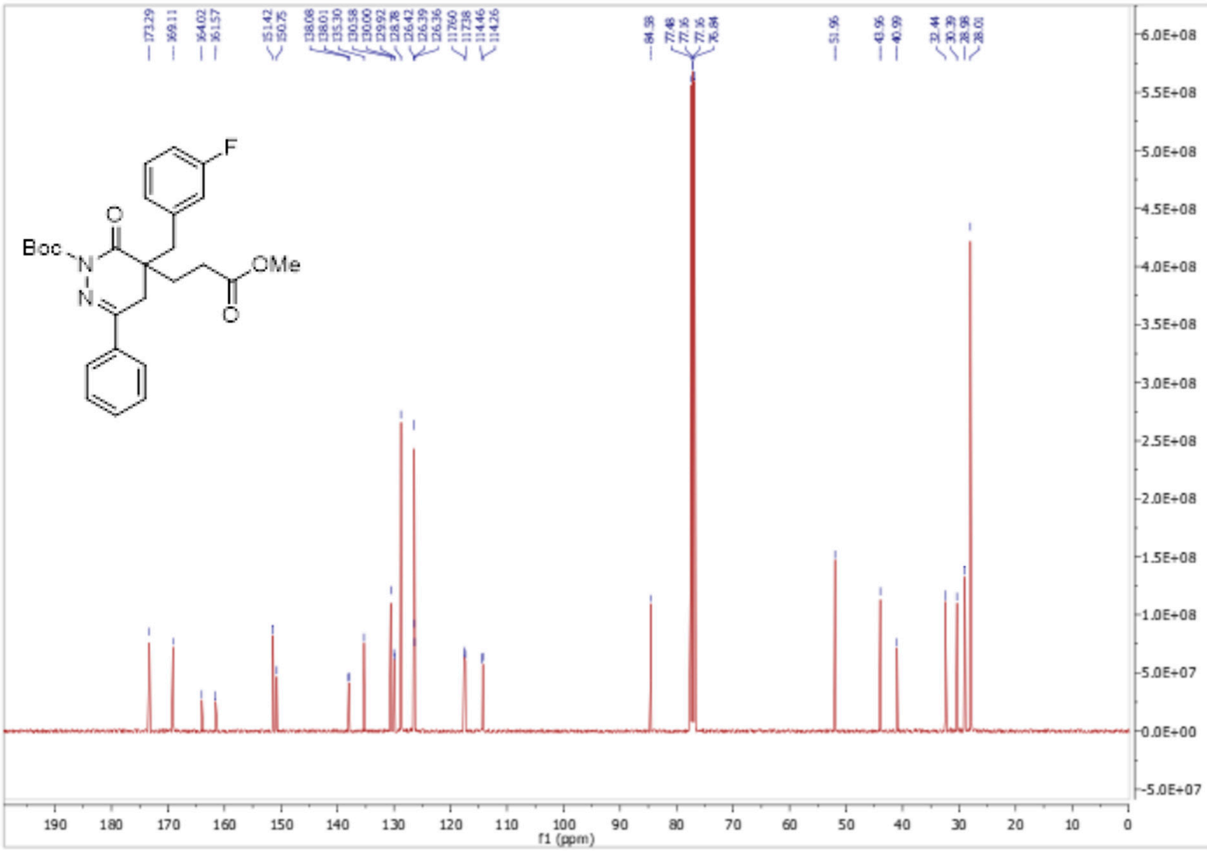
173.29
169.11
164.02
161.57
151.42
150.75
138.08
138.01
135.30
130.58
130.00
129.92
128.78
126.42
126.39
126.36
117.60
117.38
114.46
114.26
84.58
77.48
77.36
77.16
76.84
51.96
43.96
40.99
32.44
30.39
28.98
28.01
F
O
Boc
N
N
OMe
O
f1 (ppm)

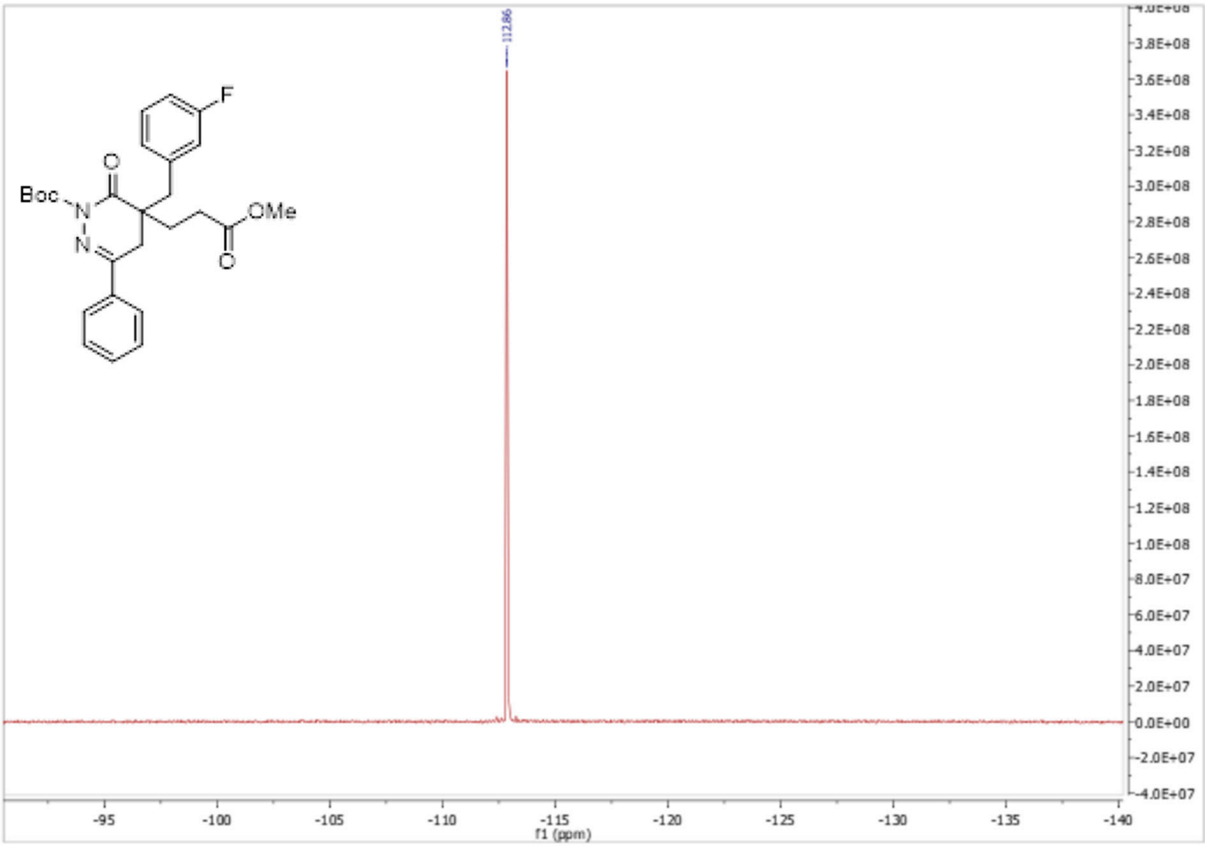
112.86
F
O
Boc
N
N
OMe
O
f1 (ppm)

***tert*-butyl 5-(3-butoxy-3-oxopropyl)-5-(3-fluorobenzyl)-6-oxo-3-phenyl-5,6-dihydropyridazine-1(*4H*)-carboxylate (10b)**

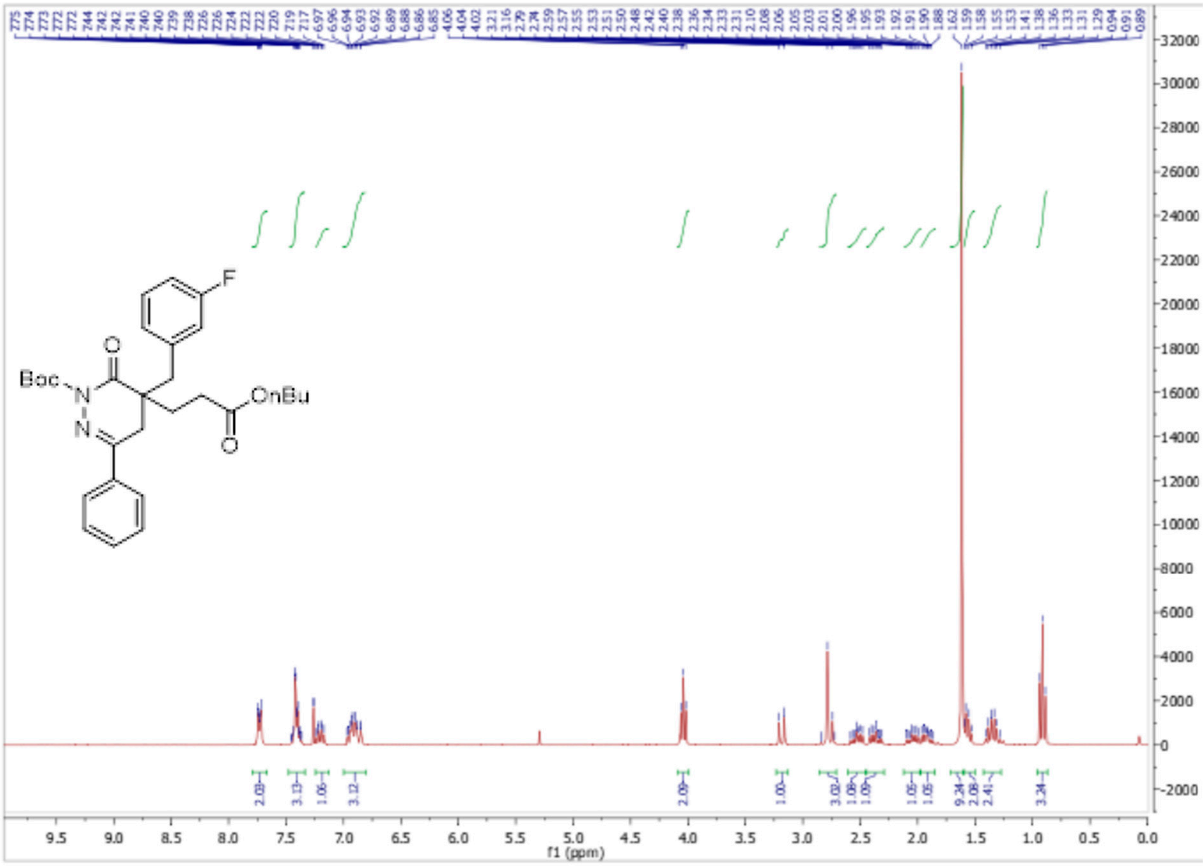

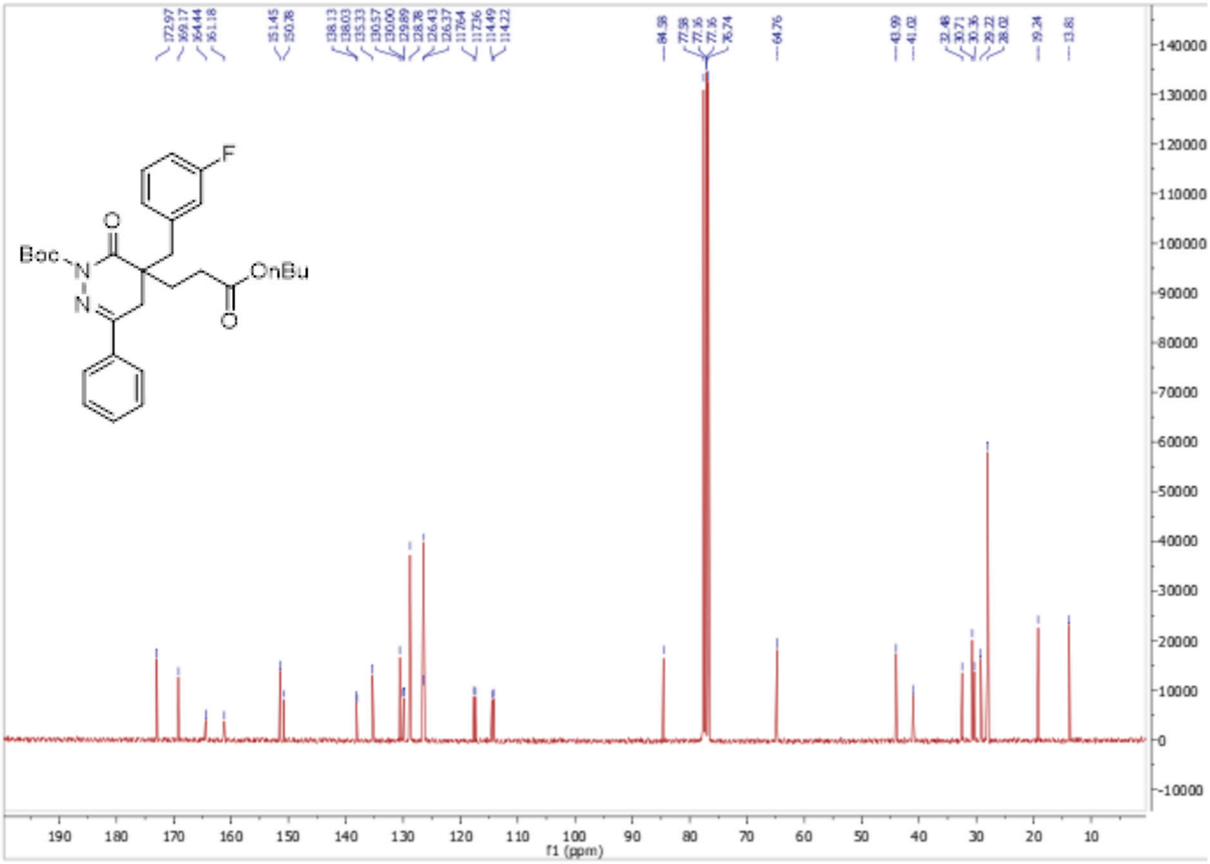

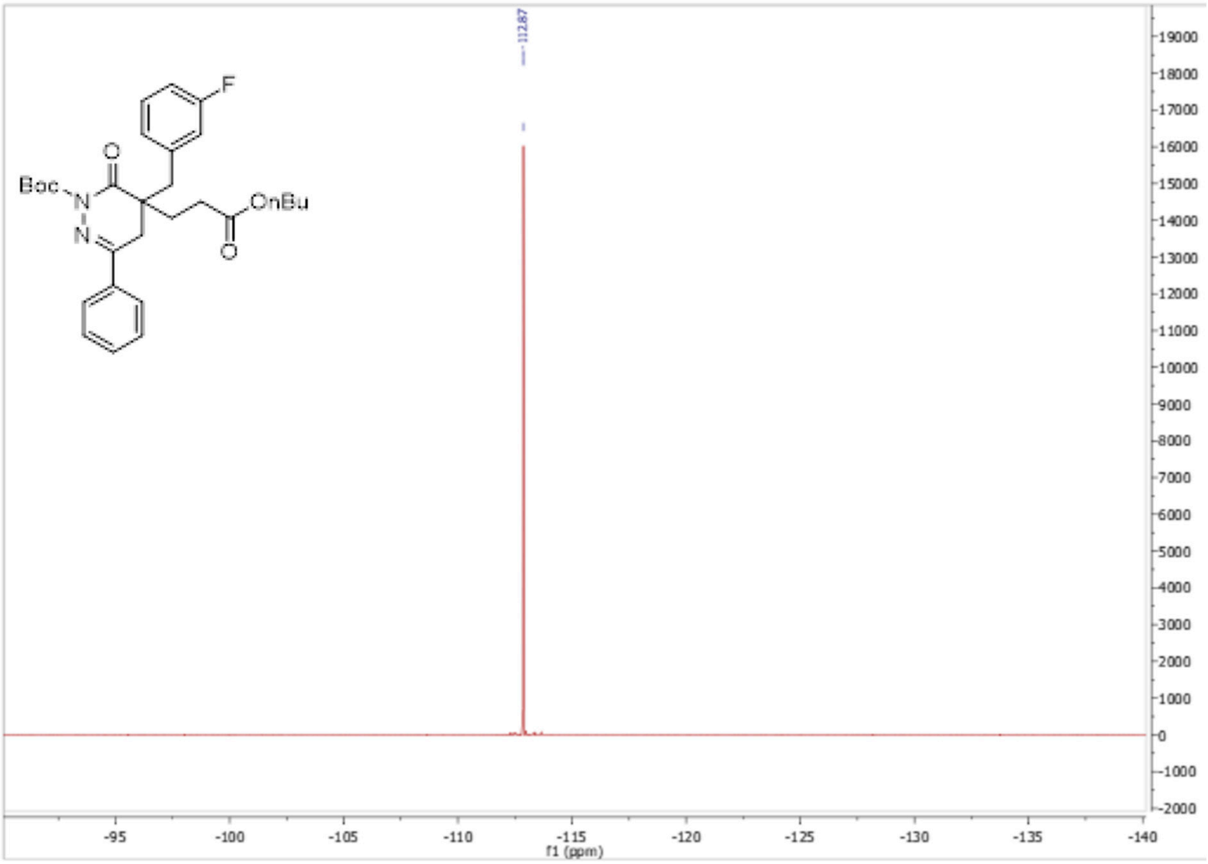

F
O
Boc
N
N
OnBu
O
-112.87
19000
18000
17000
16000
15000
14000
13000
12000
11000
10000
9000
8000
7000
6000
5000
4000
3000
2000
1000
0
-1000
-2000
-95
-100
-105
-110
-115
-120
-125
-130
-135
-140
f1 (ppm)

***tert*-butyl 5-(3-(tert-butoxy)-3-oxopropyl)-5-(3-fluorobenzyl)-6-oxo-3-phenyl-5,6-dihydropyridazine-1(*4H*)-carboxylate (10c)**

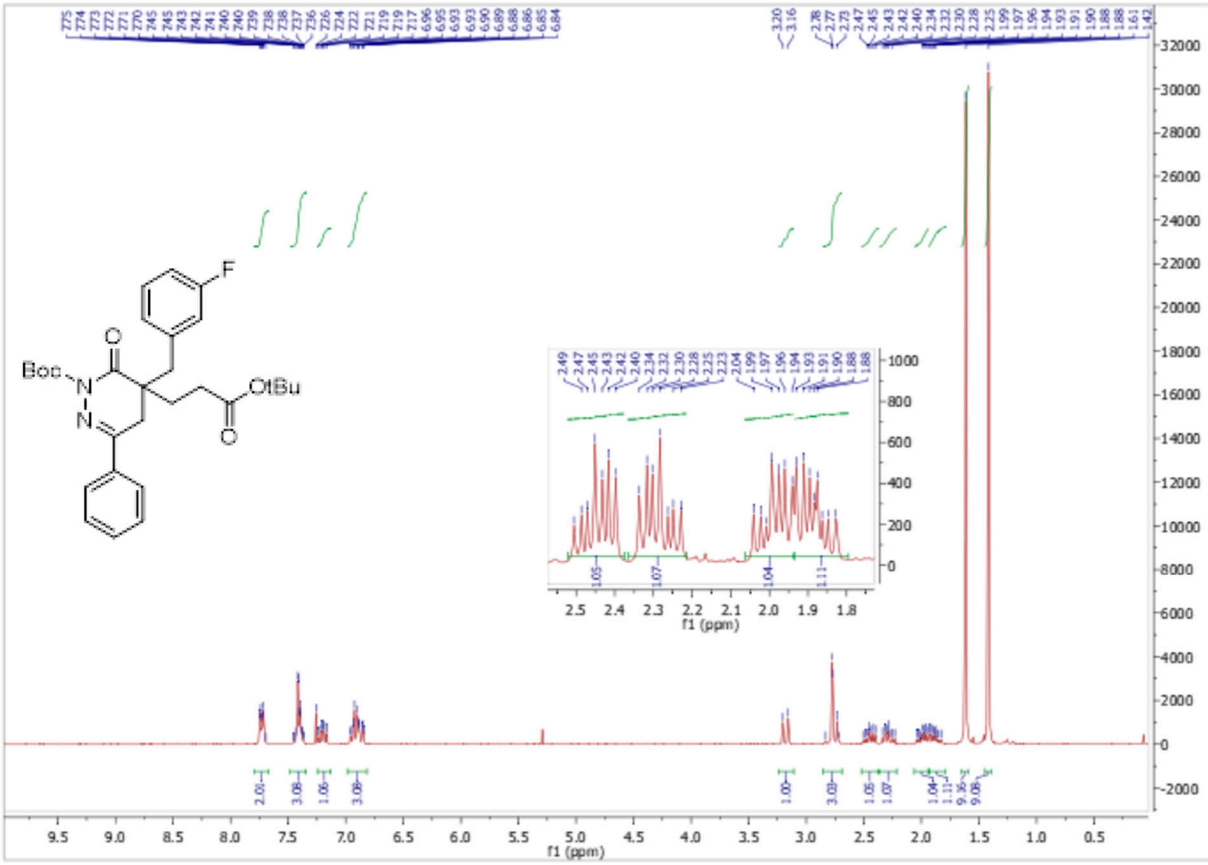

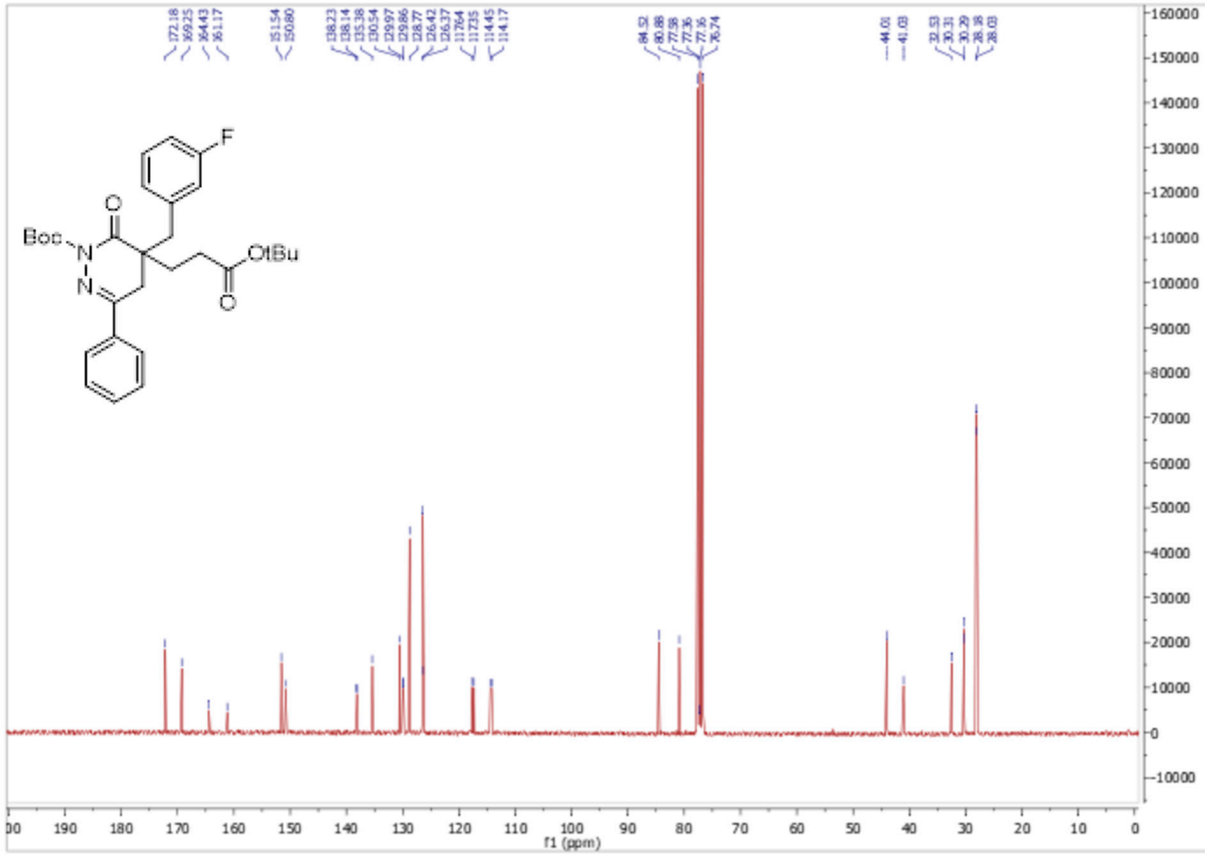

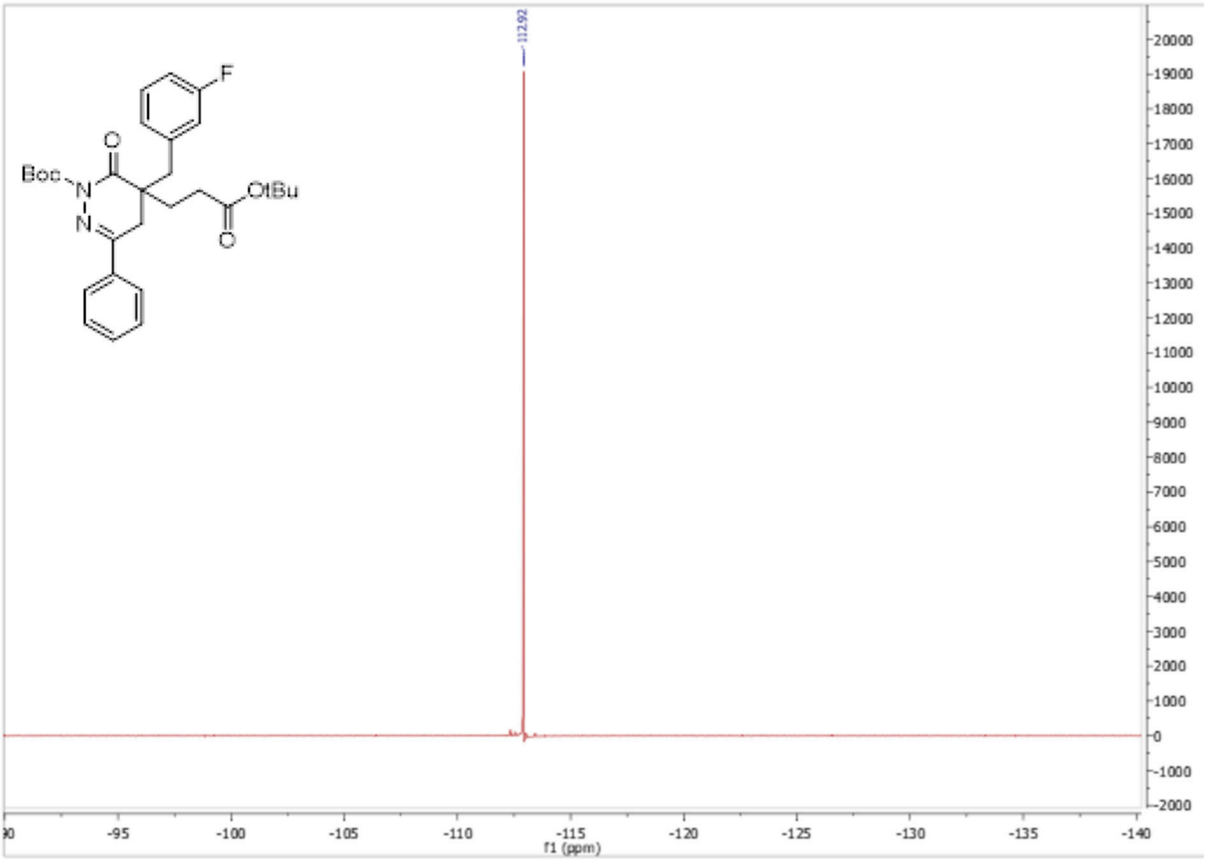

F
O
Boc
N
N
OtBu
O
-112.92
20000
19000
18000
17000
16000
15000
14000
13000
12000
11000
10000
9000
8000
7000
6000
5000
4000
3000
2000
1000
0
-1000
-2000
-95
-100
-105
-110
-115
-120
-125
-130
-135
-140
f1 (ppm)

***tert*-butyl 5-(3-(dimethylamino)-3-oxopropyl)-5-(3-fluorobenzyl)-6-oxo-3-phenyl-5,6-dihydropyridazine-1(*4H*)-carboxylate (10d)**

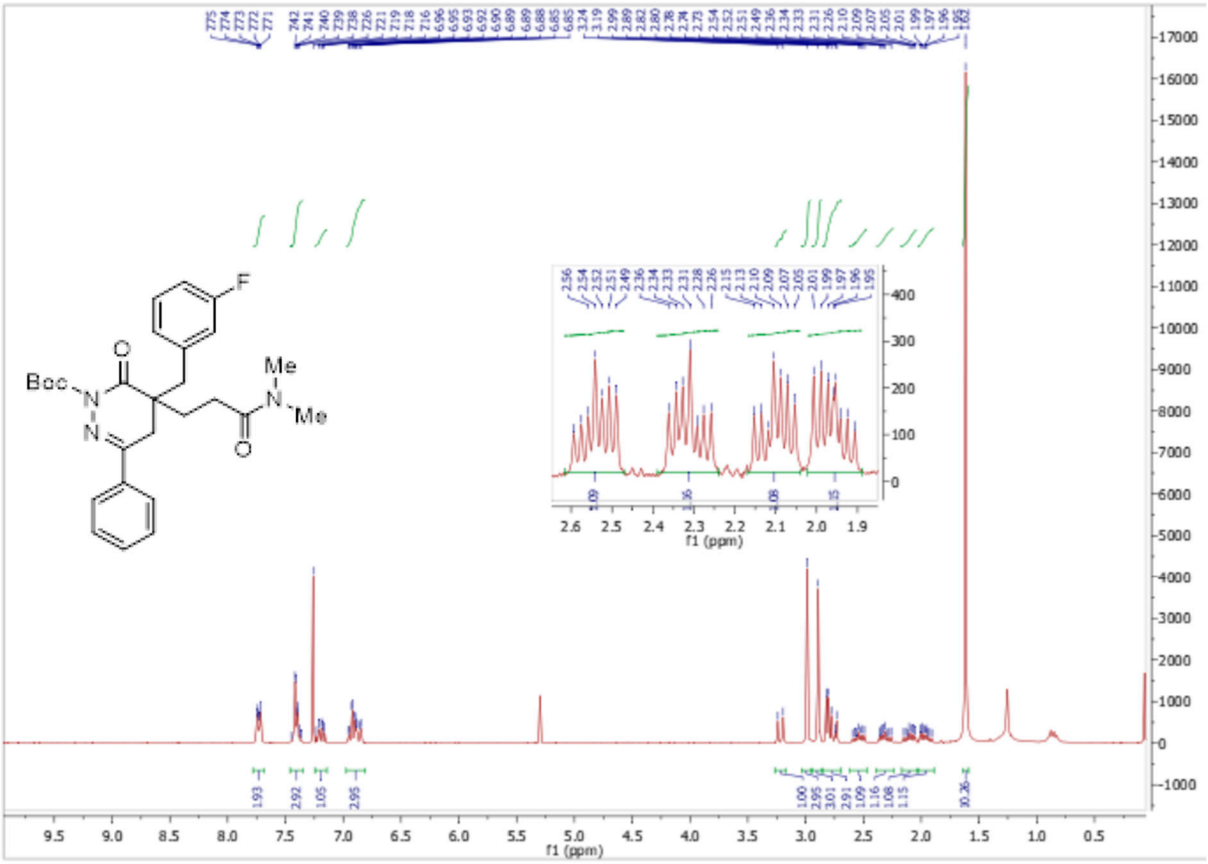

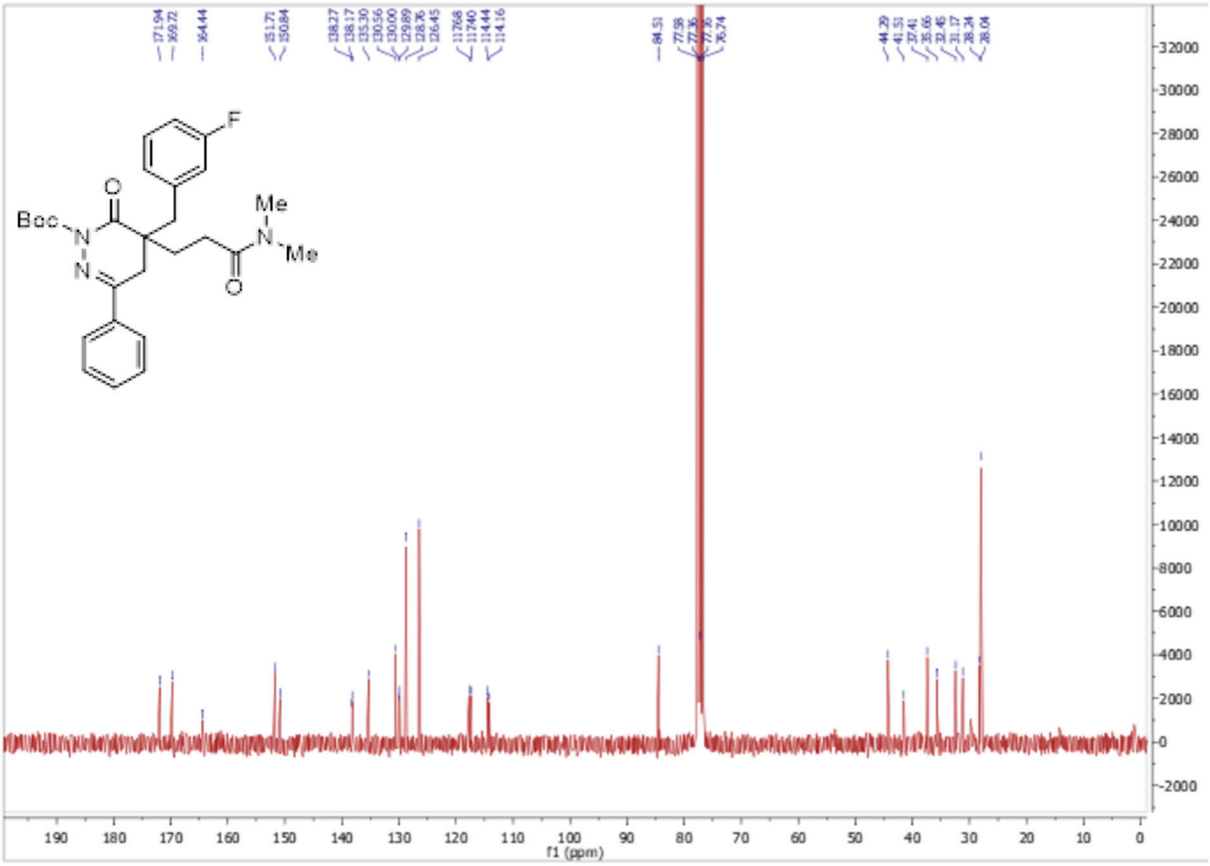

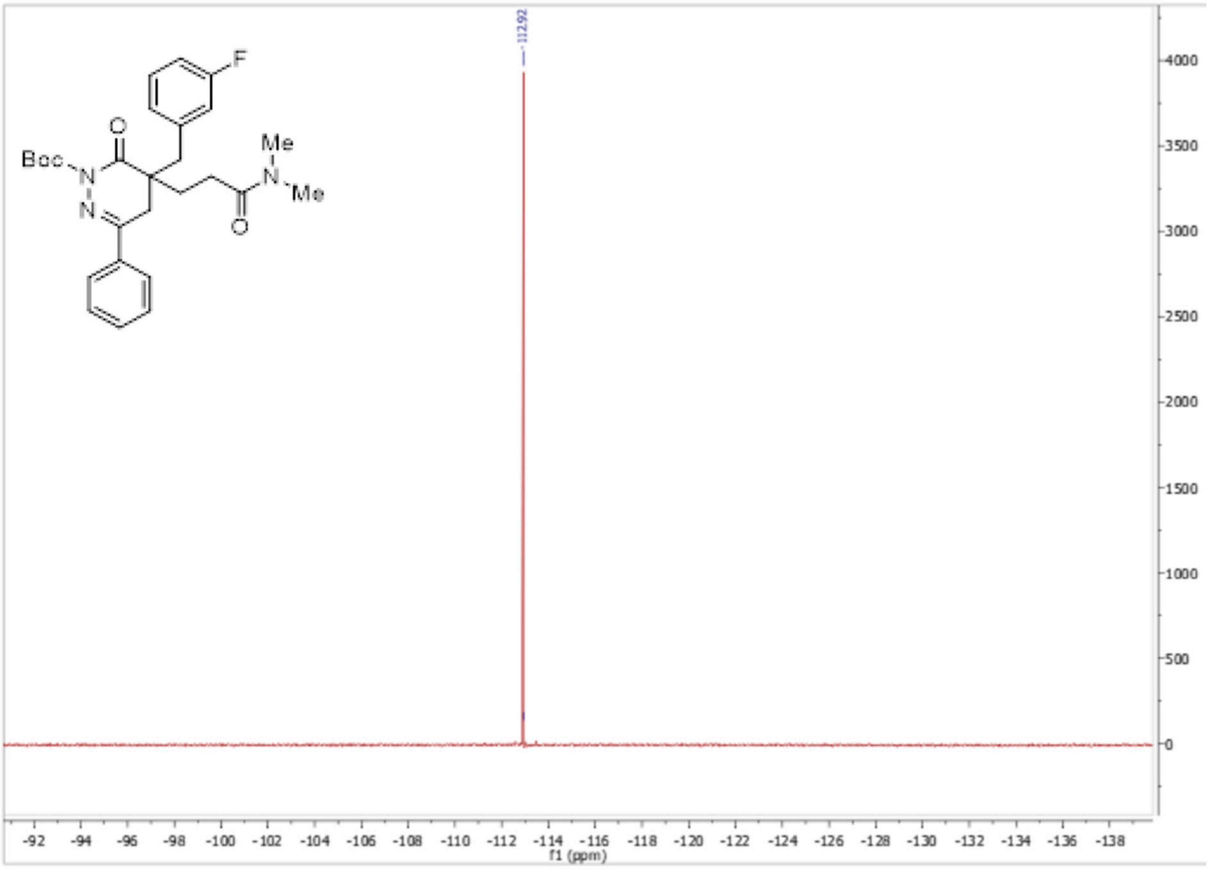
-112.92
Boc
N
N
O
F
Me
N
Me
O
4000
3500
3000
2500
2000
1500
1000
500
0
-92 -94 -96 -98 -100 -102 -104 -106 -108 -110 -112 -114 -116 -118 -120 -122 -124 -126 -128 -130 -132 -134 -136 -138
f1 (ppm)

***tert*-butyl 5-benzyl-5-(3-methoxy-3-oxopropyl)-6-oxo-3-phenyl-5,6-dihydropyridazine-1(*4H*)-carboxylate (10e)**

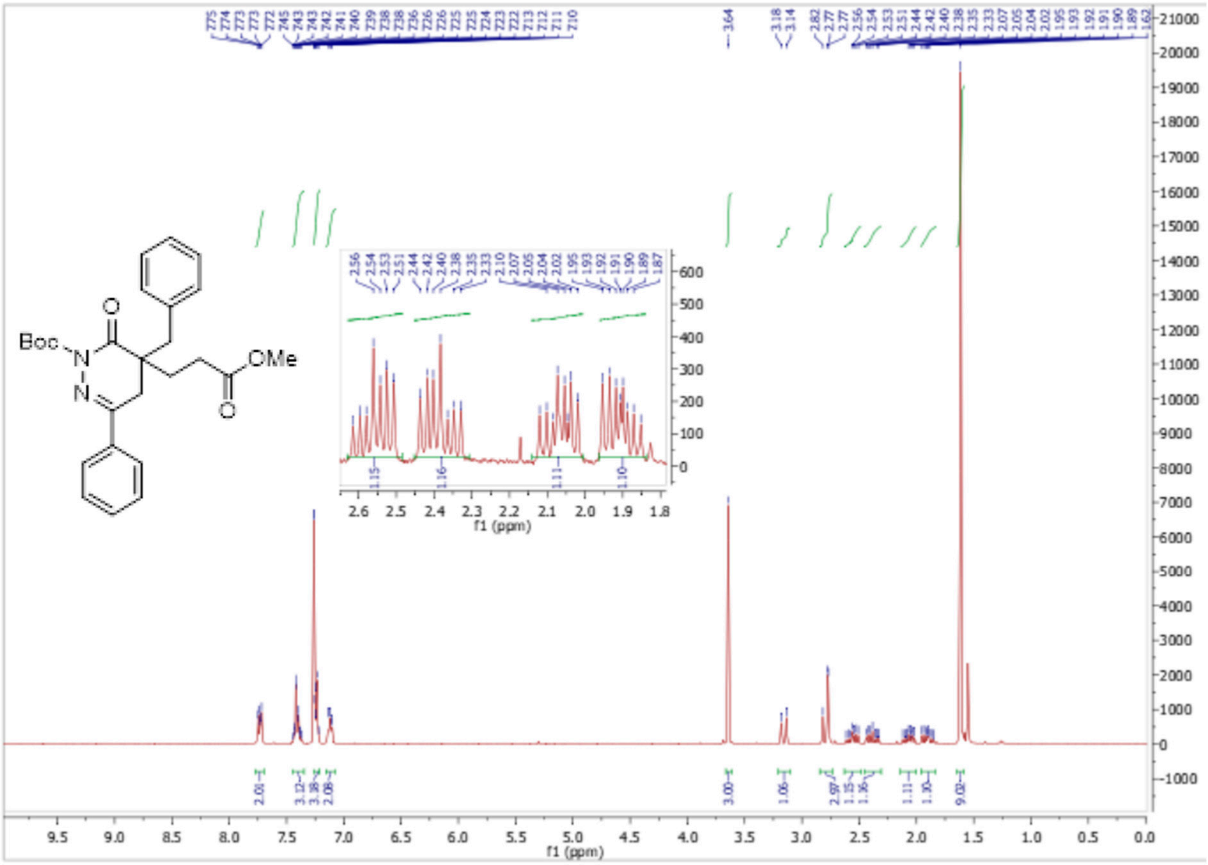

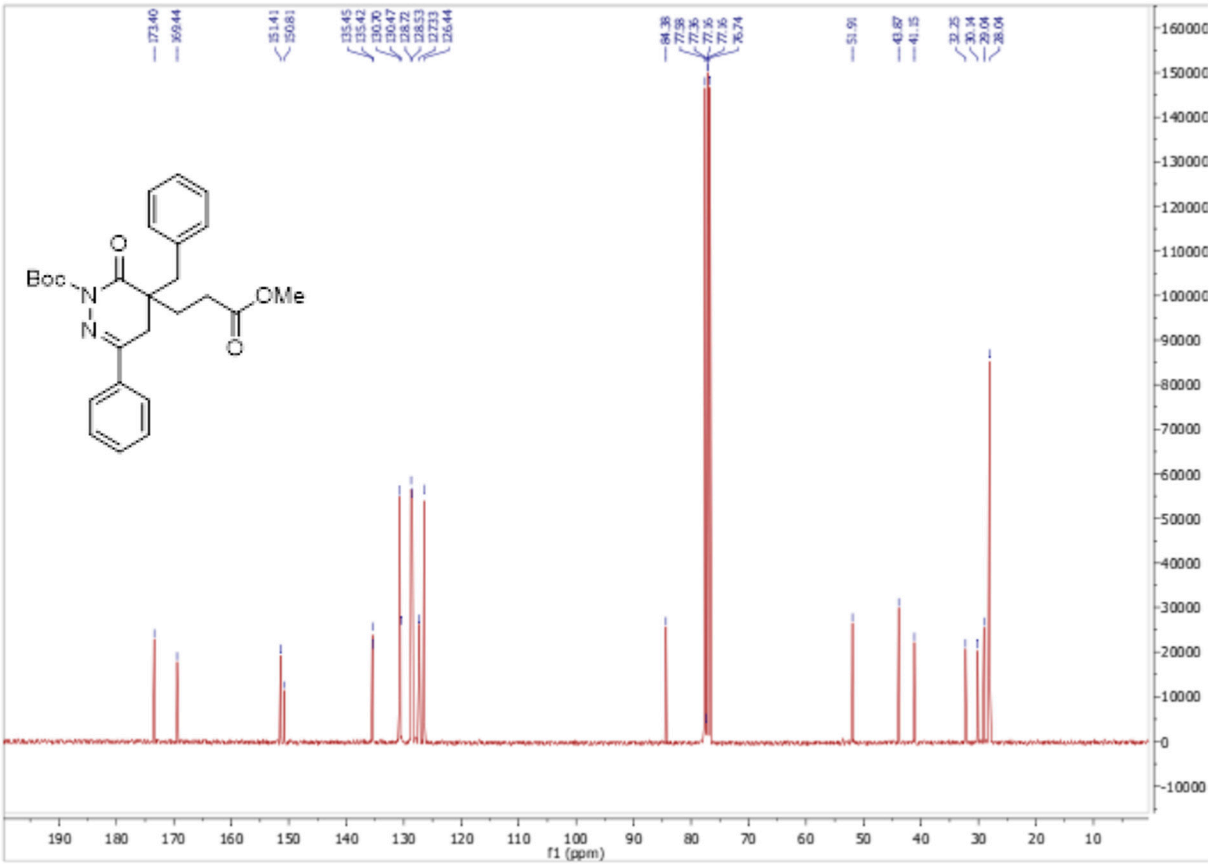

***tert*-butyl 5-(3-methoxy-3-oxopropyl)-5-(naphthalen-1-ylmethyl)-6-oxo-3-phenyl-5,6-dihydropyridazine-1(*4H*)-carboxylate (10f)**

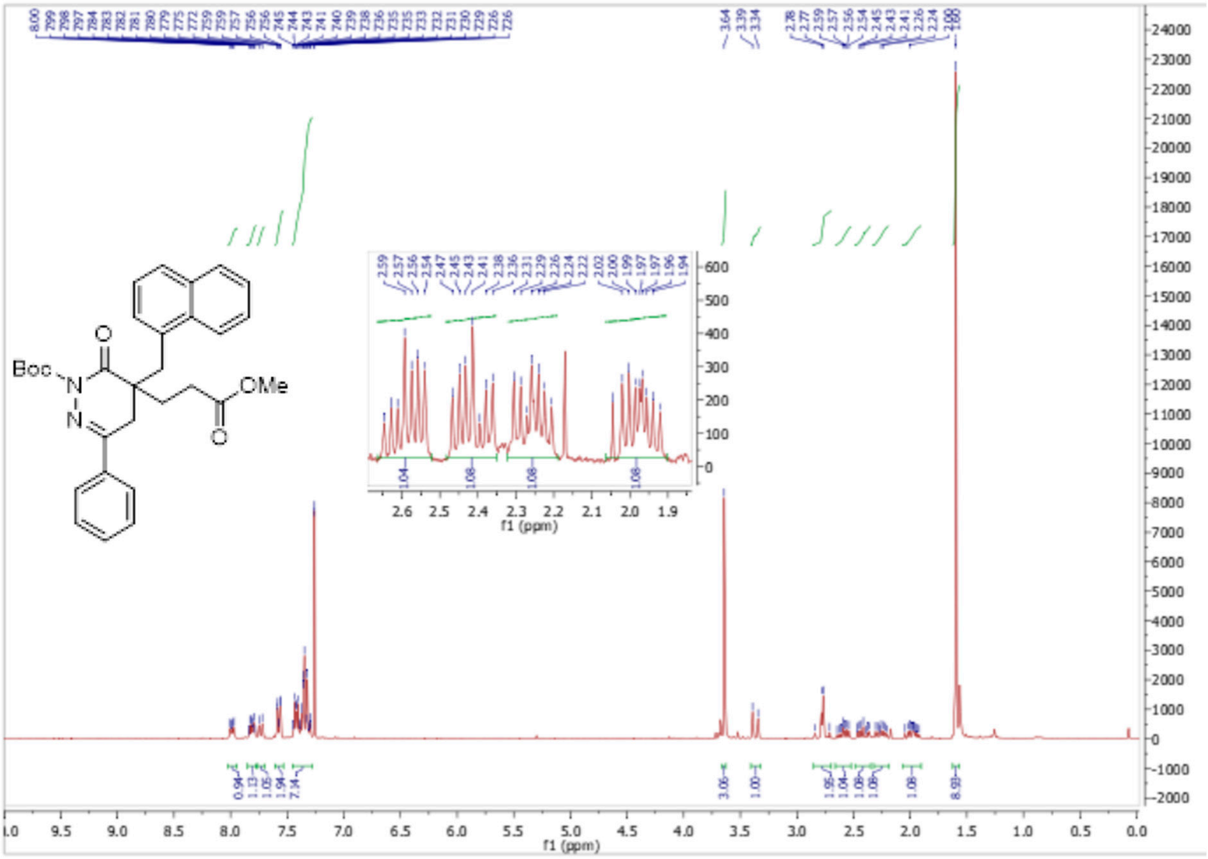

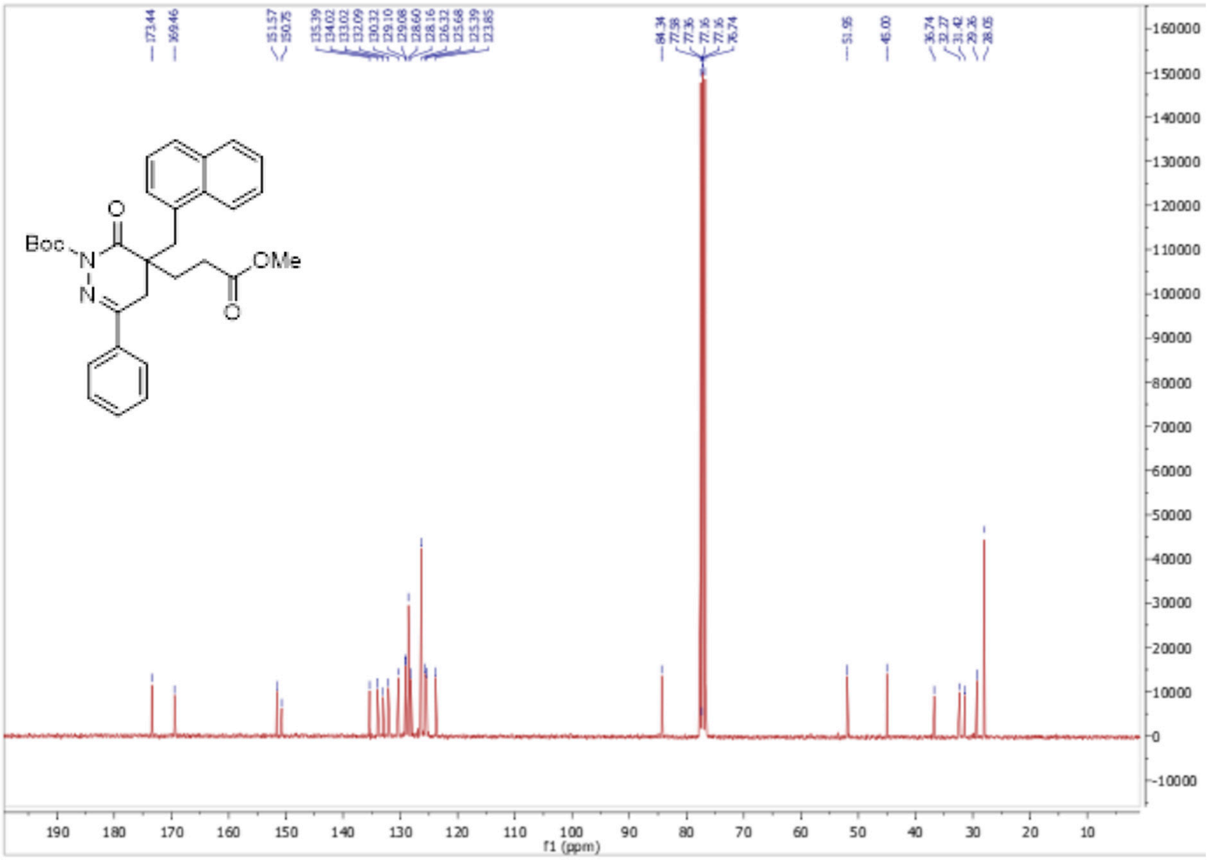

***tert*-butyl 5-(4-fluorobenzyl)-5-(3-methoxy-3-oxopropyl)-6-oxo-3-phenyl-5,6-dihydropyridazine-1(*4H*)-carboxylate** (**10g**)

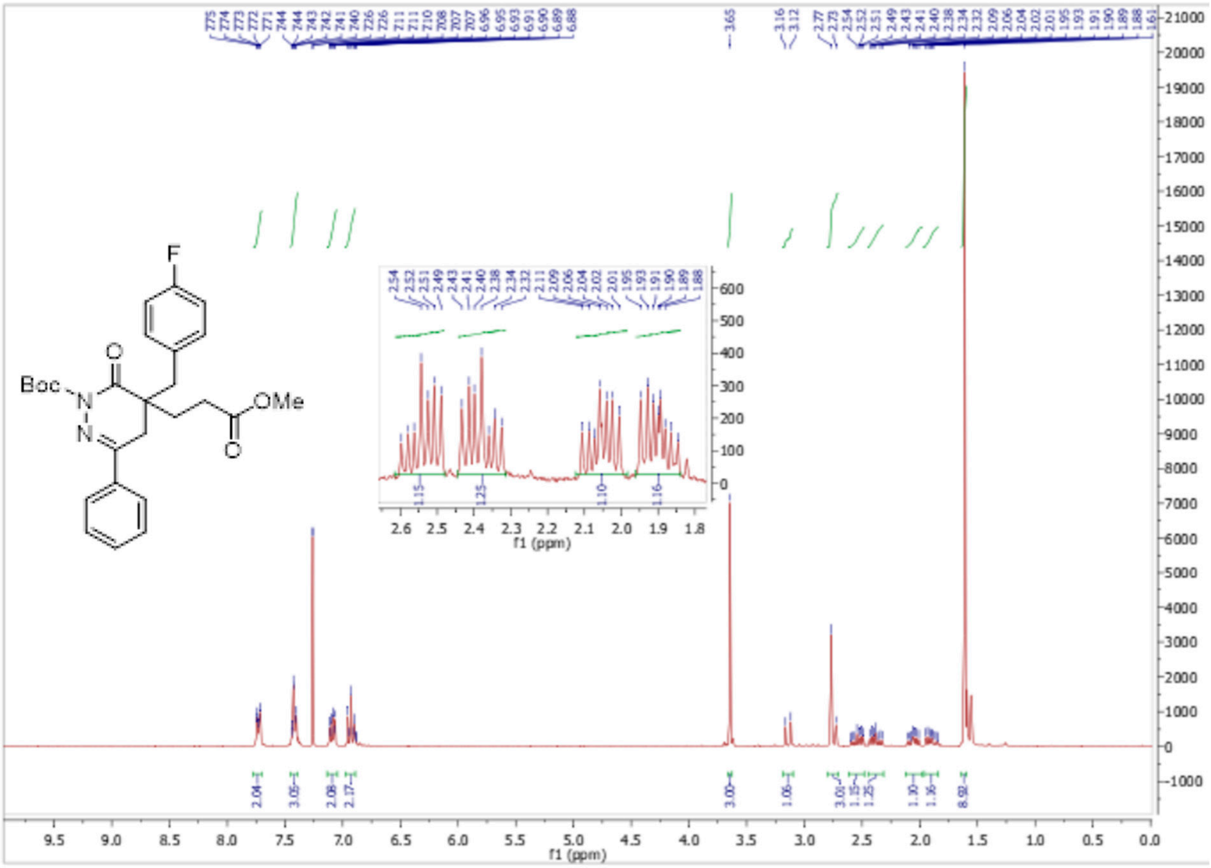

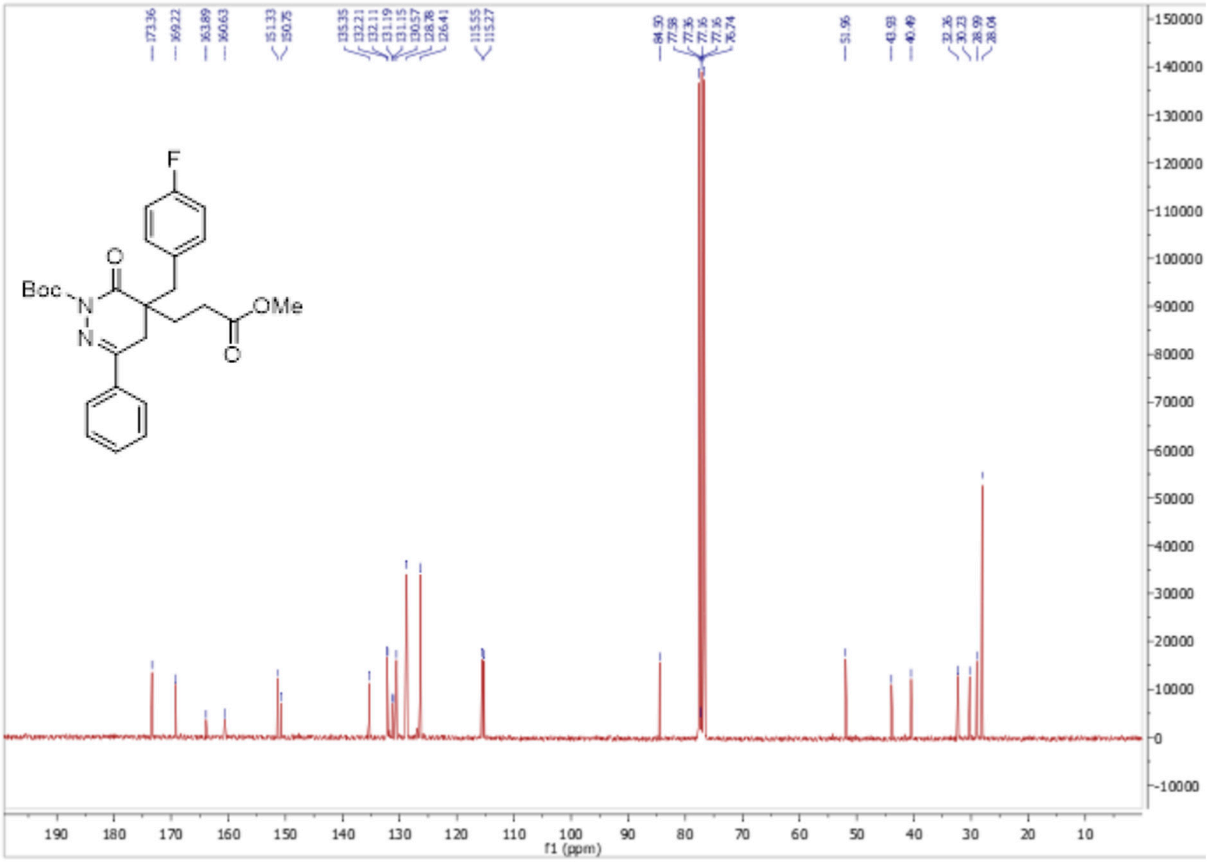

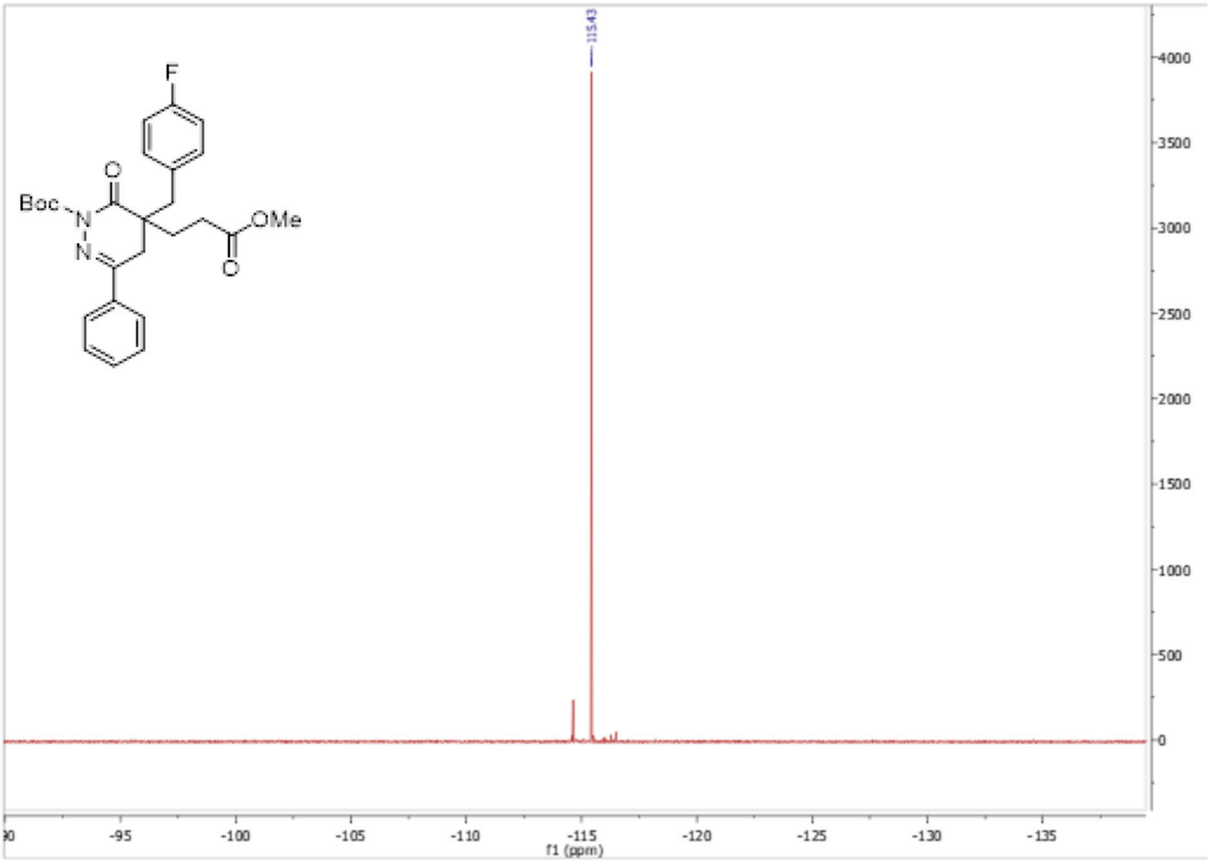
F
O
Boc
N
N
OMe
O
-115.43
f1 (ppm)

***tert*-butyl 5-(4-bromobenzyl)-5-(3-methoxy-3-oxopropyl)-6-oxo-3-phenyl-5,6-dihydropyridazine-1(*4H*)-carboxylate (10h)**

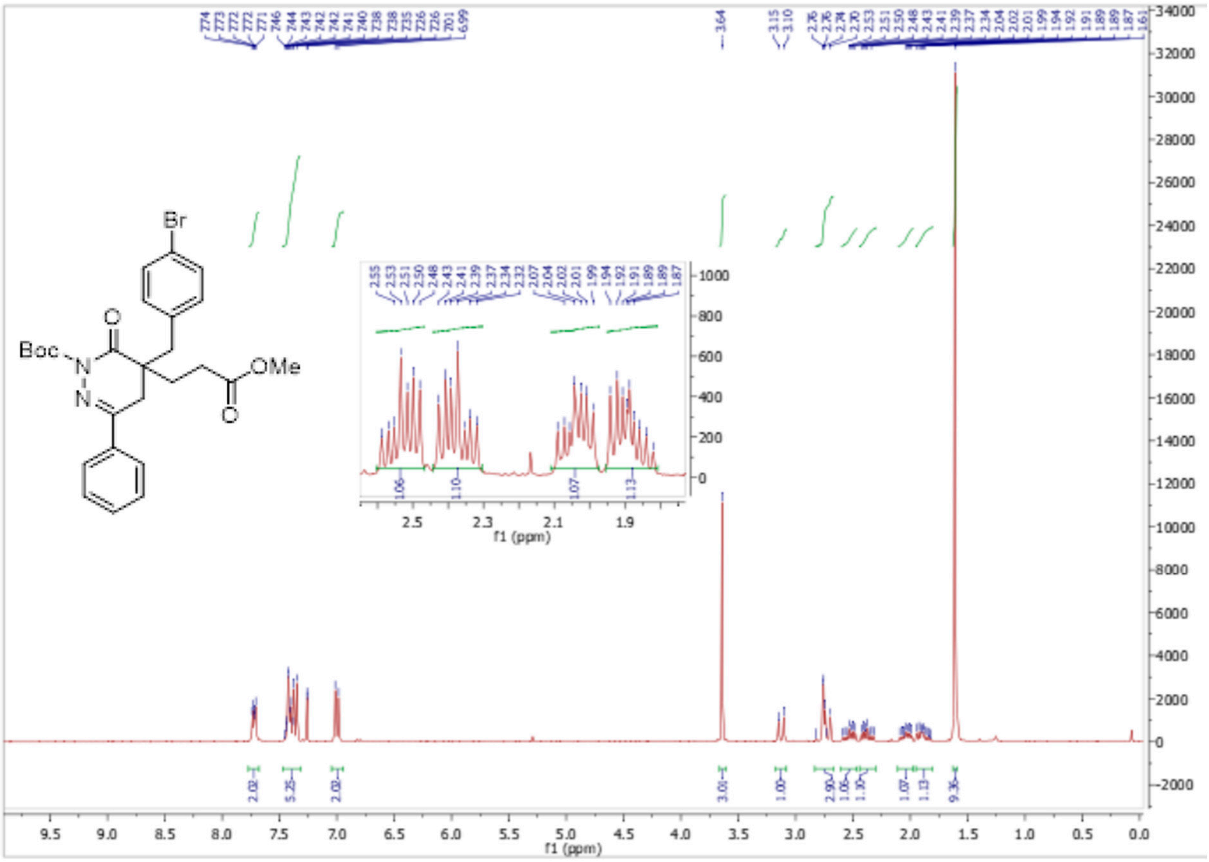

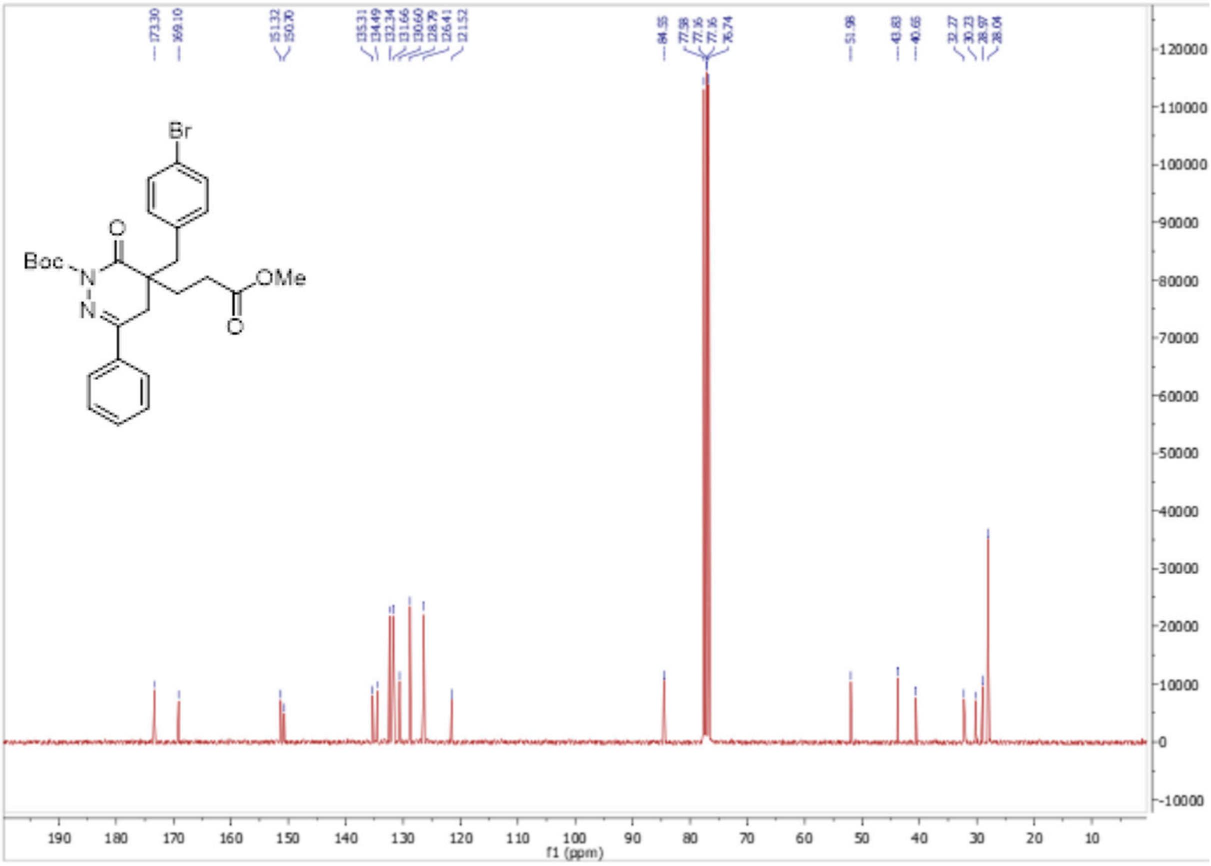

***tert*-butyl 5-(4-chlorobenzyl)-5-(3-methoxy-3-oxopropyl)-6-oxo-3-phenyl-5,6-dihydropyridazine-1(*4H*)-carboxylate (10i)**

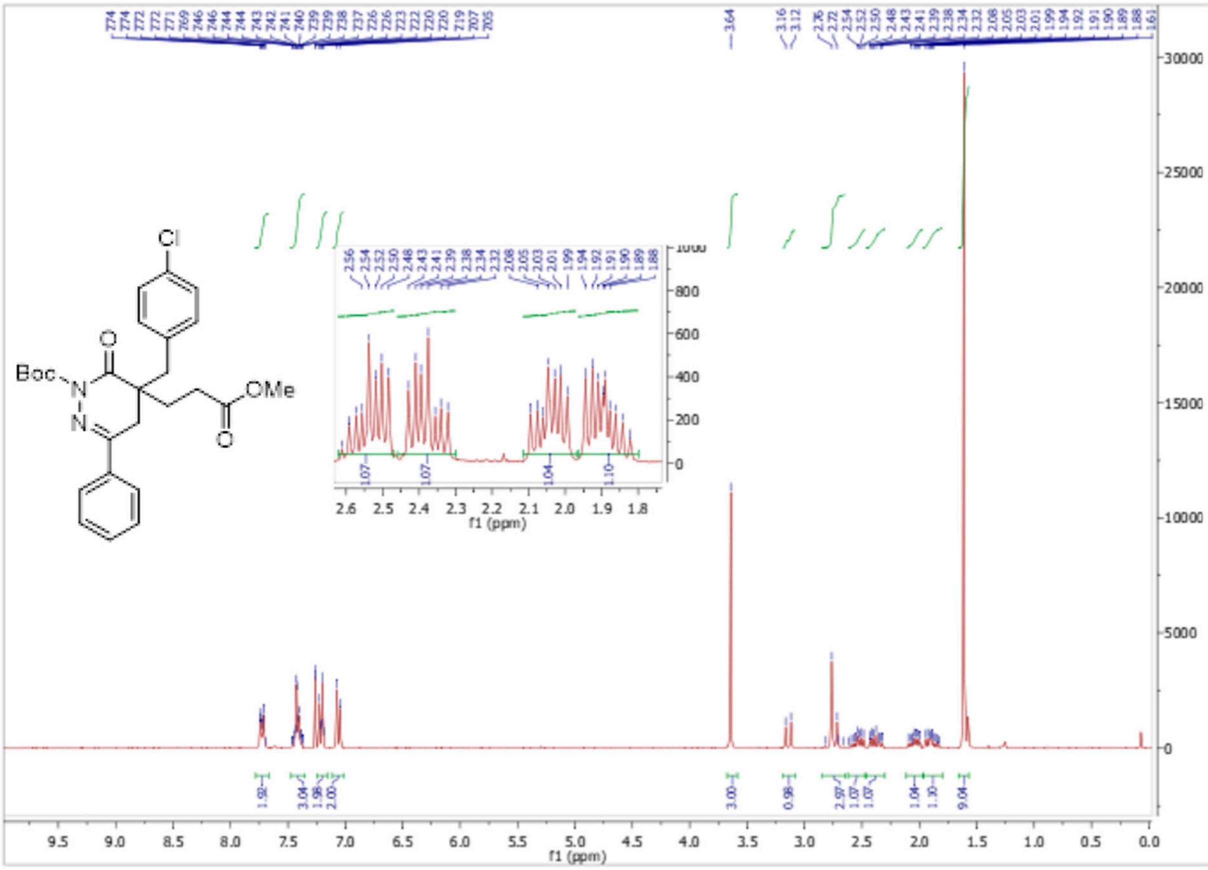

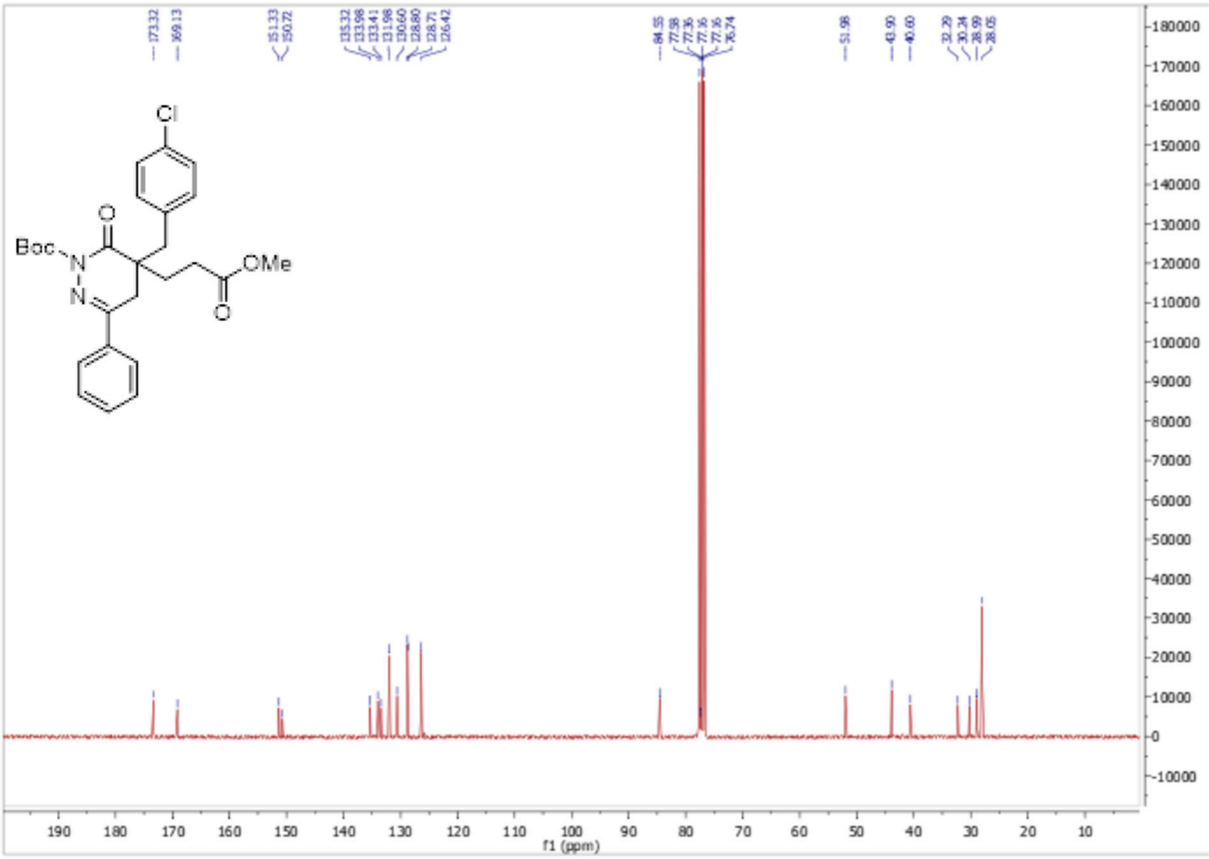

***tert*-butyl 5-(2-cyanobenzyl)-5-(3-methoxy-3-oxopropyl)-6-oxo-3-phenyl-5,6-dihydropyridazine-1(*4H*)-carboxylate (10j)**

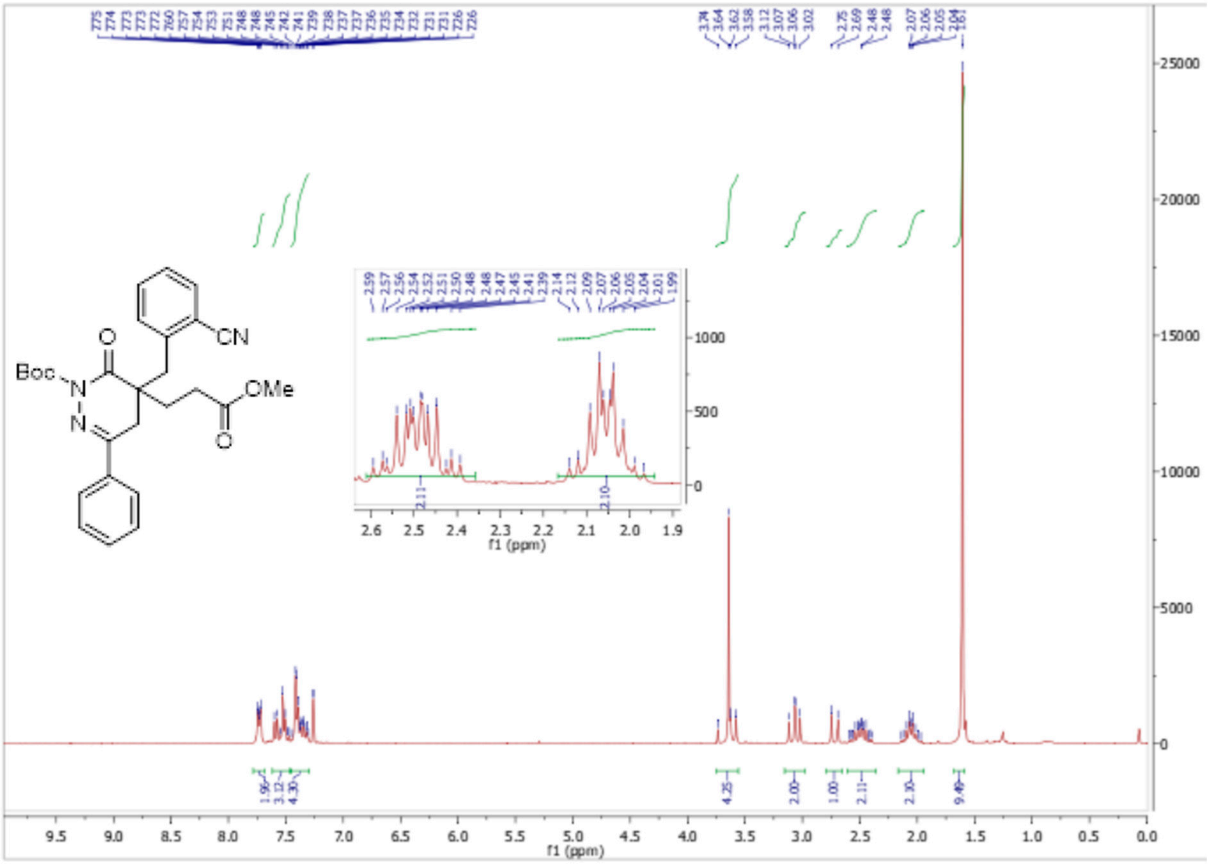

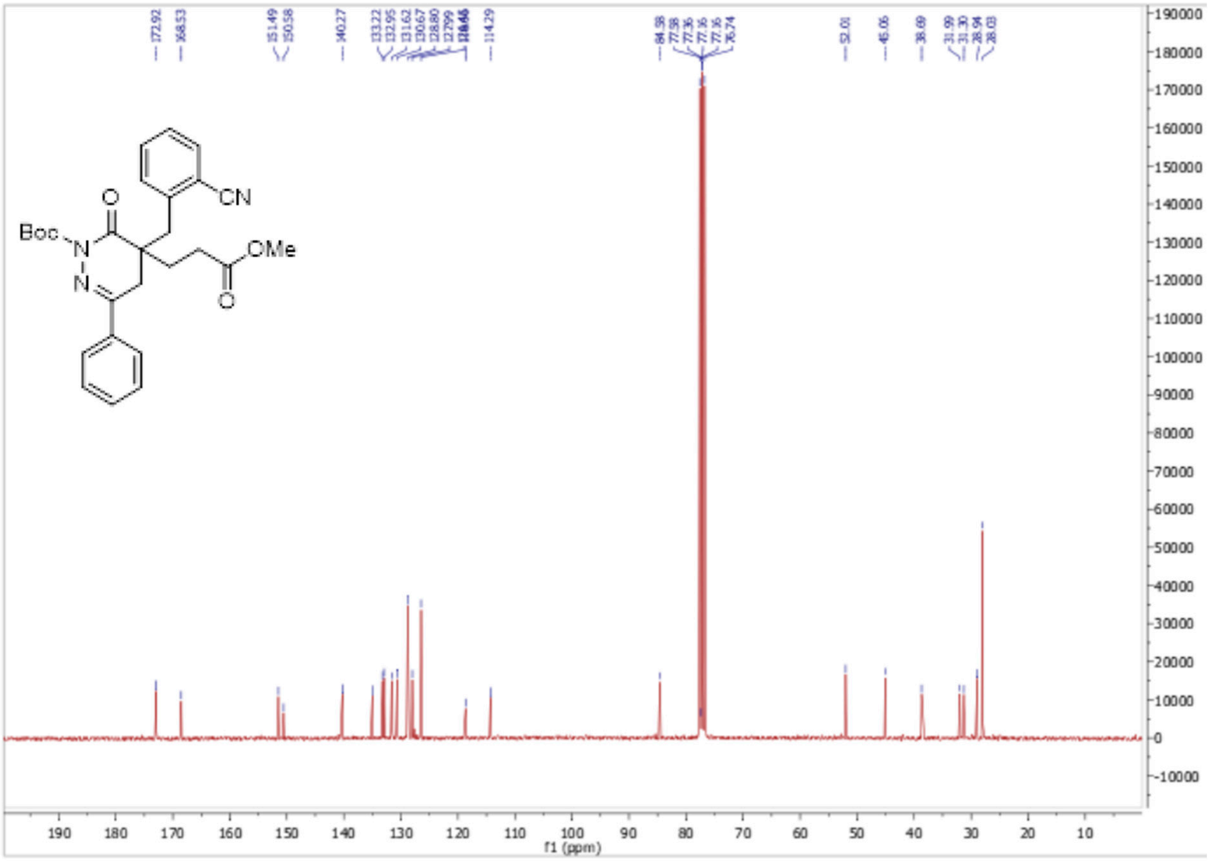

***tert*-butyl 5-(3-methoxy-3-oxopropyl)-5-(2-methoxybenzyl)-6-oxo-3-phenyl-5,6-dihydropyridazine-1(*4H*)-carboxylate (10k)**

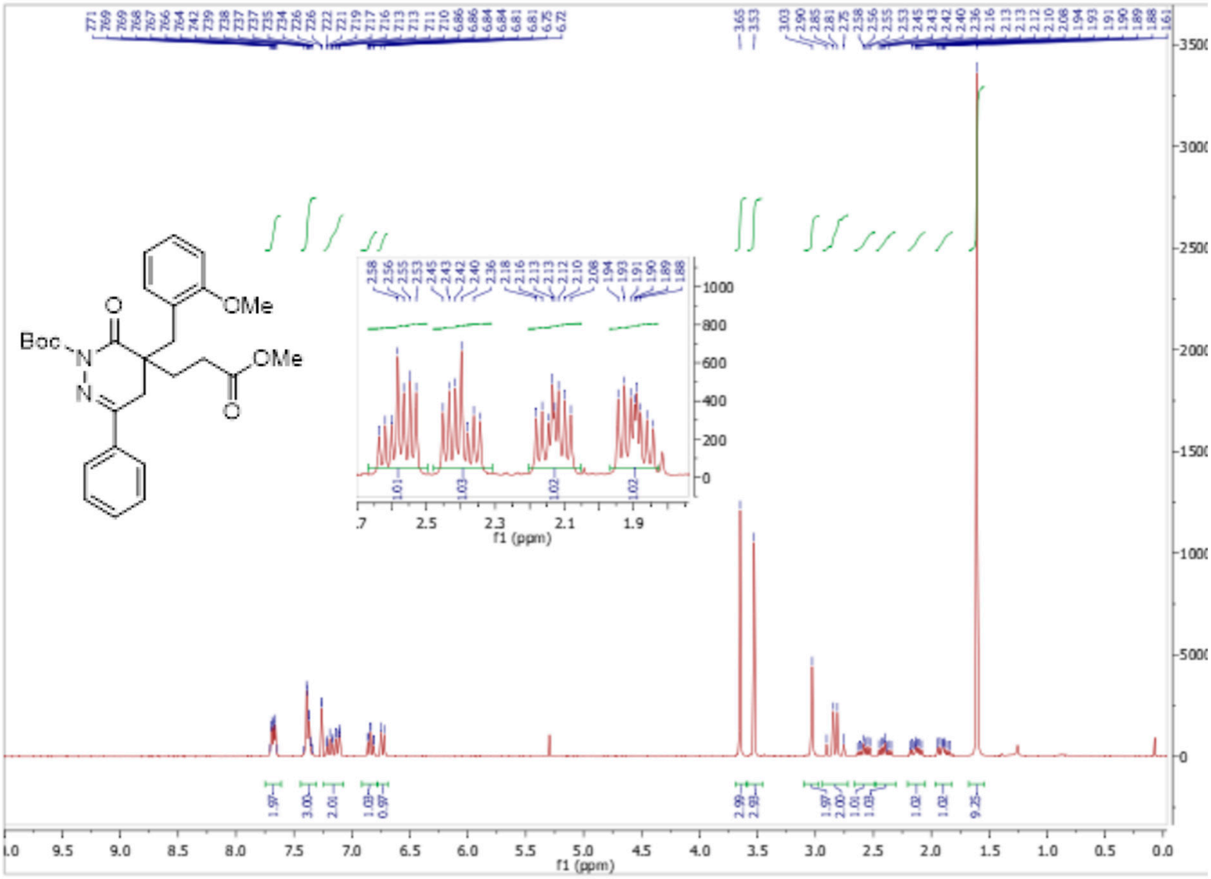

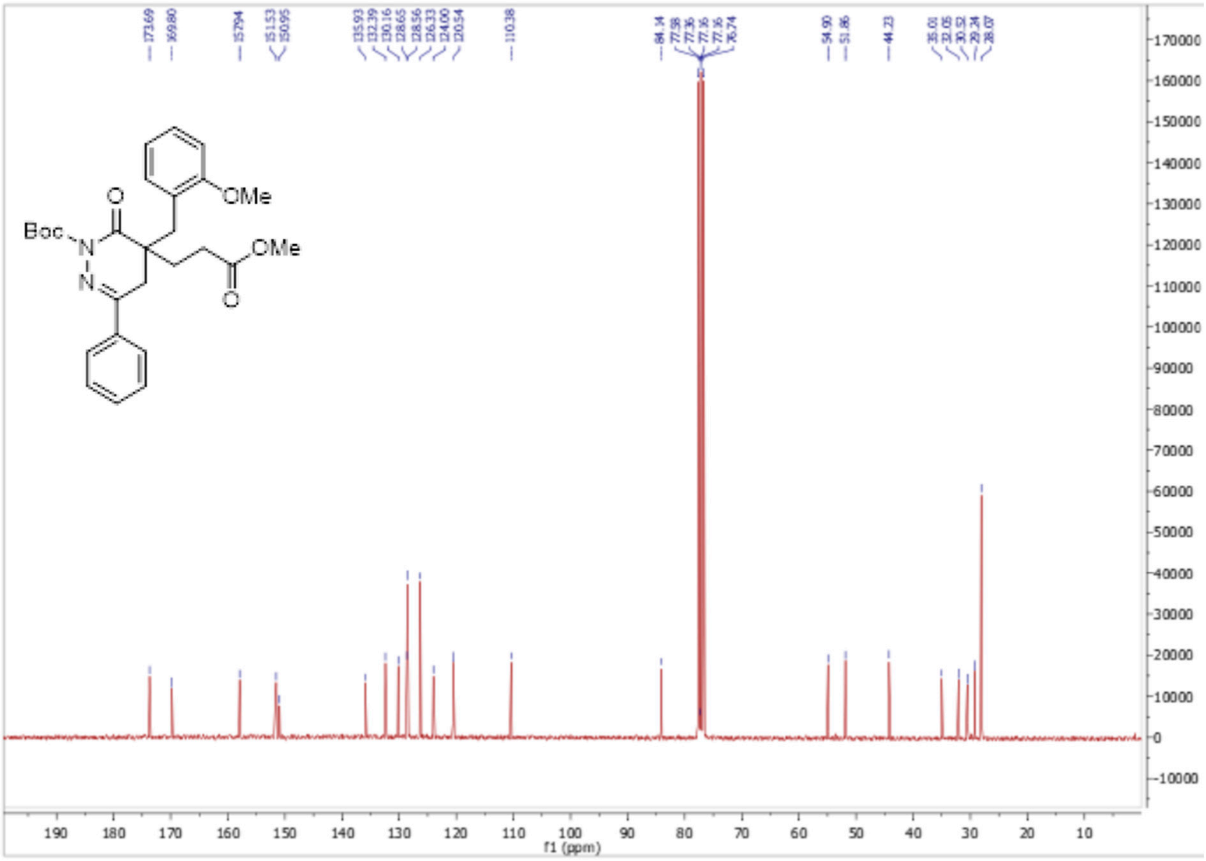

***tert*-butyl 5-(3-methoxy-3-oxopropyl)-5-(4-methylbenzyl)-6-oxo-3-phenyl-5,6-dihydropyridazine-1(*4H*)-carboxylate** (**10l**)

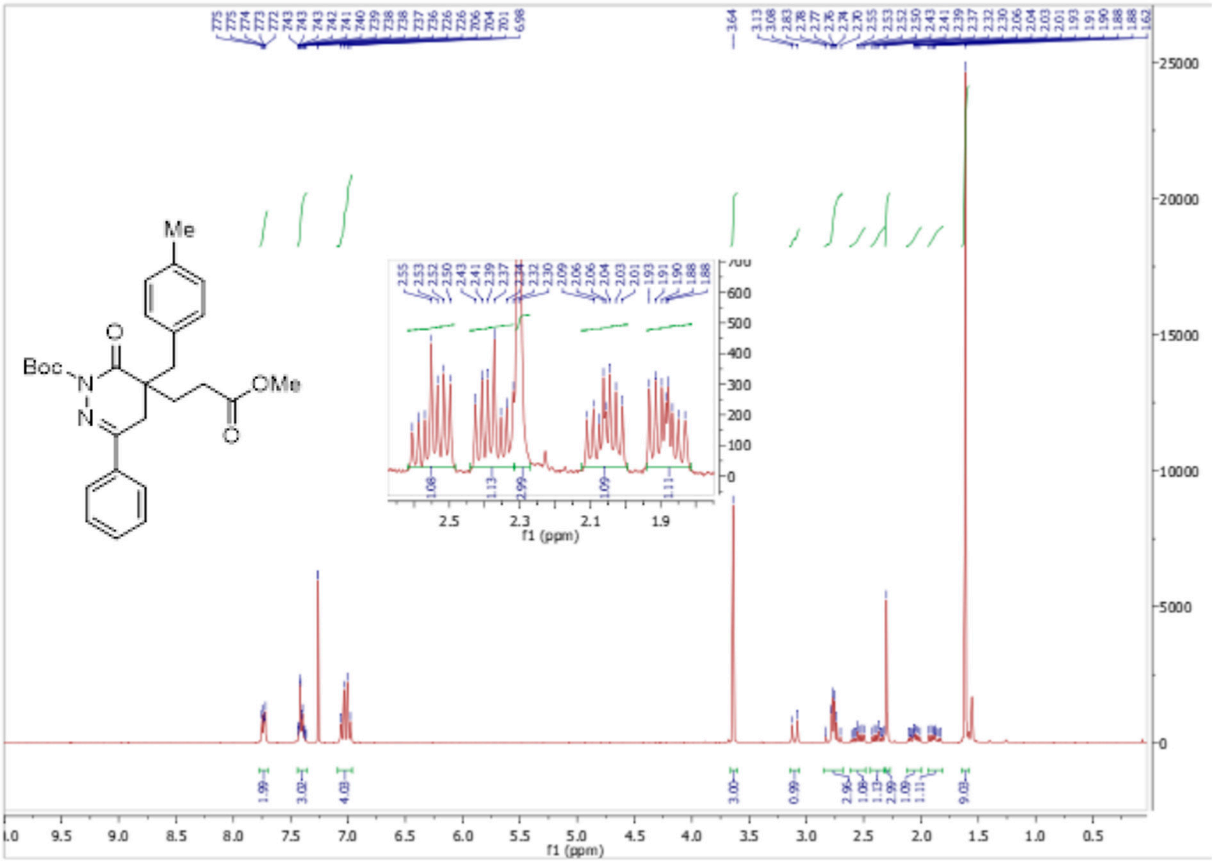

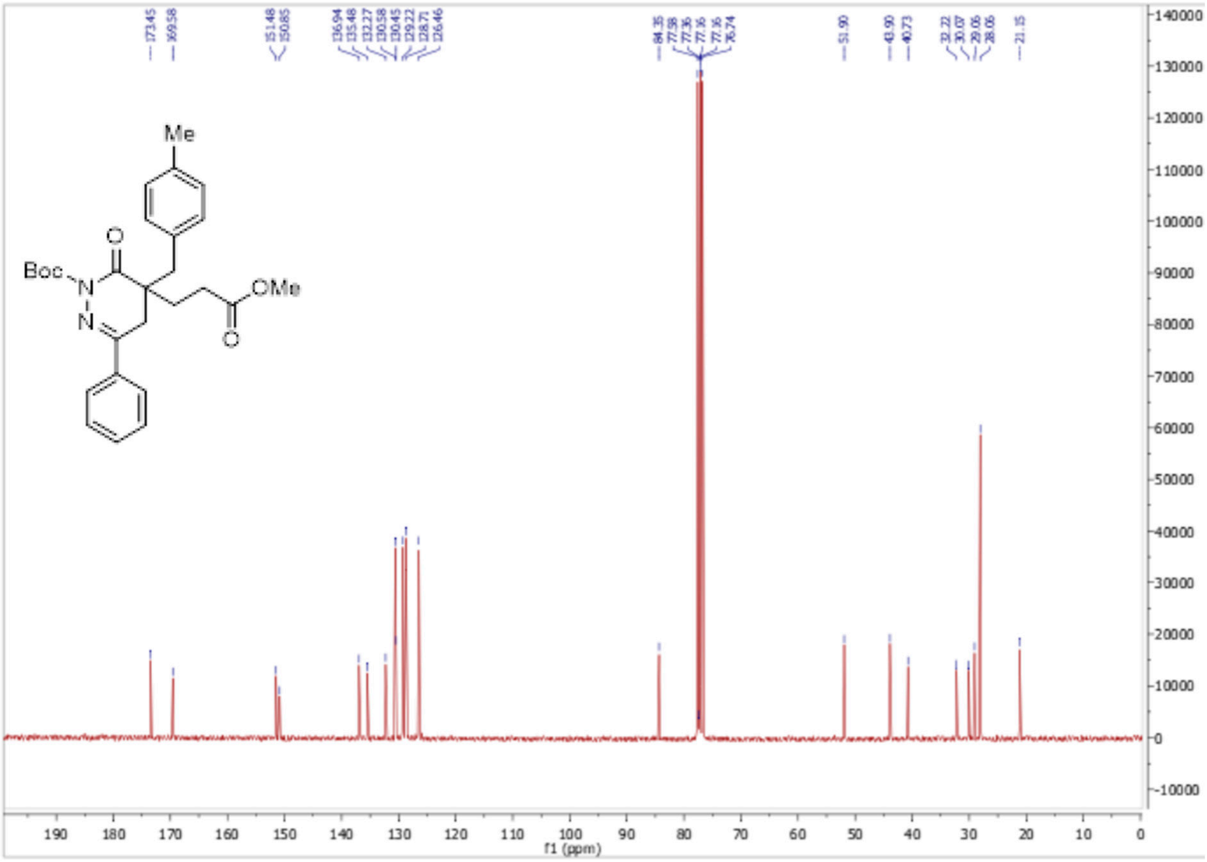

***tert*-butyl 5-(2,5-dimethylbenzyl)-5-(3-methoxy-3-oxopropyl)-6-oxo-3-phenyl-5,6-dihydropyridazine-1(*4H*)-carboxylate (10m)**

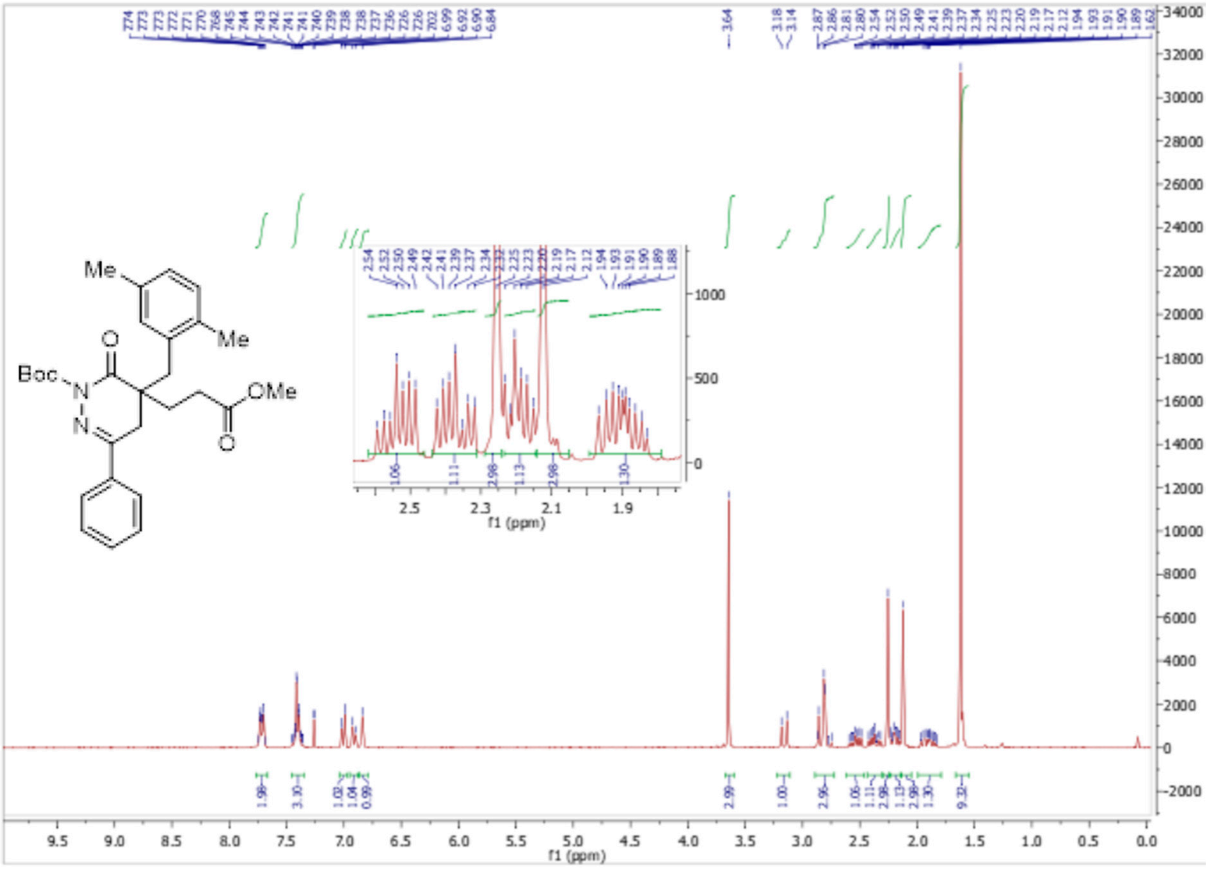

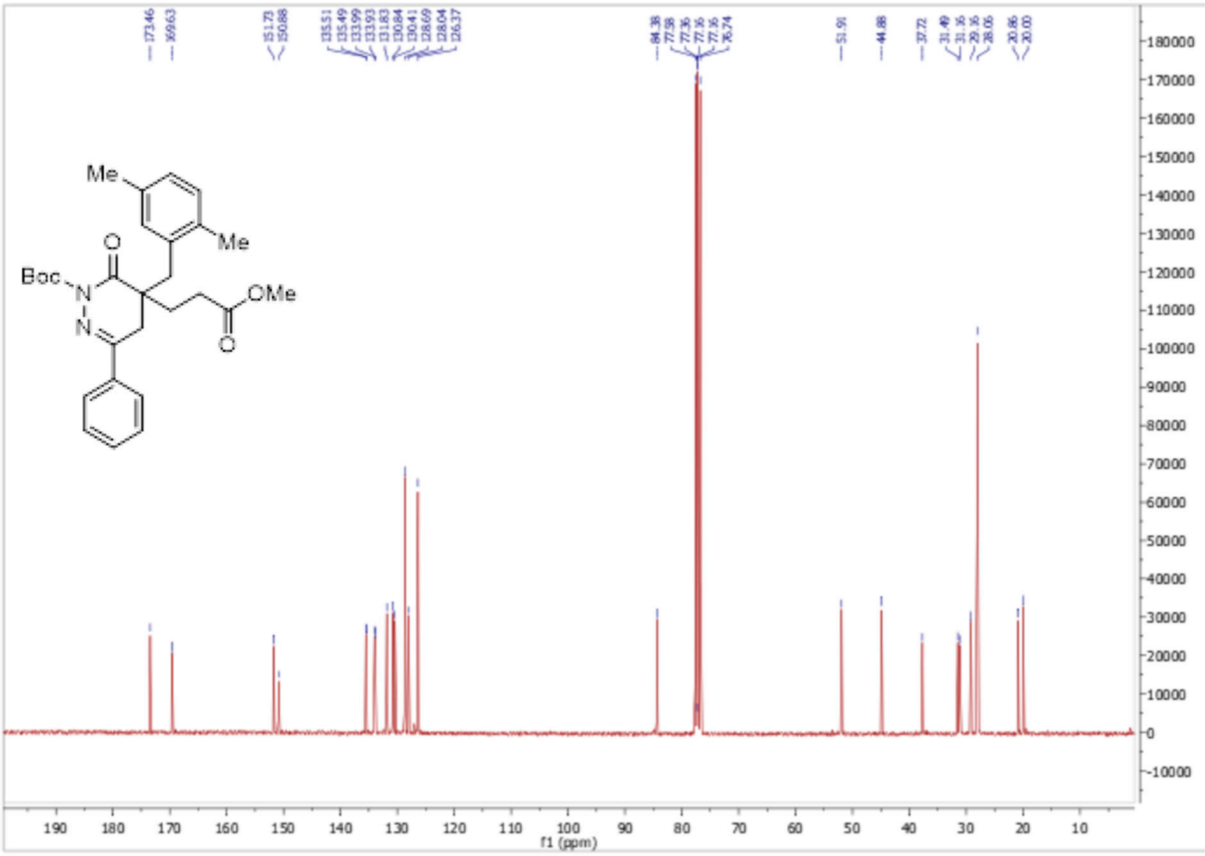

***tert*-butyl 5-(3-methoxy-3-oxopropyl)-6-oxo-3-phenyl-5-(thiophen-2-ylmethyl)-5,6-dihydropyridazine-1(*4H*)-carboxylate** (**10n**)

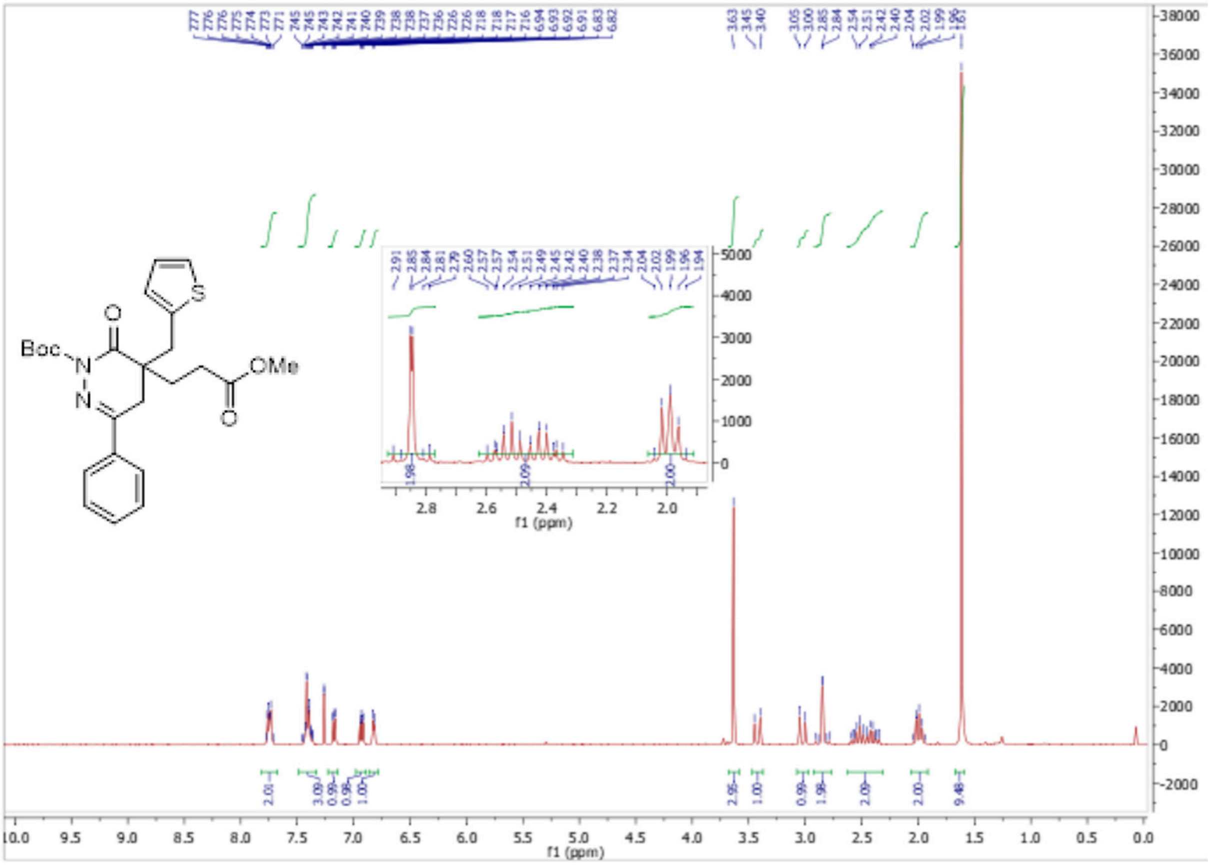

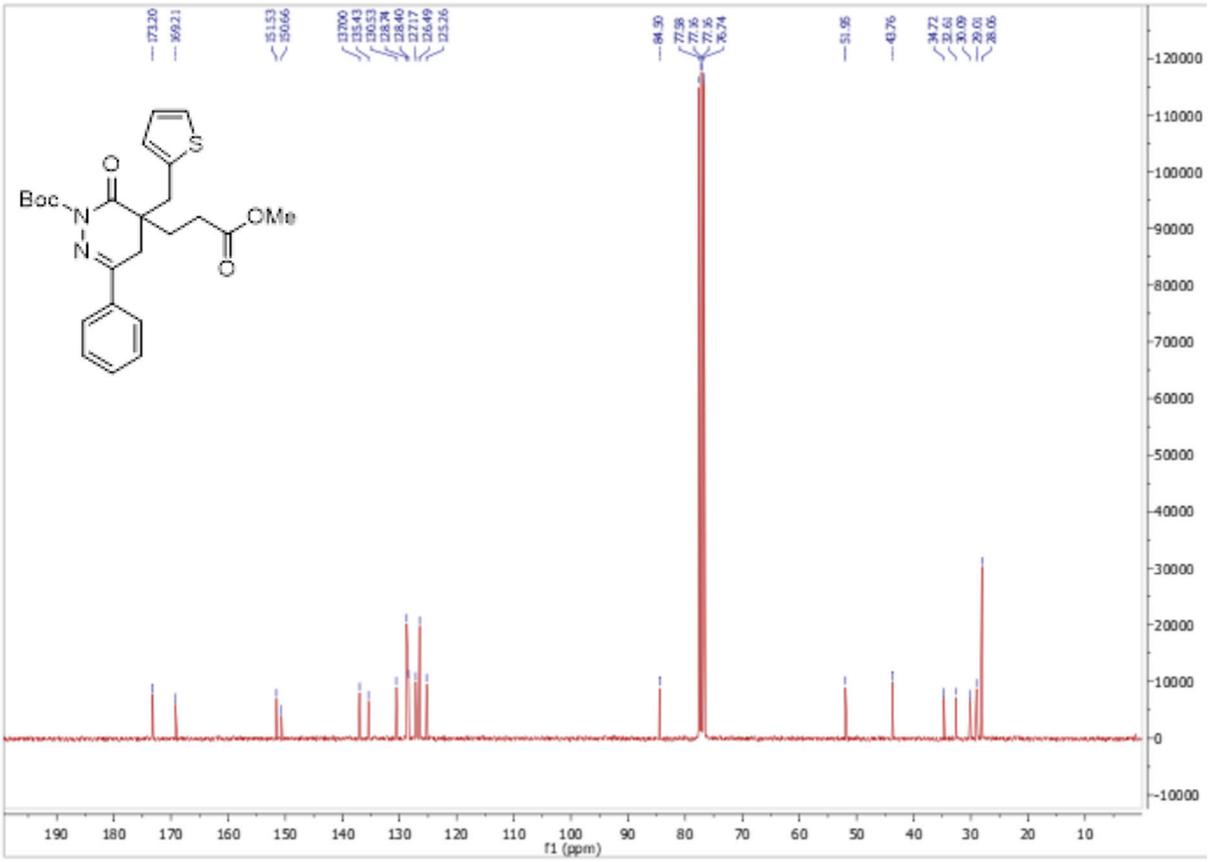

***tert*-butyl 5-isobutyl-5-(3-methoxy-3-oxopropyl)-6-oxo-3-phenyl-5,6-dihydropyridazine-1(*4H*)-carboxylate (10o)**

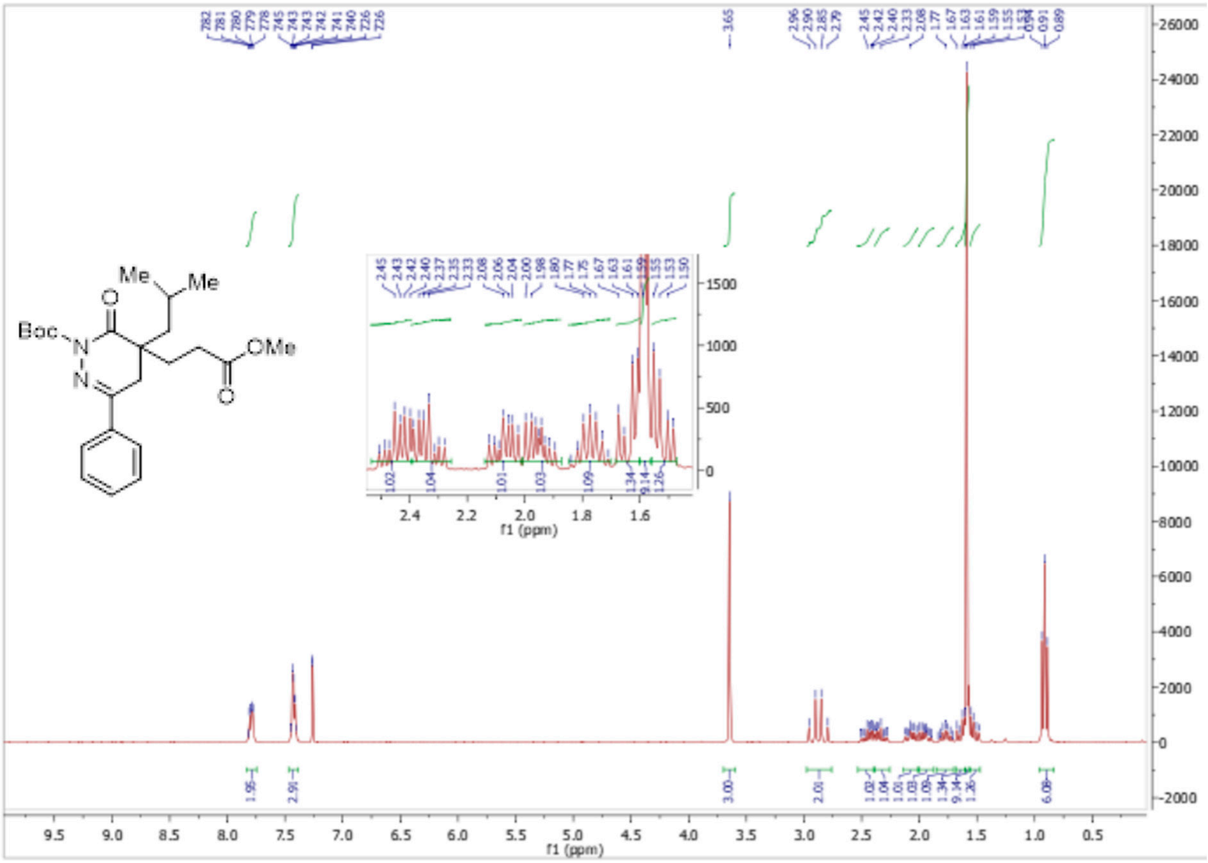

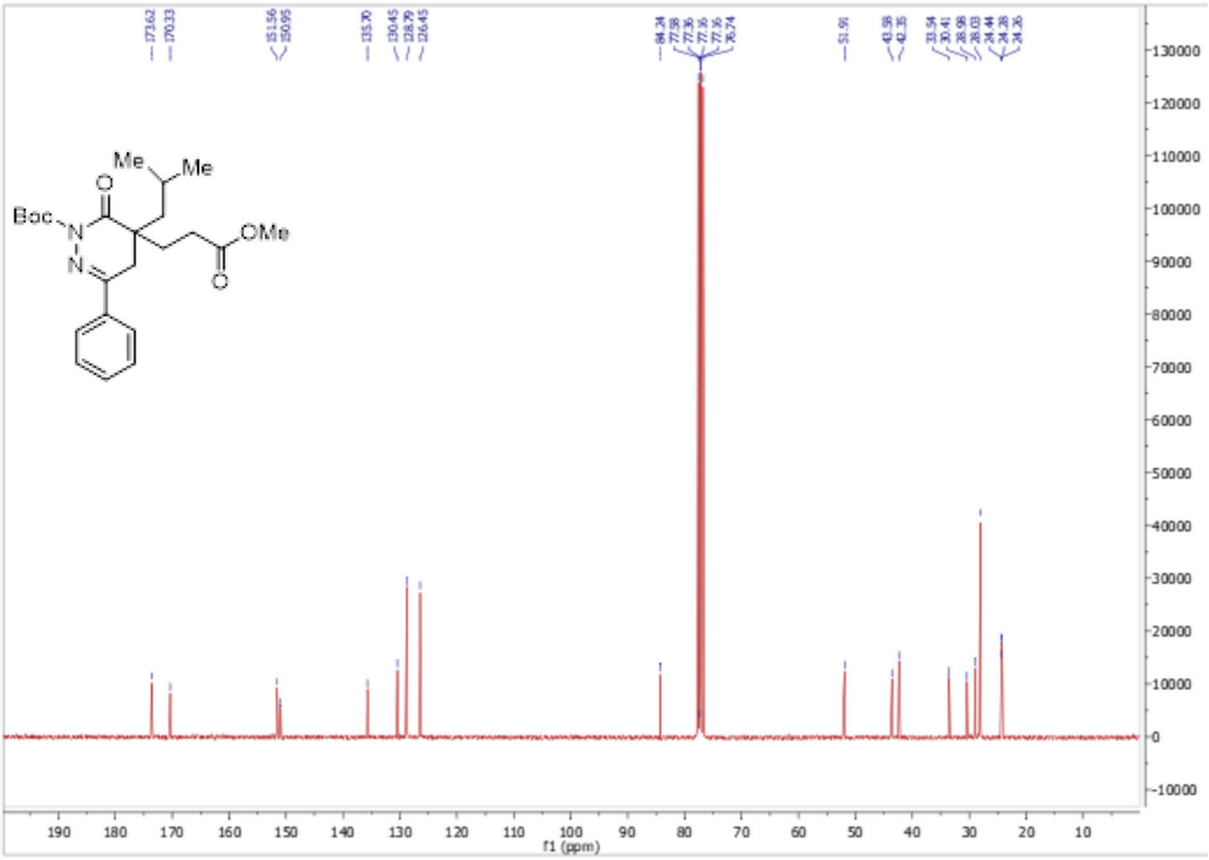

***tert*-butyl 5-(3-methoxy-3-oxopropyl)-6-oxo-3-phenyl-5-(3-phenylpropyl)-5,6-dihydropyridazine-1(*4H*)-carboxylate (10p)**

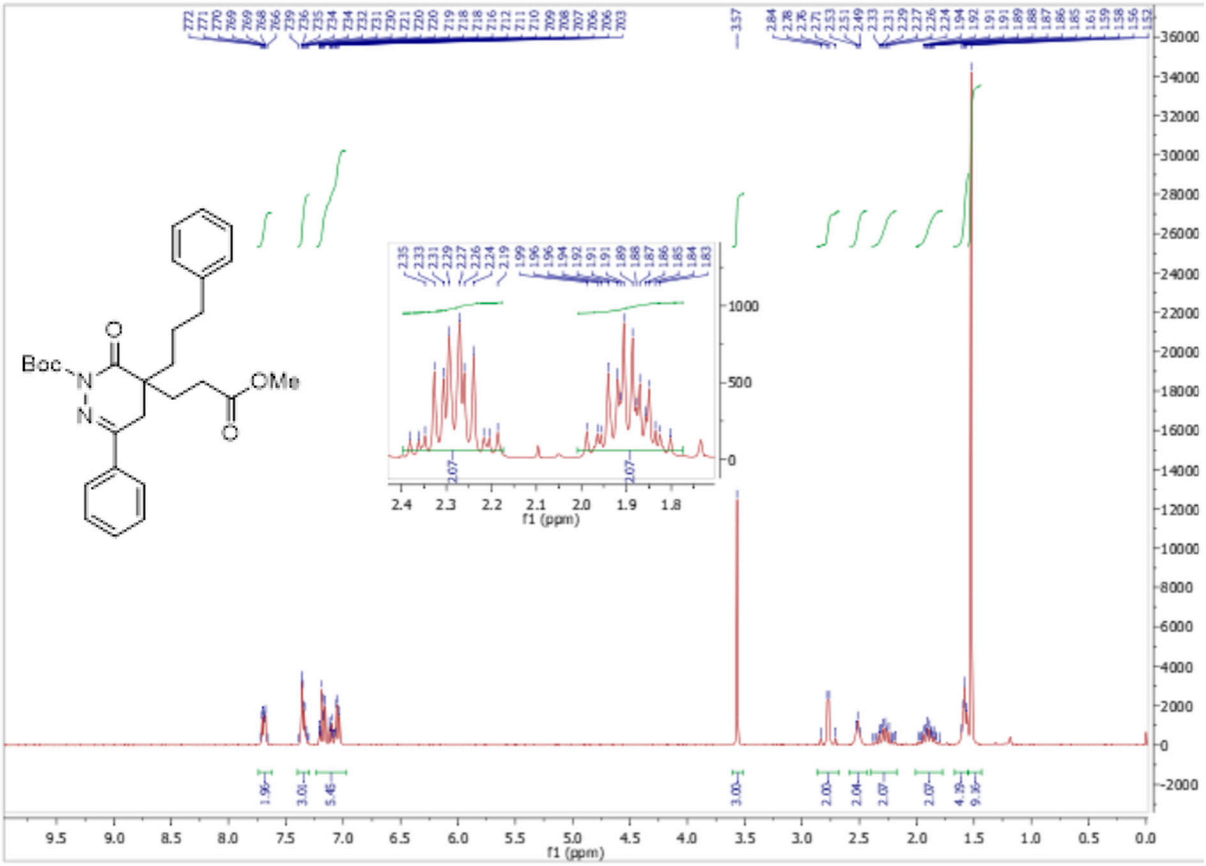

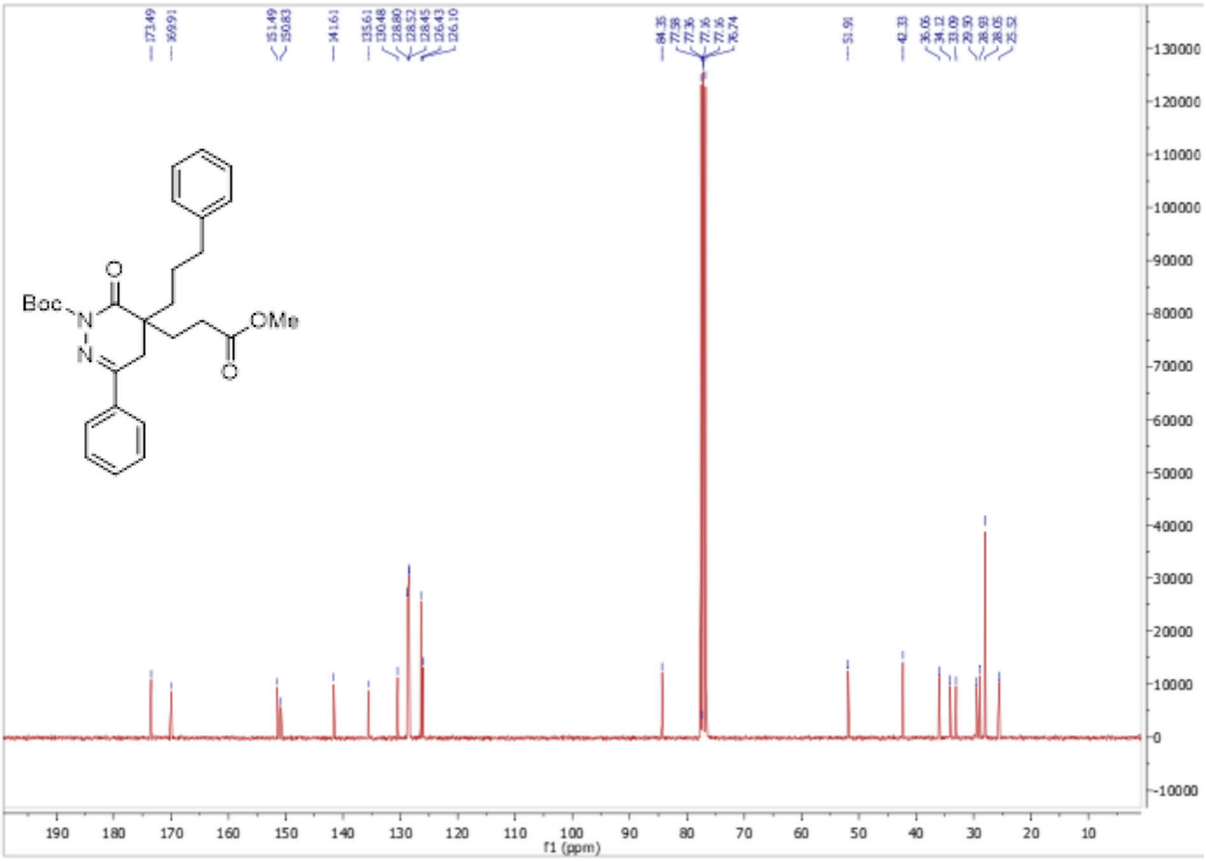

***tert*-butyl 5-(3-fluorobenzyl)-5-(3-methoxy-3-oxopropyl)-6-oxo-5,6-dihydropyridazine-1(*4H*)-carboxylate (10q)**

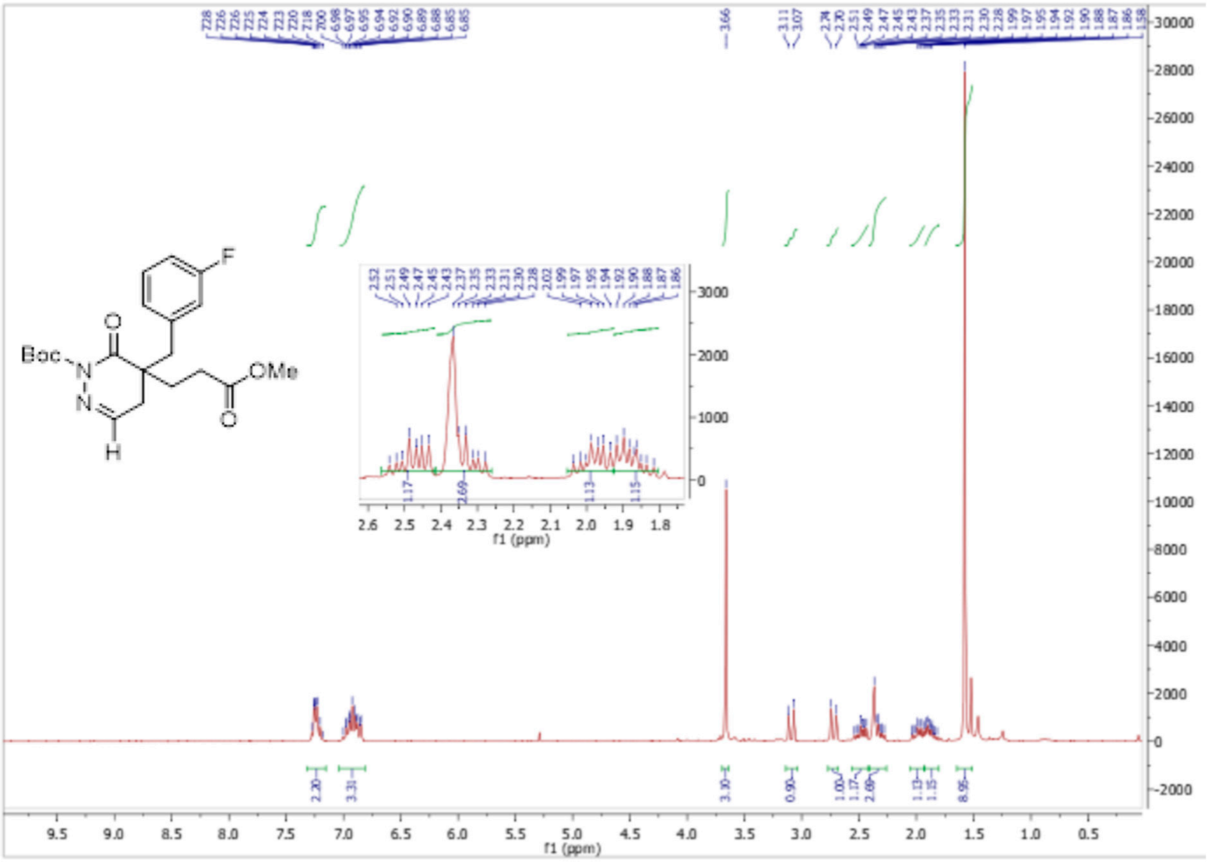

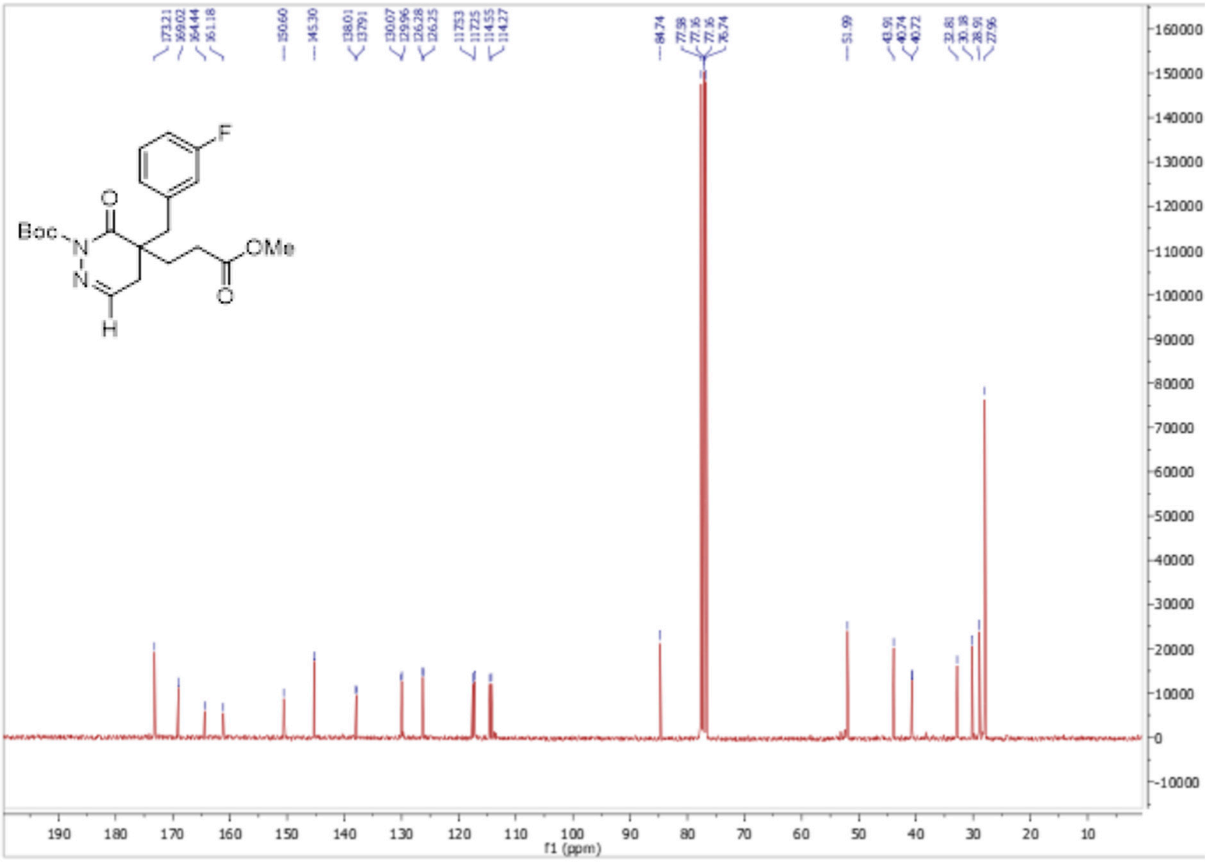

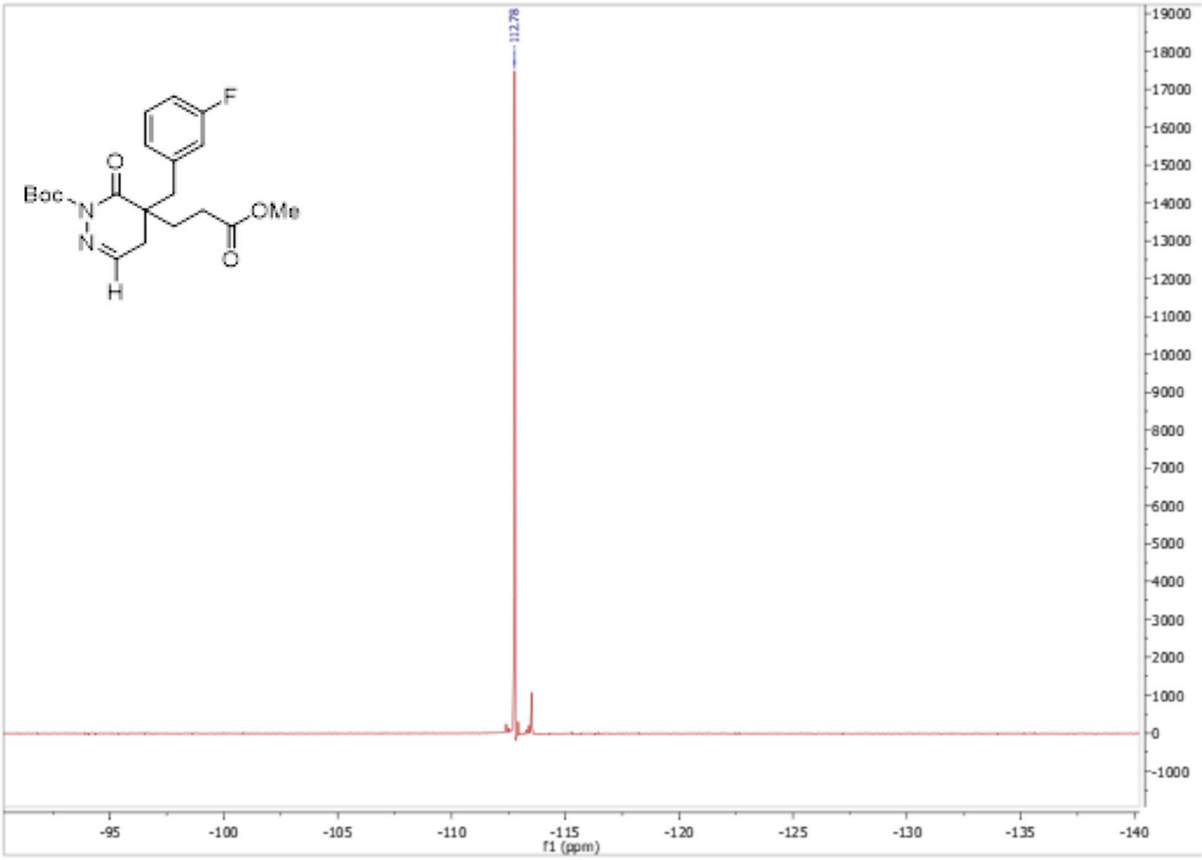

-112.78
Boc
N
N
O
H
F
OMe
O
19000
18000
17000
16000
15000
14000
13000
12000
11000
10000
9000
8000
7000
6000
5000
4000
3000
2000
1000
0
-1000
-95
-100
-105
-110
-115
-120
-125
-130
-135
-140
f1 (ppm)

***tert*-butyl 5-(3-methoxy-3-oxopropyl)-6-oxo-3,5-diphenyl-5,6-dihydropyridazine-1(*4H*)-carboxylate (10r)**

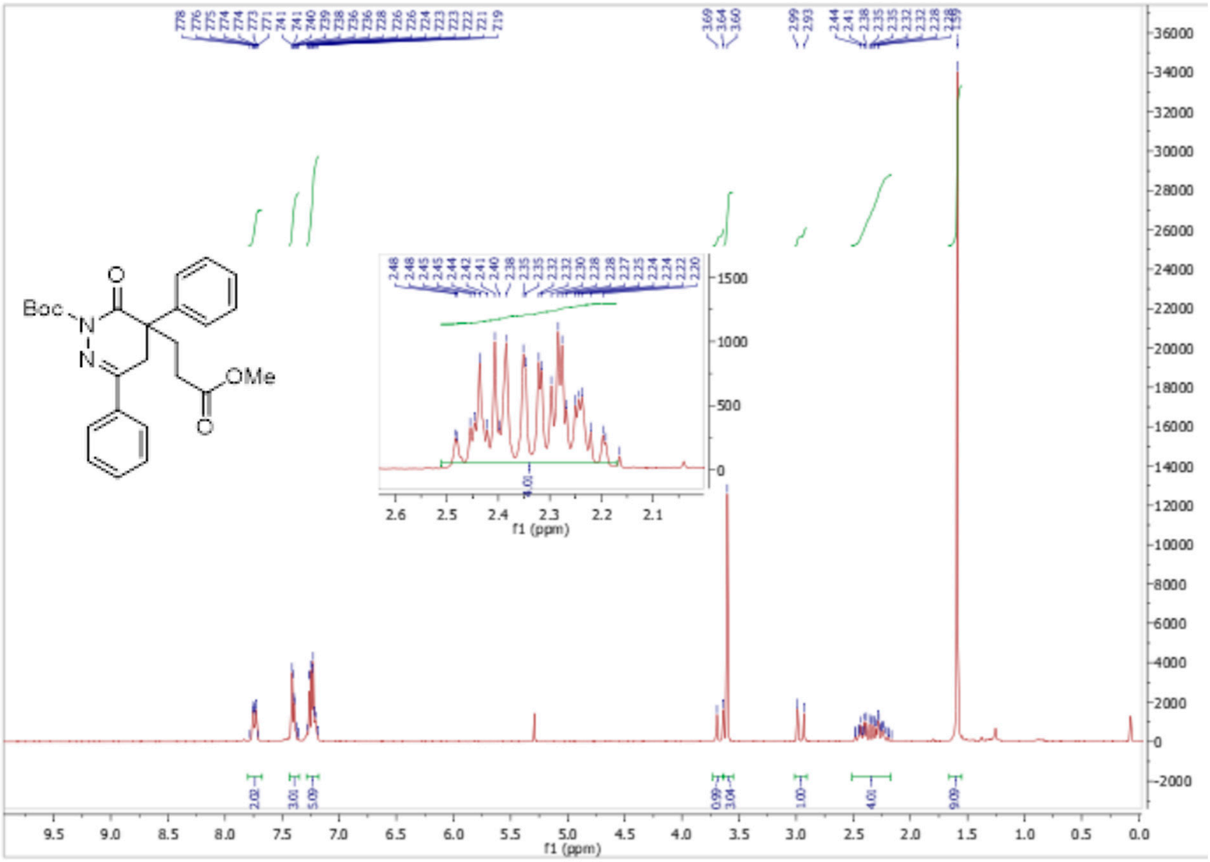

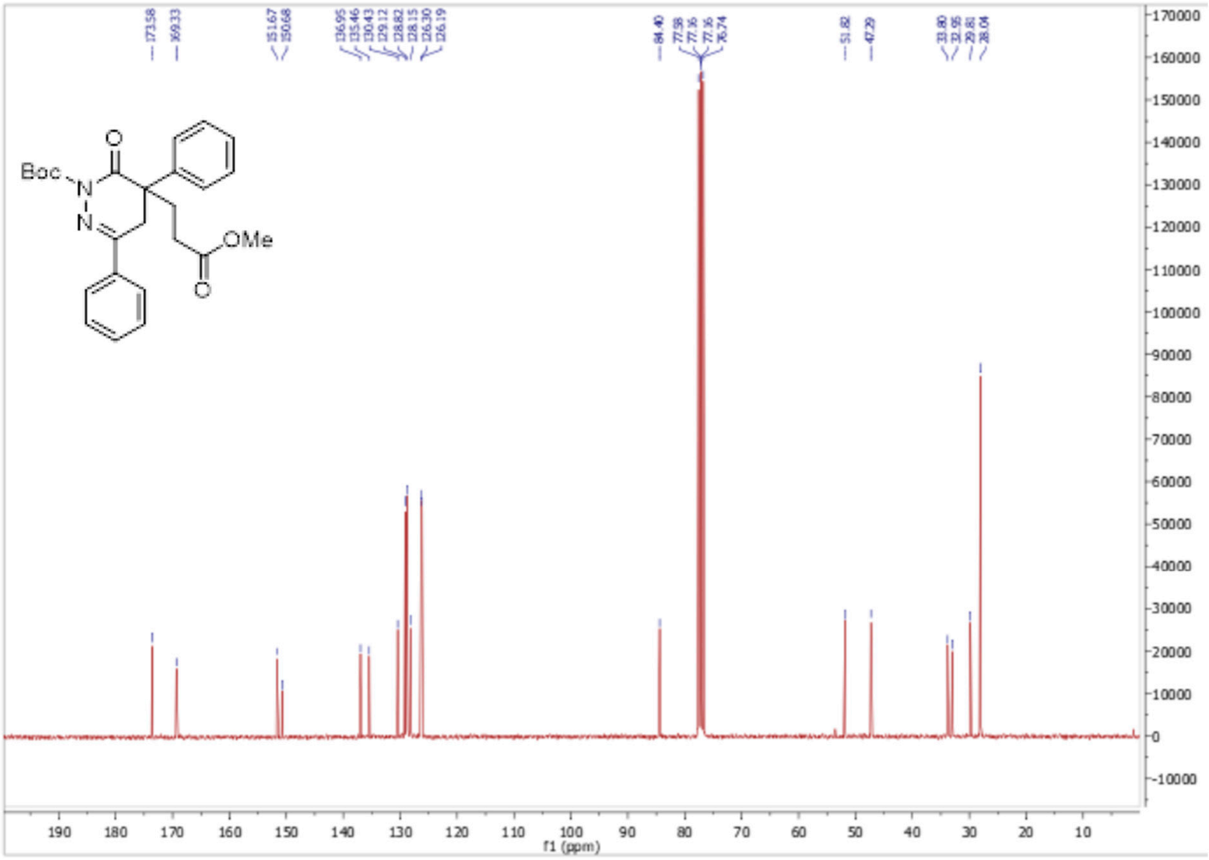

***tert*-butyl 5-((3,5-di-tert-butyl-4-hydroxyphenyl)(phenyl)methyl)-5-(3-fluorobenzyl)-6-oxo-3-phenyl-5,6-dihydropyridazine-1(*4H*)-carboxylate (12)**

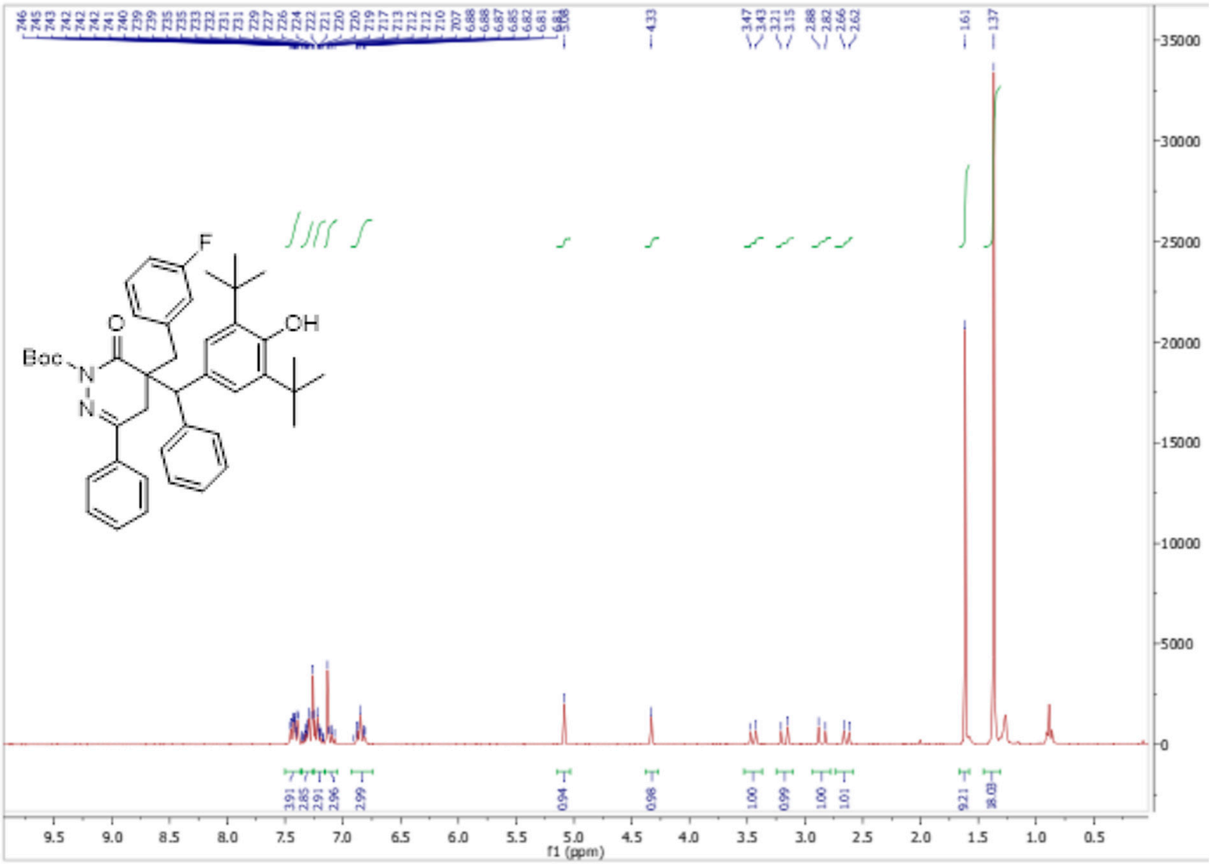

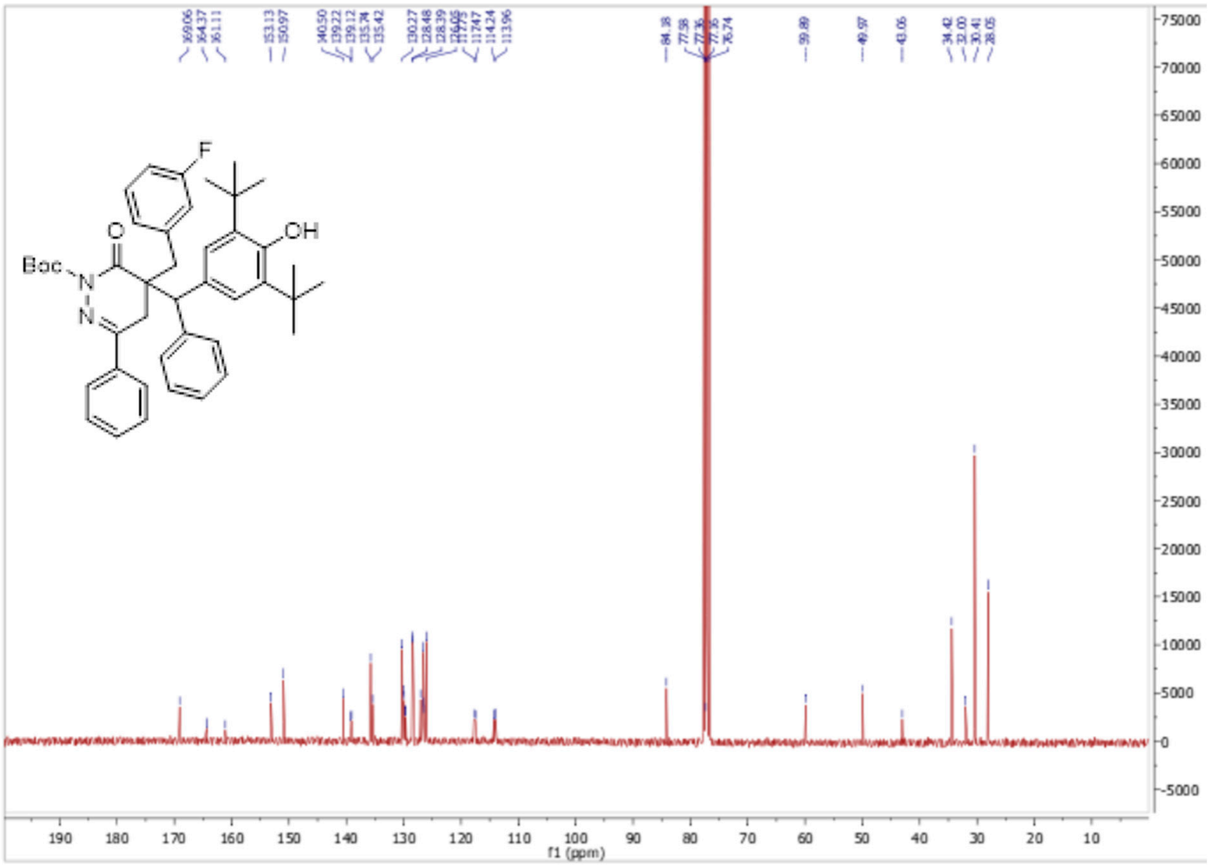

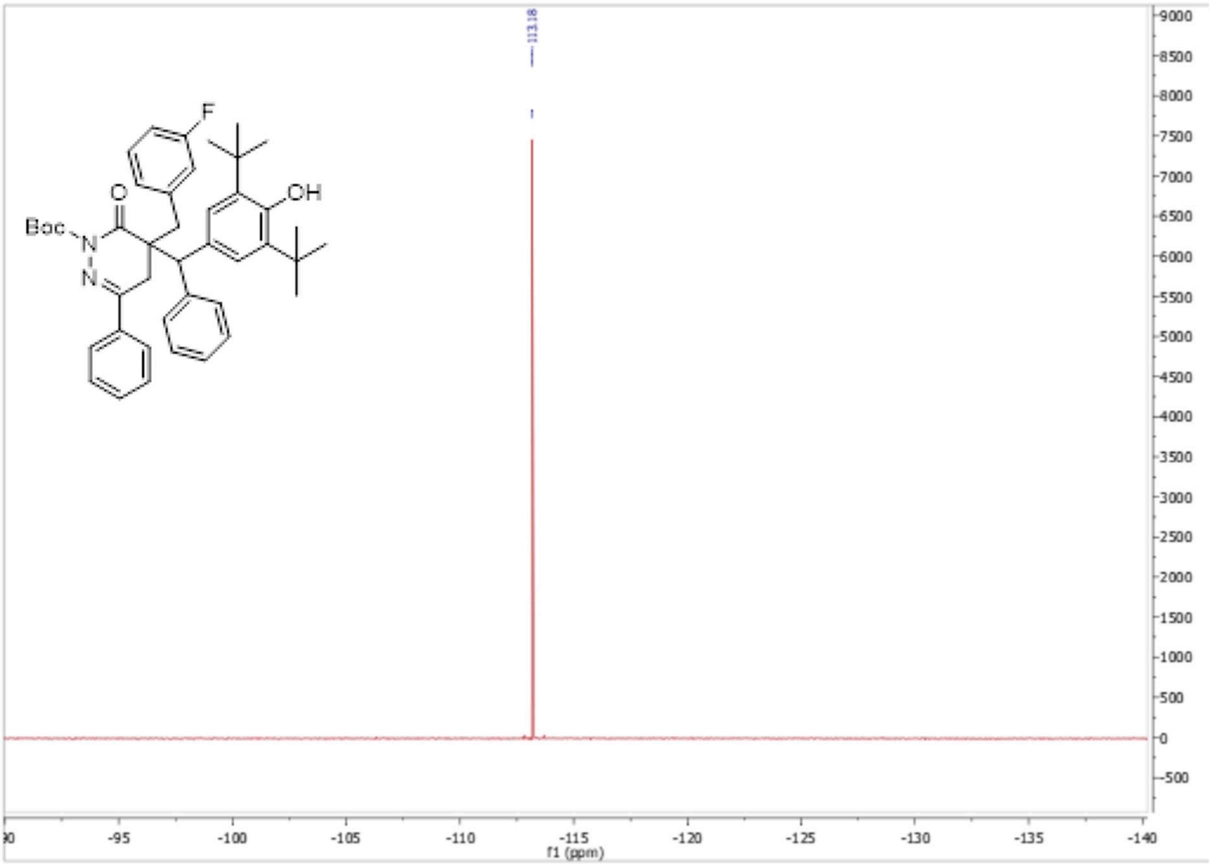

-113.18
Boc
F
OH
f1 (ppm)

V. HPLC analyses

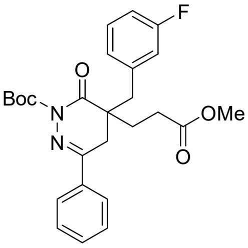

tert-butyl 5-(3-fluorobenzyl)-5-(3-methoxy-3-oxopropyl)-6-oxo-3-phenyl-5,6-dihydropyridazine-1(_4H_)-carboxylate (**10a**)

Column CHIRALPAK AD-H, hexane/iPrOH = 9:1, flow rate 1.00 mL/min, 20°C, UV 254 nm.

Racemic			19:81 er		
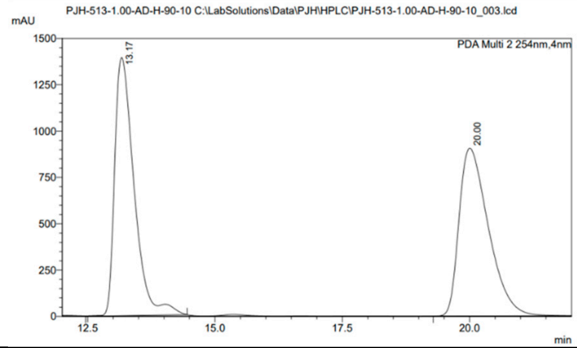			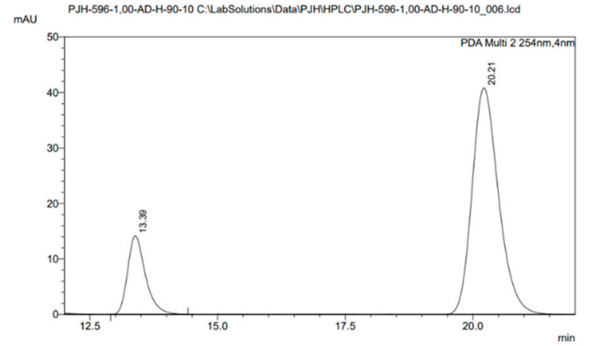		
N°	Retention time (min)	Relative area (%)	N°	Retention time (min)	Relative area (%)
1	13.8	50.39	1	13.4	19.02
2	20.0	49.61	2	20.2	80.98

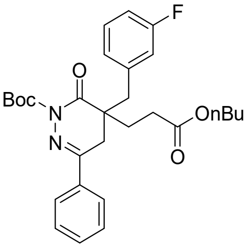

tert-butyl 5-(3-butoxy-3-oxopropyl)-5-(3-fluorobenzyl)-6-oxo-3-phenyl-5,6-dihydropyridazine-1(_4H_)-carboxylate (**10b**)

Column CHIRALPAK AD-H, hexane/_i_PrOH = 9:1, flow rate 1.00 mL/min, 20°C, UV 254 nm.

Racemic			25:75 er		
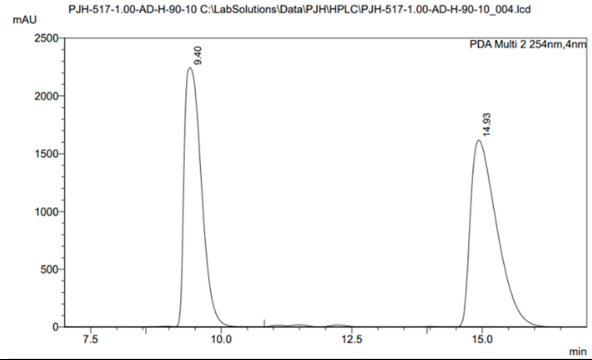			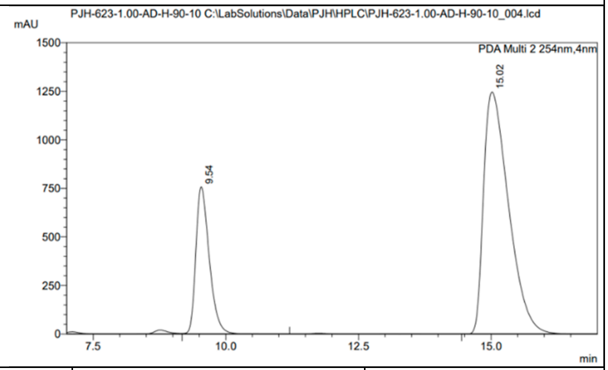		
N°	Retention time (min)	Relative area (%)	N°	Retention time (min)	Relative area (%)
1	9.4	47.51	1	9.5	24.64
2	14.9	52.49	2	15.0	75.36

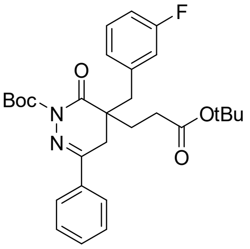

tert-butyl 5-(3-(tert-butoxy)-3-oxopropyl)-5-(3-fluorobenzyl)-6-oxo-3-phenyl-5,6-dihydropyridazine-1(*4H*)-carboxylate (10c)

Column CHIRALPAK AD-H, hexane/*i*PrOH = 98:2, flow rate 1.00 mL/min, 20°C, UV 254 nm.

Racemic			17:83 er		
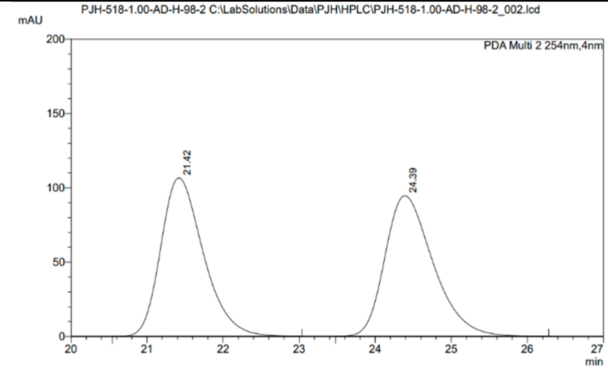			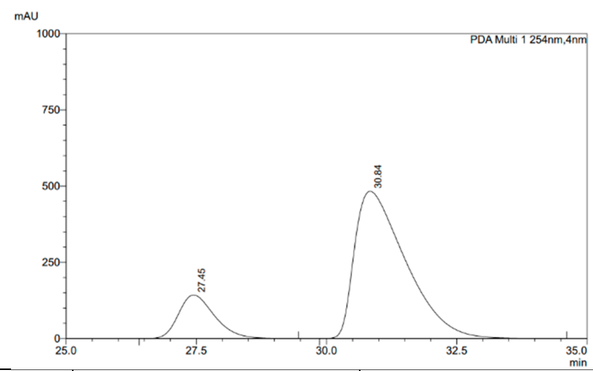		
N°	Retention time (min)	Relative area (%)	N°	Retention time (min)	Relative area (%)
1	21.4	50.01	1	27.4	17.21
2	24.4	49.99	2	30.8	82.79

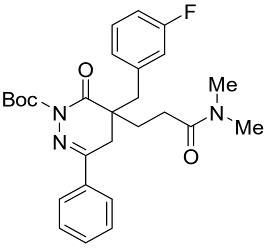

tert-butyl 5-(3-(dimethylamino)-3-oxopropyl)-5-(3-fluorobenzyl)-6-oxo-3-phenyl-5,6-dihydropyridazine-1(*4H*)-carboxylate (10d)

Column CHIRALPAK AD-H, hexane/*i*PrOH = 9:1, flow rate 1.00 mL/min, 20°C, UV 254 nm.

Racemic			32:68 er		
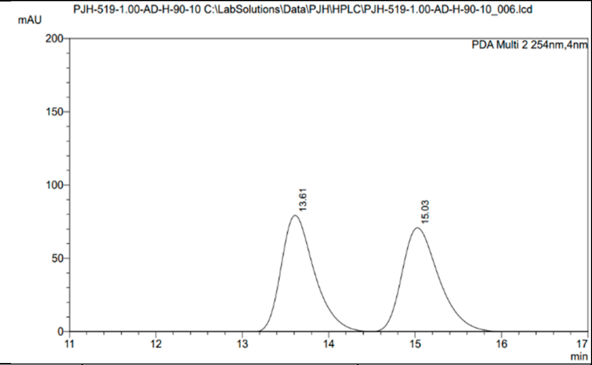			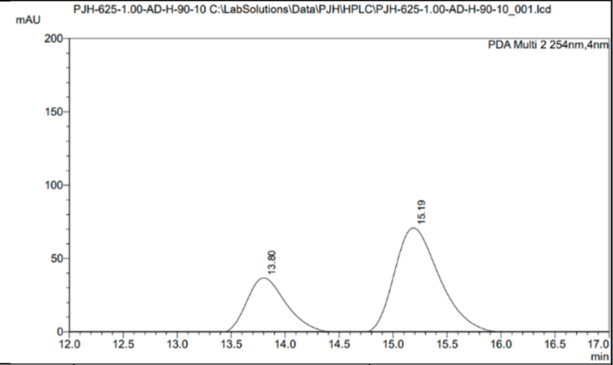		
N°	Retention time (min)	Relative area (%)	N°	Retention time (min)	Relative area (%)
1	13.6	50.01	1	13.8	32.19
2	15.0	49.99	2	15.2	67.81

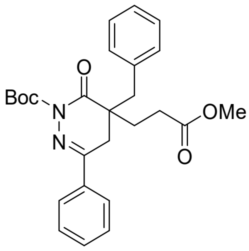

tert-butyl 5-benzyl-5-(3-methoxy-3-oxopropyl)-6-oxo-3-phenyl-5,6-dihydropyridazine-1(_4H_)-carboxylate (10e)

Column CHIRALPAK AD-H, hexane/*i*PrOH = 9:1, flow rate 1.00 mL/min, 20°C, UV 254 nm, t_{min} = 13.6 min, t_{major} = 17.5 min.

Racemic	33:67 er
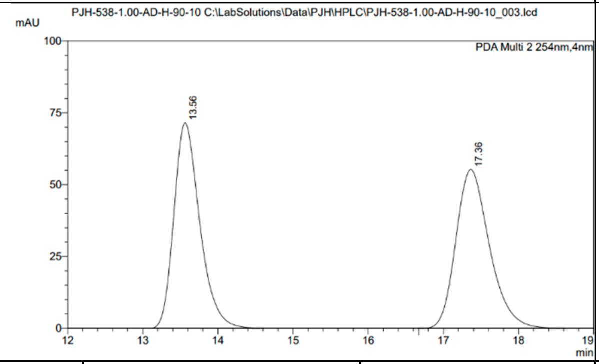	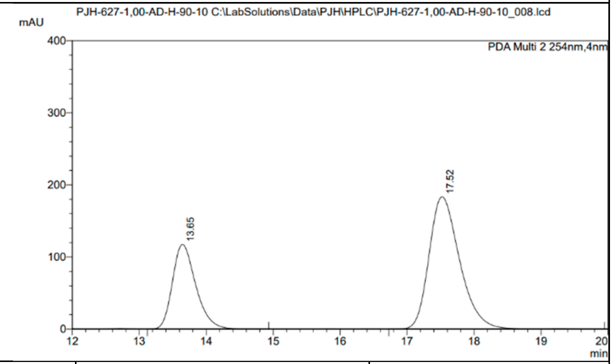

N°	Retention time (min)	Relative area (%)	N°	Retention time (min)	Relative area (%)
1	13.6	50.04	1	13.6	32.80
2	17.4	49.96	2	17.5	67.20

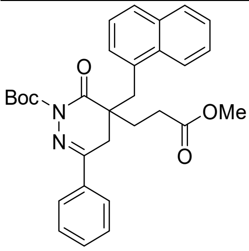

tert-butyl 5-(3-methoxy-3-oxopropyl)-5-(naphthalen-1-ylmethyl)-6-oxo-3-phenyl-5,6-dihydropyridazine-1(_4H_)-carboxylate (10f)

Column CHIRALPAK AD-H, hexane/*i*PrOH = 9:1, flow rate 1.00 mL/min, 20°C, UV 254 nm.

Racemic	30:70 er
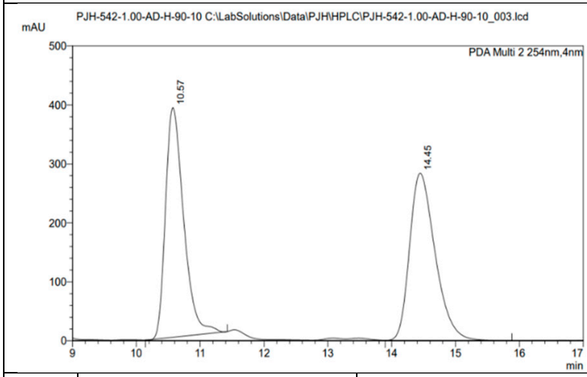	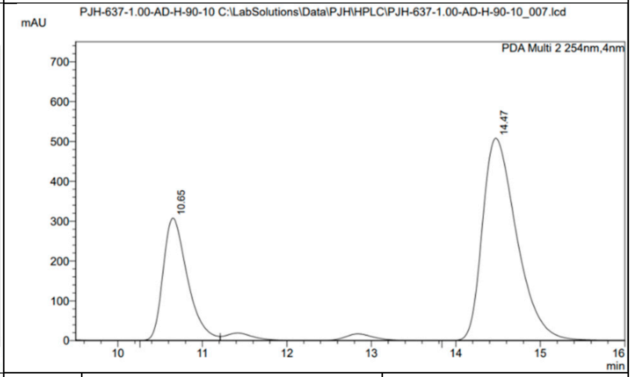

N°	Retention time (min)	Relative area (%)	N°	Retention time (min)	Relative area (%)
1	10.6	49.38	1	10.6	29.99
2	14.5	50.62	2	14.5	70.01

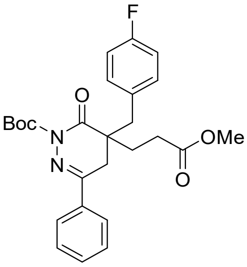

***tert*-butyl 5-(4-fluorobenzyl)-5-(3-methoxy-3-oxopropyl)-6-oxo-3-phenyl-5,6-dihydropyridazine-1(*4H*)-carboxylate** (**10g**)

Column CHIRALPAK AD-H, hexane/iPrOH = 9:1, flow rate 1.00 mL/min, 20°C, UV 254 nm.

Racemic	23:77 er

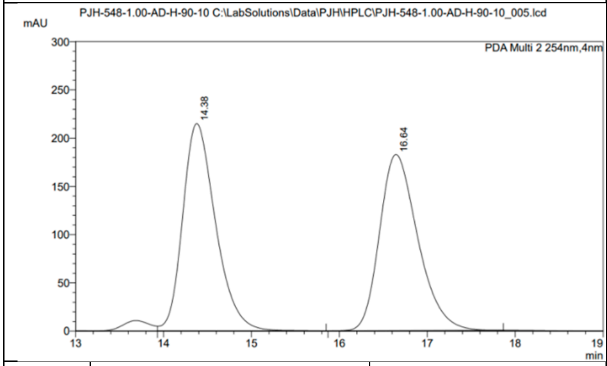

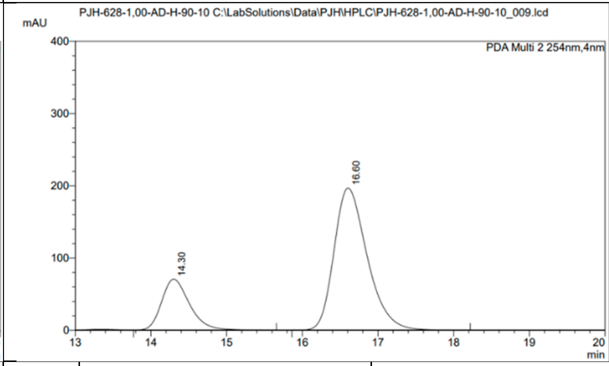

N°	Retention time (min)	Relative area (%)	N°	Retention time (min)	Relative area (%)
1	14.4	50.26	1	14.3	23.25
2	16.6	16.64	2	16.6	76.75

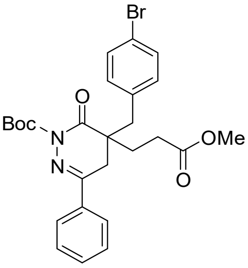

***tert*-butyl 5-(4-bromobenzyl)-5-(3-methoxy-3-oxopropyl)-6-oxo-3-phenyl-5,6-dihydropyridazine-1(*4H*)-carboxylate** (**10h**)

Column CHIRALPAK AD-H, hexane/iPrOH = 9:1, flow rate 1.00 mL/min, 20°C, UV 254 nm.

Racemic	27:73 er

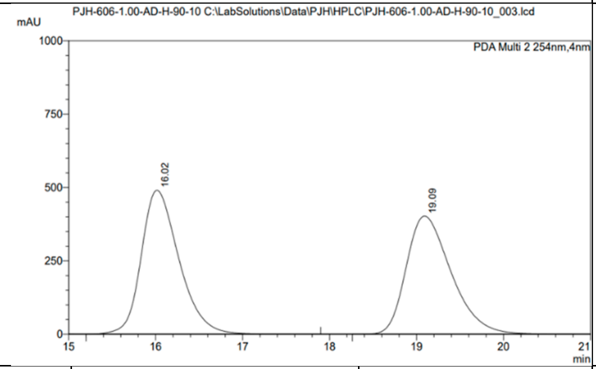

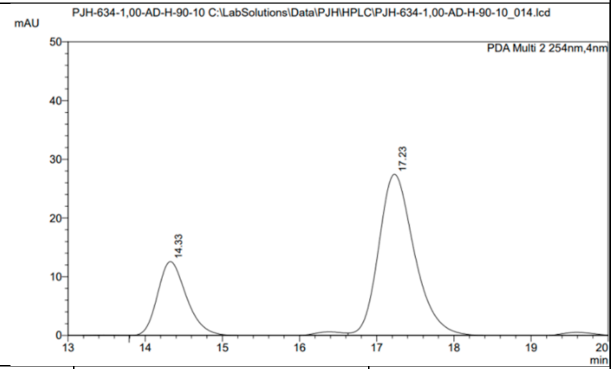

N°	Retention time (min)	Relative area (%)	N°	Retention time (min)	Relative area (%)
1	16.0	50.62	1	14.3	27.04
2	19.1	49.38	2	17.2	72.96

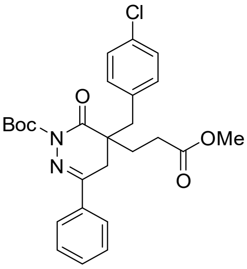

tert-butyl 5-(4-chlorobenzyl)-5-(3-methoxy-3-oxopropyl)-6-oxo-3-phenyl-5,6-dihydropyridazine-1(_4H_)-carboxylate (**10i**)

Column CHIRALPAK AD-H, hexane/iPrOH = 9:1, flow rate 1.00 mL/min, 20°C, UV 254 nm.

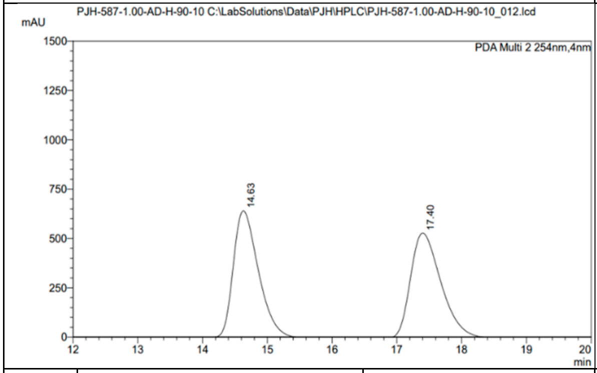

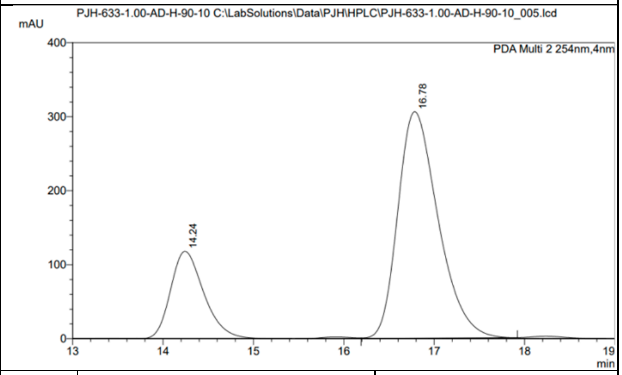

Racemic			24:76 er		
N°	Retention time (min)	Relative area (%)	N°	Retention time (min)	Relative area (%)
1	14.6	50.14	1	14.2	24.30
2	17.4	49.86	2	16.8	75.70

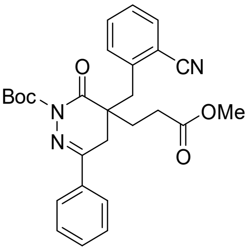

tert-butyl 5-(2-cyanobenzyl)-5-(3-methoxy-3-oxopropyl)-6-oxo-3-phenyl-5,6-dihydropyridazine-1(_4H_)-carboxylate (**10j**)

Column CHIRALPAK AD-H, hexane/*i*PrOH = 9:1, flow rate 1.00 mL/min, 20°C, UV 254 nm.

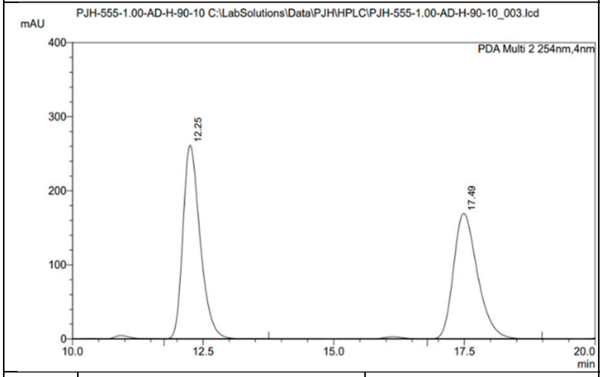

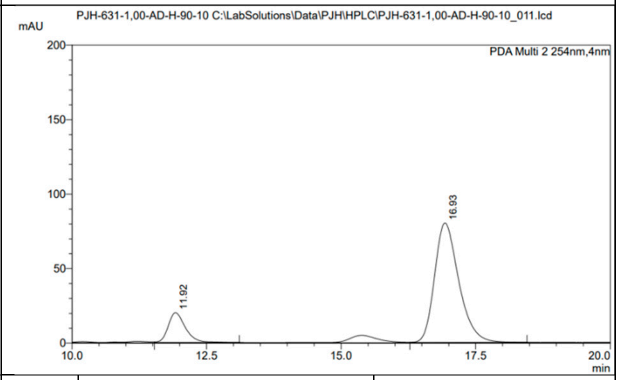

Racemic			15:85 er		
N°	Retention time (min)	Relative area (%)	N°	Retention time (min)	Relative area (%)
1	12.2	52.43	1	11.9	15.09
2	17.5	47.57	2	16.9	84.91

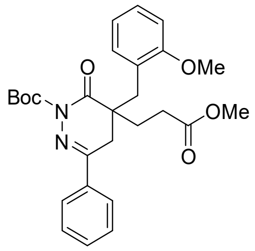

***tert*-butyl 5-(3-methoxy-3-oxopropyl)-5-(2-methoxybenzyl)-6-oxo-3-phenyl-5,6-dihydropyridazine-1(*4H*)-carboxylate** (**10k**)

Column CHIRALPAK AD-H, hexane/*i*PrOH = 9:1, flow rate 1.00 mL/min, 20°C, UV 254 nm.

Racemic			39:61 er		
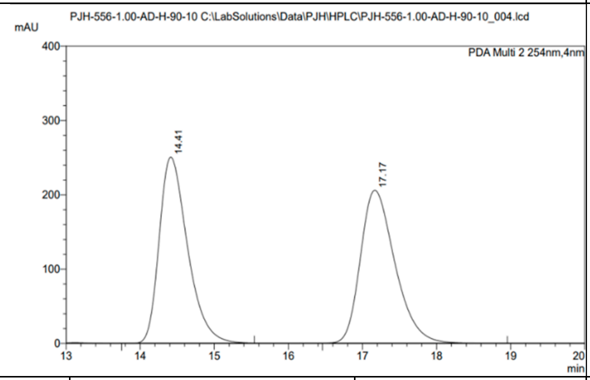			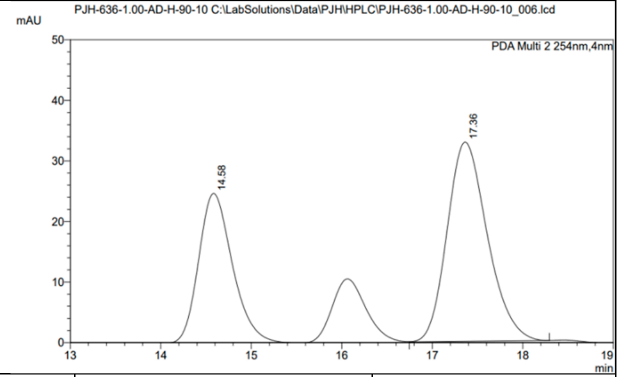		
N°	Retention time (min)	Relative area (%)	N°	Retention time (min)	Relative area (%)
1	14.4	49.88	1	14.6	38.82
2	17.2	50.12	2	17.4	61.18

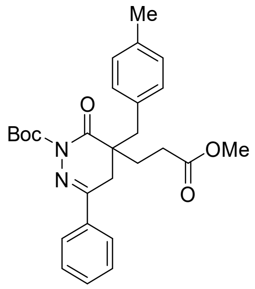

***tert*-butyl 5-(3-methoxy-3-oxopropyl)-5-(4-methylbenzyl)-6-oxo-3-phenyl-5,6-dihydropyridazine-1(*4H*)-carboxylate** (**10l**)

Column CHIRALPAK AD-H, hexane/*i*PrOH = 9:1, flow rate 1.00 mL/min, 20°C, UV 254 nm.

Racemic			36:64 er		
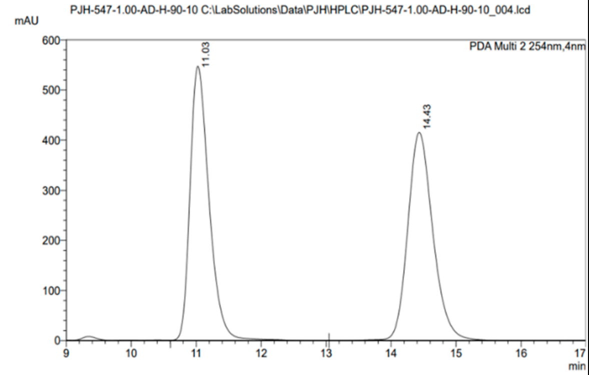			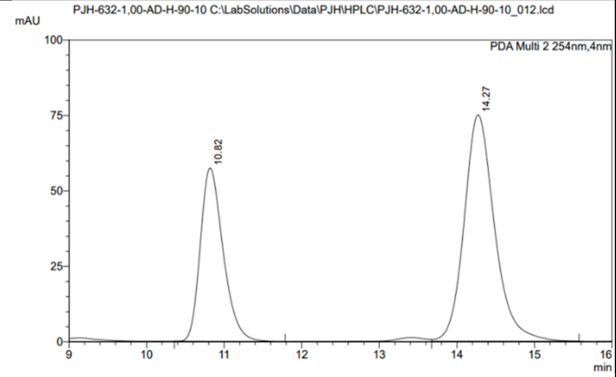		
N°	Retention time (min)	Relative area (%)	N°	Retention time (min)	Relative area (%)
1	11.0	49.81	1	10.8	36.48
2	14.4	50.19	2	14.3	63.52

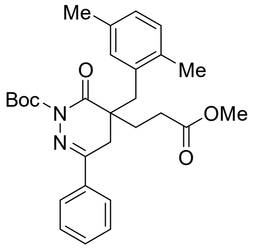

tert-butyl 5-(2,5-dimethylbenzyl)-5-(3-methoxy-3-oxopropyl)-6-oxo-3-phenyl-5,6-dihydropyridazine-1(_4H_)-carboxylate (10m)

Column CHIRALPAK AD-H, hexane/*i*PrOH = 9:1, flow rate 1.00 mL/min, 20°C, UV 254 nm.

<table>
<tr><td colspan="3">Racemic</td><td colspan="3">33:67 er</td></tr>
<tr><td colspan="3">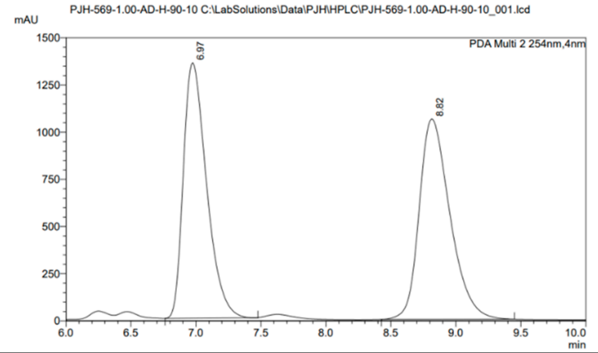
</td><td colspan="3">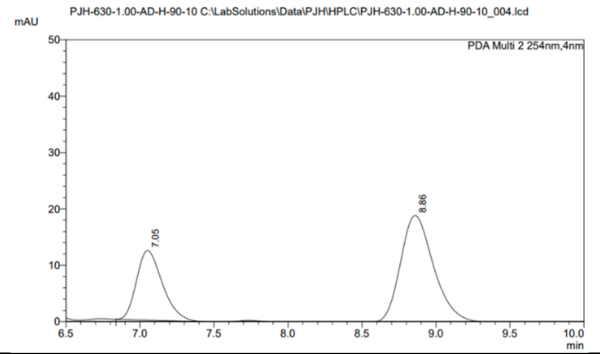
</td></tr>
<tr><td>N°</td><td>Retention time (min)</td><td>Relative area (%)</td><td>N°</td><td>Retention time (min)</td><td>Relative area (%)</td></tr>
<tr><td>1</td><td>7.0</td><td>48.98</td><td>1</td><td>7.0</td><td>33.35</td></tr>
<tr><td>2</td><td>8.8</td><td>51.02</td><td>2</td><td>8.9</td><td>66.65</td></tr>
</table>

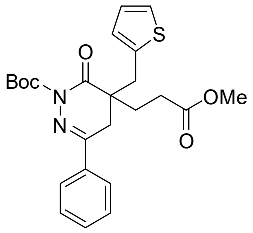

tert-butyl 5-(3-methoxy-3-oxopropyl)-6-oxo-3-phenyl-5-(thiophen-2-ylmethyl)-5,6-dihydropyridazine-1(_4H_)-carboxylate (10n)

Column CHIRALPAK AD-H, hexane/*i*PrOH = 9:1, flow rate 1.00 mL/min, 20°C, UV 254 nm, t_{min} = 16.2 min, t_{major} = 19.9 min.

<table>
<tr><td colspan="3">Racemic</td><td colspan="3">33:67 er</td></tr>
<tr><td colspan="3">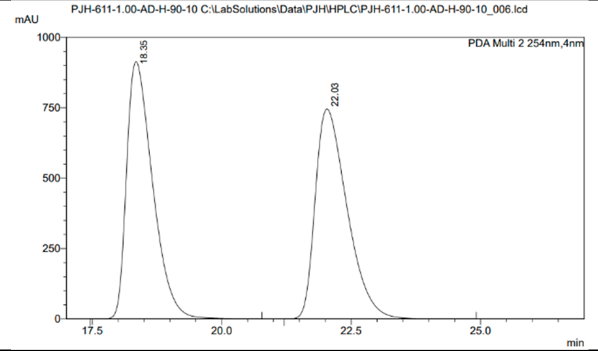
</td><td colspan="3">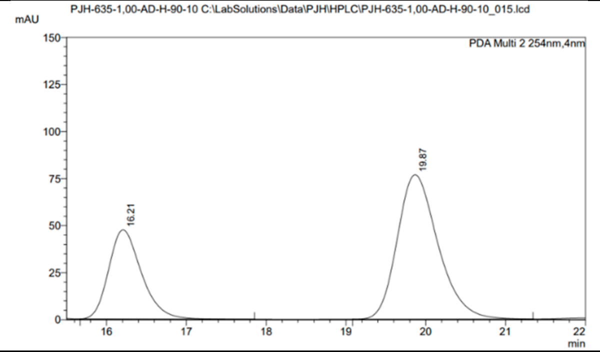
</td></tr>
<tr><td>N°</td><td>Retention time (min)</td><td>Relative area (%)</td><td>N°</td><td>Retention time (min)</td><td>Relative area (%)</td></tr>
<tr><td>1</td><td>18.3</td><td>50.04</td><td>1</td><td>16.2</td><td>33.17</td></tr>
<tr><td>2</td><td>22.0</td><td>49.96</td><td>2</td><td>19.9</td><td>66.83</td></tr>
</table>

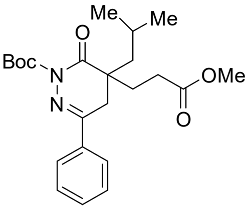

tert-butyl 5-isobutyl-5-(3-methoxy-3-oxopropyl)-6-oxo-3-phenyl-5,6-dihydropyridazine-1(4H)-carboxylate (10o)

Column CHIRALPAK AD-H, hexane/*i*PrOH = 9:1, flow rate 1.00 mL/min, 20°C, UV 254 nm.

Racemic

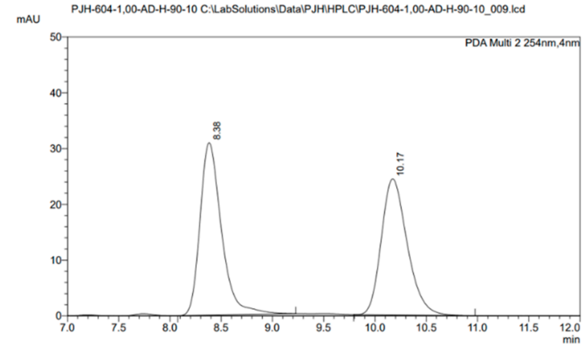

45:55 er

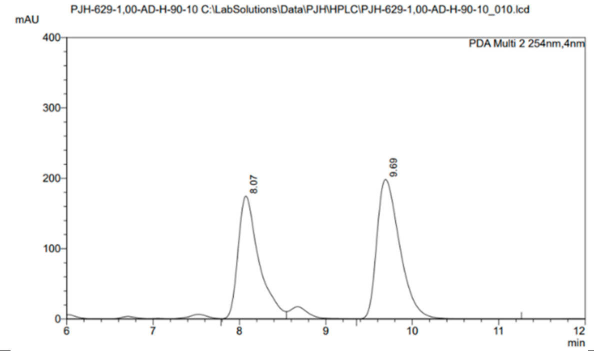

N°	Retention time (min)	Relative area (%)	N°	Retention time (min)	Relative area (%)
1	8.4	51.13	1	8.1	44.57
2	10.2	48.87	2	9.7	55.43

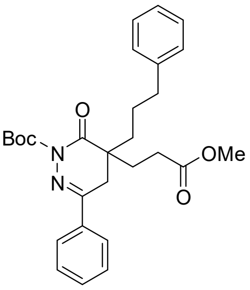

tert-butyl 5-(3-methoxy-3-oxopropyl)-6-oxo-3-phenyl-5-(3-phenylpropyl)-5,6-dihydropyridazine-1(4H)-carboxylate (10p)

Column CHIRALPAK AD-H, hexane/*i*PrOH = 9:1, flow rate 1.00 mL/min, 20°C, UV 254 nm.

Racemic

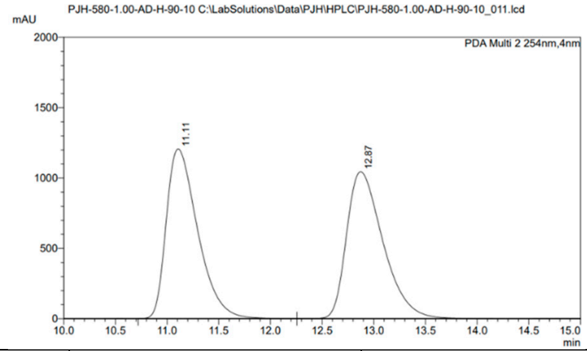

59:41 er

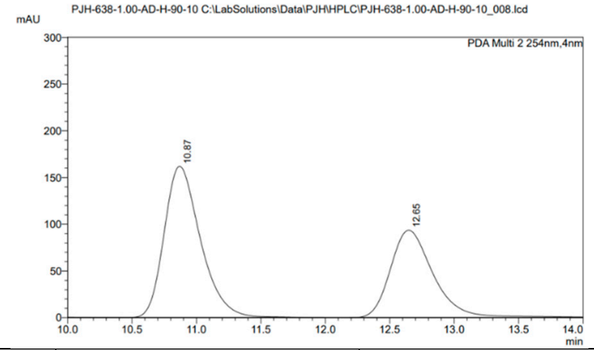

N°	Retention time (min)	Relative area (%)	N°	Retention time (min)	Relative area (%)
1	11.1	49.85	1	10.9	58.52
2	12.9	50.15	2	12.6	41.48

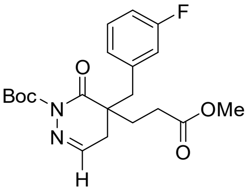

***tert*-butyl 5-(3-fluorobenzyl)-5-(3-methoxy-3-oxopropyl)-6-oxo-5,6-dihydropyridazine-1(*4H*)-carboxylate (10q)**

Column CHIRALPAK AD-H, hexane/*i*PrOH = 9:1, flow rate 1.00 mL/min, 20°C, UV 254 nm.

Racemic			78:22 er		
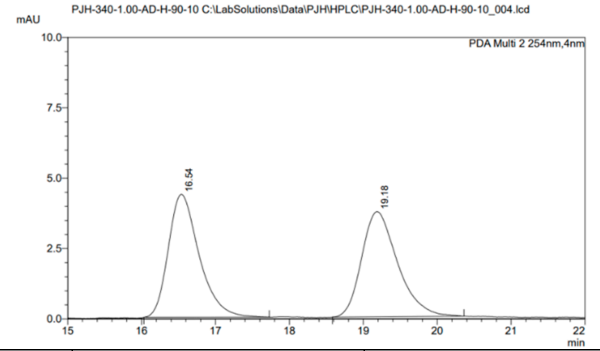			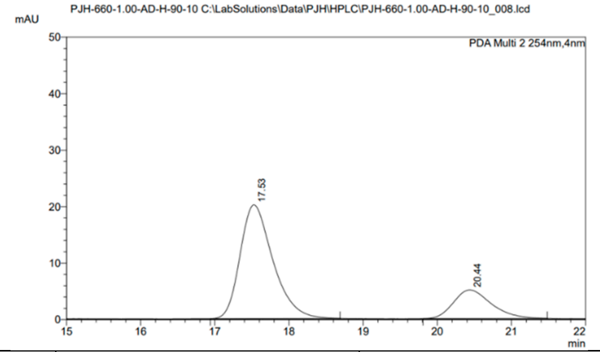		
N°	Retention time (min)	Relative area (%)	N°	Retention time (min)	Relative area (%)
1	16.4	50.07	1	17.5	77.59
2	19.2	49.93	2	20.4	22.41

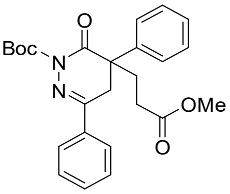

***tert*-butyl 5-(3-methoxy-3-oxopropyl)-6-oxo-3,5-diphenyl-5,6-dihydropyridazine-1(*4H*)-carboxylate (10r)**

Column CHIRALPAK AD-H, hexane/*i*PrOH = 8:2, flow rate 1.00 mL/min, 20°C, UV 254 nm.

Racemic			12:88 er		
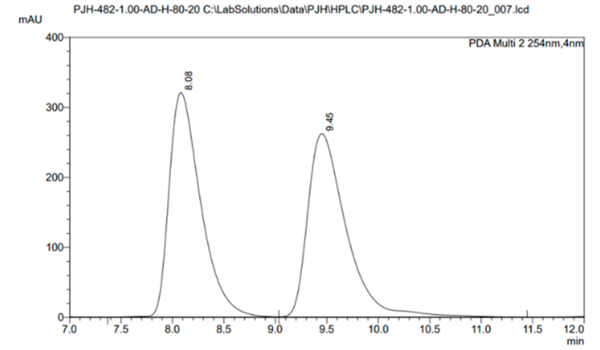			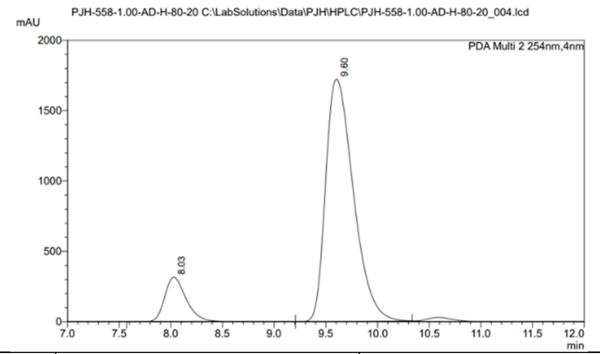		
N°	Retention time (min)	Relative area (%)	N°	Retention time (min)	Relative area (%)
1	8.1	50.19	1	8.0	12.50
2	9.4	49.81	2	9.6	87.50

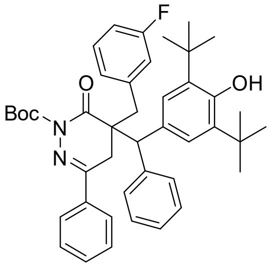

tert-butyl 5-((3,5-di-tert-butyl-4-hydroxyphenyl)(phenyl)methyl)-5-(3-fluorobenzyl)-6-oxo-3-phenyl-5,6-dihydropyridazine-1(_4H_)-carboxylate (**12**)

Column CHIRALPAK AD-H, hexane/*i*PrOH = 100:1, flow rate 1.00 mL/min, 20°C, UV 254 nm.

Racemic

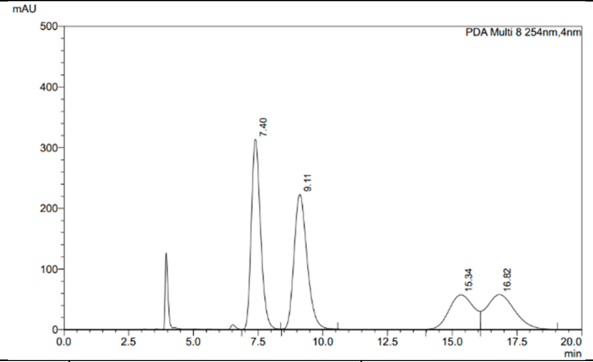

8:92 er

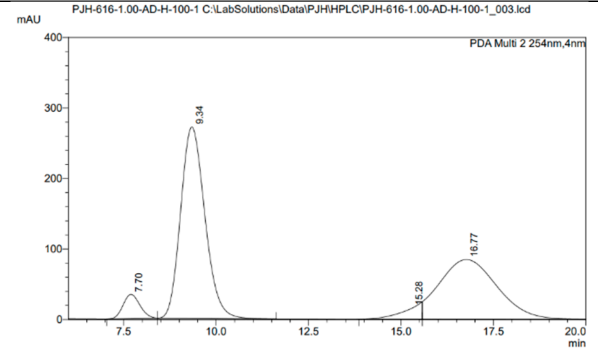

N°	Retention time (min)	Relative area (%)	N°	Retention time (min)	Relative area (%)
1	7.4	32.93	1	7.7	4.63
2	9.1	32.77	2	9.3	52.96
3	15.3	15.93	3	15.3	3.29
4	16.8	18.38	4	16.8	39.11
